# Supplementary material for: Assessment of simulation-based inference methods for stochastic compartmental models in epidemiological research
Source: PLoS One. 2026 Jul 13;21(7):e0353306. doi: 10.1371/journal.pone.0353306 (PMC13362117; doi:10.1371/journal.pone.0353306)
Supplement: S1 Text — (PDF) [file pone.0353306.s001.pdf]

S1 Supporting Information
Assessment of Simulation-based Inference Methods for Stochastic Compartmental Models in Epidemiological Research

Vincent Wieland[1,2,◕,ID], Nils Waßmuth[1,2,3,◕,ID], Lorenzo Contento[1,ID], Martin Kühn[1,2,3,ID], and Jan Hasenauer[1,2,*,ID]

[1]Bonn Center for Mathematical Life Sciences, University of Bonn, Bonn, Germany
[2]Life and Medical Science Institute, University of Bonn, Bonn, Germany
[3]Institute of Software Technology, Department for High-Performance Computing, German Aerospace Center (DLR), Cologne, Germany
◕These authors contributed equally to the work.
*To whom correspondence should be addressed; jan.hasenauer@uni-bonn.de.

June 26, 2026

Contents

S1.1 Inference Algorithms . . . 2
S1.1.1 PMMH algorithm . . . 2
S1.1.2 Tuning of Particle Filter . . . 3
S1.1.2.1 Hyperparameters and diagnostics . . . 3
S1.1.2.2 Resampling in Importance Sampling . . . 5
S1.1.3 Hyperparameters for Conditional Normalizing Flows . . . 5
S1.1.4 HMC for SDE-based models . . . 6
S1.2 Metrics and Diagnostics . . . 7
S1.2.1 KDE Plots . . . 7
S1.2.2 Posterior Predictive Plots . . . 8
S1.2.3 1-Wasserstein distance in log-parameter space . . . 8
S1.2.4 Energy score of the posterior predictive distribution . . . 8
S1.2.5 Gelman-Rubin statistic $\hat{R}$ . . . 9
S1.2.6 Effective Sample Size . . . 9
S1.2.7 Simulation-based calibration (SBC) . . . 9
S1.2.8 Parameter-recovery plots . . . 9

S1.3 Models 10
S1.3.1 Prior Bounds and Feasibility Constraints 10
S1.3.2 General Information on Data Generation 10
S1.3.3 Computation of standard deviation for the noise model 10
S1.3.4 SIS 11
S1.3.4.1 Model Equations 11
S1.3.4.2 Prior 11
S1.3.4.3 Data generation 12
S1.3.4.4 Parameter Sets 12
S1.3.5 SIR 13
S1.3.5.1 Model Equations 13
S1.3.5.2 Prior 13
S1.3.5.3 Data generation 14
S1.3.5.4 Parameter Sets 14
S1.3.6 SEIR2V 15
S1.3.6.1 Model Equations 16
S1.3.6.2 Practical non-identifiability of the two-variant SEIR model 16
S1.3.6.3 Prior 17
S1.3.6.4 Data generation 19
S1.3.6.5 Parameter Sets 20
S1.3.7 Computation Times 23

S1.1 Inference Algorithms

S1.1.1 PMMH algorithm

Particle Filters are a general tool to replace the intractable likelihood $p(y_{t_0:t_M}|\theta)$ by an unbiased estimate $\hat{p}(y_{t_0:t_M}|\theta)$ inside a Metropolis-Hastings (MH) algorithm by using the acceptance probability

$$\min\left\{1, \frac{\pi(\theta^k)}{\pi(\theta^{k-1})}\frac{\hat{p}(y|\theta^k)}{\hat{p}(y|\theta^{k-1})}\frac{q(\theta^{k-1}|\theta^k)}{q(\theta^k|\theta^{k-1})}\right\}, \tag{1}$$

where $q(\cdot|\theta)$ denotes the parameter proposal distribution from the MH algorithm.

The PMMH algorithm with a generic Particle Filter is shown in pseudocode in Algorithm 1, where we denote the estimate to the likelihood obtained from a particle filter by $L^N_{t_M}(\theta)$.

Algorithm 1 PMMH

Require: parameter space Θ, number of iterations K, parameter proposal distribution $q(\cdot|\theta)$, initial distributions $\pi(x_0), \pi(\theta)$, Particle Filter and necessary input for it (e.g., number of particles N, observations $y_{t_0:t_M}$)

Sample $\theta^0 \sim \pi(\theta)$
Run Particle Filter to generate variables $L_{t_M}(\theta^0)$
for $k = 1$ to K **do**
  Sample $\tilde{\theta} \sim \; q(\cdot\,|\theta^{k-1})$
  Run Particle Filter to generate variables $L_{t_M}(\tilde{\theta})$
  Sample $u \sim \mathcal{U}([0,1])$
  Set $v = \log[r_{PMMH}(\theta^{k-1}, \tilde{\theta})]$ with
  $r_{PMMH}(\theta^{k-1}, \tilde{\theta}) := \frac{\pi(\tilde{\theta}) L^N_{t_M}(\tilde{\theta}) q(\theta^{k-1}|\tilde{\theta})}{\pi(\theta^{k-1}) L^N_{t_M}(\theta^{k-1}) q(\tilde{\theta}|\theta^{k-1})}$
  if $\log(u) \leq v$ **then**
    Set $\theta^k := \tilde{\theta}$
  else
    Set $\theta^k := \theta^{k-1}$
  end if
end for

S1.1.2 Tuning of Particle Filter

Particle filters combined with MCMC algorithms are convincing considering their theoretical implications, but in practice tuning the performance of these algorithm can take a lot of work. For a detailed review of efficient implementations of marginal MCMC sampling, the reader may refer to [1], a numerical study on tuning PMMH is given in Chapter 16 of [2].

S1.1.2.1 Hyperparameters and diagnostics

After deciding on a inner particle filter and an outer MCMC scheme, the single most crucial choice is the number of particles N. Although any number of particles $N \geq 1$ leaves the target density invariant and theoretically leads to the correct results, the number of particles highly affects the variance of the likelihood estimate and the algorithm's ability to converge to the target distribution. Hence, choosing N resembles the trade-off between a lower variance in the likelihood estimator, efficiency, and a higher computational cost. Based on the empirical results of experiments in [2] and theoretical considerations in [1] the number of particles should satisfy the following two criteria

- The variance of the log-likelihood estimate is less than 1.
- There exists more than one unique path of particles and their ancestors over time.

Both criteria are satisfied for the SIR and SEIR model with a particle number of 100 (Figure S1.1), when evaluated around the data generating parameters. To avoid particle degeneracy issues as much as possible in the two-variant SEIR model, we use a higher number of $N = 200$ for that model.

In addition, we evaluated the convergence of the chains based on an improvement of the traditional Gelman-Rubin $\hat{R}$ criterion [3], where a value close to 1.0 is considered good convergence. To assess

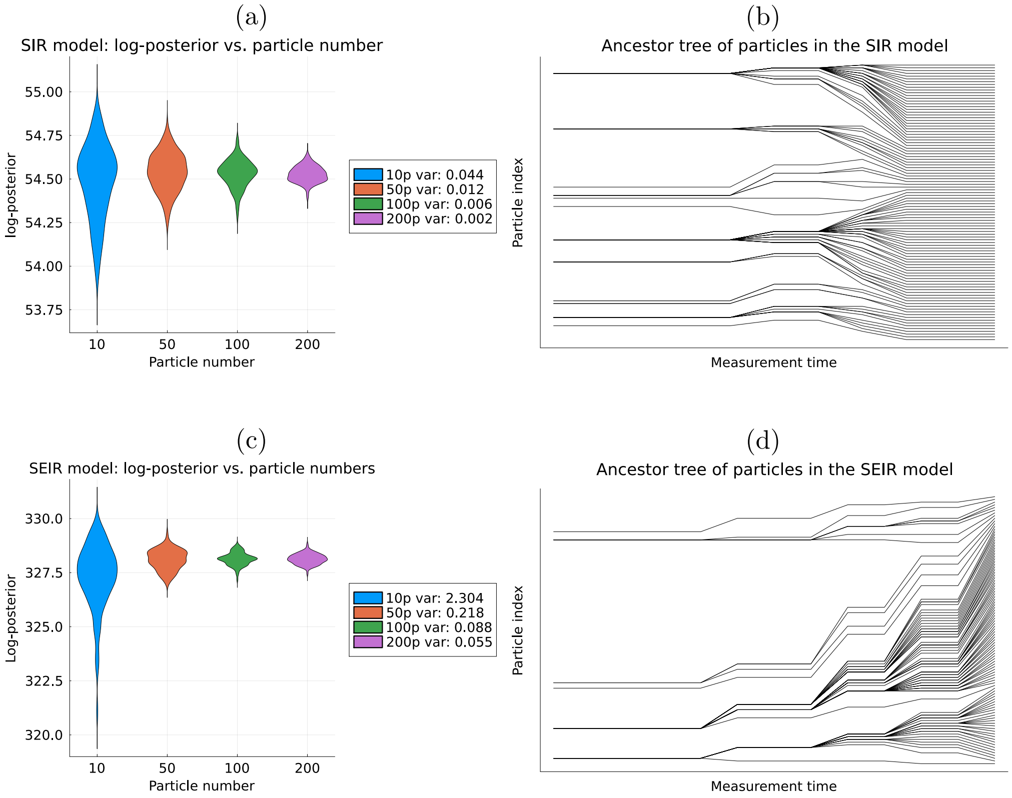

Figure S1.1: **Metrics for tuning of the particle number.** (a) and (c) visualize the variance of the log-likelihood estimate across different particle numbers for the SIR and SEIR model. (b) and (d) depict the ancestor trees of the 200 particles over time for the SIR and the SEIR model.

the mixing properties of the chain, we also monitored the effective samples size (ESS) with a maximal lag for the autocorrelation of 250. For a given estimand, it is recommended that the ESS should be at least $100 \cdot \text{nchains}$ [3]. Both criteria were evaluated using the last 10,000 samples of the chains.

S1.1.2.2 Resampling in Importance Sampling

An additional choice, that needs to be made, when using the Bootstrap filter with resampling in it, is the resampling scheme. As in importance resampling algorithms in particle filters resampling increases the variance in the past because ancestors are resampled. However, the more diverse particles decrease the variance in the present. Hence, resampling itself can be seen as sacrificing the past to save the present and is especially helpful when low weights are present. If all weights are similar, then resampling increases the variance without helping. Without resampling, the Particle Filter would fall back to a single importance sampling step and be prone to the curse of dimensionality. Conversely, the option of resampling every time introduces a step with a computational complexity of $O(N)$ at every time step and might add additional variance if the weights are all similar. Therefore, in practice, one would not resample in every iteration step, but once the variability of the weights becomes too large. Commonly, one calculates the effective sample size (ESS) to estimate this variability and triggers resampling if it falls below a certain threshold ESS_{min}, often $N/2$ is used [2].

Let $\{w^{(i)}, X^{(i)}\}_{i=1}^{N}$ be the set of weighted samples following the target distribution with density $q(x)$ and $M^{(i)}$ denote the number of offspring associated with $X^{(i)}$. The resampling process is equivalent to sampling $M^{(1:N)} = (M^{(1)}, ..., M^{(N)})$. Different schemes are proposed in the literature [4, 2] to obtain unbiased approximations via resampling, including multinomial, residual and systematic resampling. Among those, the systematic resampling is the best as it is easy to implement, very fast, and outperforms other schemes in terms of lower-variance estimators. This method performs sampling from a multinomial distribution with less variance.

Naively, one samples i.i.d. random variables $\{U_i\}_{i=1,...,N}$ from $\mathcal{U}([0, 1))$ and then applies the inverse cumulative distribution function to transform them to the desired samples $M^{(i)} = \text{cdf}^{-1}(U_i)$. A good strategy to reduce the variance of this approach is to replace the i.i.d. values with values covering $[0, 1)$ more regularly. To achieve this, one simulates a single uniformly distributed random variable $u \sim \mathcal{U}([0, \frac{1}{N}))$ and sets $U_i = u + \frac{i-1}{N}$ for $i = 1, ..., N$. Then we obtain $M^{(i)}$ by

$$M^{(i)} = \left| \left\{ U_j : \sum_{k=1}^{i-1} w^{(k)} \leq \; U_j < \sum_{k=1}^{i} w^{(k)} \right\} \right|,$$

where we use the convention $\sum_{k=1}^{0} := 0$.

For a more thorough comparison of different resampling methods, the reader may refer to [5, 6, 4].

S1.1.3 Hyperparameters for Conditional Normalizing Flows

Hyperparameters for the Normalizing-Flows workflow were mostly fixed across all experiments. Only the summary dimension was varied between models. For detailed configurations, see Tables S1.1, S1.2, S1.3, and S1.4.

Table S1.1: **Hyperparameters Summary Network:** `SequenceNetwork`

Hyperparameter	Setting
Summary Dimensions	$2 \times$ `parameter_size` $+ 2$
LSTM Units	64
Convolutional Layers	2

Table S1.2: **Invertible Network:** `InvertibleNetwork`

Coupling parameter	Setting
Coupling Type	Spline
Coupling Layers	8

S1.1.4 HMC for SDE-based models

Likelihood-based inference for discretely observed diffusion processes is challenging because the likelihood depends on the transition density, which is rarely available in closed form for nonlinear, multivariate SDEs [7]. As a consequence, "direct" MCMC on the exact marginal likelihood is typically infeasible. A standard remedy is *data augmentation* in which the unobserved diffusion path between observation times is treated as missing data and approximated by a time discretization [8, 9]. This produces an explicit (approximate) joint density over parameters and latent path increments, enabling proper MCMC—and in particular gradient-based methods such as Hamiltonian Monte Carlo (HMC)—to be applied. Different strategies to propose diffusion path segments are discussed in [10] and more recently, improved bridge constructs and guided proposals were developed [11, 12]. For the SDE model at hand we used the transition densities from the Euler-Maruyama scheme on a fine-time discretization.

We model the epidemic dynamics in terms of population fractions $x(t)$ (e.g. $x(t) = (s(t), i(t))^\top$ for SIS or $x(t) = (s(t), i(t), r(t))^\top$ for SIR) as an Itô SDE

$$dx(t) \;=\; f\big(x(t), \theta\big)\, dt \;+\; G\big(x(t), \theta\big)\, dB(t), \tag{2}$$

where θ contains epidemiological parameters (e.g. infection rate β, recovery rate γ), and $B(t)$ is a standard Brownian motion of suitable dimension.

The system is observed at irregular times $t_1 < \cdots < t_m$. First, we construct an augmented time grid $\tau_1 < \tau_2 < \cdots < \tau_K$ that contains all observation times and (optionally) intermediate points such that $\Delta\tau_k = \tau_{k+1} - \tau_k \le h_{\max}$. On this grid we approximate (2) with the Euler–Maruyama (EM) scheme:

$$x_{k+1} \;=\; x_k \;+\; f(x_k, \theta)\, \Delta\tau_k \;+\; G(x_k, \theta)\, \Delta B_k, \qquad \Delta B_k \sim \mathcal{N}(0, \Delta\tau_k I). \tag{3}$$

Writing $\Delta B_k = \sqrt{\Delta\tau_k}\, \varepsilon_k$ with $\varepsilon_k \sim \mathcal{N}(0, I)$ yields

$$x_{k+1} \;=\; x_k \;+\; f(x_k, \theta)\, \Delta\tau_k \;+\; G(x_k, \theta)\, \sqrt{\Delta\tau_k}\, \varepsilon_k. \tag{4}$$

This reparameterization makes the driving noise $\varepsilon_{1:K-1}$ explicit and i.i.d. standard Normal, so the latent path $\{x_k\}_{k=1}^K$ becomes a deterministic function of the initial condition, parameters θ, and the innovation sequence $\varepsilon_{1:K-1}$. The resulting posterior is an *approximation* to the continuous-time

Table S1.3: **Simulation Configuration.**

Hyperparameter	Setting
Training Mode	Offline
Simulations	100,000
Batch Size	32
Validation Simulations	400
Epochs	100

Table S1.4: **Calibration Setup**

Hyperparameter	Setting
Datasets	2,000
Inference Draws	100 per dataset

diffusion posterior whose accuracy improves as $h_{\max} \to 0$ [9] and crucially, for fixed (θ, ε) the latent path and the log posterior are deterministic, avoiding random simulation inside the likelihood [9].

We sample from this posterior using HMC with the No-U-Turn Sampler (NUTS) [13] as implemented in *Turing.jl*[14]. Gradients of the log posterior are obtained via automatic differentiation through the EM recursion and observation likelihood. Moreover, we ensured the convergence of the HMC chains by using standard MCMC convergence criteria as the Gelman-Rubin $\hat{R}$ statistic, autocorrelation and effective sample size.

S1.2 Metrics and Diagnostics

This section summarizes the metrics and diagnostics used to evaluate inference quality across models and datasets.

S1.2.1 KDE Plots

As a visual aid to compare multiple posteriors against each other, we opted to use 1D and 2D plots based on kernel density estimation (KDE). Plots were generated with the **seaborn** python package. The KDE plots visualize the joint and marginal posterior distributions of all model parameters. Along the diagonal, each parameter's marginal distribution is shown, allowing quick assessment of uncertainty, skewness, and whether the posterior concentrates near the true value. The off-diagonal panels display pairwise joint distributions, highlighting correlations, ridges, or identifiability issues between parameters.

Across all models, we compare multiple inference methods by overlaying their posterior samples, MAP estimates. Additionally we display reference parameter values (either true parameters used for data generation or publication parameters). This visualization allows us to diagnose:

- how well each method recovers the ground truth,
- whether posteriors are overly diffuse or overly concentrated,

- whether parameters exhibit strong posterior correlations,
- and whether the two inference methods differ systematically in bias or uncertainty.

In short, KDE plots give us a compact diagnostic for posterior geometry and method agreement.

S1.2.2 Posterior Predictive Plots

As a visual complement, we also generated plots displaying the posterior predictive distribution for our observational functions. Each subpanel represents one observational function (infection count and seroprevalence) and shows the 50%, 90% and 95% percentiles of trajectories generated from the posterior samples from each method. Additionally, it also shows the data used for inference and for the publication data from [15] with error bars depicting the standard deviation of the data.

This allows us to assess:

- how well each method's posterior predictive distribution captures the observed data.
- whether uncertainty bands are appropriately calibrated (too narrow, too wide, or misaligned),
- whether either method systematically over- or under-predicts certain phases of the epidemic,
- and how closely the inferred trajectories track the true underlying dynamics.

This plot serves as a diagnostic for predictive accuracy of the inference methods.

S1.2.3 1-Wasserstein distance in log-parameter space

To quantify discrepancies between inferred and true parameter distributions, we compute the 1-Wasserstein distance [16] between posterior samples of two methods. The distance is evaluated in log-parameter space to account for multiplicative scales and to avoid domination by parameters spanning several orders of magnitude.

The 1-Wasserstein distance has a simple heuristic interpretation: it measures the average amount of probability mass that must be moved to transform one posterior distribution into the other. A value of d means that, on average, posterior mass lies roughly a factor of $\exp(d)$ away from the second posterior.

Since the Wasserstein distance is computed from finite posterior samples, it inherits a non-negligible Monte Carlo error. This requires the usage of a proper baseline (e.g., the distance between two independent posterior draws from the same method), which reflects the intrinsic Monte Carlo noise of the estimator.

S1.2.4 Energy score of the posterior predictive distribution

Predictive performance is assessed using the energy score, a proper scoring rule for multivariate predictive distributions [17]. For each dataset, we draw 10,000 posterior predictive trajectories and compute the energy score using its standard pairwise formulation.

The pairwise term involves 10^8 trajectory pairs, which is computationally expensive. We therefore approximate it by subsampling 10,000 random pairs, which yields an unbiased Monte-Carlo estimate.

Monte Carlo error: The variance of the pairwise term decreases as $1/M$, where M is the number of sampled pairs. With $M = 10,000$, the Monte-Carlo standard error is typically well below the scale of variation across datasets, and we verified empirically that increasing M does not materially change the ranking or magnitude of energy scores. This provides a reliable and efficient approximation.

S1.2.5 Gelman-Rubin statistic $\hat{R}$

The Gelman-Rubin statistic $\hat{\mathbf{R}}$ measures convergence of MCMC chains by comparing within-chain to between-chain variance, where values below 1.01 indicate well mixed chains [18]. Intuitively this means that the chains have "forgotten" their initial values and therefore both variances are close to each other. To mitigate incorrect diagnoses of convergence failures for chains with heavy tails or varying variance across chains, we use an improved version using rank-normalization, folding and localization [3].

S1.2.6 Effective Sample Size

To measure the quality and uncertainty of the resulting posterior we report the effective sample size (ESS). The ESS quantifies, how much effectively independent information the chains contain and is defined in terms of a pooled autocorrelation estimate combining within-chain and between-chain information [3].

S1.2.7 Simulation-based calibration (SBC)

We assess calibration using simulation-based calibration. For each synthetic dataset, we compute (via the bayesflow package [19]):

- **Rank histograms**, obtained by ranking the true parameter among posterior samples
- **Empirical Cumulative Distribution Function (ECDF)** curves, comparing the empirical distribution of ranks to the uniform distribution.

Well-calibrated inference yields uniform rank histograms and ECDFs lying within the expected sampling envelope.

S1.2.8 Parameter-recovery plots

For conditional normalizing flows, we visualize parameter recovery by plotting posterior means and medians against the true parameters across all datasets using bayesflow's plotting functionalities [19]. These plots provide a direct diagnostic of bias, shrinkage, and systematic distortions in the learned posterior.

S1.3 Models

This section describes the stochastic compartmental models used in our simulation study, together with their prior distributions, feasibility constraints, and model-specific data-generation procedures. We consider three model classes: SIS, SIR, and the two-variant SEIR model (SEIR2V) in both its full and reparameterized forms.

S1.3.1 Prior Bounds and Feasibility Constraints

In addition to the stated priors, we impose simple feasibility constraints to ensure that simulated trajectories remain within a plausible epidemiological range. These constraints are based on the basic reproduction number $r_0 = \beta\gamma^{-1}$ which becomes unrealistically small or large for many naïve combinations of β and γ^{-1}. To avoid such pathological regimes, we restrict the admissible values of β for a given γ^{-1} by repeatedly resampling β until the resulting r_0 lies within a reasonable range.

We additionally constrain the infectious period γ^{-1} to be greater than one day. This avoids numerical instability in the simulation and is consistent with epidemiological knowledge, as infectious periods shorter than one day are implausible for the diseases considered here.

S1.3.2 General Information on Data Generation

This section summarizes the components of the data-generation process that are shared across all models in our simulation study. These include the observational model, the noise assumptions, and the construction of dense and sparse observation schedules. Model-specific data-generation settings—such as parameter regimes, compartmental transitions, and any additional constraints—are provided in the corresponding model subsections below.

Across all models (SIS, SIR, and SEIR2V in both its full and reparameterized forms), we generated multiple synthetic datasets using dense observation schedules as the baseline, with sparse datasets derived where appropriate to mimic realistic epidemiological reporting. All datasets were generated under the requirement that simulated trajectories retain non-degenerate variance. Observations follow the measurement model described in Section 2.4 and are perturbed by binomial noise. For inference, we approximate this binomial noise using a Gaussian model; the rationale and computation of this approximation are detailed in the following subsection.

S1.3.3 Computation of standard deviation for the noise model

When conducting Bernoulli trials, such as testing individuals in a population, the sum of positive test is adequately modeled by a binomial distribution, provided the trials are independent and the success probability remains constant. If the number n of such Bernoulli trials conducted is large or the success probability p is neither exceedingly low nor high, the resulting binomial distribution $B(n, p)$ is well approximated by a normal distribution $\mathcal{N}(np, np(1-p))$, thanks to the de Moivre-Laplace Theorem, a special case of the central limit Theorem [20]. This approximation allows us to compute the standard deviation of the measurement noise directly from the count data and the known population size, and to incorporate it into the noise model. For our setups, the success probabilities are described in Section 2.4 of the main manuscript.

However, this approximation may become inaccurate for small sample sizes or highly skewed probabilities, where alternative methods or exact distributions might be preferable. When generating our synthetic data, we chose $n = 500$, which is sufficiently large to justify the Gaussian approximation.

S1.3.4 SIS

We consider a stochastic SIS model.

Table S1.5: **SIS model description** Compartments and their respective description for the SIS model.

Compartment	Description
S	Susceptible; individuals may become infected upon contact with *Infectious* individuals.
I	Infectious; individuals infected with the disease.

Table S1.6: **Parameters and their description for SIS model.**

Parameter	Description
β	Transmission risk for an individual susceptible to the disease.
γ^{-1}	Average infectious time.

Infection and recovery dynamics governed by the following SDE system.

S1.3.4.1 Model Equations

Set of stochastic differential equations describing the time evolution of individuals in the compartments of the SIS model.

$$dS(t) = -\beta \frac{S(t)I(t)}{N(t)} dt + \gamma I(t) dt - \sqrt{\beta \frac{S(t)I(t)}{N(t)}} dB_1(t) + \sqrt{\gamma I(t)} dB_2(t)$$

$$dI(t) = \beta \frac{S(t)I(t)}{N(t)} dt - \gamma I(t) dt + \sqrt{\beta \frac{S(t)I(t)}{N(t)}} dB_1(t) - \sqrt{\gamma I(t)} dB_2(t)$$

where B_j, $j \in \{1, 2\}$ are independent Brownian Motions. Parameters with respective prior distributions and feasibility bounds are given in Table S1.7.

S1.3.4.2 Prior

The SIS model parameters are constrained to epidemiologically plausible ranges, with priors summarized in Table S1.7.

Table S1.7: **List of model parameters, bounds and priors for the SIS model** Bounds reflect feasibility constraints ensuring epidemiologically plausible values; see Supplement subsection S1.3.1 for details

Parameter	Lower bound	Upper bound	Prior
β	$1.1 \cdot \gamma$	$2.5 \cdot \gamma$	$\text{Uniform}(0.2, 1)$
γ^{-1}	1	20	$\text{Uniform}(1, 20)$

S1.3.4.3 Data generation

For the SIS model we drew ten parameter sets from the prior (subject to the non-degenerate variance constraint), listed in Table S1.8. From each set we generated one densely sampled dataset, with observations every 5 days from day 5 to day 50. Observations consist of a single observable, the infection count. The ten datasets are labeled *sis-1* to *sis-10*.

S1.3.4.4 Parameter Sets

Table S1.8: **Ground-truth parameter sets corresponding to the datasets for the SIS model.**

Set	β	γ^{-1}
sis-1	0.5213	3.43
sis-2	0.8004	2.49
sis-3	0.6469	2.25
sis-4	0.5453	2.17
sis-5	0.9875	1.25
sis-6	0.3839	4.10
sis-7	0.3332	4.64
sis-8	0.4416	3.83
sis-9	0.3925	4.35
sis-10	0.7037	2.24

Table S1.9: **SIR model description** Compartments and their respective description for the SIR model.

Compartment	Description
S	Susceptible; individuals may become infected upon contact with *Infectious* individuals.
I	Infectious; individuals infected with the disease.
R	Recovered; individuals who have recovered from an infection.

S1.3.5 SIR

We consider a stochastic SIR model.

Table S1.10: **Parameters and their description for SIR model.**

Parameter	Description
β	Transmission risk for an individual susceptible to the disease.
γ^{-1}	Average infectious time.

Infection and recovery dynamics governed by the following SDE system.

S1.3.5.1 Model Equations

Set of stochastic differential equations describing the time evolution of individuals in the compartments of the SIR model.

$$
\begin{aligned}
dS(t) &= -\beta \frac{S(t)I(t)}{N(t)} dt - \sqrt{\beta \frac{S(t)I(t)}{N(t)}} dB_1(t) \\
dI(t) &= \beta \frac{S(t)I(t)}{N(t)} dt - \gamma I(t) dt + \sqrt{\beta \frac{S(t)I(t)}{N(t)}} dB_1(t) - \sqrt{\gamma I(t)} dB_2(t) \\
dR(t) &= \gamma I(t) dt + \sqrt{\gamma I(t)} dB_2(t)
\end{aligned}
$$

where B_j, $j \in \{1, 2\}$ are independent Brownian Motions. Parameters with respective prior distributions and bounds are given in Table S1.11.

S1.3.5.2 Prior

The SIR model parameters are constrained to epidemiologically plausible ranges, with priors summarized in Table S1.11.

Table S1.11: **List of model parameters, bounds and priors for the SIR model** Bounds reflect feasibility constraints ensuring epidemiologically plausible values; see Supplement subsection S1.3.1 for details

Parameter	Lower bound	Upper bound	Prior
β	$0.95 \cdot \gamma$	$5 \cdot \gamma$	$\text{Uniform}(0, 1)$
γ^{-1}	1	30	$\text{Uniform}(1, 30)$

S1.3.5.3 Data generation

For the SIR model we generated twelve parameter sets using two regimes:

1. Two hand-crafted parameter sets designed to mimic epidemiological settings.
2. Ten parameter sets drawn from the prior (again under the non-degenerate variance constraint), providing an unbiased foundation for our comparisons.

The parameter sets are listed in Table S1.12. From each set, we generated one densely sampled dataset, with observation points every 10 days from 10 to 100. Observations consist of two distinct observational functions: infection count and seroprevalence. This results in twelve datasets, labeled *sir-1* to *sir-12*, with *sir-1* and *sir-2* corresponding to the hand-crafted parameter sets.

S1.3.5.4 Parameter Sets

Table S1.12: **Ground-truth parameter sets corresponding to the datasets for the SIR model.**

**Set**	β	γ^{-1}
sir-1	0.1000	20.00
sir-2	0.2200	5.00
sir-3	0.4306	7.31
sir-4	0.3346	11.05
sir-5	0.3329	10.05
sir-6	0.4962	8.29
sir-7	0.4462	4.40
sir-8	0.5871	7.13
sir-9	0.3920	9.94
sir-10	0.4380	8.51
sir-11	0.2193	14.75
sir-12	0.9121	1.24

Table S1.13: **Two-variant SEIR model description** Compartments and their respective description for the two-variant SEIR model.

**Compartment**	**Description**
S	Susceptible; individuals may become infected upon contact with *Infectious* individuals.
E_{wt}	Exposed; individuals with no prior infection and infected with the wild-type but not yet infectious.
E_{var}	Exposed; individuals with no prior infection and infected with the variant but not yet infectious.
$E_{wt \cdot var}$	Exposed; individuals with a prior infection from the wild-type and infected with the variant but not yet infectious.
I_{wt}	Infectious; individuals with no prior infection, infected with the wild-type and currently infectious.
I_{var}	Infectious; individuals with no prior infection, infected with the variant and currently infectious.
$I_{wt \cdot var}$	Infectious; individuals infected with the variant, infectious and who have previously been infected by the wild-type.
R_{wt}	Recovered; individuals who have recovered from a wild-type infection, not currently infected.
R_{var}	Recovered; individuals who have recovered from a variant infection, not currently infected.
$R_{wt \cdot var}$	Recovered; individuals recovered from a reinfection with the variant, after having have recovered from a prior wild-type infection.

308 ### S1.3.6 SEIR2V

309 We consider a stochastic two variant SEIR model with the following compartments and parameters

Table S1.14: **Parameters and their description for the two-variant SEIR model.**

**Parameter**	**Description**
β	Transmission risk for an individual susceptible to one of the two-variants.
κ^{-1}	Average latency period length for the *wild-type* and the *variant*.
γ^{-1}	Average infectious time for the *wild-type*.
s	Scaling factor, measuring the discrepancy between (measured and reported) newly infected and actually infected.
I_0	Number of individuals infected with the *wild-type* at start of the simulation.
t_{var}	Time at which n_{var} individuals are introduced into the compartment I_{var}.

310 Infection and recovery dynamics are governed by the following SDE system.

S1.3.6.1 Model Equations

Set of stochastic differential equations describing the time evolution of individuals in the compartments of the two variant SEIR model.

$$
\begin{aligned}
dS(t) &= -\beta\frac{S(t)\mathrm{I}_{\mathrm{wt}}(t)}{N(t)}dt - \beta\frac{S(t)(I_{var}(t)+I_{both}(t))}{N(t)}dt \\
&\quad - \sqrt{\beta\frac{S(t)I_{wt}(t)}{N(t)}}dB_1(t) - \sqrt{\beta\frac{S(t)(I_{var}(t)+I_{both}(t))}{N(t)}}dB_7(t) \\
dE_{wt}(t) &= \beta\frac{S(t)I_{wt}(t)}{N(t)}dt - \kappa E_{wt}(t)dt + \sqrt{\beta\frac{S(t)I_{wt}(t)}{N(t)}}dB_1(t) - \sqrt{\kappa E_{wt}(t)}dB_2(t) \\
dI_{wt}(t) &= \kappa E_{wt}(t)dt - \gamma I_{wt}(t)dt + \sqrt{\kappa E_{wt}(t)}dB_2(t) - \sqrt{\gamma I_{wt}(t)}dB_3(t) \\
dR_{wt}(t) &= \gamma I_{wt}(t)dt - \beta\frac{R_{wt}(t)(I_{var}(t)+I_{both}(t))}{N(t)}dt \\
&\quad + \sqrt{\gamma I_{wt}(t)}dB_3(t) - \sqrt{\beta\frac{R_{wt}(t)(I_{var}(t)+I_{both}(t))}{N(t)}}dB_4(t) \\
dE_{var}(t) &= \beta\frac{S(t)(I_{var}(t)+I_{both}(t))}{N(t)}dt - \kappa E_{var}(t)dt \\
&\quad + \sqrt{\beta\frac{S(t)(I_{var}(t)+I_{both}(t))}{N(t)}}dB_7(t) - \sqrt{\kappa E_{var}(t)}dB_8(t) \\
dI_{var}(t) &= \kappa E_{var}(t)dt - \frac{\gamma}{c}I_{var}(t)dt + \sqrt{\kappa E_{var}(t)}dB_8(t) - \sqrt{\frac{\gamma}{c}I_{var}(t)}dB_9(t) \\
dR_{var}(t) &= \frac{\gamma}{c}I_{var}(t)dt + \sqrt{\frac{\gamma}{c}I_{var}(t)}dB_9(t) \\
dE_{both}(t) &= \beta\frac{R_{wt}(t)(I_{var}(t)+I_{both}(t))}{N(t)}dt - \kappa E_{both}(t)dt \\
&\quad + \sqrt{\beta\frac{R_{wt}(t)(I_{var}(t)+I_{both}(t))}{N(t)}}dB_4(t) - \sqrt{\kappa E_{both}(t)}dB_5(t) \\
dI_{both}(t) &= \kappa E_{both}(t)dt - \frac{\gamma}{c}I_{both}(t)dt + \sqrt{\kappa E_{both}(t)}dB_5(t) - \sqrt{\frac{\gamma}{c}I_{both}(t)}dB_6(t) \\
dR_{both}(t) &= \frac{\gamma}{c}I_{both}(t)dt + \sqrt{\frac{\gamma}{c}I_{both}(t)}dB_6(t),
\end{aligned}
$$

where B_j, $j \in \{1, ..., 9\}$ are independent Brownian Motions. The variant infectious-period factor $c = 1.35$ rescales the recovery rate of variant-infected individuals, so that the variant remains infectious c times longer than the wild-type ($\gamma_{\mathrm{var}} = \gamma/c$). Following [15], it is held fixed and not inferred. Parameters with respective prior distributions and bounds are given in Table S1.15.

S1.3.6.2 Practical non-identifiability of the two-variant SEIR model

In our comparison we observed correlated parameter estimates leading to posterior misalignment between the methods. In several parameter combinations, the posterior mass was concentrated along a lower-dimensional manifold in parameter space (Figure S1.2), indicating practical non-identifiability.

This manifold can be approximated in terms of the following reparameterization into epidemiologically interpretable quantities:

$$
\begin{aligned}
r_0 &= \beta\,\gamma^{-1}, \\
e_0 &= \kappa^{-1} + \gamma^{-1}, \\
s_0 &= s\,\gamma^{-1}, \\
t_{\text{var}} &= t_{\text{var}}, \\
I_0 &= I_0.
\end{aligned} \tag{5}
$$

where r_0 is the basic reproduction number, e_0 the mean latent-plus-infectious period, i.e., mean time an individual spends from catching the infection until it stops being infectious, and s_0 the effective detection factor integrated over the average infectious period, so how many cases are likely to be detected per infectious person, averaged over their infectious period.

A representative example is shown in Figure S1.3. The substantial overlap between the trajectories shows that combinations of widely different parameters can produce virtually identical epidemic dynamics.

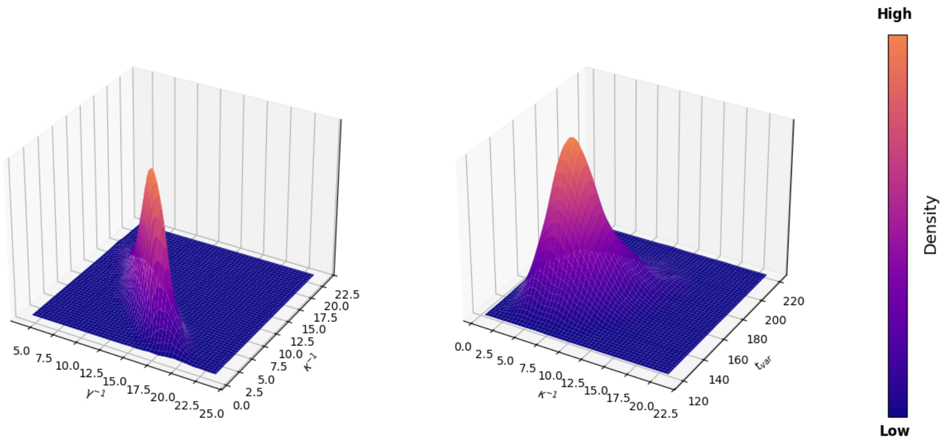

Figure S1.2: **Posterior Geometry** 3D Plot of selected pairwise density plots. The *mountain pass* geometry on the left makes inference of parameters more challenging compared to the *mountain peak* geometry on the right.

S1.3.6.3 Prior

The SEIR2v model parameters are constrained to epidemiologically plausible ranges, with priors summarized in Table S1.15 and Table S1.16. The prior for the full two-variant SEIR model is a slightly adjusted version of the prior used in [15].

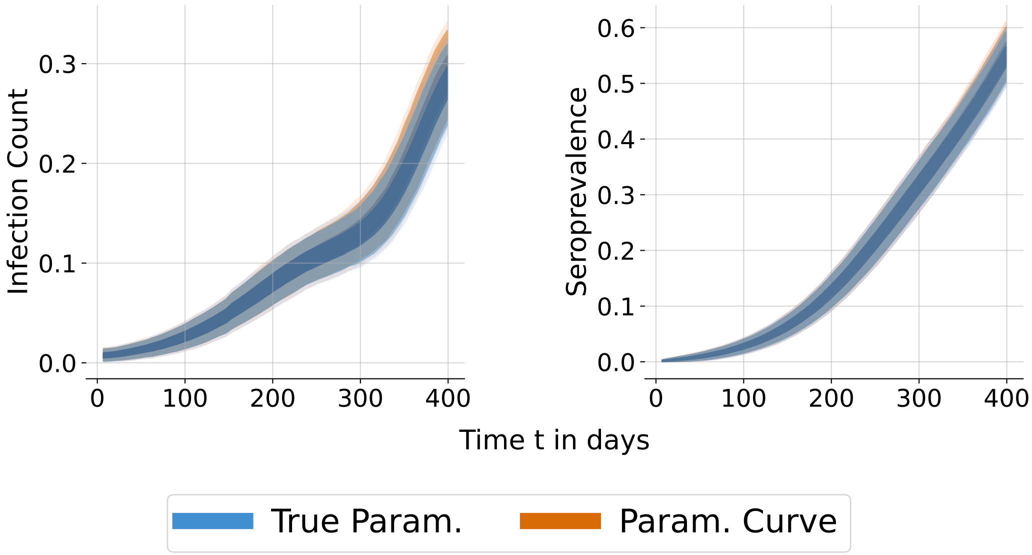

Figure S1.3: **Illustration of a practical identifiability manifold in the two-variant SEIR model.** Orange: simulations from the fixed parameter set (17, 5, 0.08, 3, 150, 500). Blue: simulations from parameter sets with γ^{-1} ranging from 11 to 20 and other parameters adjusted according to the reparametrization. The near-complete overlap of trajectories demonstrates that distinct parameter combinations can produce almost indistinguishable epidemic curves.

Table S1.15: **List of model parameters, bounds and priors for the two variant SEIR model.** Prior distributions were taken from the published model. Bounds reflect feasibility constraints ensuring epidemiologically plausible values; see Supplementary Information S1 for details.

Parameter	Lower bound	Upper bound	Prior
γ^{-1}	0	∞	Normal$(15.7, 6.7)$
κ	0	∞	Lognormal$(1.63, 0.5)$
β	$0.95 \cdot \gamma$	$4 \cdot \gamma$	Uniform$(0, 1)$
s	0.1	10	Uniform$(0.1, 10)$
t_{var}	120	360	Uniform$(120, 360)$
I_0	10	1000	Uniform$(10, 1000)$

Table S1.16: **List of model parameters, bounds and priors for the reparametrized two variant SEIR model** Bounds reflect feasibility constraints ensuring epidemiologically plausible values; see Supplement subsection S1.3.1 for details.

Parameter	Lower bound	Upper bound	Prior
r_0	0.95	4.0	Uniform$(0.95, 4.0)$
e_0	6	30	Uniform$(6, 30)$
s_0	1	100	Uniform$(1, 100)$
t_{var}	120	360	Uniform$(120, 360)$
I_0	10	1000	Uniform$(10, 1000)$

S1.3.6.4 Data generation

SEIR2V (full) For the full SEIR2V model we generated twelve parameter sets using three regimes:

1. One hand-crafted parameter set based on the results for the SEIR2V model in [15].
2. One hand-crafted parameter set designed to mimic epidemiological settings.
3. Ten parameter sets drawn from the prior (again under the non-degenerate variance constraint), providing an unbiased foundation for our comparisons.

The resulting datasets and their associated ground-truth parameter values are listed in Table S1.17. For each of the two hand-crafted parameter sets, we generated two densely sampled datasets (four datasets total), labeled *d-1-1*, *d-1-2*, *d-2-1*, and *d-2-2*. A dense dataset consists of observations every 7 days, from day 7 through day 399. Each of the ten prior-drawn parameter sets produced one densely sampled dataset, corresponding to *d-3* through *d-12*. This yields fourteen dense datasets in total.

To mimic realistic data sparsity, we constructed sparse datasets from the dense datasets as follows:

- From dense datasets *d-1-1* and *d-1-2*, which correspond to the publication-based parameter set, we generated three sparse datasets each using the following procedure:

1. draw three integers between 0 and 8, specifying the number of infection-count timepoints, seroprevalence timepoints, and jointly observed timepoints;
2. randomly subsample the dense dataset accordingly.

- From dense datasets *d-3* to *d-12*, which correspond to the ten parameter sets drawn from the prior, we generated one sparse dataset per dense dataset by randomly subsampling 8 infection-count observation times and 8 seroprevalence observation times.

This yields sixteen sparse datasets in total. The six sparse datasets derived from *d-1-1* and *d-1-2* are labeled *s-1-1-1* to *s-1-1-3* and *s-1-2-1* to *s-1-2-3*, respectively. The ten sparse datasets derived from *d-3* to *d-12* are labeled *s-3* to *s-12*. Table S1.18 provides the corresponding ground-truth parameter values for all sparse datasets.

SEIR2V (reparametrized) For the reparameterized SEIR2V model we generated eleven parameter sets using two regimes:

1. One hand-crafted parameter set based on the results for the SEIR2V model in [15]. Under the transformation in (5), this parameter set corresponds directly to the publication-based parameter set used for the full SEIR2V model.
2. Ten parameter sets drawn from the prior (again under the non-degenerate variance constraint), providing an unbiased foundation for our comparisons.

For the publication-based parameter set, we reused the same simulated trajectories as in the full model, namely the dense datasets *d-1-1* and *d-1-2*. For each of the ten prior-drawn parameter sets, we generated one densely sampled dataset. Observations consist of two distinct observational functions (infection count and seroprevalence), recorded every 7 days from day 7 to day 399.

In total, this yields twelve densely sampled datasets: the two reused datasets *d-1-1* and *d-1-2*, followed by ten newly generated datasets labeled *r-1* to *r-10*. Table S1.19 provides the ground-truth parameter values for the reparameterized model.

Additionally, to enable direct comparison between the reparameterized and full SEIR2V models, we transformed each reparameterized parameter set into its corresponding full-model parameterization using (5). Table S1.20 lists the resulting full-model parameter values.

Publication Data For an application of the methods to real-world data, we use data from a longitudinal cohort study in Ethiopia[15]. As reference parameters we use the parameter set reported therein. This dataset is labeled as *eth.*.

S1.3.6.5 Parameter Sets

Table S1.17: **Ground-truth parameter sets corresponding to the dense datasets for the full SEIR2V model.**

**Set**	γ^{-1}	κ^{-1}	β	s	t_{var}	I_0
d-1-1	17.00	5.00	0.0800	3.00	150	500.0
d-1-2	17.00	5.00	0.0800	3.00	150	500.0
d-2-1	11.70	8.40	0.2300	1.60	222	560.0
d-2-2	11.70	8.40	0.2300	1.60	222	560.0
d-3	15.47	3.97	0.0655	8.40	226	477.3
d-4	11.57	6.11	0.1605	8.34	318	169.6
d-5	24.05	6.63	0.0423	6.45	122	789.9
d-6	22.21	4.37	0.0807	1.91	274	831.2
d-7	23.34	3.52	0.0631	6.43	329	79.0
d-8	16.66	3.35	0.0699	4.95	289	604.8
d-9	17.98	6.59	0.1430	3.55	317	859.7
d-10	16.77	5.57	0.1786	1.18	172	31.8
d-11	18.63	9.93	0.1807	1.07	180	157.8
d-12	8.51	3.80	0.1731	4.66	299	327.2

Table S1.18: **Ground-truth parameter sets corresponding to the sparse datasets for the full SEIR2V model.**

**Set**	γ^{-1}	κ^{-1}	β	s	t_{var}	I_0
s-1-1-1	17.00	5.00	0.0800	3.00	150	500.0
s-1-1-2	17.00	5.00	0.0800	3.00	150	500.0
s-1-1-3	17.00	5.00	0.0800	3.00	150	500.0
s-1-2-1	17.00	5.00	0.0800	3.00	150	500.0
s-1-2-2	17.00	5.00	0.0800	3.00	150	500.0
s-1-2-3	17.00	5.00	0.0800	3.00	150	500.0
s-3	15.47	3.97	0.0655	8.40	226	477.3
s-4	11.57	6.11	0.1605	8.34	318	169.6
s-5	24.05	6.63	0.0423	6.45	122	789.9
s-6	22.21	4.37	0.0807	1.91	274	831.2
s-7	23.34	3.52	0.0631	6.43	329	79.0
s-8	16.66	3.35	0.0699	4.95	289	604.8
s-9	17.98	6.59	0.1430	3.55	317	859.7
s-10	16.77	5.57	0.1786	1.18	172	31.8
s-11	18.63	9.93	0.1807	1.07	180	157.8
s-12	8.51	3.80	0.1731	4.66	299	327.2

Table S1.19: **Ground-truth parameter sets corresponding to the (dense) datasets for the reparametrized SEIR2V model.**

**Set**	r_0	e_0	s_0	t_{var}	I_0
d-1-1	1.360	22.00	51.00	150	500.0
d-1-2	1.360	22.00	51.00	150	500.0
r-1	3.239	13.63	20.80	138	563.0
r-2	1.213	6.83	34.36	353	410.5
r-3	2.625	17.48	44.50	252	551.7
r-4	3.566	21.43	45.99	338	114.4
r-5	1.235	9.61	51.29	171	227.2
r-6	1.217	10.79	5.37	337	497.2
r-7	1.769	24.29	99.31	142	748.2
r-8	1.062	13.71	80.25	167	940.2
r-9	2.621	13.69	8.07	265	135.1
r-10	1.166	27.22	35.70	147	428.9

Table S1.20: **Ground-truth parameter sets corresponding to the (dense) datasets for the reparametrized SEIR2V model, transformed to the parameter space of the full SEIR2V model.**

**Set**	γ^{-1}	κ^{-1}	β	s	t_{var}	I_0
d-1-1	17.00	5.00	0.0800	3.00	150	500.0
d-1-2	17.00	5.00	0.0800	3.00	150	500.0
r-1	8.63	5.00	0.3754	2.41	138	563.0
r-2	1.83	5.00	0.6617	18.74	353	410.5
r-3	12.48	5.00	0.2104	3.57	252	551.7
r-4	16.43	5.00	0.2171	2.80	338	114.4
r-5	4.61	5.00	0.2678	11.12	171	227.2
r-6	5.79	5.00	0.2102	0.93	337	497.2
r-7	19.29	5.00	0.0917	5.15	142	748.2
r-8	8.71	5.00	0.1219	9.21	167	940.2
r-9	8.69	5.00	0.3018	0.93	265	135.1
r-10	22.22	5.00	0.0525	1.61	147	428.9

Table S1.21: **Parameter sets for the full SEIR2V model corresponding to the real-world dataset from a study in Ethiopia[15].**

**Set**	γ^{-1}	κ^{-1}	β	s	t_{var}	I_0
eth.	16.70	5.0	0.08	2.3	184.5	635.0

397 ### S1.3.7 Computation Times

Table S1.22: **Comparison of computation times:** Accumulated CPU-time in minutes for both methods applied to a dense dataset. For the PF we ran 4 chains on 4 cores in parallel, the CNF was parallelized over 20 cores.

**Datasets**	**PF**	**CNF**
sir-1	1163.33	106.72
d-1-1	2095.98	252.96
s-1-1-1	1798.1	132.29

References

[1] Doucet A, Pitt MK, Deligiannidis G, Kohn R. Efficient implementation of Markov chain Monte Carlo when using an unbiased likelihood estimator. Biometrika. 2015;102(2):295-313. doi:10.1093/biomet/asu075.

[2] Chopin N, Papaspiliopoulos O. An Introduction to sequential Monte Carlo. Springer Series in Statistics. Springer International Publishing; 2020. doi:10.1007/978-3-030-47845-2.

[3] Vehtari A, Gelman A, Simpson D, Carpenter B, Bürkner PC. Rank-normalization, folding, and localization: An improved $\hat{R}$ for assessing convergence of MCMC (with discussion). Bayesian analysis. 2021;16(2). doi:10.1214/20-BA1221.

[4] Doucet A, Johansen AM. A Tutorial on Particle Filtering and Smoothing: Fifteen years later. Oxford Handbook of Nonlinear filtering. 2008. Available from: https://www.academia.edu/129324213/A_tutorial_on_particle_filtering_and_smoothing_fifteen_years_later.

[5] Doucet A, de Freitas N, Gordon NJ. Sequential Monte Carlo methods in practice. Statistics for Engineering and Information Science. Springer New York; 2001. doi:10.1007/978-1-4757-3437-9.

[6] Douc R, Cappe O. Comparison of resampling schemes for particle filtering. Proceedings of the 4th International Symposium on Image and Signal Processing and Analysis. 2005:64-9. doi:10.1109/ISPA.2005.195385.

[7] Roberts GO. On inference for partially observed nonlinear diffusion models using the Metropolis-Hastings algorithm. Biometrika. 2001;88(3):603-21. doi:10.1093/biomet/88.3.603.

[8] Golightly A, Wilkinson DJ. Bayesian inference for stochastic kinetic models using a diffusion approximation. Biometrics. 2005;61(3):781-8. doi:10.1111/j.1541-0420.2005.00345.x.

[9] Papaspiliopoulos O, Roberts GO, Stramer O. Data Augmentation for Diffusions. Journal of Computational and Graphical Statistics. 2013;22(3):665-88. doi:10.1080/10618600.2013.783484.

[10] Dragatz C. Bayesian inference for diffusion processes. Fakultät für Mathematik, Informatik und Statistik der Ludwig-Maximilians-Universität München; 2010. doi:10.5282/edoc.12136.

[11] Whitaker GA, Golightly A, Boys RJ, Sherlock C. Improved bridge constructs for stochastic differential equations. Statistics and Computing. 2015. doi:10.1007/s11222-016-9660-3.

[12] van der Meulen F, Schauer M. Bayesian estimation of discretely observed multi-dimensional diffusion processes using guided proposals. Electronic Journal of Statistics. 2017. doi:10.1214/17-EJS1290.

[13] Hoffman MD, Gelman A, et al. The No-U-Turn sampler: adaptively setting path lengths in Hamiltonian Monte Carlo. J Mach Learn Res. 2014;15(1):1593-623.

[14] Fjelde TE, Xu K, Widmann D, Tarek M, Pfiffer C, Trapp M, et al. Turing.jl: a general-purpose probabilistic programming language. ACM Transactions on Probabilistic Machine Learning. 2025.

[15] Gudina EK, Ali S, Girma E, Gize A, Tegene B, Hundie GB, et al. Seroepidemiology and model-based prediction of SARS CoV 2 in Ethiopia: longitudinal cohort study among front-line hospital workers and communities. The Lancet Global Health. 2021;9(11):e1517-27. doi:10.1016/S2214-109X(21)00386-7.

[16] Villani C, et al. Optimal transport: old and new. vol. 338. Springer; 2009. doi:https://doi.org/10.1007/978-3-540-71050-9.

[17] Gneiting T, Raftery AE. Strictly proper scoring rules, prediction, and estimation. Journal of the American statistical Association. 2007;102(477):359-78. doi:https://doi.org/10.1198/016214506000001437.

[18] Gelman A, Rubin DB. Inference from iterative simulation using multiple sequences. Statistical science. 1992;7(4):457-72. doi:10.1214/ss/1177011136.

[19] Radev ST, Schmitt M, Schumacher L, Elsemüller L, Pratz V, Schälte Y, et al.. BayesFlow: Amortized Bayesian Workflows With Neural Networks. arXiv; 2023. doi:10.48550/arXiv.2306.16015.

[20] Walker HM, Helen M. De Moivre on the law of normal probability. Smith, David Eugene A Source Book in Mathematics, Dover. 1985:64690-4. Available from: https://www.york.ac.uk/depts/maths/histstat/demoivre.pdf.
