# Supplementary material for: Assessment of simulation-based inference methods for stochastic compartmental models in epidemiological research
Source: PLoS One. 2026 Jul 13;21(7):e0353306. doi: 10.1371/journal.pone.0353306 (PMC13362117; doi:10.1371/journal.pone.0353306)
Supplement: S2 Result — (PDF) [file pone.0353306.s002.pdf]

S2 Supplementary Results SIS-Model

Assessment of Simulation-based Inference Methods for Stochastic Compartmental Models in Epidemiological Research

Vincent Wieland[1,2,◑], Nils Waßmuth[1,2,3,◑], Lorenzo Contento[1], Martin Kühn[1,2,3], and Jan Hasenauer[1,2,*]

[1]Bonn Center for Mathematical Life Sciences, University of Bonn, Bonn, Germany
[2]Life and Medical Science Institute, University of Bonn, Bonn, Germany
[3]Institute of Software Technology, Department for High-Performance Computing, German Aerospace Center (DLR), Cologne, Germany
[◑]These authors contributed equally to the work.
[*]To whom correspondence should be addressed; jan.hasenauer@uni-bonn.de.

June 26, 2026

Contents

S2.A Supplementary Figures 2
S2.B Supplementary Tables 11

S2.A Supplementary Figures

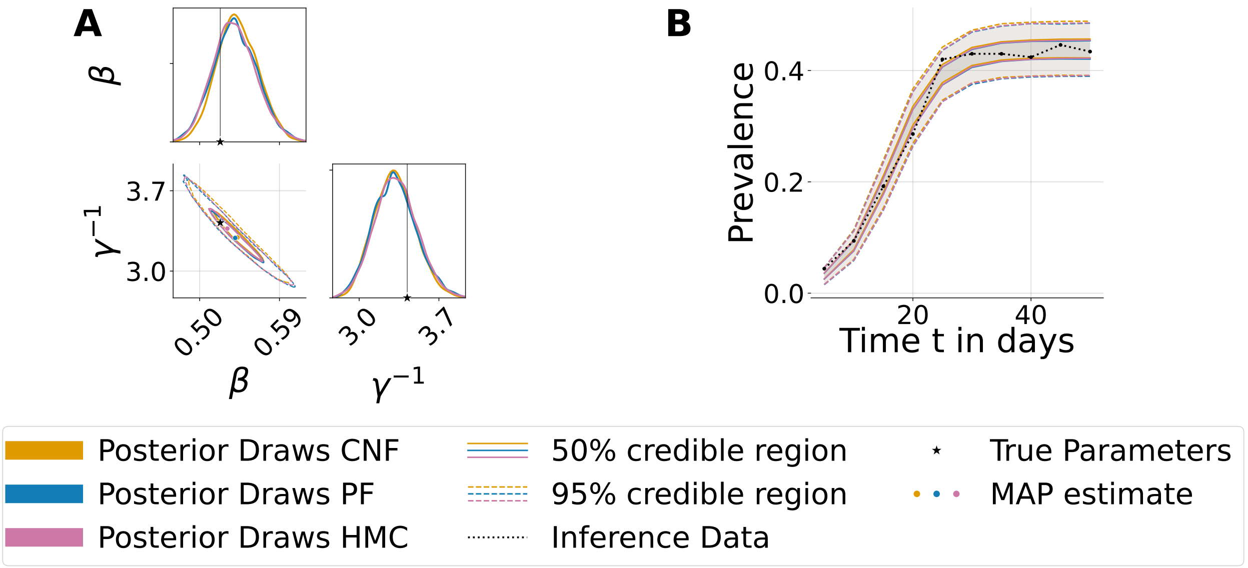

Figure S2.1: **Results of the SIS model for *sis-1*.**
A Posterior approximations from 10,000 samples. Contour gives the 50% (solid) and 95% (dashed) credible regions, coloured by method. Diagonals show the 1D marginals. Black stars mark the true parameters, coloured circles the joint MAP estimates. **B** Posterior predictive fit: bands give the 50% and 95% pointwise predictive intervals from the same samples (line styles as in **A**) with inference data shown as a dotted line.

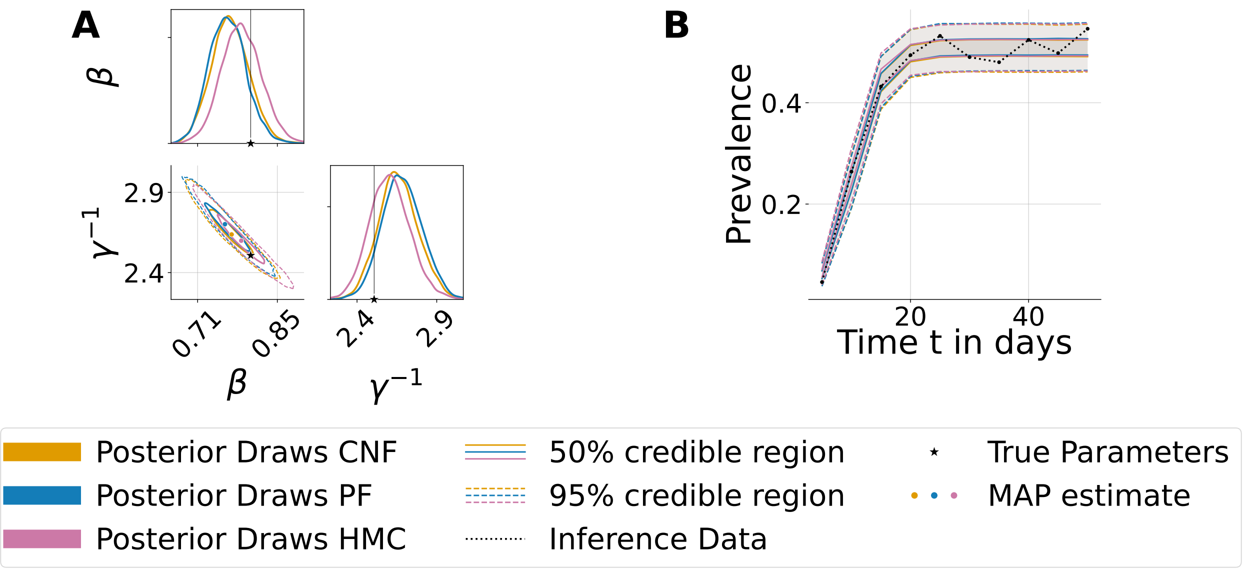

Figure S2.2: **Results of the SIS model for *sis-2*.**
A Posterior approximations from 10,000 samples. Contour gives the 50% (solid) and 95% (dashed) credible regions, coloured by method. Diagonals show the 1D marginals. Black stars mark the true parameters, coloured circles the joint MAP estimates. **B** Posterior predictive fit: bands give the 50% and 95% pointwise predictive intervals from the same samples (line styles as in **A**) with inference data shown as a dotted line.

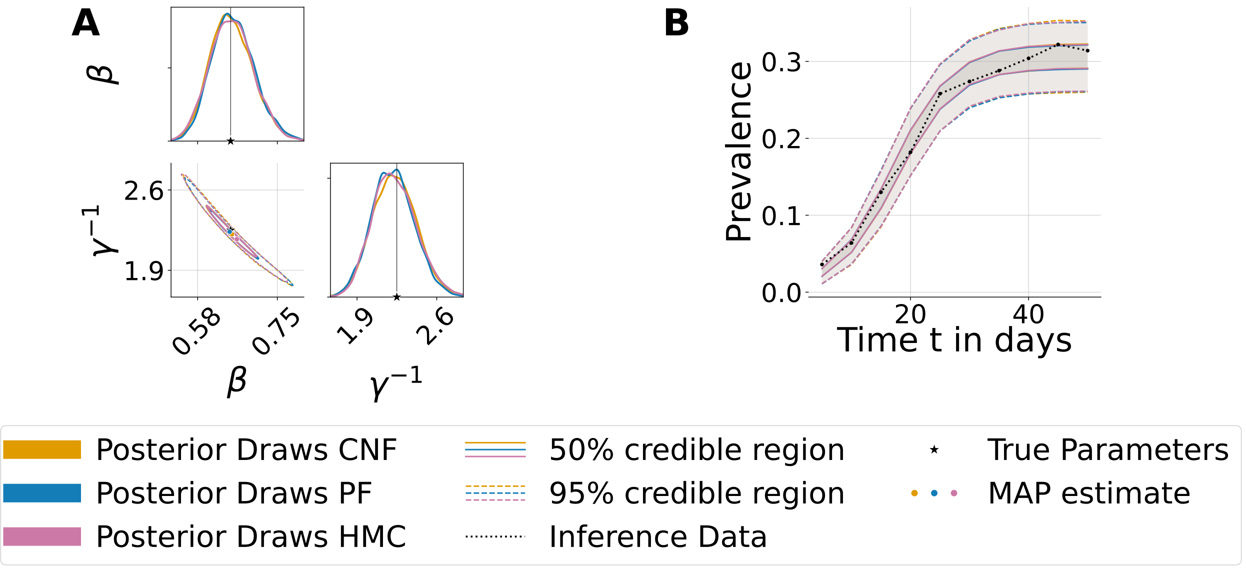

Figure S2.3: **Results of the SIS model for *sis-3*.**
A Posterior approximations from 10,000 samples. Contour gives the 50% (solid) and 95% (dashed) credible regions, coloured by method. Diagonals show the 1D marginals. Black stars mark the true parameters, coloured circles the joint MAP estimates. **B** Posterior predictive fit: bands give the 50% and 95% pointwise predictive intervals from the same samples (line styles as in **A**) with inference data shown as a dotted line.

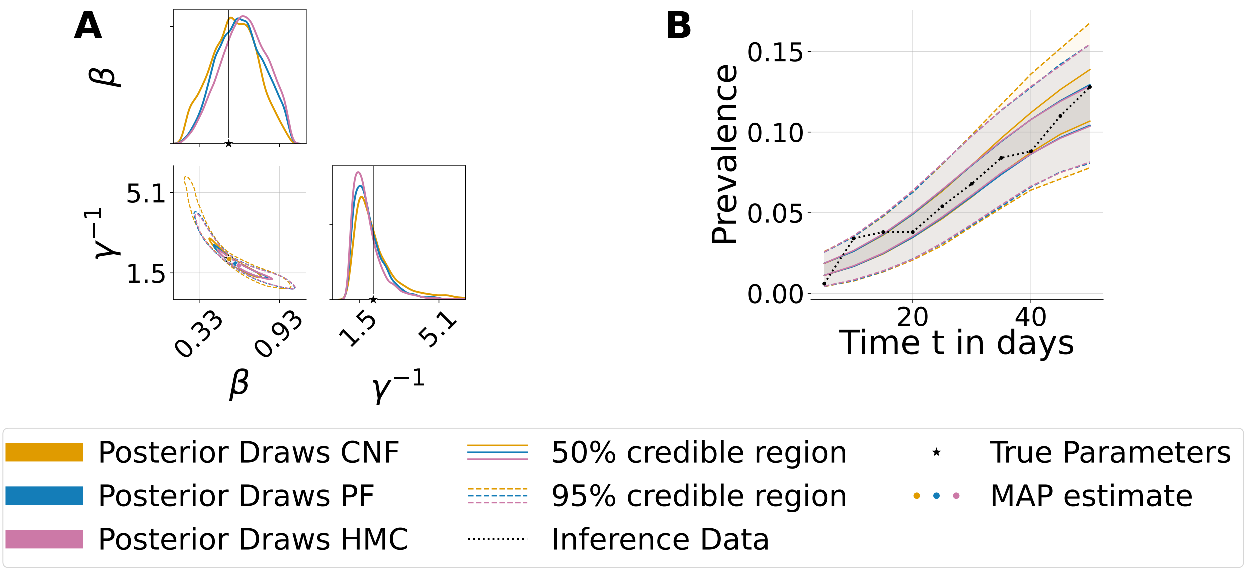

Figure S2.4: **Results of the SIS model for *sis-4*.**
A Posterior approximations from 10,000 samples. Contour gives the 50% (solid) and 95% (dashed) credible regions, coloured by method. Diagonals show the 1D marginals. Black stars mark the true parameters, coloured circles the joint MAP estimates. **B** Posterior predictive fit: bands give the 50% and 95% pointwise predictive intervals from the same samples (line styles as in **A**) with inference data shown as a dotted line.

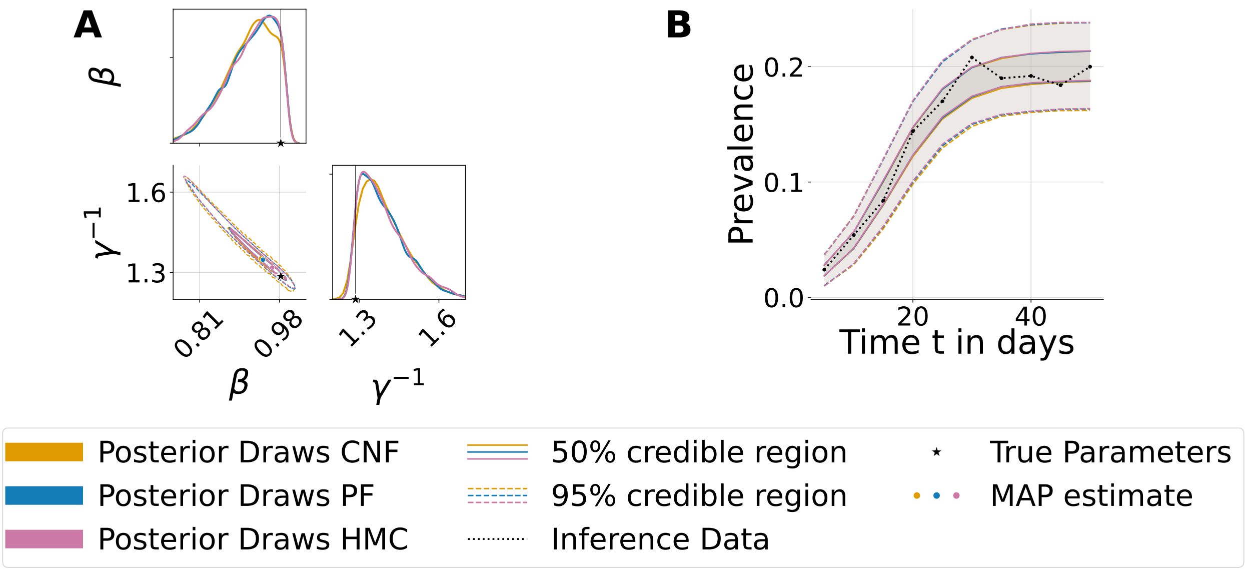

Figure S2.5: **Results of the SIS model for *sis-5*.**
A Posterior approximations from 10,000 samples. Contour gives the 50% (solid) and 95% (dashed) credible regions, coloured by method. Diagonals show the 1D marginals. Black stars mark the true parameters, coloured circles the joint MAP estimates. **B** Posterior predictive fit: bands give the 50% and 95% pointwise predictive intervals from the same samples (line styles as in **A**) with inference data shown as a dotted line.

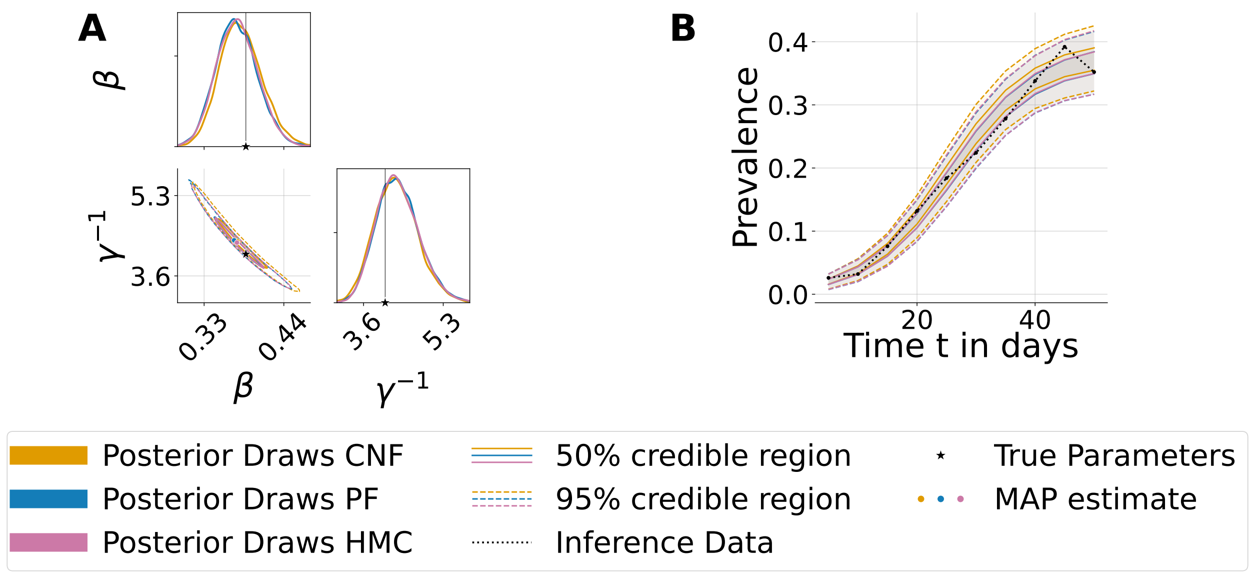

Figure S2.6: **Results of the SIS model for *sis-6*.**
A Posterior approximations from 10,000 samples. Contour gives the 50% (solid) and 95% (dashed) credible regions, coloured by method. Diagonals show the 1D marginals. Black stars mark the true parameters, coloured circles the joint MAP estimates. **B** Posterior predictive fit: bands give the 50% and 95% pointwise predictive intervals from the same samples (line styles as in **A**) with inference data shown as a dotted line.

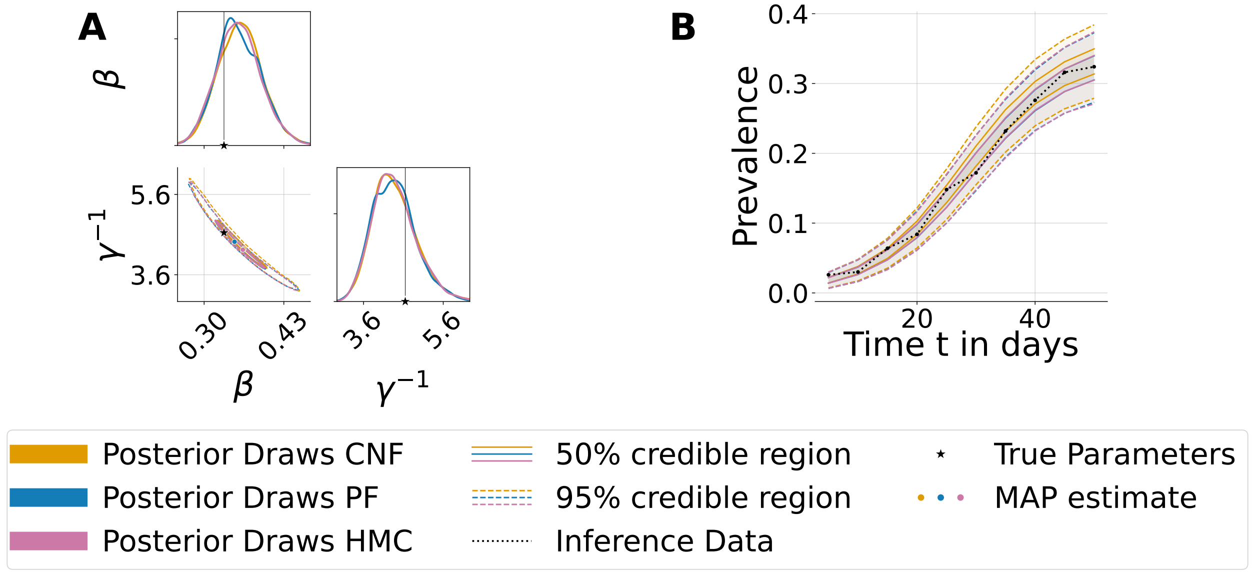

Figure S2.7: **Results of the SIS model for *sis-7*.**
A Posterior approximations from 10,000 samples. Contour gives the 50% (solid) and 95% (dashed) credible regions, coloured by method. Diagonals show the 1D marginals. Black stars mark the true parameters, coloured circles the joint MAP estimates. **B** Posterior predictive fit: bands give the 50% and 95% pointwise predictive intervals from the same samples (line styles as in **A**) with inference data shown as a dotted line.

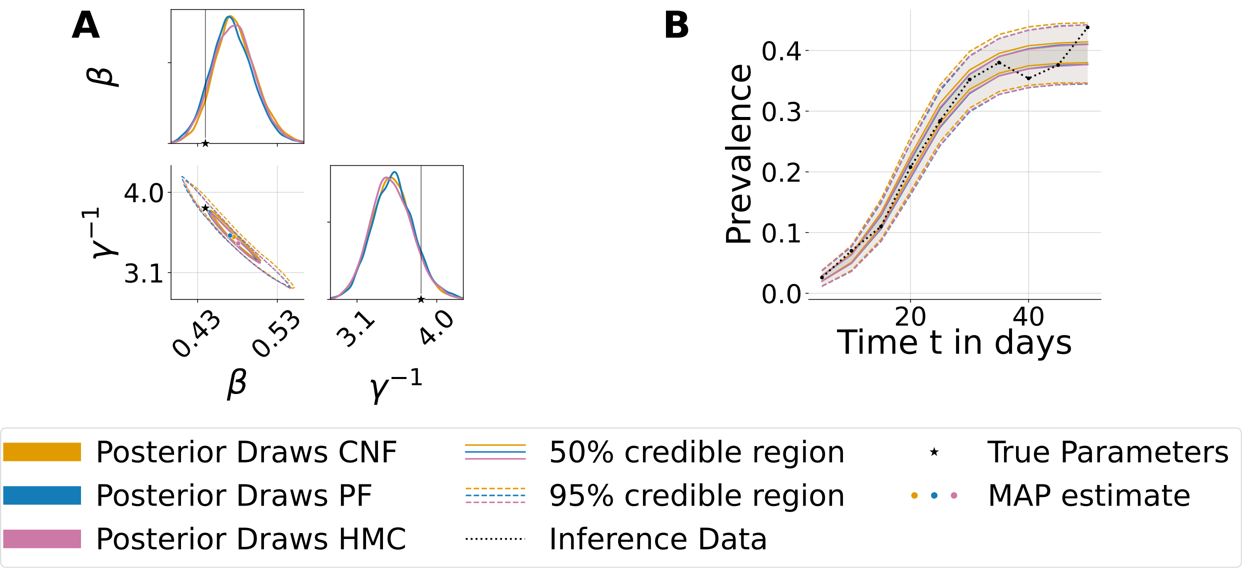

Figure S2.8: **Results of the SIS model for *sis-8*.**
A Posterior approximations from 10,000 samples. Contour gives the 50% (solid) and 95% (dashed) credible regions, coloured by method. Diagonals show the 1D marginals. Black stars mark the true parameters, coloured circles the joint MAP estimates. **B** Posterior predictive fit: bands give the 50% and 95% pointwise predictive intervals from the same samples (line styles as in **A**) with inference data shown as a dotted line.

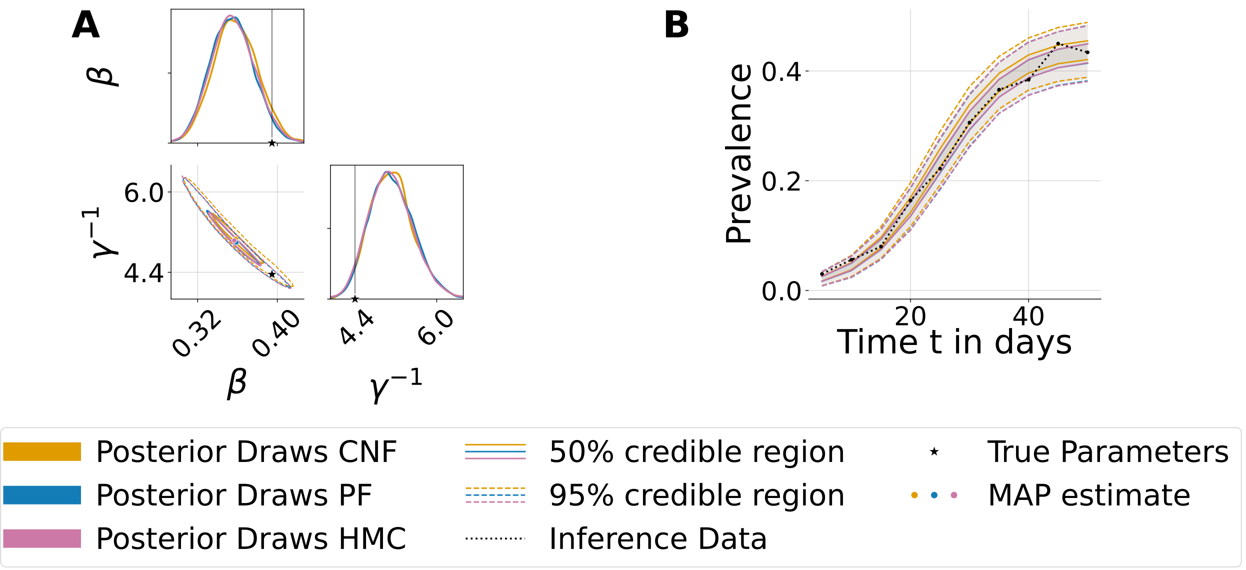

Figure S2.9: **Results of the SIS model for *sis-9*.**
A Posterior approximations from 10,000 samples. Contour gives the 50% (solid) and 95% (dashed) credible regions, coloured by method. Diagonals show the 1D marginals. Black stars mark the true parameters, coloured circles the joint MAP estimates. **B** Posterior predictive fit: bands give the 50% and 95% pointwise predictive intervals from the same samples (line styles as in **A**) with inference data shown as a dotted line.

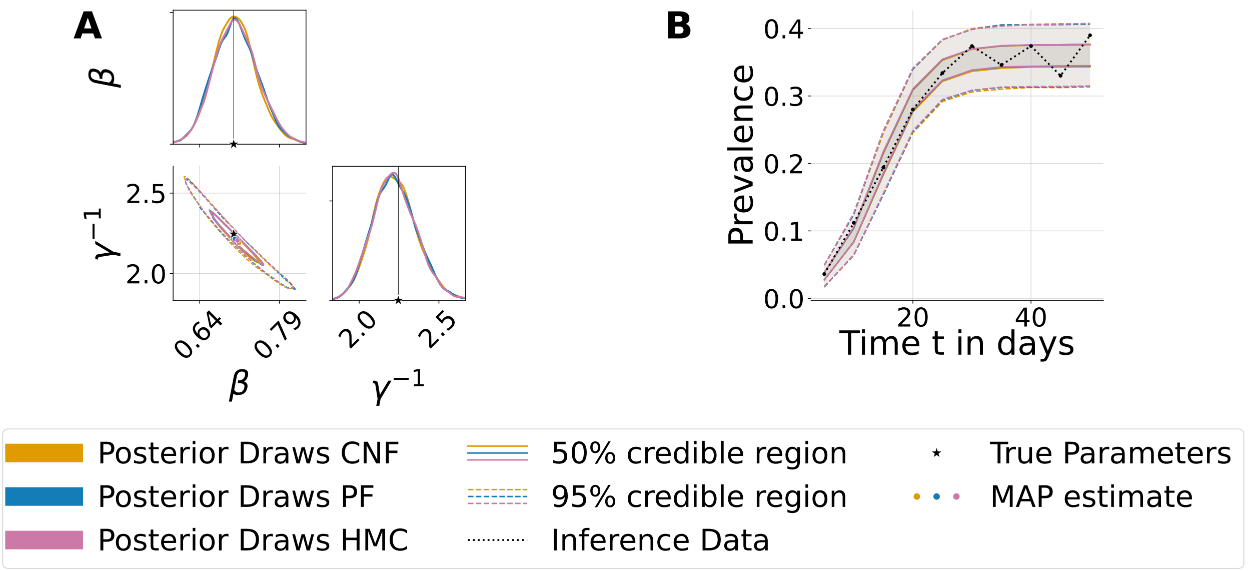

Figure S2.10: **Results of the SIS model for *sis-10*.**
A Posterior approximations from 10,000 samples. Contour gives the 50% (solid) and 95% (dashed) credible regions, coloured by method. Diagonals show the 1D marginals. Black stars mark the true parameters, coloured circles the joint MAP estimates. **B** Posterior predictive fit: bands give the 50% and 95% pointwise predictive intervals from the same samples (line styles as in **A**) with inference data shown as a dotted line.

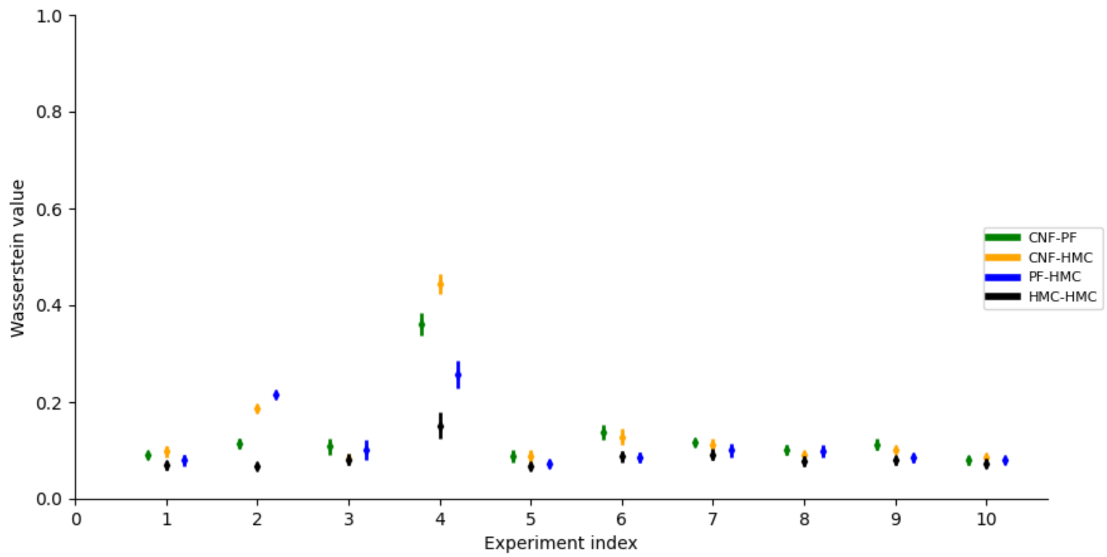

Figure S2.11: **Wasserstein distances for the SIS model**
Pairwise 1-Wasserstein distances between posterior samples across experiment indices for all method combinations. Error bars show Monte-Carlo variability.

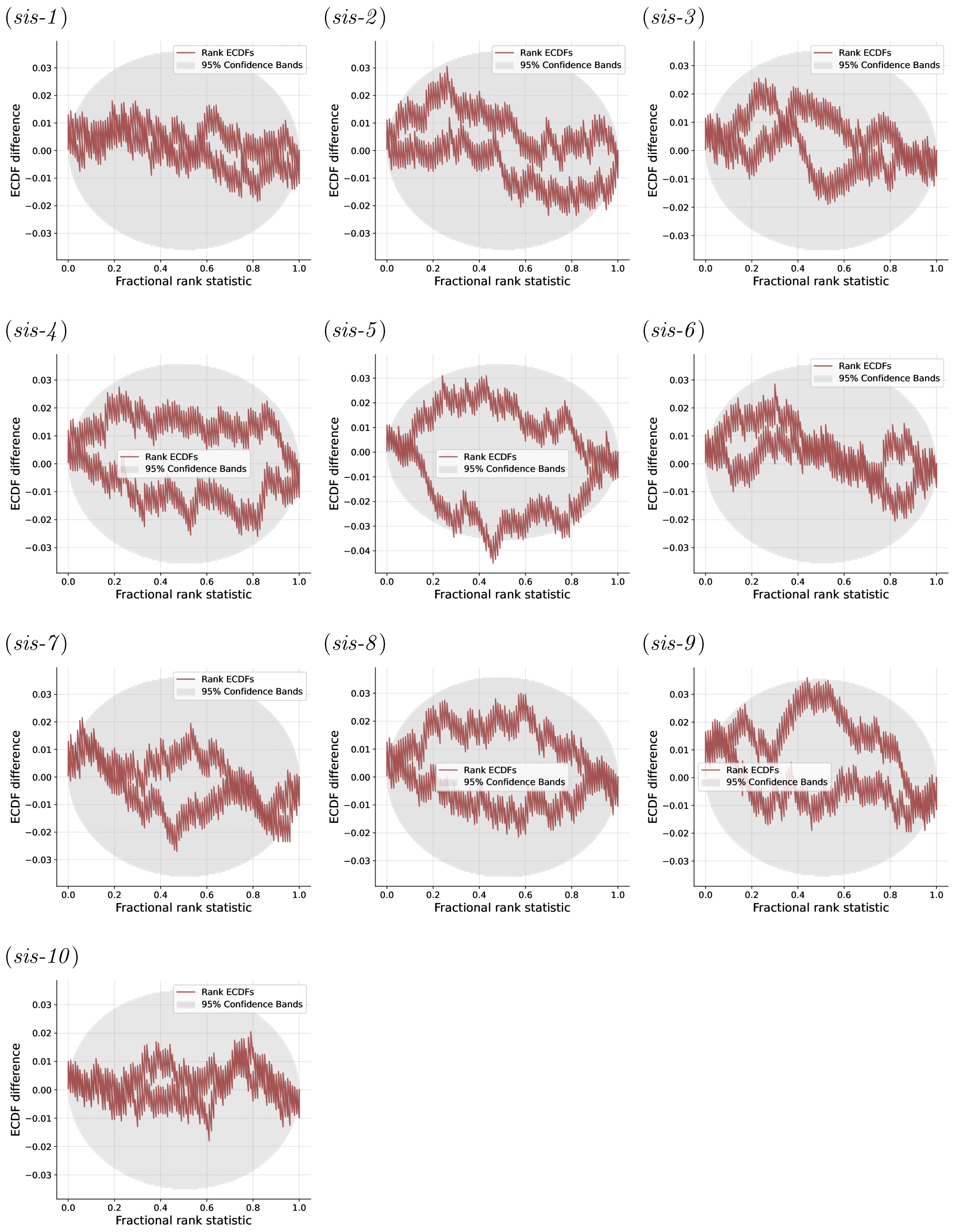

Figure S2.12: **ECDF Calibration plots for the SIS model.**

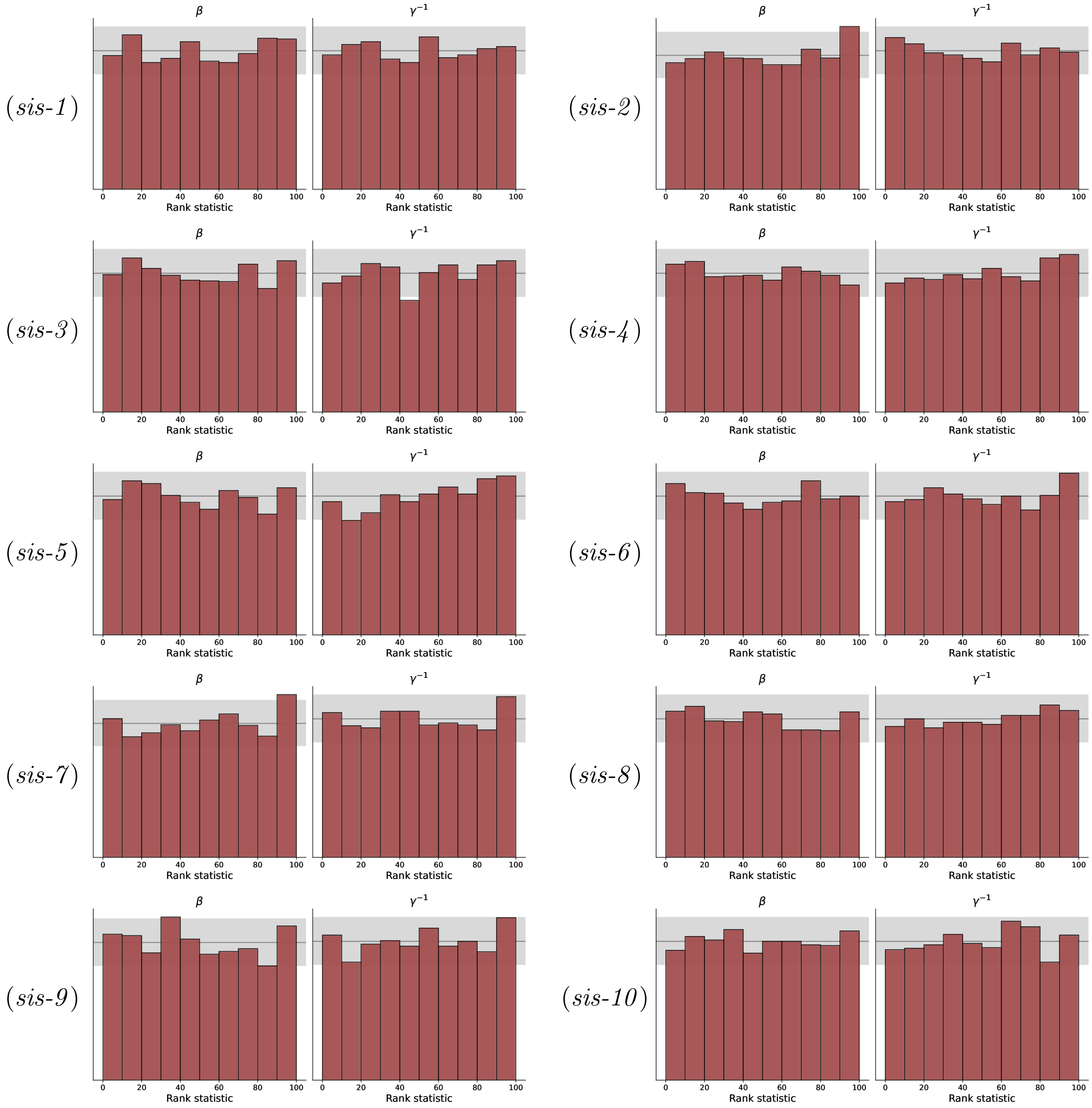

Figure S2.13: **SBC Histograms for the SIS model.**

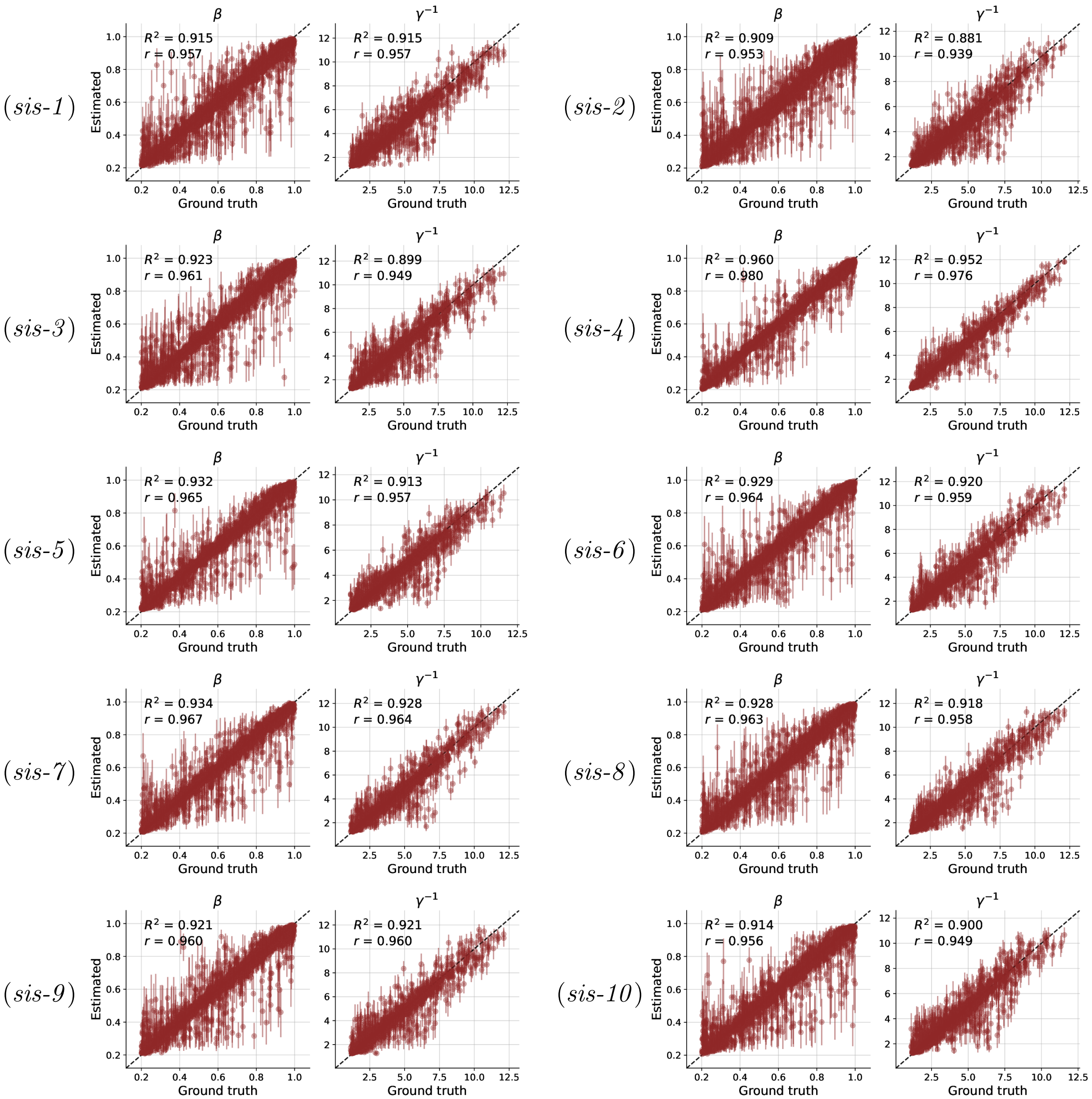

Figure S2.14: **Parameter recovery for the SIS model.**

S2.B Supplementary Tables

Table S2.1: Posterior MAP estimates with 95% intervals for SIS datasets *sis-1*–*sis-10*.

Dataset	Method	β	γ^{-1}
sis-1	True	0.5213	3.43
	CNF	0.5419 (0.49, 0.59)	3.29 (2.94, 3.71)
	PF	0.5392 (0.49, 0.59)	3.28 (2.91, 3.75)
	HMC	0.5298 (0.49, 0.59)	3.37 (2.93, 3.74)
sis-2	True	0.8004	2.49
	CNF	0.7665 (0.70, 0.83)	2.64 (2.39, 2.97)
	PF	0.7547 (0.70, 0.83)	2.72 (2.41, 2.99)
	HMC	0.7836 (0.72, 0.86)	2.60 (2.32, 2.91)
sis-3	True	0.6469	2.25
	CNF	0.6498 (0.56, 0.75)	2.22 (1.88, 2.62)
	PF	0.6448 (0.57, 0.75)	2.24 (1.88, 2.60)
	HMC	0.6602 (0.56, 0.74)	2.18 (1.90, 2.63)
sis-4	True	0.5453	2.17
	CNF	0.5500 (0.26, 0.91)	2.16 (1.23, 5.31)
	PF	0.5973 (0.31, 0.96)	1.96 (1.18, 4.12)
	HMC	0.6216 (0.32, 0.96)	1.88 (1.16, 3.96)
sis-5	True	0.9875	1.25
	CNF	0.9438 (0.79, 1.00)	1.32 (1.24, 1.60)
	PF	0.9482 (0.80, 1.00)	1.32 (1.25, 1.59)
	HMC	0.9687 (0.80, 1.00)	1.29 (1.25, 1.59)
sis-6	True	0.3839	4.10
	CNF	0.3723 (0.32, 0.44)	4.37 (3.51, 5.30)
	PF	0.3677 (0.32, 0.43)	4.39 (3.58, 5.34)
	HMC	0.3716 (0.32, 0.43)	4.34 (3.58, 5.33)
sis-7	True	0.3332	4.64
	CNF	0.3640 (0.29, 0.43)	4.22 (3.40, 5.69)
	PF	0.3496 (0.29, 0.43)	4.40 (3.37, 5.64)
	HMC	0.3625 (0.29, 0.43)	4.20 (3.38, 5.63)
sis-8	True	0.4416	3.83
	CNF	0.4774 (0.43, 0.54)	3.48 (3.00, 4.00)
	PF	0.4724 (0.42, 0.53)	3.50 (3.02, 4.04)
	HMC	0.4827 (0.43, 0.53)	3.41 (3.01, 4.02)
sis-9	True	0.3925	4.35
	CNF	0.3566 (0.31, 0.40)	5.05 (4.31, 6.04)
	PF	0.3571 (0.31, 0.40)	4.98 (4.31, 6.03)
	HMC	0.3551 (0.31, 0.40)	5.02 (4.28, 6.03)
sis-10	True	0.7037	2.24
	CNF	0.7138 (0.63, 0.79)	2.18 (1.93, 2.55)
	PF	0.7059 (0.63, 0.79)	2.21 (1.93, 2.55)
	HMC	0.7111 (0.63, 0.80)	2.20 (1.92, 2.54)

Table S2.2: **Effective sample sizes (ESS) per parameter for the SIS model.** ESS computed on the last 10,000 samples of the chains resulting from running the PF method on the SIS model and using a maximum lag size of 250 for the autocorrelation.

**Dataset**	$\boldsymbol{\beta}$	$\boldsymbol{\gamma^{-1}}$
sis-1	4638.8	4692.9
sis-2	6860.0	7073.3
sis-3	6664.3	6641.3
sis-4	4501.1	4524.1
sis-5	5138.9	5237.5
sis-6	7213.8	7186.9
sis-7	4681.3	4649.2
sis-8	5990.2	6085.0
sis-9	7317.5	7269.7
sis-10	7211.3	7208.7

Table S2.3: **$\hat{\mathbf{R}}$ diagnostics for the SIS model.**

**Dataset**	$\boldsymbol{\beta}$	$\boldsymbol{\gamma^{-1}}$
sis-1	1.001	1.001
sis-2	1.001	1.001
sis-3	1.001	1.001
sis-4	1.001	1.001
sis-5	1.002	1.002
sis-6	1.001	1.001
sis-7	1.002	1.002
sis-8	1.001	1.001
sis-9	1.001	1.001
sis-10	1.001	1.001
