# Supplementary material for: Assessment of simulation-based inference methods for stochastic compartmental models in epidemiological research
Source: PLoS One. 2026 Jul 13;21(7):e0353306. doi: 10.1371/journal.pone.0353306 (PMC13362117; doi:10.1371/journal.pone.0353306)
Supplement: S3 Result — (PDF) [file pone.0353306.s003.pdf]

S3 Supplementary Results SIR-Model
Assessment of Simulation-based Inference Methods for Stochastic Compartmental Models in Epidemiological Research

Vincent Wieland[1,2,◑], Nils Waßmuth[1,2,3,◑], Lorenzo Contento[1], Martin Kühn[1,2,3], and Jan Hasenauer[1,2,*]

[1]Bonn Center for Mathematical Life Sciences, University of Bonn, Bonn, Germany
[2]Life and Medical Science Institute, University of Bonn, Bonn, Germany
[3]Institute of Software Technology, Department for High-Performance Computing, German Aerospace Center (DLR), Cologne, Germany
[◑]These authors contributed equally to the work.
[*]To whom correspondence should be addressed; jan.hasenauer@uni-bonn.de.

June 26, 2026

Contents

S3.A Supplementary Figures 2
S3.B Supplementary Tables 12

S3.A Supplementary Figures

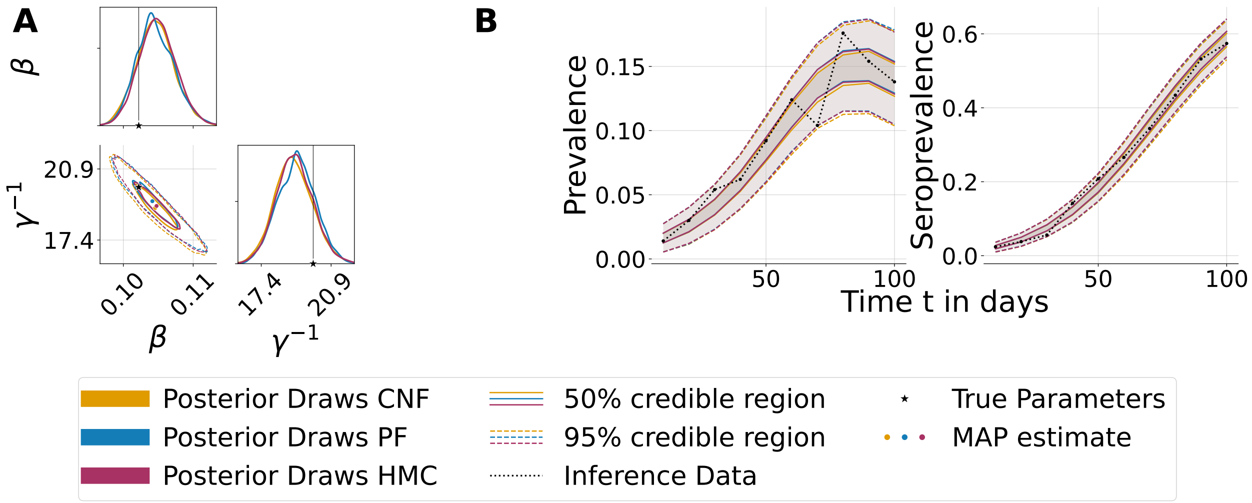

Figure S3.1: **Results of the SIR model for *sir-1*.**
A Posterior approximations from 10,000 samples. Contour gives the 50% (solid) and 95% (dashed) credible regions, coloured by method. Diagonals show the 1D marginals. Black stars mark the true parameters, coloured circles the joint MAP estimates. **B** Posterior predictive fit: bands give the 50% and 95% pointwise predictive intervals from the same samples (line styles as in **A**) with inference data shown as a dotted line.

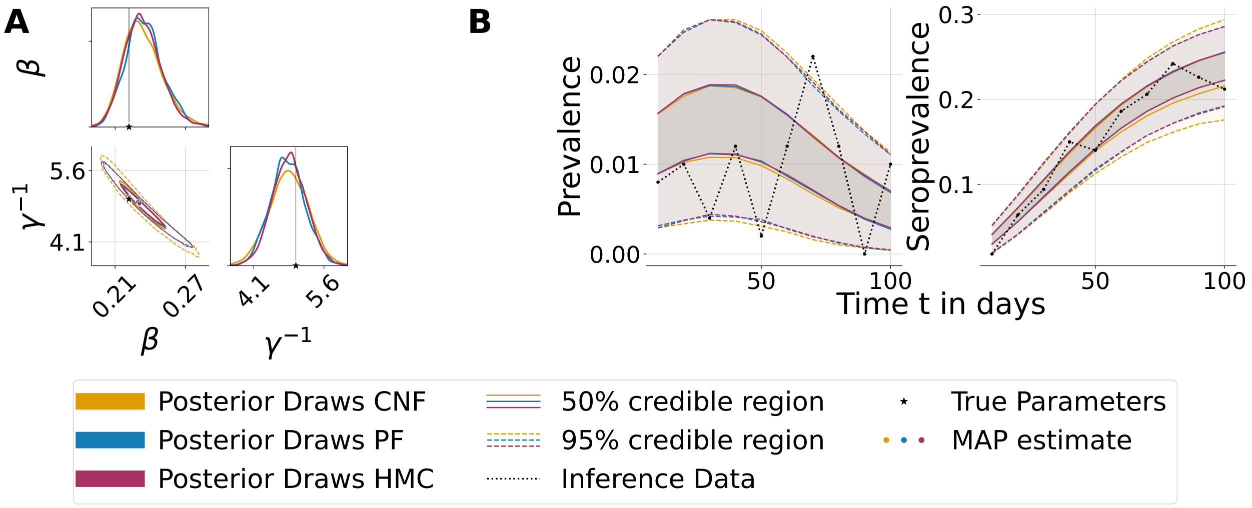

Figure S3.2: **Results of the SIR model for *sir-2*.**
A Posterior approximations from 10,000 samples. Contour gives the 50% (solid) and 95% (dashed) credible regions, coloured by method. Diagonals show the 1D marginals. Black stars mark the true parameters, coloured circles the joint MAP estimates. **B** Posterior predictive fit: bands give the 50% and 95% pointwise predictive intervals from the same samples (line styles as in **A**) with inference data shown as a dotted line.

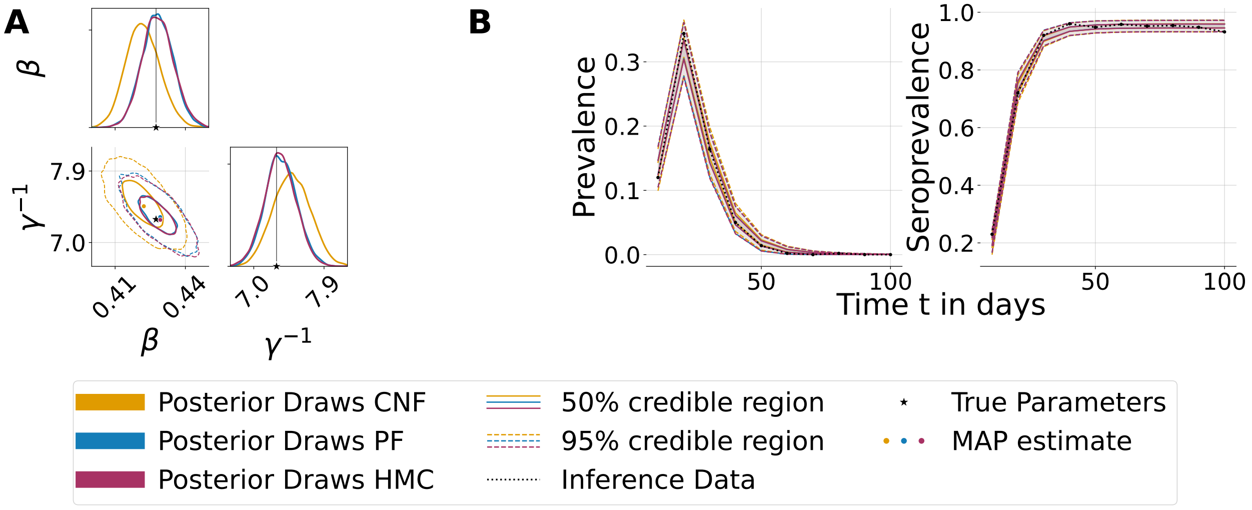

Figure S3.3: **Results of the SIR model for *sir-3*.**
A Posterior approximations from 10,000 samples. Contour gives the 50% (solid) and 95% (dashed) credible regions, coloured by method. Diagonals show the 1D marginals. Black stars mark the true parameters, coloured circles the joint MAP estimates. **B** Posterior predictive fit: bands give the 50% and 95% pointwise predictive intervals from the same samples (line styles as in **A**) with inference data shown as a dotted line.

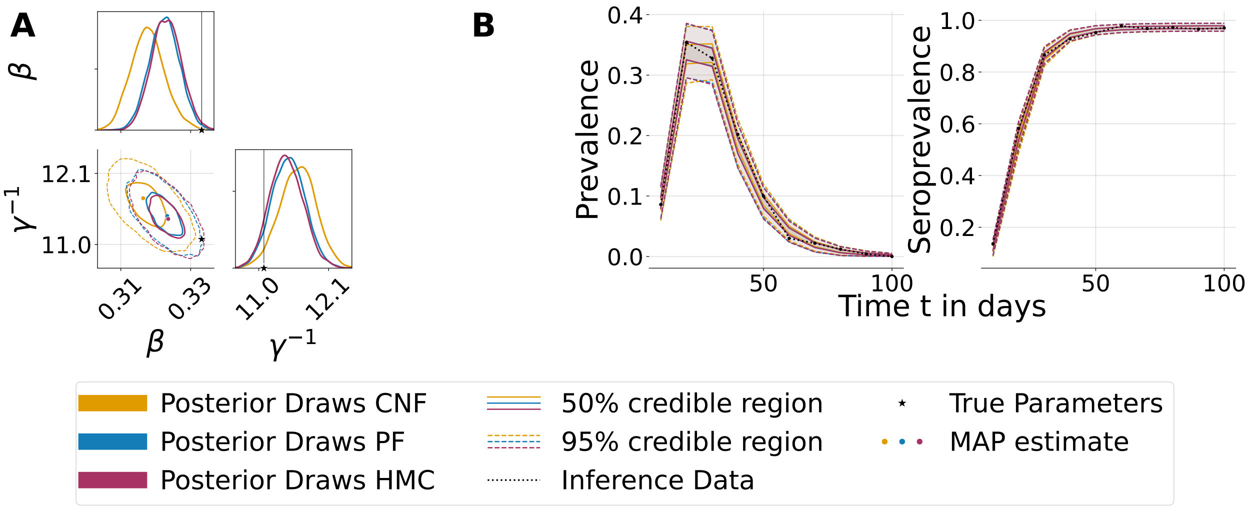

Figure S3.4: **Results of the SIR model for *sir-4*.**
A Posterior approximations from 10,000 samples. Contour gives the 50% (solid) and 95% (dashed) credible regions, coloured by method. Diagonals show the 1D marginals. Black stars mark the true parameters, coloured circles the joint MAP estimates. **B** Posterior predictive fit: bands give the 50% and 95% pointwise predictive intervals from the same samples (line styles as in **A**) with inference data shown as a dotted line.

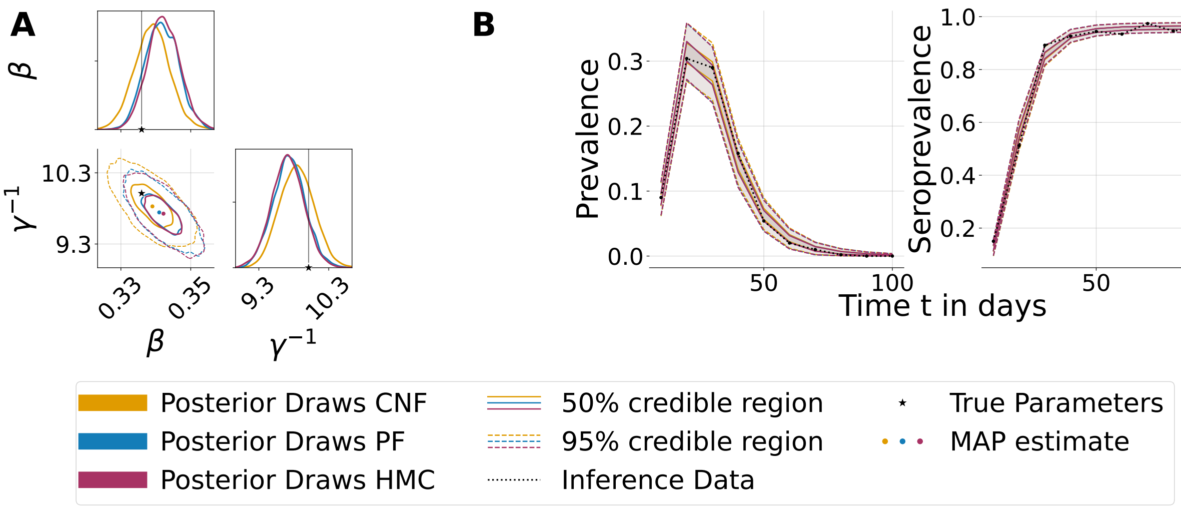

Figure S3.5: **Results of the SIR model for *sir-5*.**
A Posterior approximations from 10,000 samples. Contour gives the 50% (solid) and 95% (dashed) credible regions, coloured by method. Diagonals show the 1D marginals. Black stars mark the true parameters, coloured circles the joint MAP estimates. **B** Posterior predictive fit: bands give the 50% and 95% pointwise predictive intervals from the same samples (line styles as in **A**) with inference data shown as a dotted line.

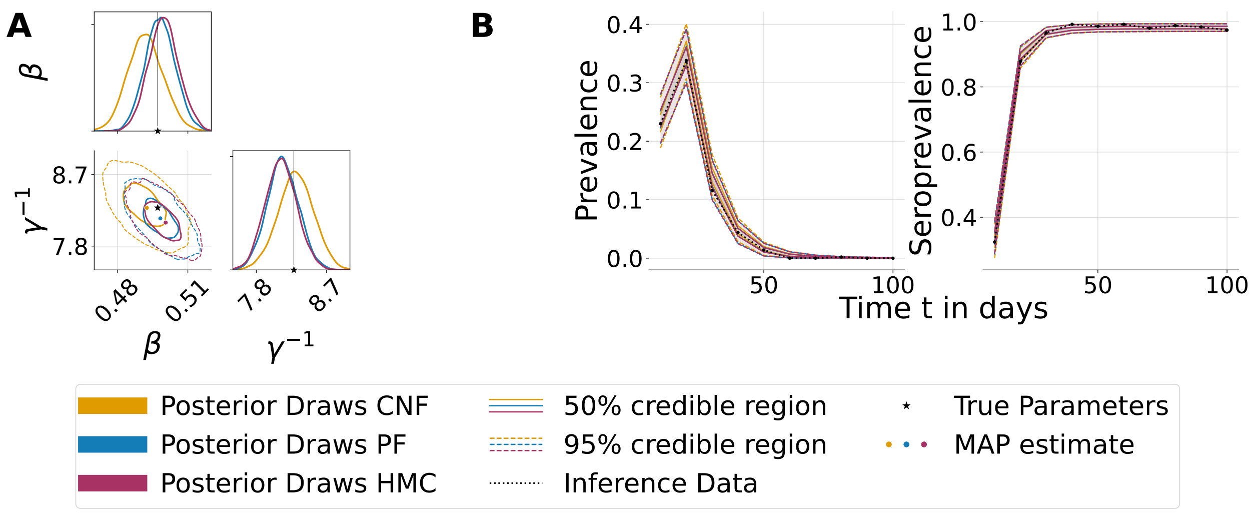

Figure S3.6: **Results of the SIR model for *sir-6*.**
A Posterior approximations from 10,000 samples. Contour gives the 50% (solid) and 95% (dashed) credible regions, coloured by method. Diagonals show the 1D marginals. Black stars mark the true parameters, coloured circles the joint MAP estimates. **B** Posterior predictive fit: bands give the 50% and 95% pointwise predictive intervals from the same samples (line styles as in **A**) with inference data shown as a dotted line.

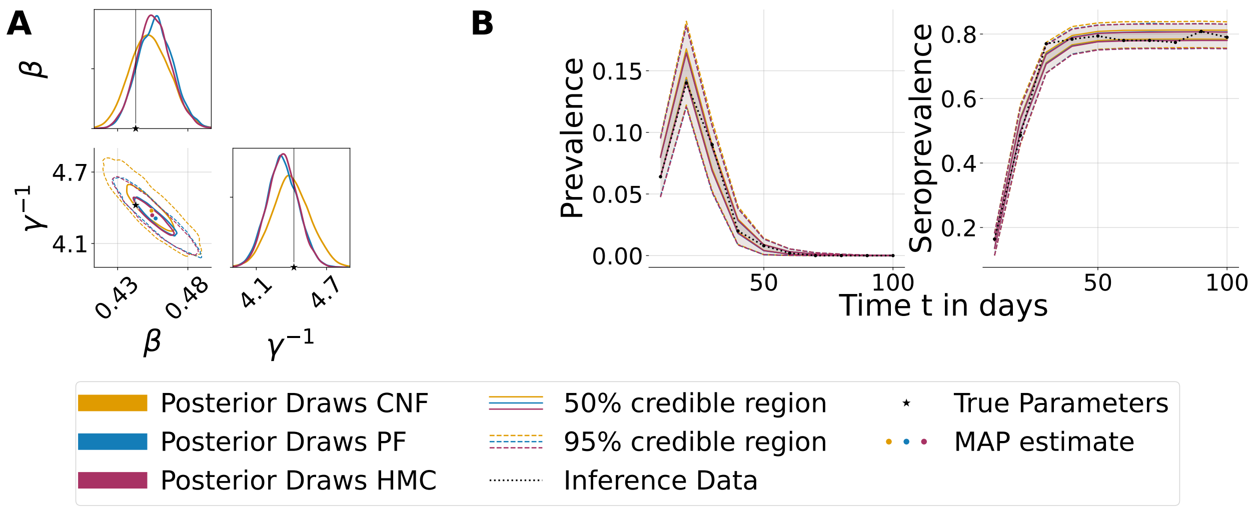

Figure S3.7: **Results of the SIR model for *sir-7*.**
A Posterior approximations from 10,000 samples. Contour gives the 50% (solid) and 95% (dashed) credible regions, coloured by method. Diagonals show the 1D marginals. Black stars mark the true parameters, coloured circles the joint MAP estimates. **B** Posterior predictive fit: bands give the 50% and 95% pointwise predictive intervals from the same samples (line styles as in **A**) with inference data shown as a dotted line.

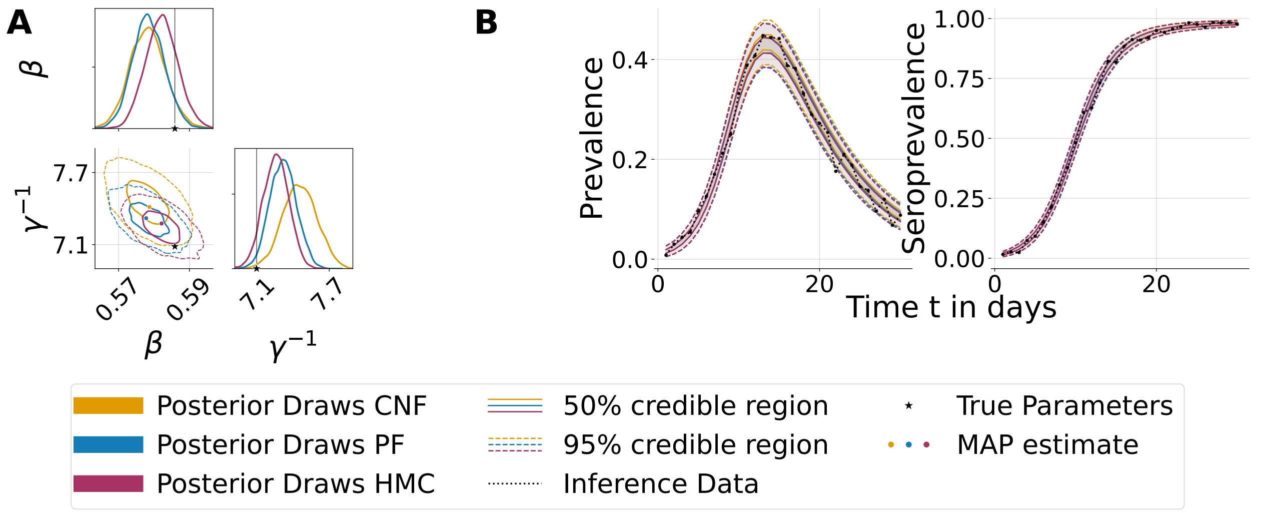

Figure S3.8: **Results of the SIR model for *sir-8*.**
A Posterior approximations from 10,000 samples. Contour gives the 50% (solid) and 95% (dashed) credible regions, coloured by method. Diagonals show the 1D marginals. Black stars mark the true parameters, coloured circles the joint MAP estimates. **B** Posterior predictive fit: bands give the 50% and 95% pointwise predictive intervals from the same samples (line styles as in **A**) with inference data shown as a dotted line.

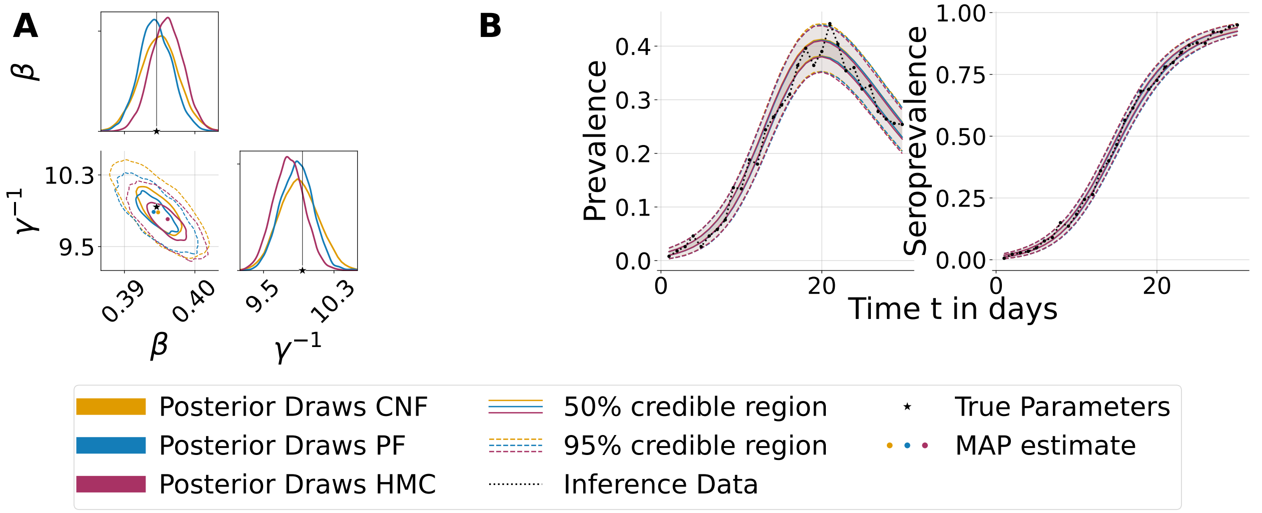

Figure S3.9: **Results of the SIR model for *sir-9*.**
A Posterior approximations from 10,000 samples. Contour gives the 50% (solid) and 95% (dashed) credible regions, coloured by method. Diagonals show the 1D marginals. Black stars mark the true parameters, coloured circles the joint MAP estimates. **B** Posterior predictive fit: bands give the 50% and 95% pointwise predictive intervals from the same samples (line styles as in **A**) with inference data shown as a dotted line.

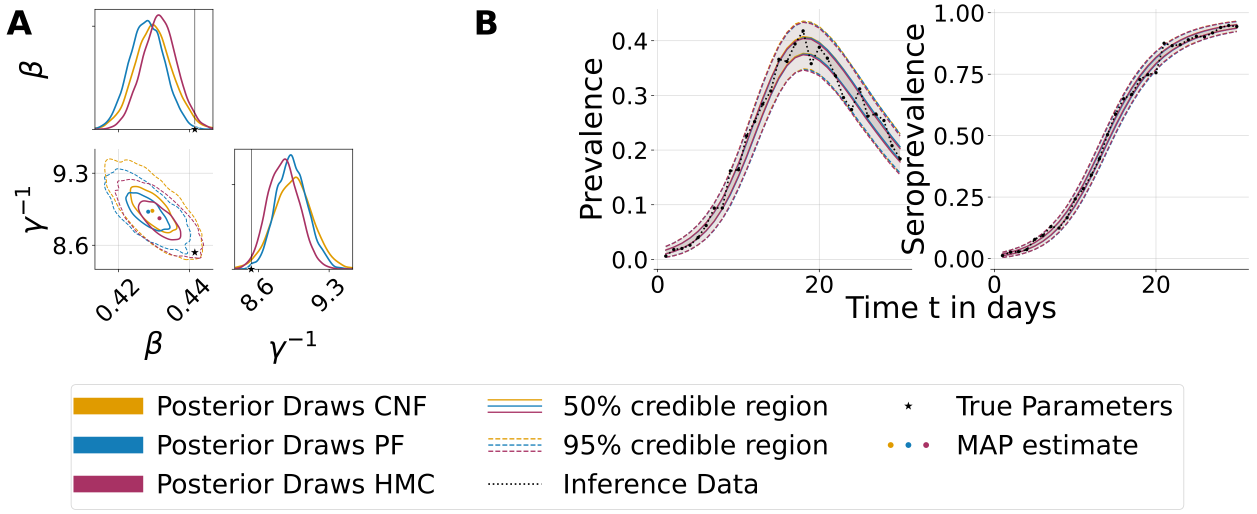

Figure S3.10: **Results of the SIR model for *sir-10*.**
A Posterior approximations from 10,000 samples. Contour gives the 50% (solid) and 95% (dashed) credible regions, coloured by method. Diagonals show the 1D marginals. Black stars mark the true parameters, coloured circles the joint MAP estimates. **B** Posterior predictive fit: bands give the 50% and 95% pointwise predictive intervals from the same samples (line styles as in **A**) with inference data shown as a dotted line.

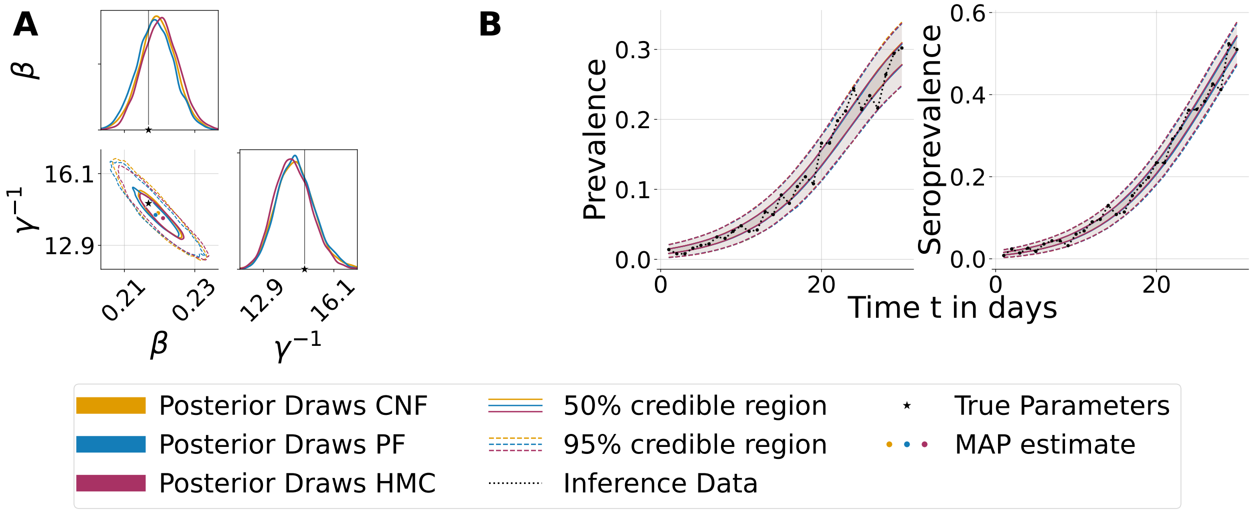

Figure S3.11: **Results of the SIR model for *sir-11*.**
A Posterior approximations from 10,000 samples. Contour gives the 50% (solid) and 95% (dashed) credible regions, coloured by method. Diagonals show the 1D marginals. Black stars mark the true parameters, coloured circles the joint MAP estimates. **B** Posterior predictive fit: bands give the 50% and 95% pointwise predictive intervals from the same samples (line styles as in **A**) with inference data shown as a dotted line.

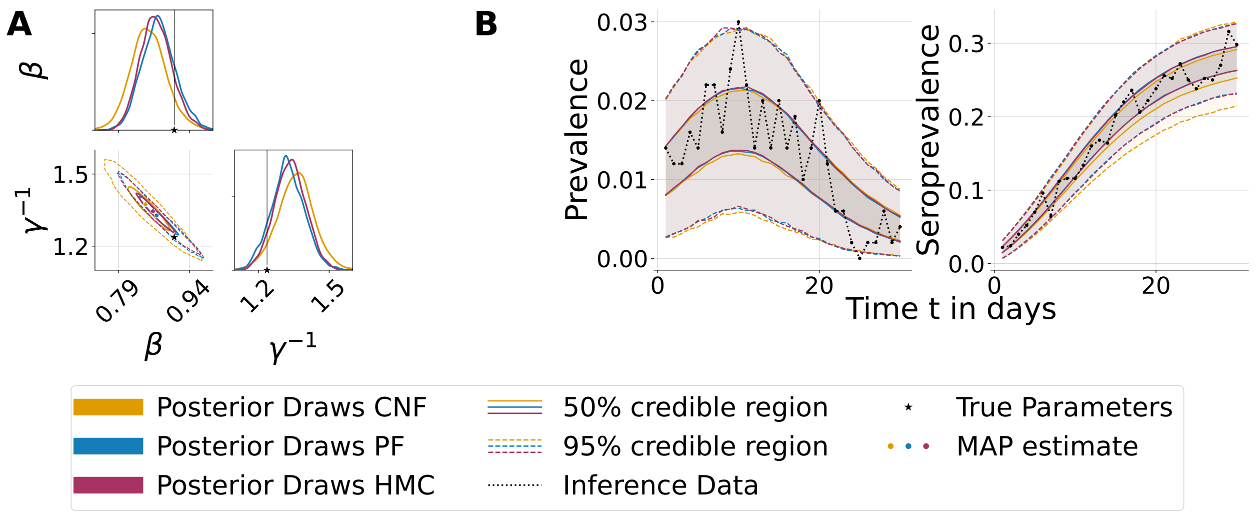

Figure S3.12: **Results of the SIR model for *sir-12*.**
A Posterior approximations from 10,000 samples. Contour gives the 50% (solid) and 95% (dashed) credible regions, coloured by method. Diagonals show the 1D marginals. Black stars mark the true parameters, coloured circles the joint MAP estimates. **B** Posterior predictive fit: bands give the 50% and 95% pointwise predictive intervals from the same samples (line styles as in **A**) with inference data shown as a dotted line.

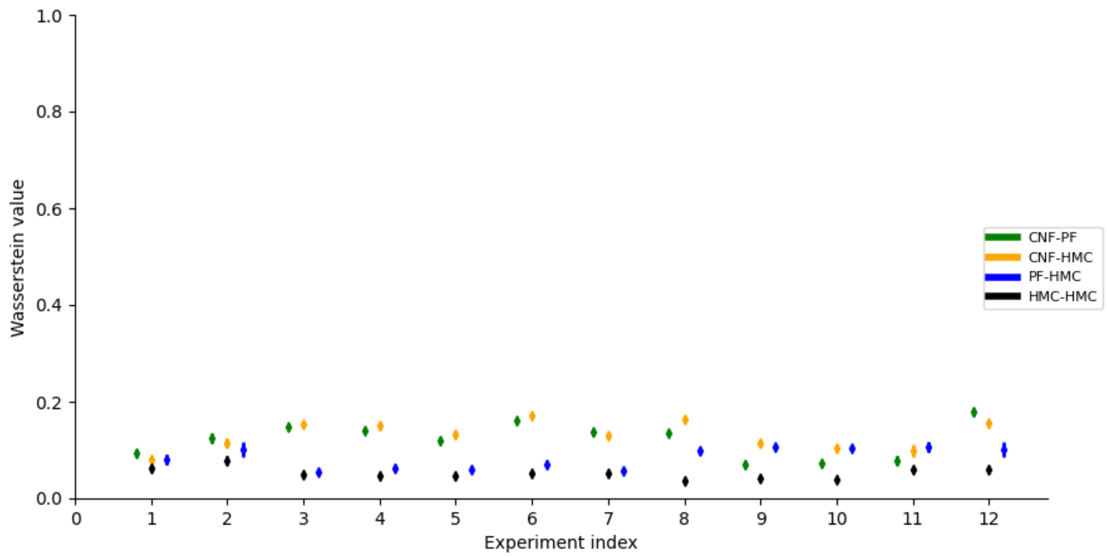

Figure S3.13: **Wasserstein distances for the SIR model**
Pairwise 1-Wasserstein distances between posterior samples across experiment indices for all method combinations. Error bars show Monte-Carlo variability.

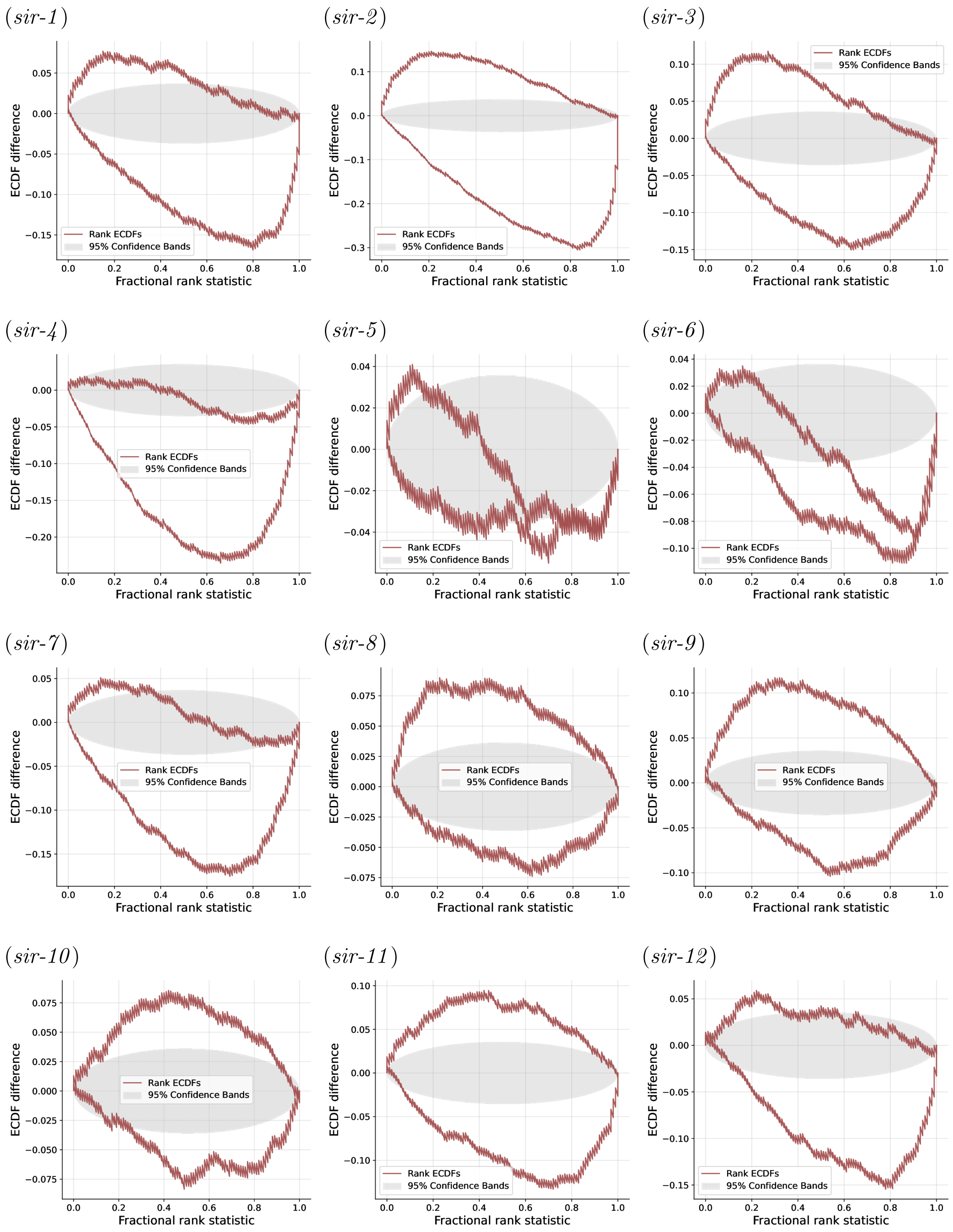

Figure S3.14: **ECDF Calibration plots for the SIR model.**

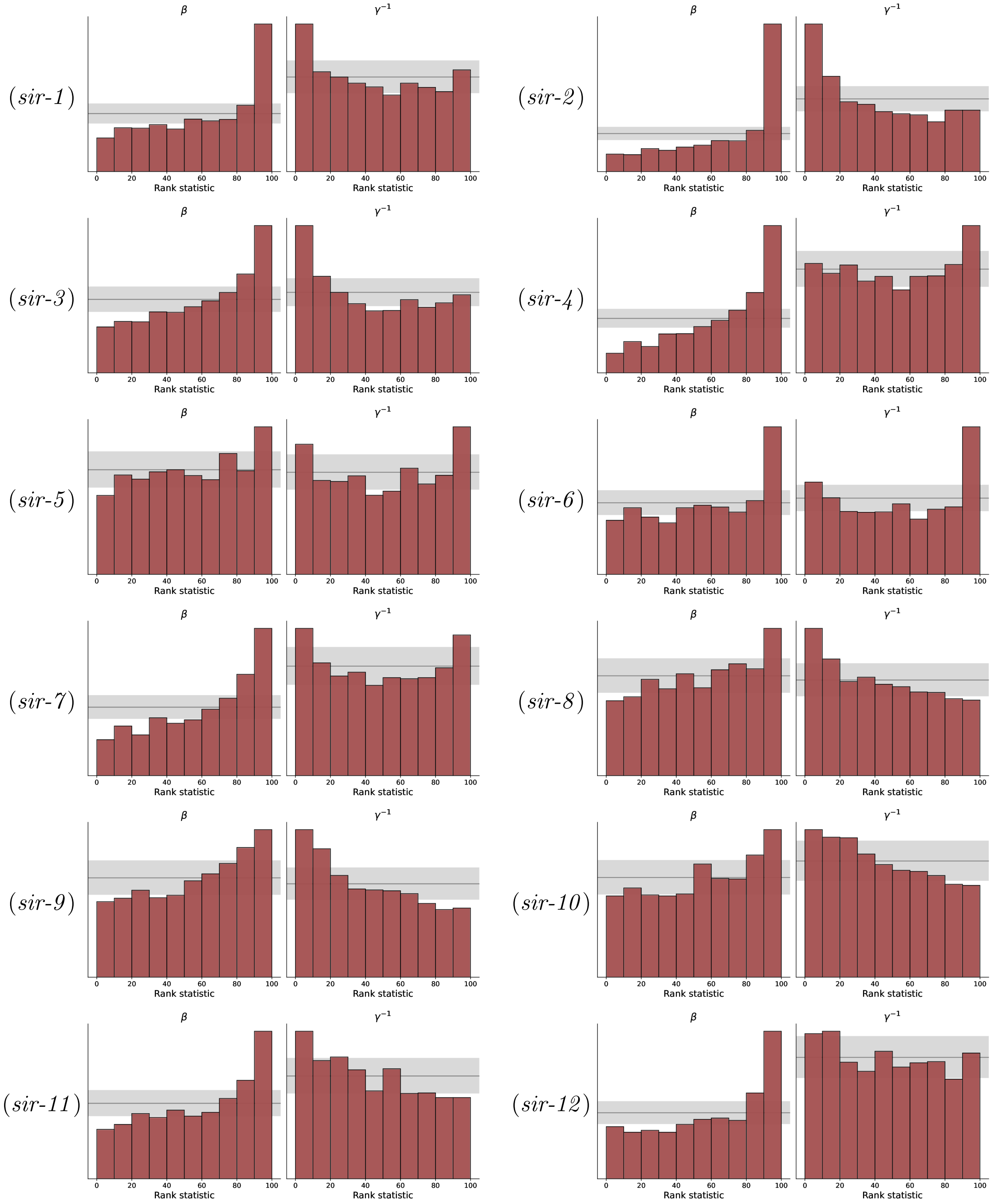

Figure S3.15: **SBC Histograms for the SIR model.**

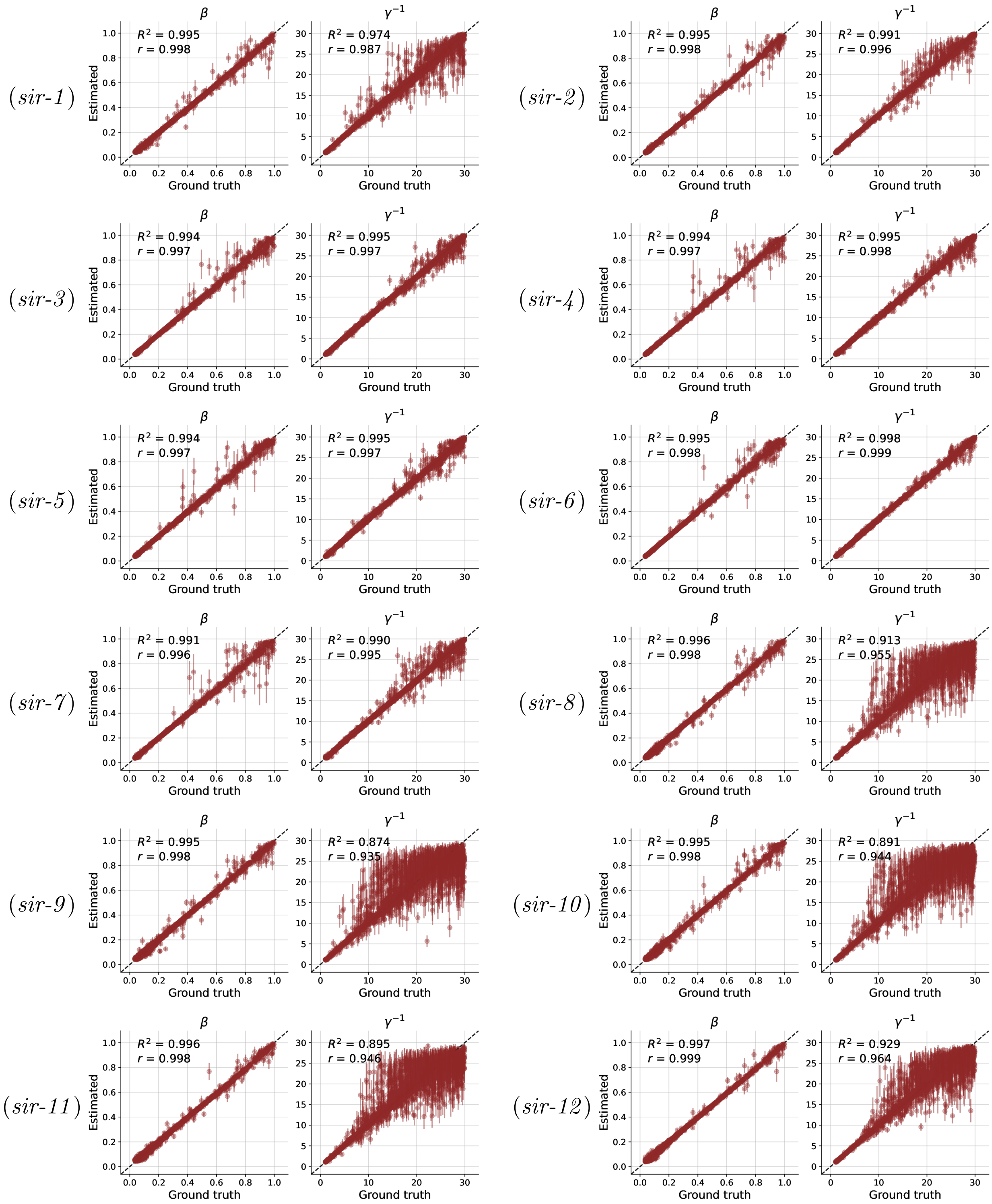

Figure S3.16: **Parameter recovery for the SIR model.**

S3.B Supplementary Tables

Table S3.1: Posterior MAP estimates with 95% intervals for SIR datasets *sir-1*–*sir-7*.

Dataset	Method	β	γ^{-1}
sir-1	True	0.1000	20.00
	CNF	0.1026 (0.10,0.11)	18.99 (17.22,20.98)
	PF	0.1021 (0.10,0.11)	19.32 (17.34,21.01)
	HMC	0.1028 (0.10,0.11)	19.08 (17.33,20.97)
sir-2	True	0.2200	5.00
	CNF	0.2284 (0.20,0.27)	4.90 (4.04,5.64)
	PF	0.2277 (0.20,0.27)	4.94 (4.16,5.54)
	HMC	0.2290 (0.20,0.27)	4.91 (4.17,5.57)
sir-3	True	0.4306	7.31
	CNF	0.4253 (0.41,0.44)	7.46 (7.07,7.90)
	PF	0.4324 (0.42,0.44)	7.33 (7.01,7.73)
	HMC	0.4325 (0.42,0.45)	7.30 (6.99,7.71)
sir-4	True	0.3346	11.05
	CNF	0.3180 (0.31,0.33)	11.70 (11.00,12.17)
	PF	0.3248 (0.32,0.33)	11.42 (10.93,11.98)
	HMC	0.3251 (0.32,0.33)	11.37 (10.91,11.96)
sir-5	True	0.3329	10.05
	CNF	0.3361 (0.33,0.35)	9.86 (9.40,10.38)
	PF	0.3381 (0.33,0.35)	9.78 (9.31,10.25)
	HMC	0.3394 (0.33,0.35)	9.76 (9.29,10.21)
sir-6	True	0.4962	8.29
	CNF	0.4908 (0.47,0.51)	8.29 (7.85,8.76)
	PF	0.4975 (0.48,0.51)	8.16 (7.76,8.56)
	HMC	0.5002 (0.48,0.51)	8.10 (7.74,8.53)

Table S3.2: Posterior MAP estimates with 95% intervals for SIR datasets *sir-7*–*sir-12*.

Dataset	Method	β	γ^{-1}
sir-7	True	0.4462	4.40
	CNF	0.4561 (0.43,0.48)	4.35 (4.07,4.69)
	PF	0.4586 (0.44,0.48)	4.29 (4.05,4.56)
	HMC	0.4565 (0.44,0.48)	4.32 (4.06,4.56)
sir-8	True	0.5871	7.13
	CNF	0.5802 (0.57,0.59)	7.44 (7.20,7.74)
	PF	0.5793 (0.57,0.59)	7.35 (7.14,7.55)
	HMC	0.5835 (0.58,0.59)	7.31 (7.08,7.48)
sir-9	True	0.3920	9.94
	CNF	0.3924 (0.38,0.40)	9.88 (9.46,10.35)
	PF	0.3914 (0.39,0.40)	9.88 (9.50,10.23)
	HMC	0.3944 (0.39,0.40)	9.79 (9.42,10.14)
sir-10	True	0.4380	8.51
	CNF	0.4290 (0.42,0.44)	8.90 (8.54,9.28)
	PF	0.4282 (0.42,0.44)	8.89 (8.61,9.20)
	HMC	0.4305 (0.42,0.44)	8.83 (8.54,9.11)
sir-11	True	0.2193	14.75
	CNF	0.2218 (0.21,0.23)	14.32 (12.77,16.24)
	PF	0.2211 (0.21,0.23)	14.24 (12.85,16.18)
	HMC	0.2231 (0.21,0.23)	14.10 (12.74,15.94)
sir-12	True	0.9121	1.24
	CNF	0.8508 (0.79,0.94)	1.35 (1.21,1.46)
	PF	0.8747 (0.81,0.95)	1.31 (1.20,1.42)
	HMC	0.8668 (0.81,0.94)	1.33 (1.21,1.42)

Table S3.3: **Effective sample sizes (ESS) per parameter and data for the SIR model.** ESS computed on the last 10,000 samples of the chains resulting from running the PF method on the standard SIR model and using a maximum lag size of 250 for the autocorrelation.

**Dataset**	$\boldsymbol{\beta}$	$\boldsymbol{\gamma^{-1}}$
sir-1	5189.6	5131.9
sir-2	2259.2	2256.6
sir-3	7162.1	6531.6
sir-4	7307.2	7176.5
sir-5	5045.7	5055.4
sir-6	6496.6	7255.0
sir-7	5929.0	5934.0
sir-8	4668.5	4380.9
sir-9	5075.1	5066.2
sir-10	5983.1	6051.6
sir-11	3458.6	3439.2
sir-12	2468.8	2449.7

Table S3.4: **$\hat{\mathbf{R}}$ diagnostics for the SIR model.**

**Dataset**	$\boldsymbol{\beta}$	$\boldsymbol{\gamma^{-1}}$
sir-1	1.001	1.000
sir-2	1.003	1.003
sir-3	1.001	1.001
sir-4	1.001	1.001
sir-5	1.001	1.001
sir-6	1.001	1.001
sir-7	1.001	1.001
sir-8	1.001	1.003
sir-9	1.001	1.001
sir-10	1.001	1.001
sir-11	1.001	1.001
sir-12	1.000	1.000
