# Supplementary material for: Assessment of simulation-based inference methods for stochastic compartmental models in epidemiological research
Source: PLoS One. 2026 Jul 13;21(7):e0353306. doi: 10.1371/journal.pone.0353306 (PMC13362117; doi:10.1371/journal.pone.0353306)
Supplement: S4 Result — (PDF) [file pone.0353306.s004.pdf]

S4 Supplementary Results SEIR-Model with Dense Data
Assessment of Simulation-based Inference Methods for Stochastic Compartmental Models in Epidemiological Research

Vincent Wieland[1,2,◑], Nils Waßmuth[1,2,3,◑], Lorenzo Contento[1], Martin Kühn[1,2,3], and Jan Hasenauer[1,2,*]

[1]Bonn Center for Mathematical Life Sciences, University of Bonn, Bonn, Germany
[2]Life and Medical Science Institute, University of Bonn, Bonn, Germany
[3]Institute of Software Technology, Department for High-Performance Computing, German Aerospace Center (DLR), Cologne, Germany
[◑]These authors contributed equally to the work.
[*]To whom correspondence should be addressed; jan.hasenauer@uni-bonn.de.

June 26, 2026

Contents

S4.A Supplementary Figures 2
S4.B Supplementary Tables 23

S4.A Supplementary Figures

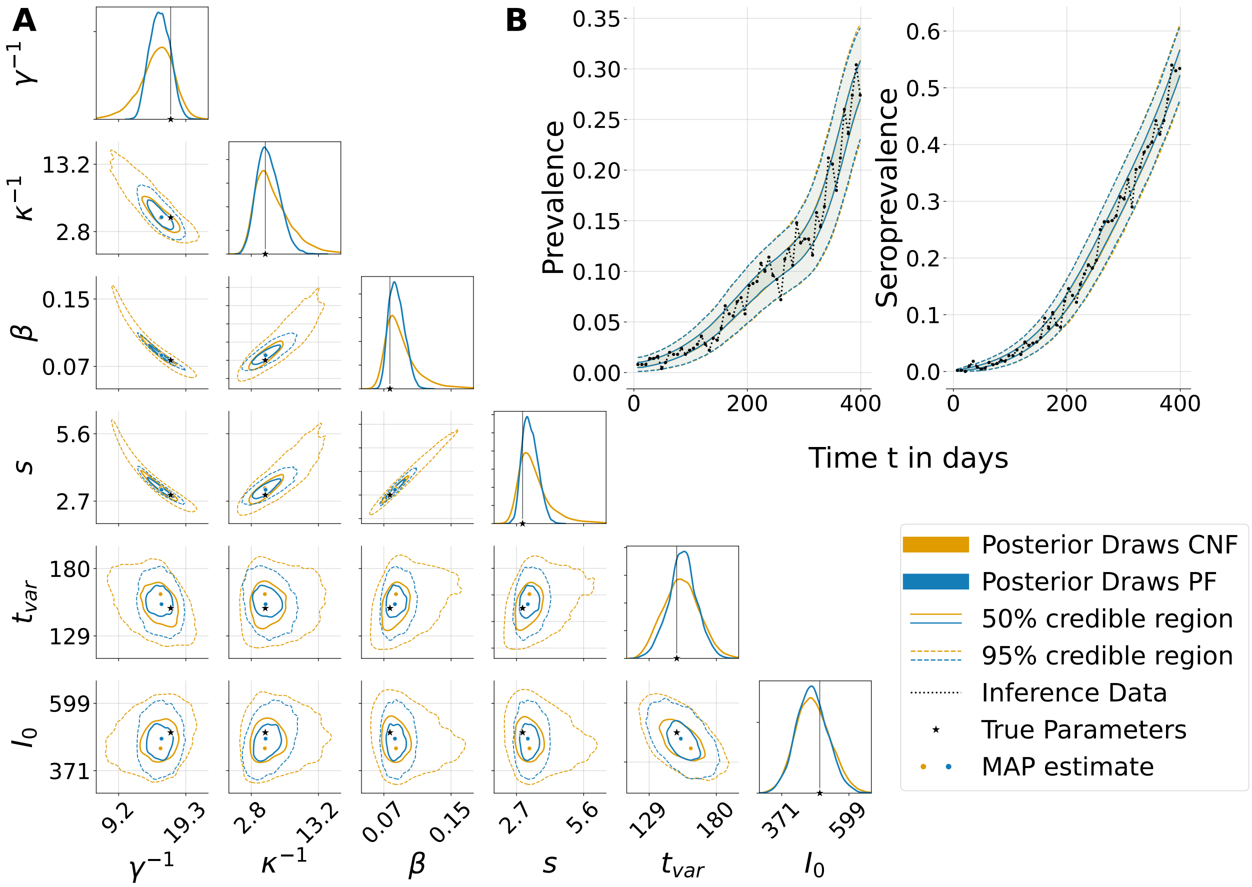

Figure S4.1: **Results of the two-variant SEIR model for *d-1-1*.**
A Posterior approximations from 10,000 samples. Contour gives the 50% (solid) and 95% (dashed) credible regions, coloured by method. Diagonals show the 1D marginals. Black stars mark the true parameters, coloured circles the joint MAP estimates. **B** Posterior predictive fit: bands give the 50% and 95% pointwise predictive intervals from the same samples (line styles as in **A**) with inference data shown as a dotted line.

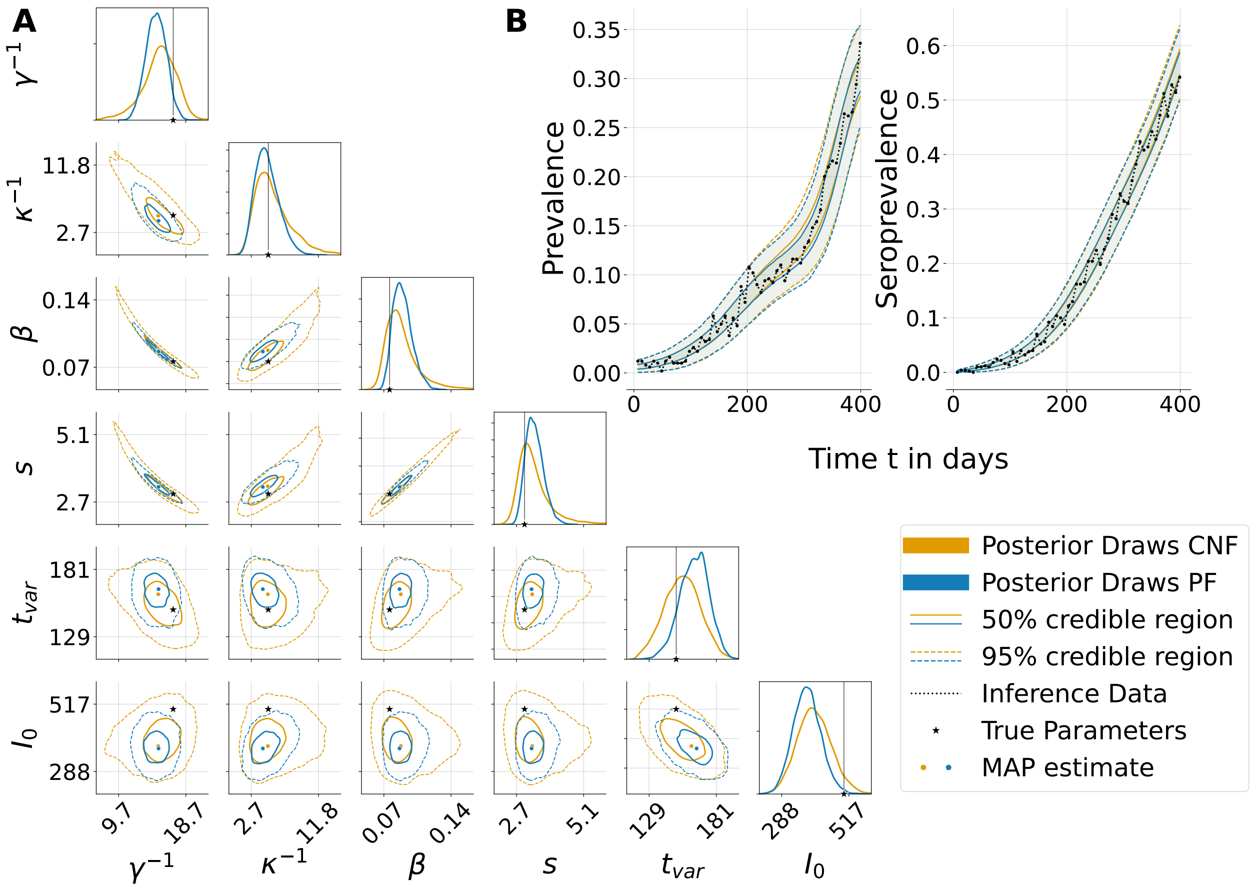

Figure S4.2: **Results of the two-variant SEIR model for *d-1-2*.**
A Posterior approximations from 10,000 samples. Contour gives the 50% (solid) and 95% (dashed) credible regions, coloured by method. Diagonals show the 1D marginals. Black stars mark the true parameters, coloured circles the joint MAP estimates. **B** Posterior predictive fit: bands give the 50% and 95% pointwise predictive intervals from the same samples (line styles as in **A**) with inference data shown as a dotted line.

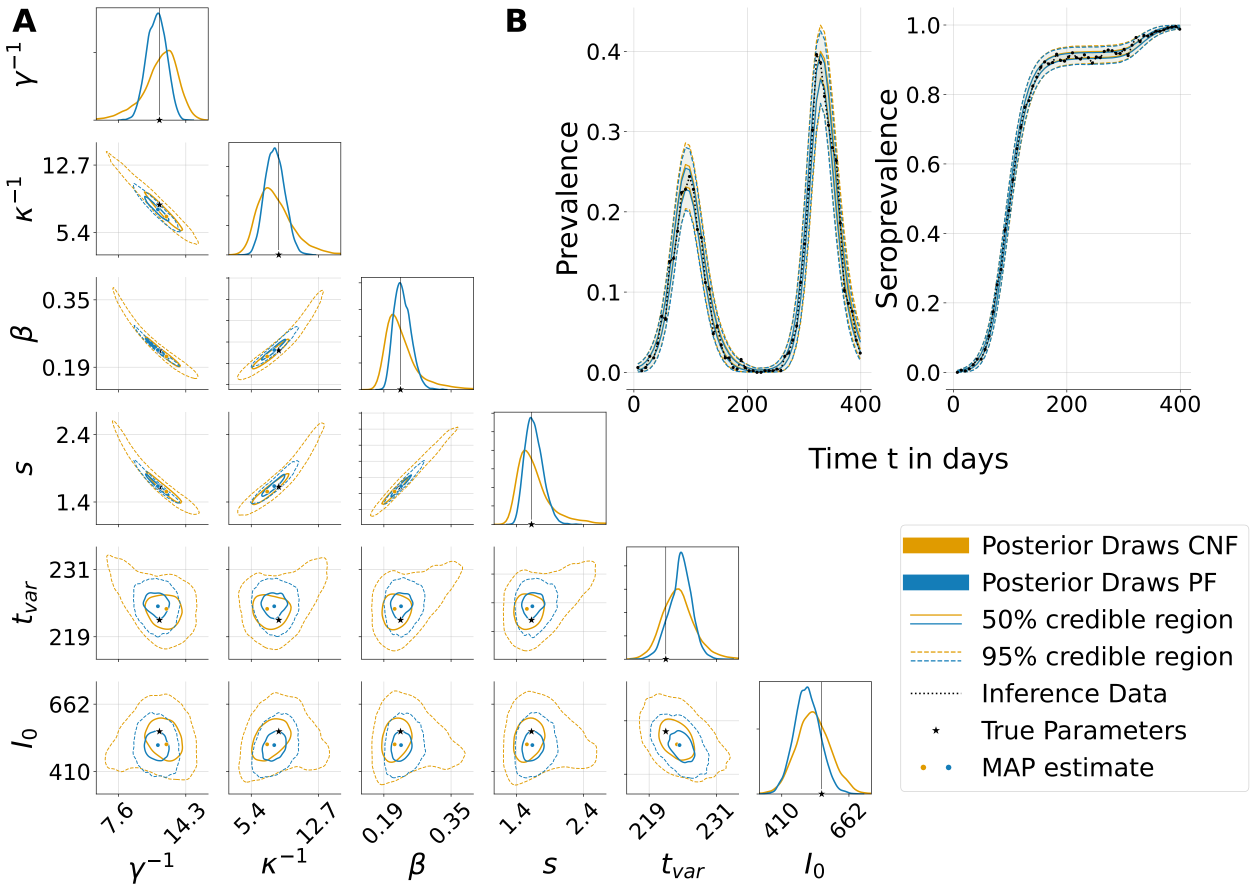

Figure S4.3: **Results of the two-variant SEIR model for *d-2-1*.**
A Posterior approximations from 10,000 samples. Contour gives the 50% (solid) and 95% (dashed) credible regions, coloured by method. Diagonals show the 1D marginals. Black stars mark the true parameters, coloured circles the joint MAP estimates. **B** Posterior predictive fit: bands give the 50% and 95% pointwise predictive intervals from the same samples (line styles as in **A**) with inference data shown as a dotted line.

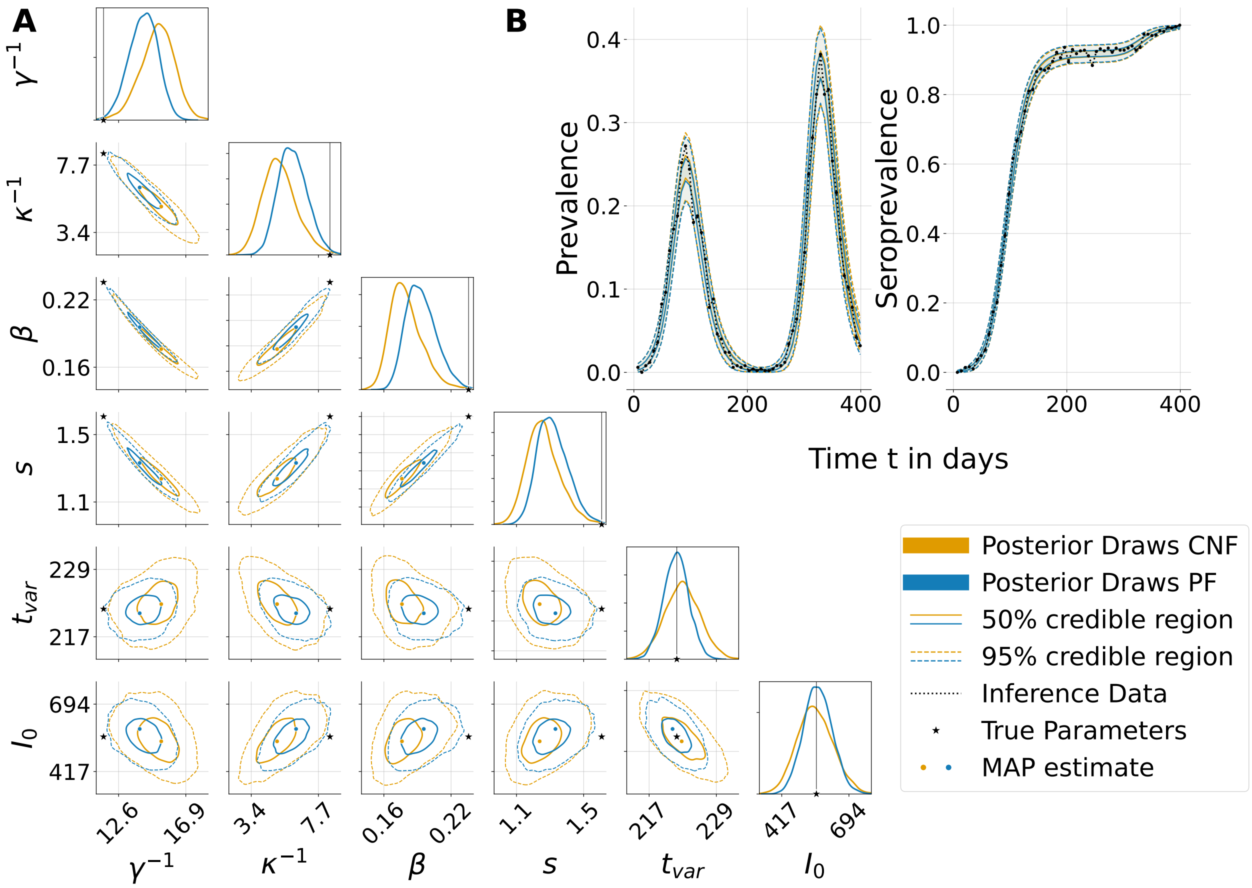

Figure S4.4: **Results of the two-variant SEIR model for *d-2-2*.**
A Posterior approximations from 10,000 samples. Contour gives the 50% (solid) and 95% (dashed) credible regions, coloured by method. Diagonals show the 1D marginals. Black stars mark the true parameters, coloured circles the joint MAP estimates. **B** Posterior predictive fit: bands give the 50% and 95% pointwise predictive intervals from the same samples (line styles as in **A**) with inference data shown as a dotted line.

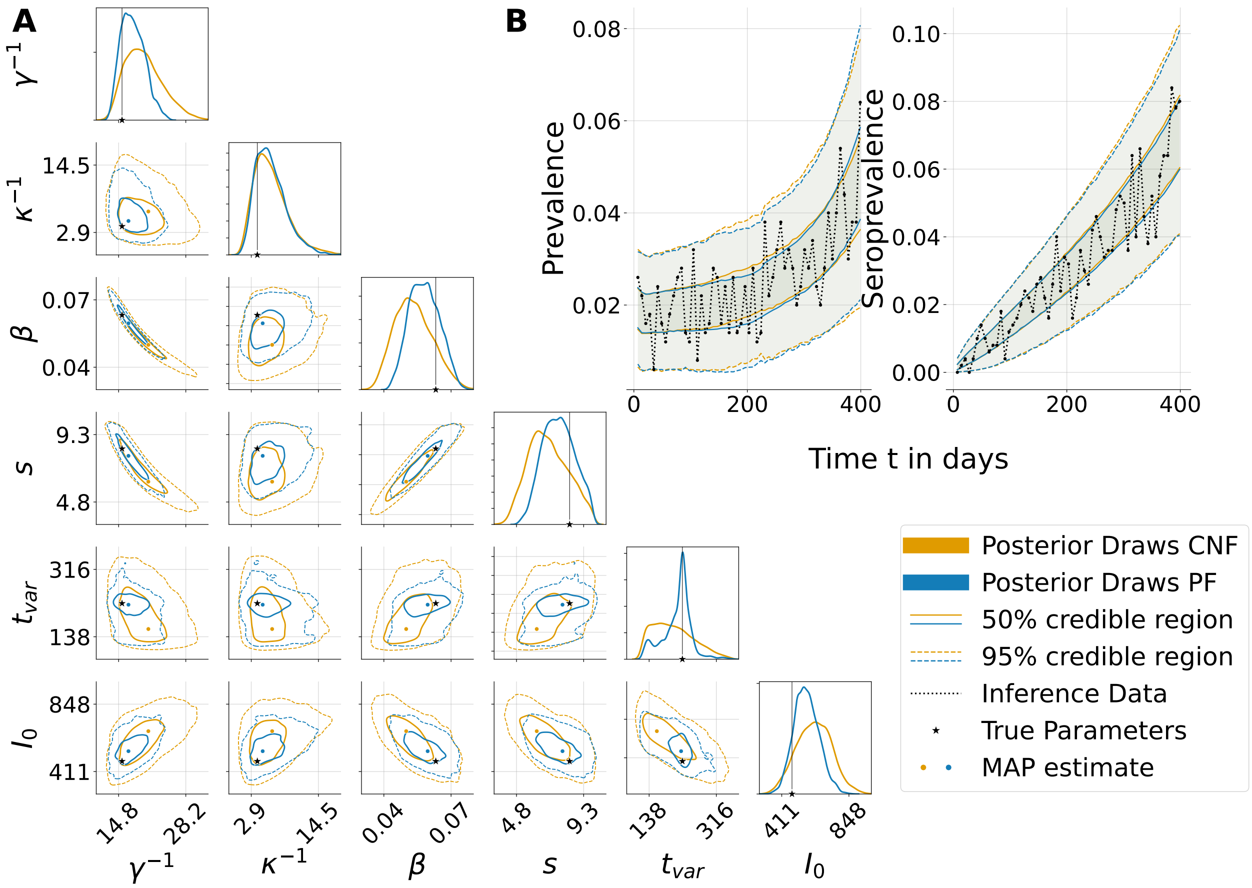

Figure S4.5: **Results of the two-variant SEIR model for *d-3*.**
A Posterior approximations from 10,000 samples. Contour gives the 50% (solid) and 95% (dashed) credible regions, coloured by method. Diagonals show the 1D marginals. Black stars mark the true parameters, coloured circles the joint MAP estimates. **B** Posterior predictive fit: bands give the 50% and 95% pointwise predictive intervals from the same samples (line styles as in **A**) with inference data shown as a dotted line.

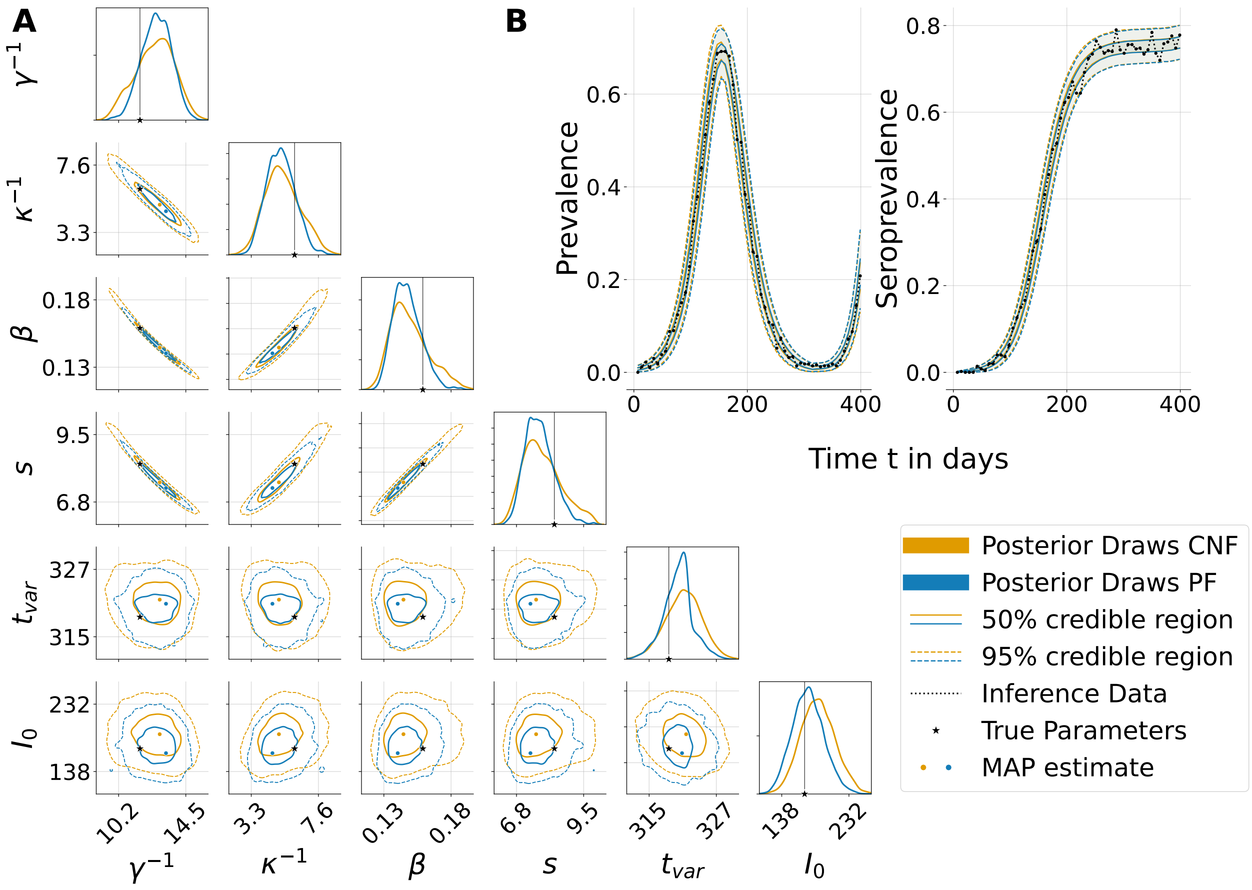

Figure S4.6: **Results of the two-variant SEIR model for *d-4*.**
A Posterior approximations from 10,000 samples. Contour gives the 50% (solid) and 95% (dashed) credible regions, coloured by method. Diagonals show the 1D marginals. Black stars mark the true parameters, coloured circles the joint MAP estimates. **B** Posterior predictive fit: bands give the 50% and 95% pointwise predictive intervals from the same samples (line styles as in **A**) with inference data shown as a dotted line.

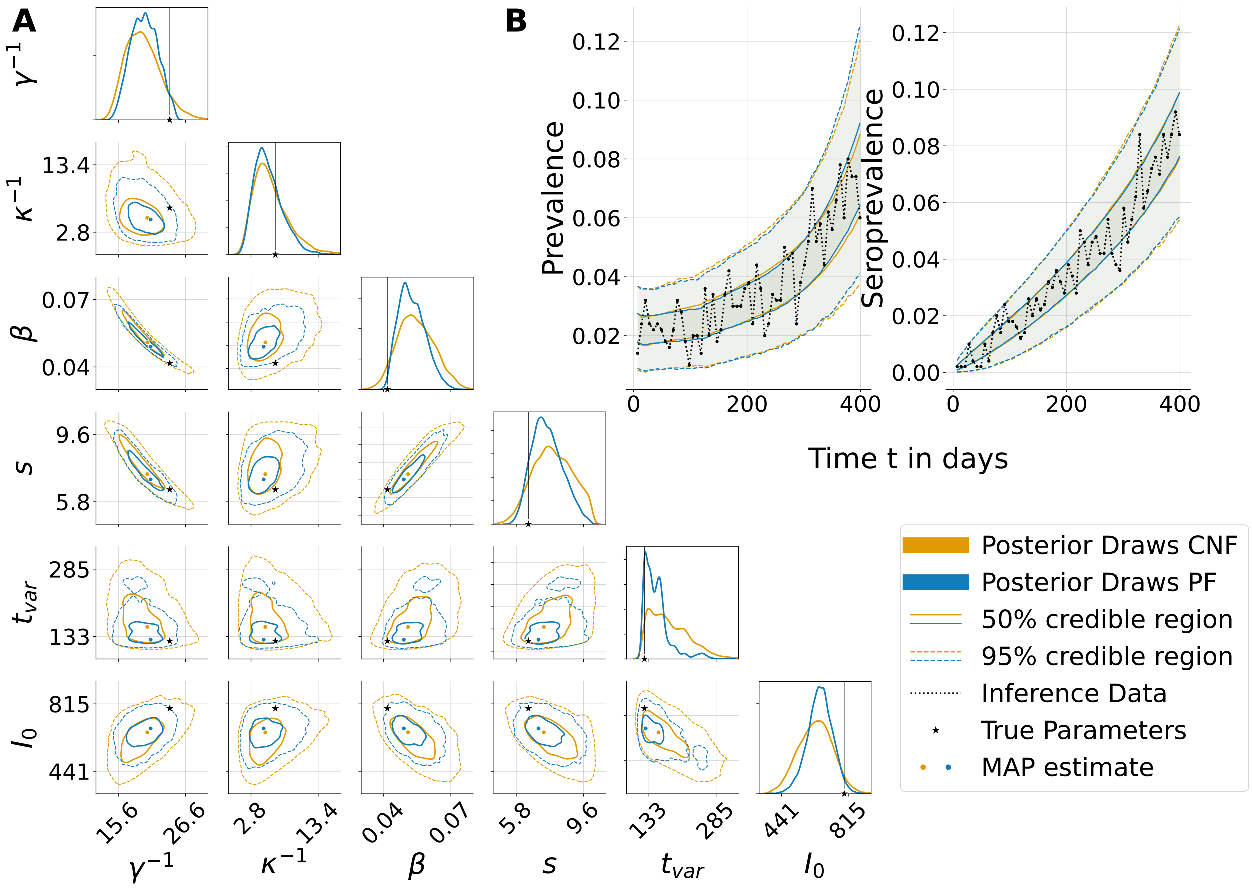

Figure S4.7: **Results of the two-variant SEIR model for *d-5*.**
A Posterior approximations from 10,000 samples. Contour gives the 50% (solid) and 95% (dashed) credible regions, coloured by method. Diagonals show the 1D marginals. Black stars mark the true parameters, coloured circles the joint MAP estimates. **B** Posterior predictive fit: bands give the 50% and 95% pointwise predictive intervals from the same samples (line styles as in **A**) with inference data shown as a dotted line.

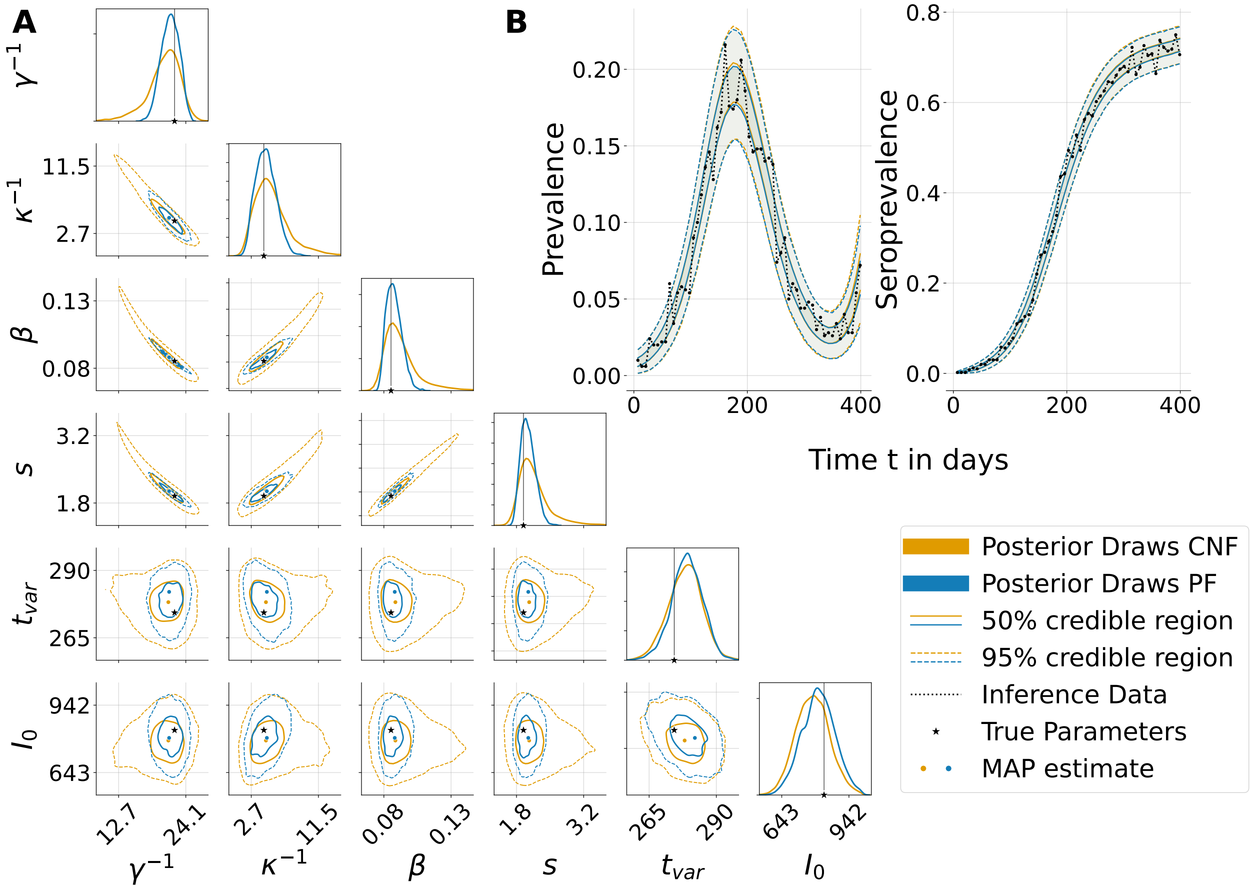

Figure S4.8: **Results of the two-variant SEIR model for *d-6*.**
A Posterior approximations from 10,000 samples. Contour gives the 50% (solid) and 95% (dashed) credible regions, coloured by method. Diagonals show the 1D marginals. Black stars mark the true parameters, coloured circles the joint MAP estimates. **B** Posterior predictive fit: bands give the 50% and 95% pointwise predictive intervals from the same samples (line styles as in **A**) with inference data shown as a dotted line.

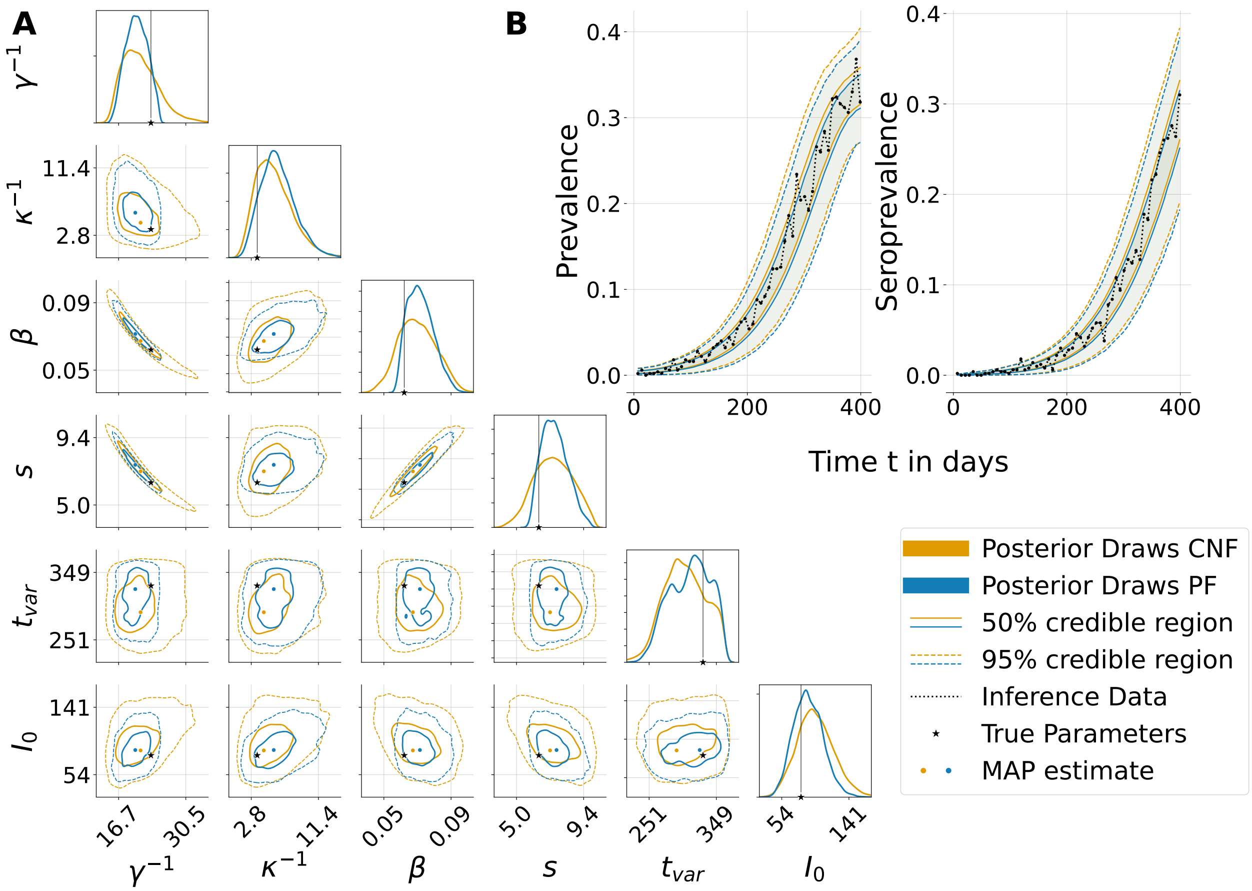

Figure S4.9: **Results of the two-variant SEIR model for d-7.**
A Posterior approximations from 10,000 samples. Contour gives the 50% (solid) and 95% (dashed) credible regions, coloured by method. Diagonals show the 1D marginals. Black stars mark the true parameters, coloured circles the joint MAP estimates. **B** Posterior predictive fit: bands give the 50% and 95% pointwise predictive intervals from the same samples (line styles as in **A**) with inference data shown as a dotted line.

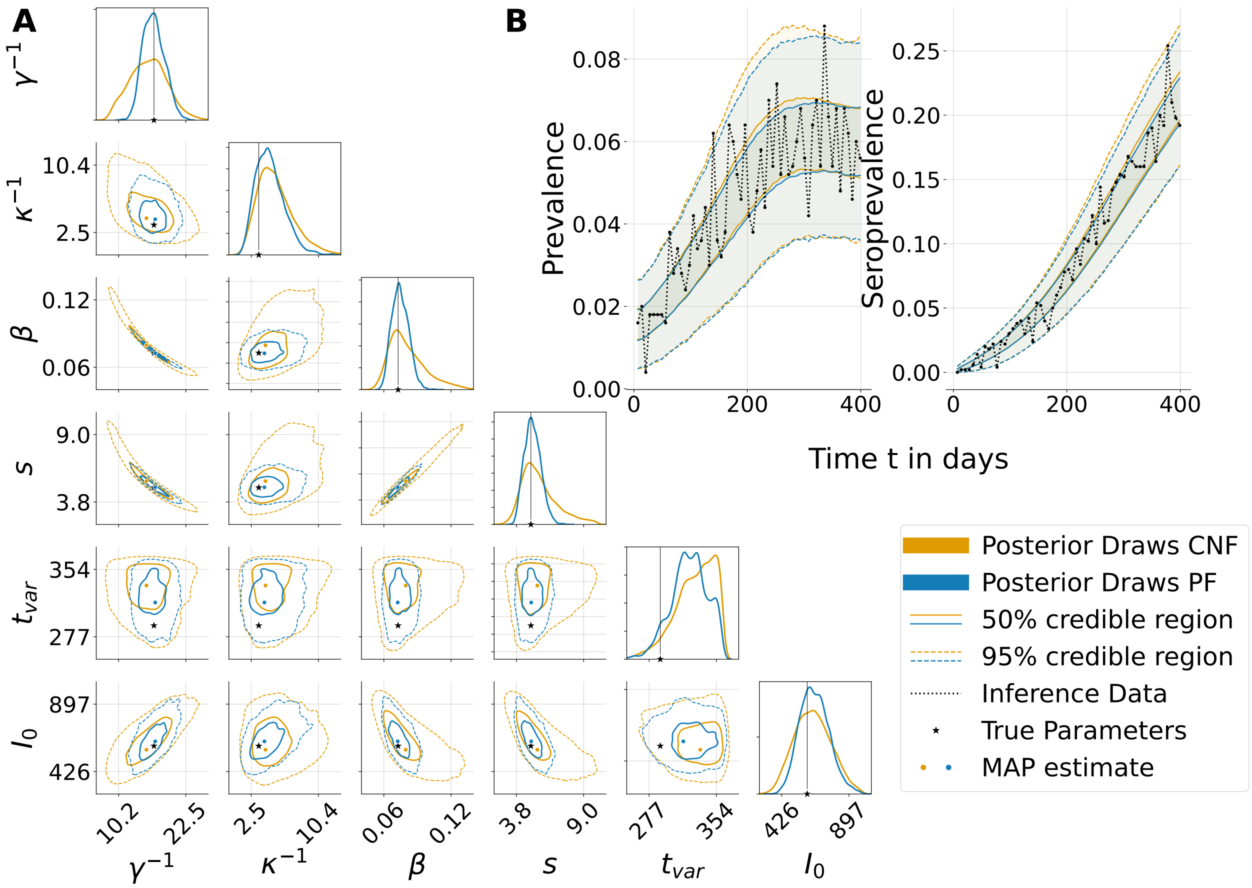

Figure S4.10: **Results of the two-variant SEIR model for *d-8*.**

A Posterior approximations from 10,000 samples. Contour gives the 50% (solid) and 95% (dashed) credible regions, coloured by method. Diagonals show the 1D marginals. Black stars mark the true parameters, coloured circles the joint MAP estimates. **B** Posterior predictive fit: bands give the 50% and 95% pointwise predictive intervals from the same samples (line styles as in **A**) with inference data shown as a dotted line.

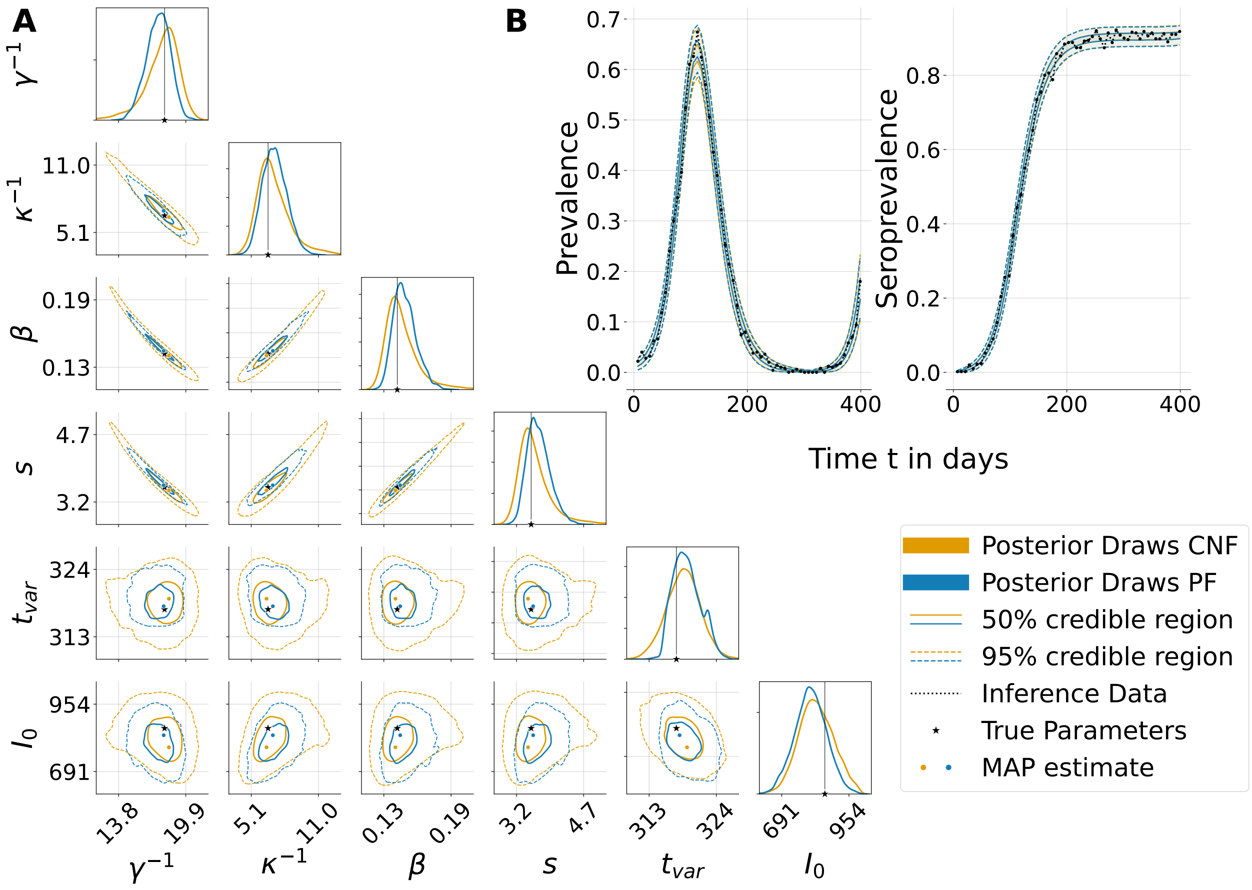

Figure S4.11: **Results of the two-variant SEIR model for *d-9*.**

A Posterior approximations from 10,000 samples. Contour gives the 50% (solid) and 95% (dashed) credible regions, coloured by method. Diagonals show the 1D marginals. Black stars mark the true parameters, coloured circles the joint MAP estimates. **B** Posterior predictive fit: bands give the 50% and 95% pointwise predictive intervals from the same samples (line styles as in **A**) with inference data shown as a dotted line.

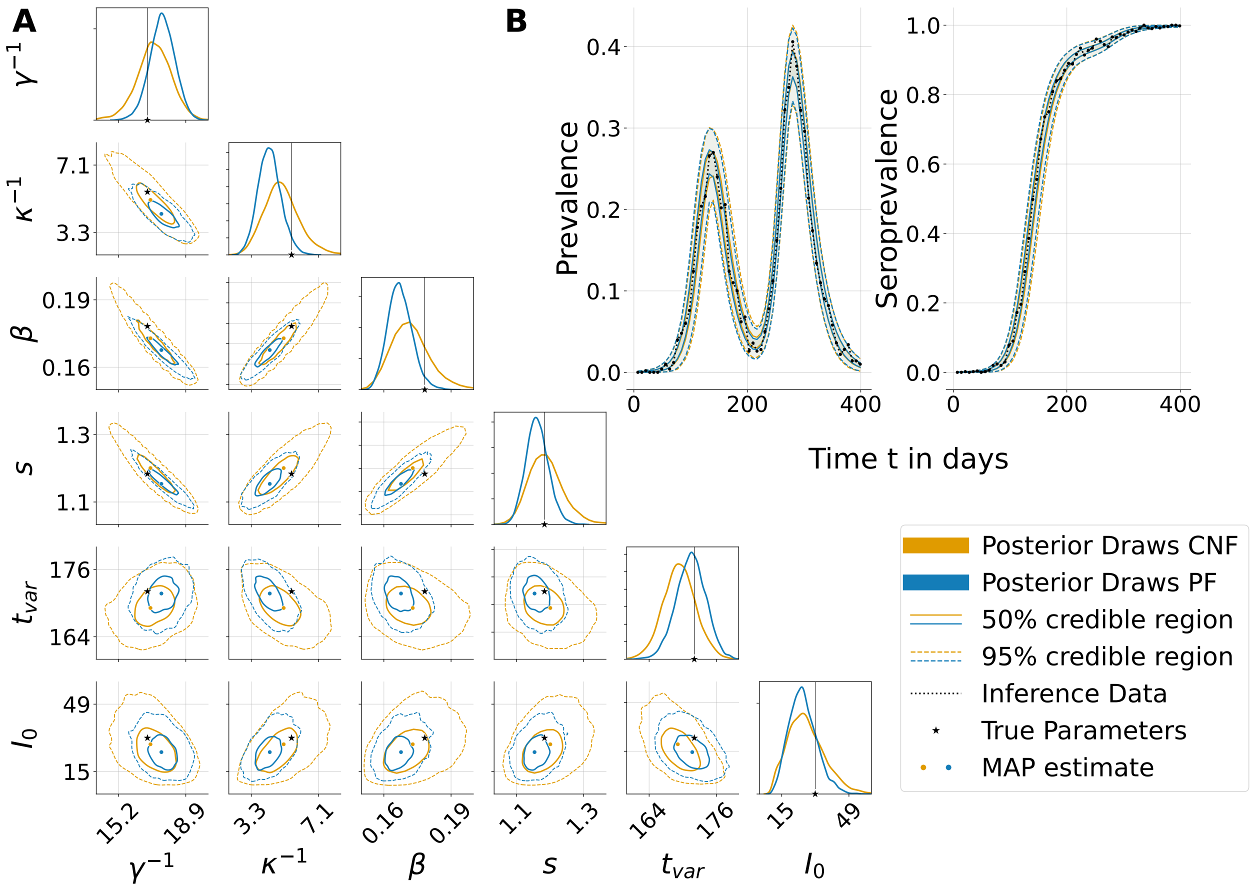

Figure S4.12: **Results of the two-variant SEIR model for *d-10*.**
A Posterior approximations from 10,000 samples. Contour gives the 50% (solid) and 95% (dashed) credible regions, coloured by method. Diagonals show the 1D marginals. Black stars mark the true parameters, coloured circles the joint MAP estimates. **B** Posterior predictive fit: bands give the 50% and 95% pointwise predictive intervals from the same samples (line styles as in **A**) with inference data shown as a dotted line.

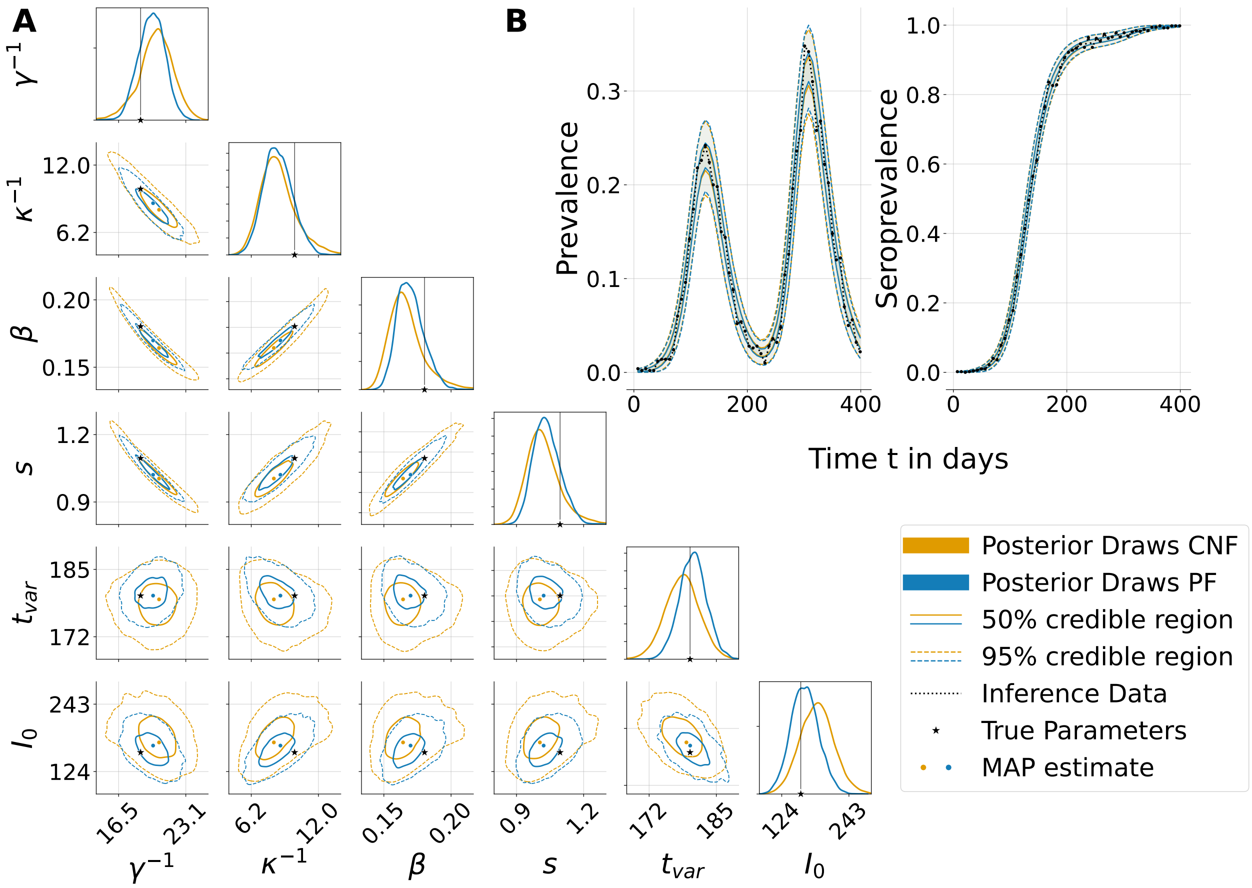

Figure S4.13: **Results of the two-variant SEIR model for *d-11*.**

A Posterior approximations from 10,000 samples. Contour gives the 50% (solid) and 95% (dashed) credible regions, coloured by method. Diagonals show the 1D marginals. Black stars mark the true parameters, coloured circles the joint MAP estimates. **B** Posterior predictive fit: bands give the 50% and 95% pointwise predictive intervals from the same samples (line styles as in **A**) with inference data shown as a dotted line.

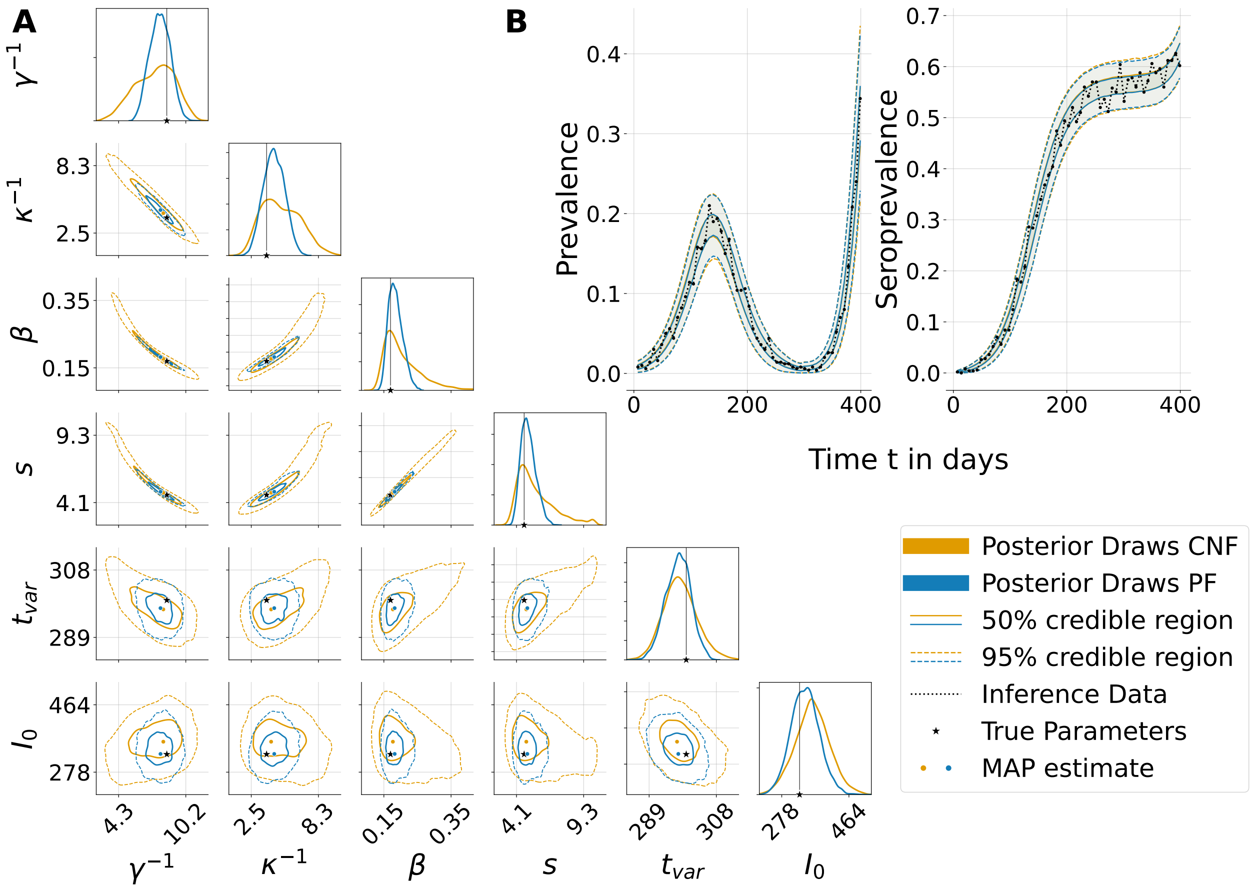

Figure S4.14: **Results of the two-variant SEIR model for *d-12*.**
A Posterior approximations from 10,000 samples. Contour gives the 50% (solid) and 95% (dashed) credible regions, coloured by method. Diagonals show the 1D marginals. Black stars mark the true parameters, coloured circles the joint MAP estimates. **B** Posterior predictive fit: bands give the 50% and 95% pointwise predictive intervals from the same samples (line styles as in **A**) with inference data shown as a dotted line.

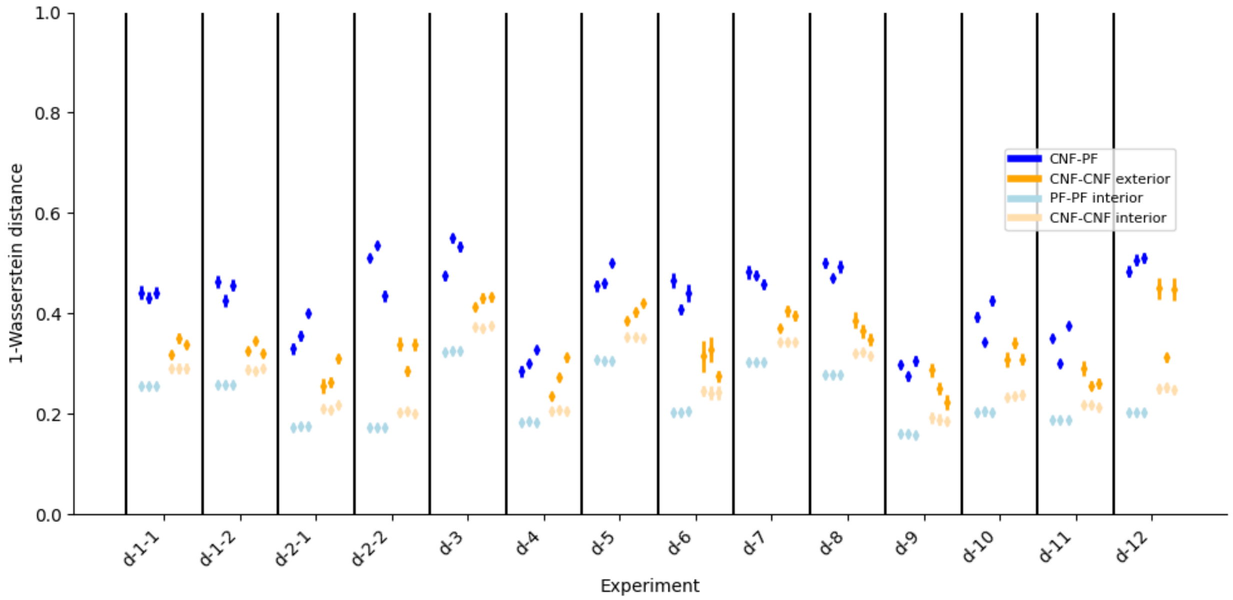

Figure S4.15: **Pairwise 1-Wasserstein distances between PF and CNF with multiple CNF reruns for the two-variant SEIR model** Interior distances quantify within-run Monte-Carlo variability, while exterior distances capture variability across repeated CNF runs.

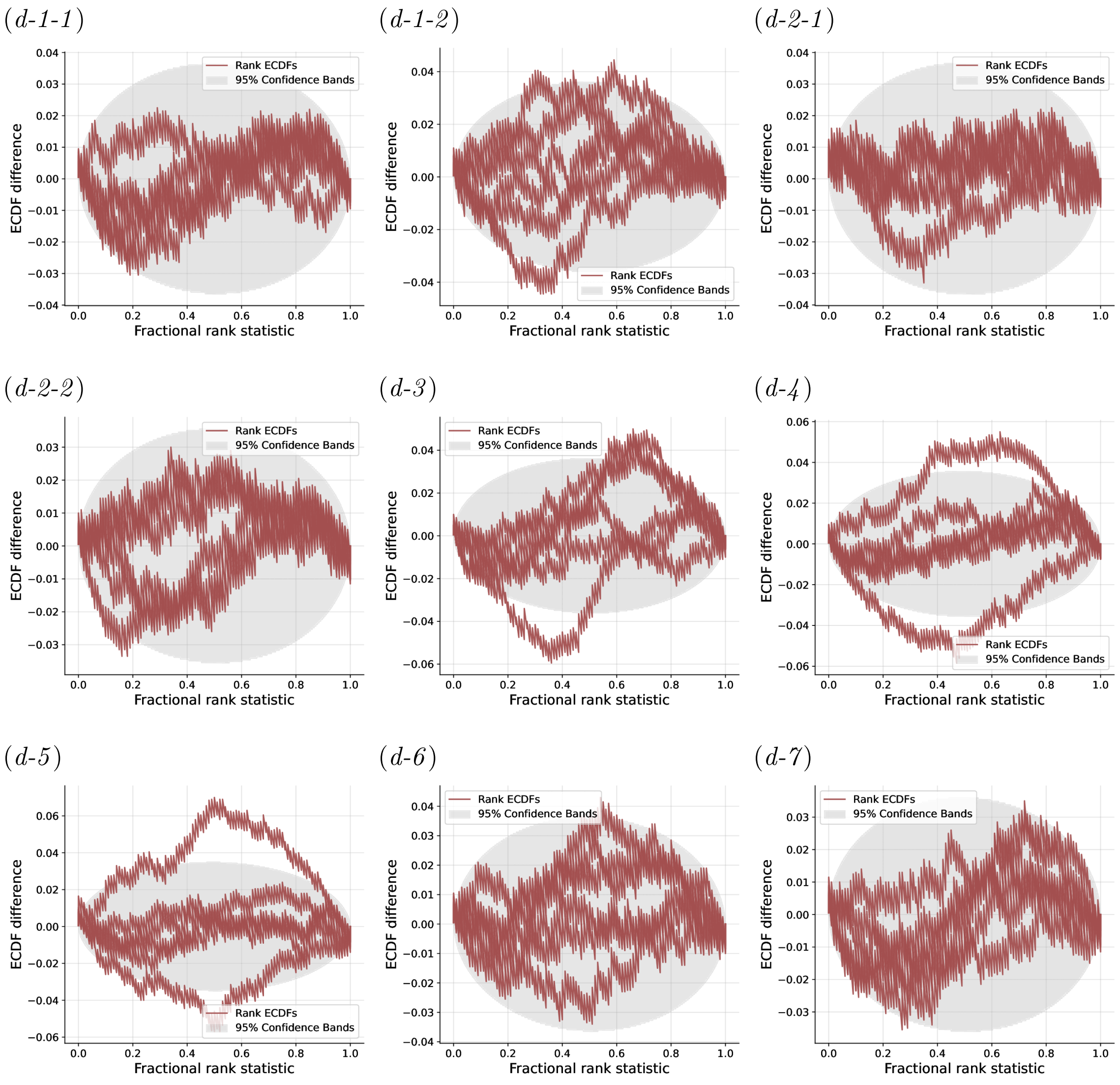
(d-1-1)
(d-1-2)
(d-2-1)
(d-2-2)
(d-3)
(d-4)
(d-5)
(d-6)
(d-7)
Rank ECDFs
95% Confidence Bands
ECDF difference
Fractional rank statistic

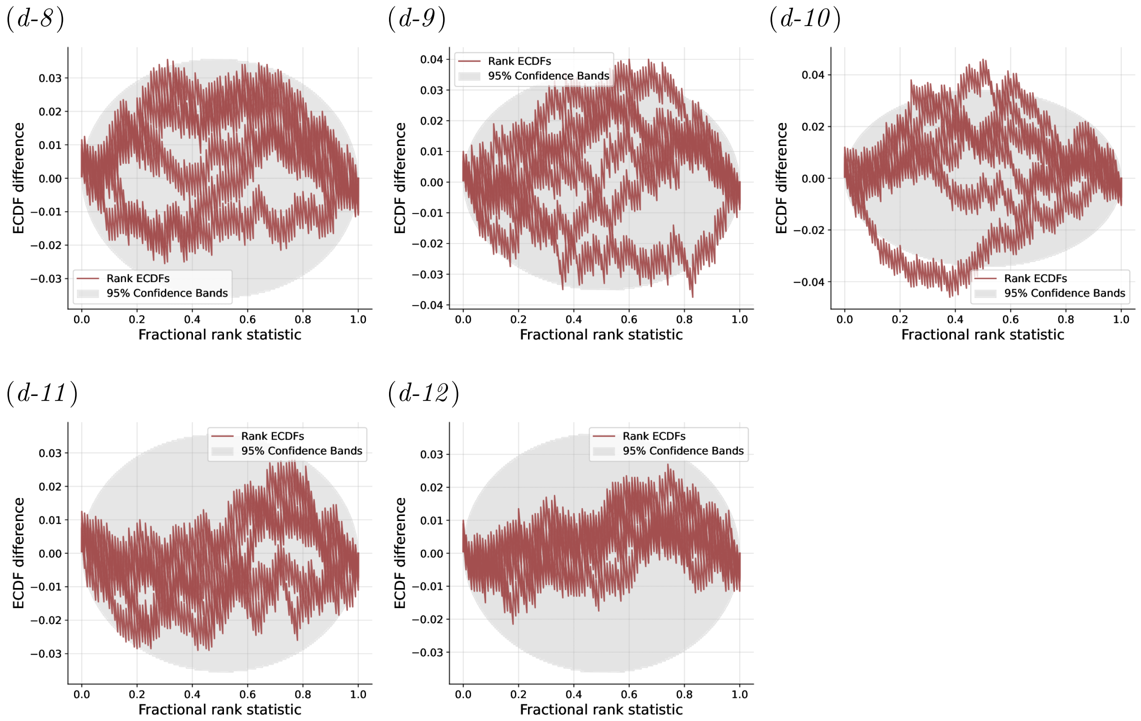

Figure S4.16: **ECDF Calibration plots for the full SEIR model and dense datasets.**

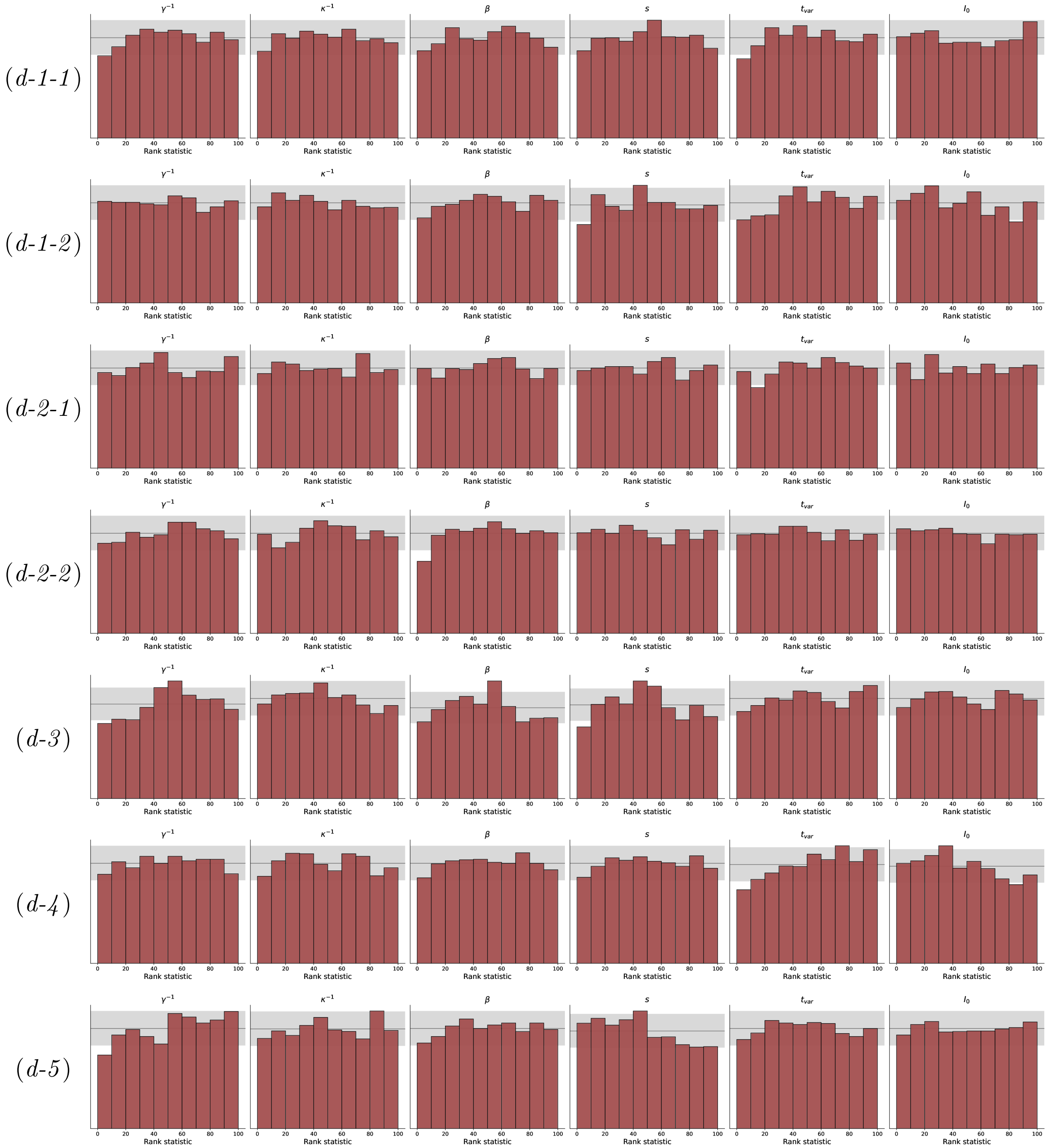

(d-1-1)
(d-1-2)
(d-2-1)
(d-2-2)
(d-3)
(d-4)
(d-5)
γ^{-1}
κ^{-1}
β
s
t_{var}
I_0
Rank statistic
0 20 40 60 80 100

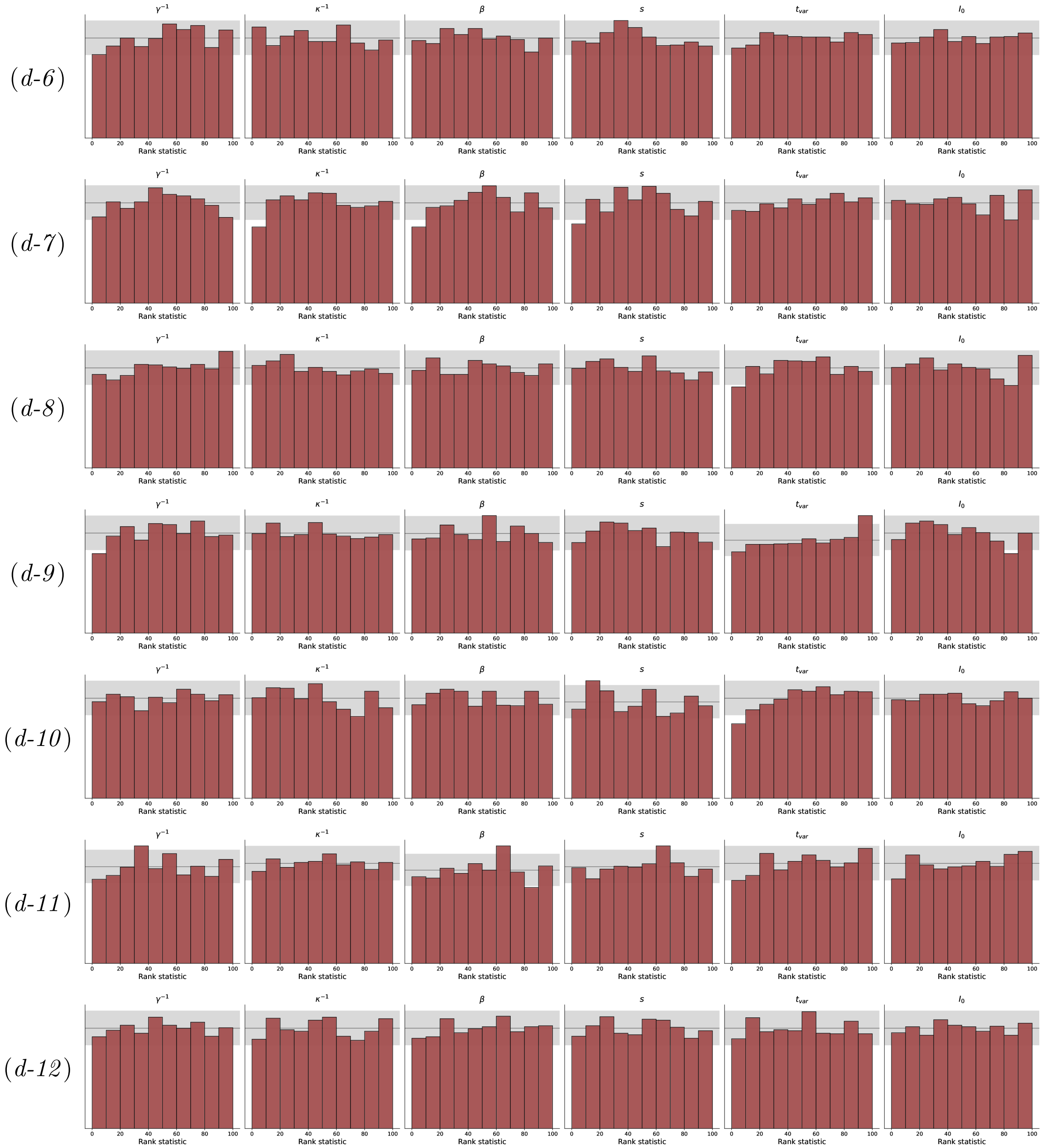

Figure S4.16: **SBC Histograms for the full SEIR model and dense datasets.**

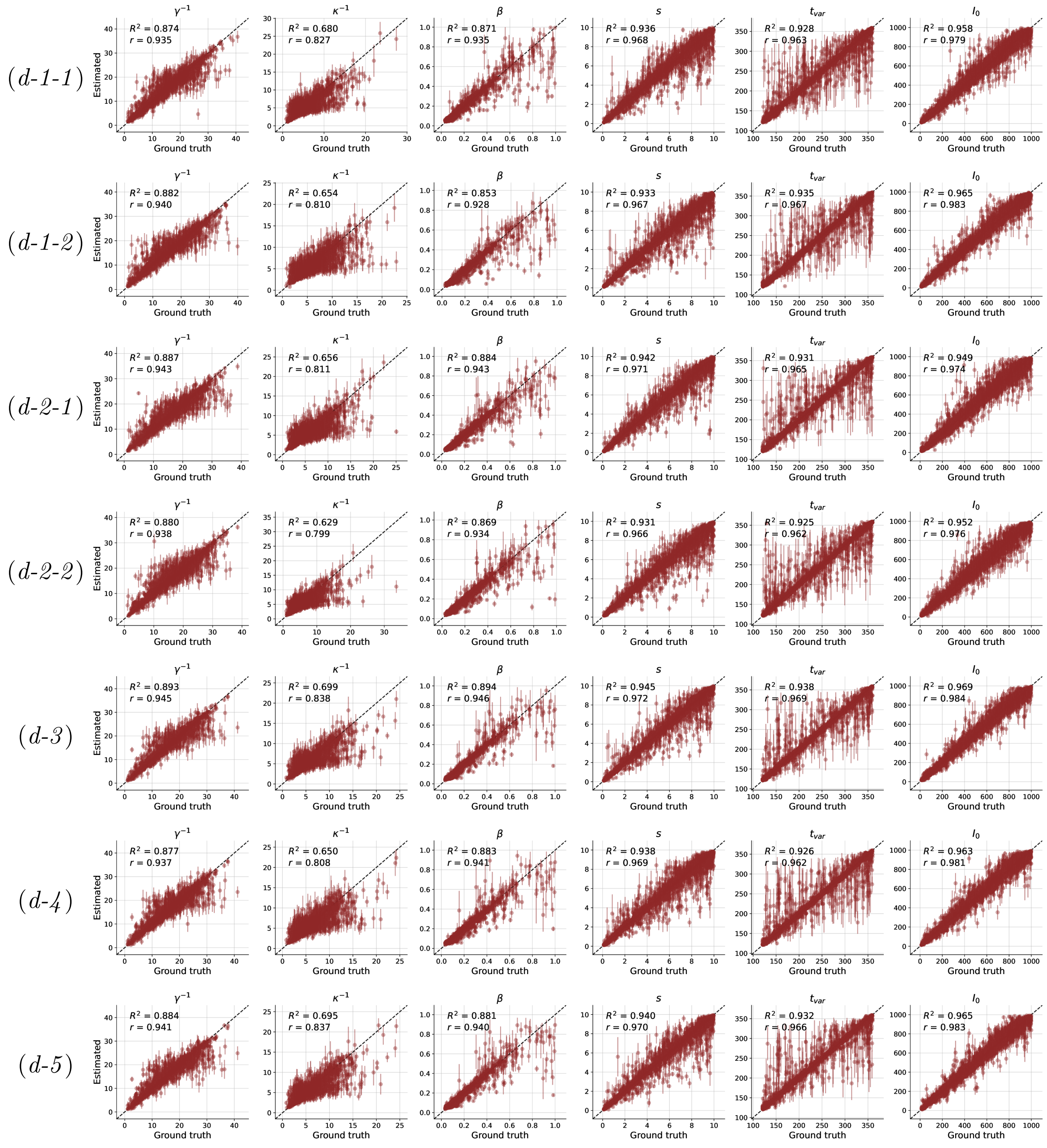
(d-1-1)
γ^{-1} R^2 = 0.874 r = 0.935
κ^{-1} R^2 = 0.680 r = 0.827
β R^2 = 0.871 r = 0.935
s R^2 = 0.936 r = 0.968
t_{var} R^2 = 0.928 r = 0.963
I_0 R^2 = 0.958 r = 0.979
(d-1-2)
γ^{-1} R^2 = 0.882 r = 0.940
κ^{-1} R^2 = 0.654 r = 0.810
β R^2 = 0.853 r = 0.928
s R^2 = 0.933 r = 0.967
t_{var} R^2 = 0.935 r = 0.967
I_0 R^2 = 0.965 r = 0.983
(d-2-1)
γ^{-1} R^2 = 0.887 r = 0.943
κ^{-1} R^2 = 0.656 r = 0.811
β R^2 = 0.884 r = 0.943
s R^2 = 0.942 r = 0.971
t_{var} R^2 = 0.931 r = 0.965
I_0 R^2 = 0.949 r = 0.974
(d-2-2)
γ^{-1} R^2 = 0.880 r = 0.938
κ^{-1} R^2 = 0.629 r = 0.799
β R^2 = 0.869 r = 0.934
s R^2 = 0.931 r = 0.966
t_{var} R^2 = 0.925 r = 0.962
I_0 R^2 = 0.952 r = 0.976
(d-3)
γ^{-1} R^2 = 0.893 r = 0.945
κ^{-1} R^2 = 0.699 r = 0.838
β R^2 = 0.894 r = 0.946
s R^2 = 0.945 r = 0.972
t_{var} R^2 = 0.938 r = 0.969
I_0 R^2 = 0.969 r = 0.984
(d-4)
γ^{-1} R^2 = 0.877 r = 0.937
κ^{-1} R^2 = 0.650 r = 0.808
β R^2 = 0.883 r = 0.941
s R^2 = 0.938 r = 0.969
t_{var} R^2 = 0.926 r = 0.962
I_0 R^2 = 0.963 r = 0.981
(d-5)
γ^{-1} R^2 = 0.884 r = 0.941
κ^{-1} R^2 = 0.695 r = 0.837
β R^2 = 0.881 r = 0.940
s R^2 = 0.940 r = 0.970
t_{var} R^2 = 0.932 r = 0.966
I_0 R^2 = 0.965 r = 0.983
Estimated
Ground truth

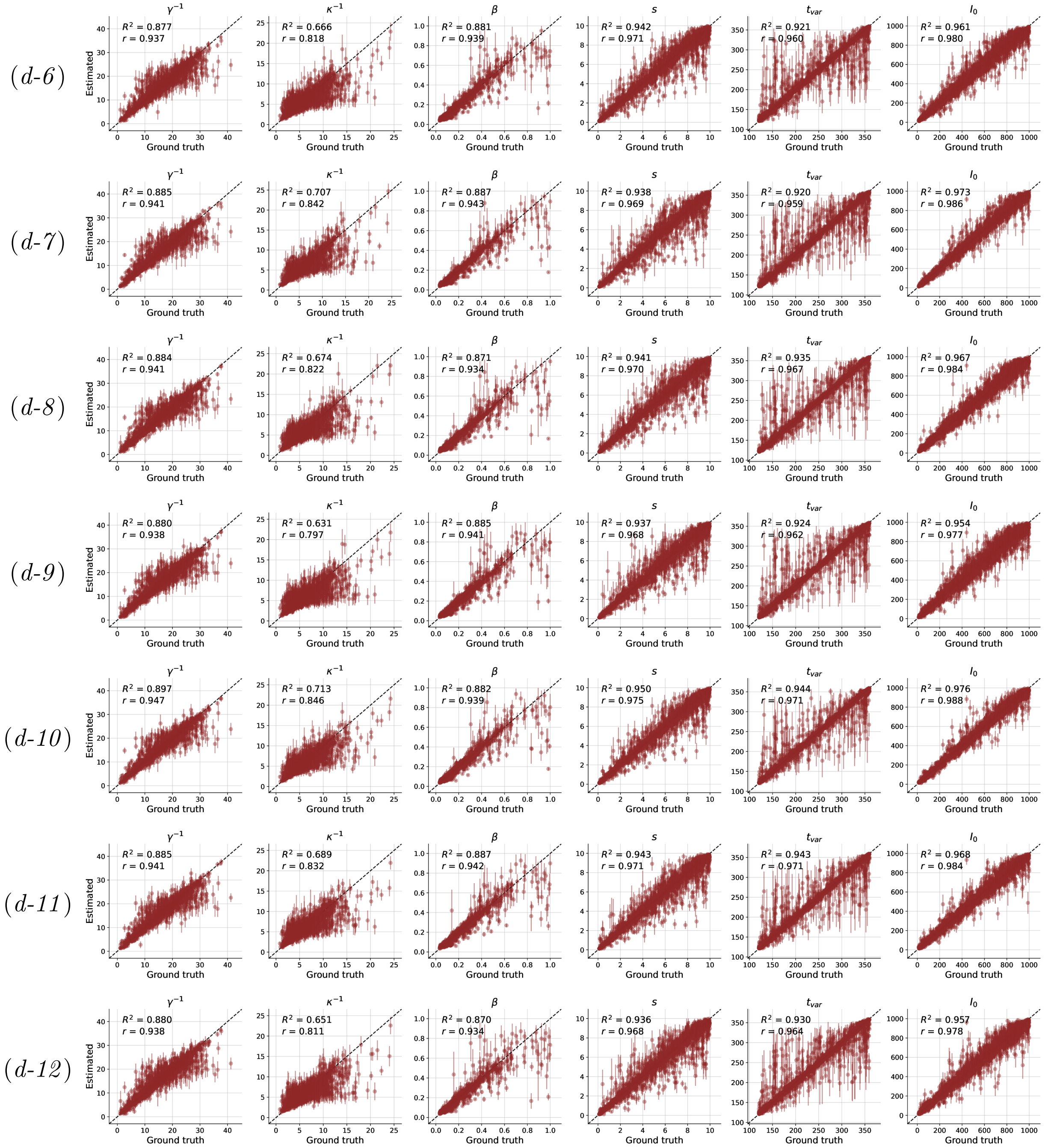

Figure S4.16: **Parameter recovery for the full SEIR model and dense datasets.**

S4.B Supplementary Tables

Table S4.1: Posterior MAP estimates with 95% intervals for datasets *d-1-1* to *d-5*.

Dataset	Method	γ^{-1}	κ^{-1}	β	s	t_{var}	I_0
d-1-1	True	17.00	5.00	0.0800	3.00	150.0	500.0
	CNF	15.47 (8.83, 19.26)	4.93 (2.42, 13.54)	0.0867 (0.07, 0.15)	3.29 (2.6, 5.8)	160.6 (128.5, 180.8)	446.3 (370.1, 598.2)
	PF	15.62 (12.65, 18.29)	5.04 (2.45, 9.18)	0.0855 (0.07, 0.11)	3.22 (2.8, 4.0)	153.0 (132.8, 175.5)	479.2 (371.7, 581.0)
d-1-2	True	17.00	5.00	0.0800	3.00	150.0	500.0
	CNF	15.00 (9.86, 19.00)	4.95 (2.24, 11.77)	0.0902 (0.07, 0.14)	3.28 (2.6, 5.1)	161.7 (128.3, 180.7)	374.7 (291.7, 519.0)
	PF	15.04 (12.10, 17.34)	4.26 (2.22, 8.39)	0.0890 (0.08, 0.11)	3.25 (2.8, 4.0)	165.6 (141.1, 183.6)	366.6 (285.9, 470.9)
d-2-1	True	11.70	8.40	0.2300	1.60	222.0	560.0
	CNF	12.39 (7.46, 14.56)	7.13 (5.11, 12.92)	0.2162 (0.18, 0.36)	1.53 (1.3, 2.5)	223.9 (219.2, 231.1)	511.8 (407.6, 659.3)
	PF	11.55 (9.57, 13.19)	7.91 (6.04, 10.09)	0.2313 (0.20, 0.28)	1.61 (1.4, 1.9)	224.4 (220.5, 228.3)	509.0 (412.9, 603.8)
d-2-2	True	11.70	8.40	0.2300	1.60	222.0	560.0
	CNF	15.32 (12.73, 17.03)	5.06 (3.32, 7.74)	0.1774 (0.16, 0.21)	1.26 (1.1, 1.5)	222.8 (217.1, 228.8)	541.3 (416.0, 706.4)
	PF	13.97 (12.37, 15.85)	6.26 (4.43, 7.97)	0.1947 (0.17, 0.22)	1.34 (1.2, 1.5)	221.3 (217.7, 226.0)	592.1 (454.0, 684.7)
d-3	True	15.47	3.97	0.0655	8.40	226.0	477.3
	CNF	20.72 (13.99, 28.29)	6.52 (2.07, 14.34)	0.0501 (0.04, 0.07)	6.20 (4.7, 9.6)	158.4 (123.8, 319.3)	672.8 (401.5, 843.7)
	PF	16.75 (13.80, 22.70)	4.89 (2.32, 12.81)	0.0612 (0.05, 0.07)	7.92 (5.8, 9.7)	222.7 (129.6, 287.9)	542.8 (421.3, 741.6)
d-4	True	11.57	6.11	0.1605	8.34	318.0	169.6
	CNF	12.81 (10.06, 14.68)	5.10 (3.31, 7.73)	0.1451 (0.13, 0.19)	7.58 (6.6, 9.7)	321.6 (314.9, 327.1)	189.7 (142.7, 236.4)
	PF	13.20 (10.76, 14.31)	4.69 (3.55, 7.06)	0.1406 (0.13, 0.17)	7.35 (6.7, 9.0)	320.9 (314.9, 325.5)	163.3 (133.0, 219.4)
d-5	True	24.05	6.63	0.0423	6.45	122.0	789.9
	CNF	20.37 (15.04, 26.37)	5.06 (2.10, 13.28)	0.0512 (0.04, 0.07)	7.33 (5.8, 9.9)	154.6 (122.3, 282.7)	657.2 (446.9, 811.7)
	PF	20.91 (15.89, 24.17)	4.80 (2.18, 10.68)	0.0494 (0.04, 0.06)	7.03 (6.1, 9.3)	125.7 (120.7, 246.2)	678.9 (509.4, 786.1)

Table S4.2: Posterior MAP estimates with 95% intervals for datasets *d-6* to *d-12*.

Dataset	Method	γ^{-1}	κ^{-1}	β	s	t_{var}	I_0
d-6	True	22.21	4.37	0.0807	1.91	274.0	831.2
	CNF	21.15 (13.34, 24.72)	4.64 (2.22, 11.87)	0.0841 (0.07, 0.13)	2.03 (1.7, 3.2)	277.9 (265.6, 289.9)	785.1 (638.1, 929.8)
	PF	21.28 (18.43, 24.14)	4.76 (2.42, 7.40)	0.0836 (0.07, 0.10)	2.02 (1.8, 2.3)	281.7 (266.9, 289.5)	796.6 (660.6, 948.5)
d-7	True	23.34	3.52	0.0631	6.43	329.0	79.0
	CNF	21.21 (15.72, 30.12)	4.37 (2.11, 11.52)	0.0679 (0.05, 0.09)	7.17 (5.0, 9.7)	291.2 (246.0, 357.0)	85.4 (54.4, 148.1)
	PF	20.12 (16.35, 24.43)	5.65 (2.55, 11.31)	0.0718 (0.06, 0.09)	7.60 (6.1, 9.3)	324.9 (255.7, 356.9)	85.9 (53.8, 128.5)
d-8	True	16.66	3.35	0.0699	4.95	289.0	604.8
	CNF	15.28 (9.43, 22.57)	4.14 (2.08, 10.52)	0.0774 (0.05, 0.12)	5.44 (3.6, 9.1)	335.3 (275.8, 359.2)	578.8 (412.6, 900.3)
	PF	16.89 (13.15, 20.59)	4.02 (2.04, 8.39)	0.0696 (0.06, 0.09)	4.97 (4.0, 6.4)	316.1 (278.3, 357.1)	637.3 (473.0, 874.3)
d-9	True	17.98	6.59	0.1430	3.55	317.0	859.7
	CNF	18.38 (13.45, 20.16)	6.46 (4.92, 11.30)	0.1415 (0.13, 0.19)	3.49 (3.2, 4.8)	318.9 (312.8, 323.8)	785.7 (696.0, 960.2)
	PF	17.90 (15.18, 19.36)	7.01 (5.29, 9.31)	0.1454 (0.13, 0.17)	3.60 (3.3, 4.3)	317.7 (315.3, 323.5)	832.8 (680.1, 924.6)
d-10	True	16.77	5.57	0.1786	1.18	172.0	31.8
	CNF	16.94 (15.03, 18.97)	5.14 (3.30, 7.31)	0.1727 (0.16, 0.20)	1.20 (1.1, 1.4)	169.4 (163.8, 175.6)	28.6 (11.8, 48.3)
	PF	17.55 (16.18, 18.95)	4.36 (3.17, 5.69)	0.1669 (0.16, 0.18)	1.13 (1.1, 1.2)	172.0 (166.4, 176.9)	24.6 (13.5, 41.3)
d-11	True	18.63	9.93	0.1807	1.07	180.0	157.8
	CNF	20.45 (16.25, 23.27)	8.16 (6.05, 12.37)	0.1640 (0.15, 0.20)	0.97 (0.9, 1.2)	179.3 (171.8, 185.1)	175.4 (129.6, 246.2)
	PF	19.86 (17.22, 22.15)	8.72 (6.24, 11.05)	0.1698 (0.15, 0.19)	0.99 (0.9, 1.1)	180.1 (175.3, 185.8)	169.8 (118.9, 214.4)
d-12	True	8.51	3.80	0.1731	4.66	299.0	327.2
	CNF	8.21 (4.09, 10.39)	4.20 (2.32, 8.54)	0.1802 (0.14, 0.36)	4.83 (3.8, 9.4)	297.2 (289.2, 308.5)	362.0 (267.8, 458.2)
	PF	7.97 (6.28, 9.60)	4.47 (2.72, 6.22)	0.1855 (0.15, 0.23)	4.91 (4.0, 6.2)	297.5 (290.2, 304.3)	328.2 (268.0, 421.9)

Table S4.3: **Effective sample sizes (ESS) per parameter for the full SEIR2V model with dense datasets.** ESS computed on the last 10,000 samples of the chains resulting from running the PF method on the two-variant SEIR model and using a maximum lag size of 250 for the autocorrelation.

**Dataset**	$\boldsymbol{\gamma^{-1}}$	$\boldsymbol{\kappa^{-1}}$	$\boldsymbol{\beta}$	**s**	$\mathbf{t}_{\text{var}}$	$\mathbf{I_0}$
d-1-1	501.6	658.8	514.8	528.8	1174.2	1537.4
d-1-2	436.2	539.8	401.8	428.6	1493.4	1768.6
d-2-1	445.3	459.9	440.9	457.8	1676.1	1842.0
d-2-2	1549.2	1633.8	1608.7	1652.8	1954.5	1687.8
d-3	660.1	1395.9	680.7	1544.2	703.5	1068.0
d-4	789.4	843.0	765.5	2166.4	773.3	1933.8
d-5	967.9	2134.2	869.9	958.7	1055.1	1190.6
d-6	1034.9	1213.3	994.5	2822.5	1082.6	2566.2
d-7	482.4	1727.4	496.0	1583.2	488.4	1670.8
d-8	513.7	1372.9	488.5	2116.0	533.3	931.4
d-9	1737.7	1764.2	1721.3	2198.1	1709.5	2796.7
d-10	3044.8	3274.7	3089.5	2723.8	3038.7	3166.4
d-11	2092.2	2129.0	2070.7	2892.3	1952.4	2572.2
d-12	323.8	365.7	322.3	1399.6	331.8	1879.7

Table S4.4: $\hat{\mathbf{R}}$ **diagnostics for the SEIR model with dense data.**

**Dataset**	$\boldsymbol{\gamma^{-1}}$	$\boldsymbol{\kappa^{-1}}$	$\boldsymbol{\beta}$	$\mathbf{s}$	$\mathbf{t}_{\mathrm{var}}$	$\mathbf{I_0}$
d-1-1	1.023	1.010	1.022	1.022	1.007	1.005
d-1-2	1.025	1.014	1.024	1.021	1.004	1.002
d-2-1	1.015	1.012	1.008	1.022	1.007	1.009
d-2-2	1.014	1.009	1.008	1.022	1.007	1.004
d-3	1.043	1.008	1.043	1.008	1.039	1.017
d-4	1.019	1.019	1.019	1.003	1.017	1.007
d-5	1.004	1.002	1.005	1.008	1.005	1.005
d-6	1.025	1.020	1.024	1.005	1.021	1.003
d-7	1.062	1.009	1.059	1.004	1.063	1.008
d-8	1.054	1.011	1.054	1.004	1.052	1.020
d-9	1.009	1.010	1.009	1.009	1.009	1.004
d-10	1.002	1.001	1.003	1.002	1.003	1.002
d-11	1.010	1.009	1.009	1.005	1.011	1.006
d-12	1.125	1.108	1.125	1.009	1.120	1.005
