# Supplementary material for: Assessment of simulation-based inference methods for stochastic compartmental models in epidemiological research
Source: PLoS One. 2026 Jul 13;21(7):e0353306. doi: 10.1371/journal.pone.0353306 (PMC13362117; doi:10.1371/journal.pone.0353306)
Supplement: S5 Result — (PDF) [file pone.0353306.s005.pdf]

S5 Supplementary Results SEIR-Model with Sparse Data
Assessment of Simulation-based Inference Methods for Stochastic Compartmental Models in Epidemiological Research

Vincent Wieland[1,2,◕], Nils Waßmuth[1,2,3,◕], Lorenzo Contento[1], Martin Kühn[1,2,3], and Jan Hasenauer[1,2,*]

[1]Bonn Center for Mathematical Life Sciences, University of Bonn, Bonn, Germany
[2]Life and Medical Science Institute, University of Bonn, Bonn, Germany
[3]Institute of Software Technology, Department for High-Performance Computing, German Aerospace Center (DLR), Cologne, Germany
[◕]These authors contributed equally to the work.
[*]To whom correspondence should be addressed; jan.hasenauer@uni-bonn.de.

June 26, 2026

Contents

S5.A Supplementary Figures 2
S5.B Supplementary Tables 24

S5.A Supplementary Figures

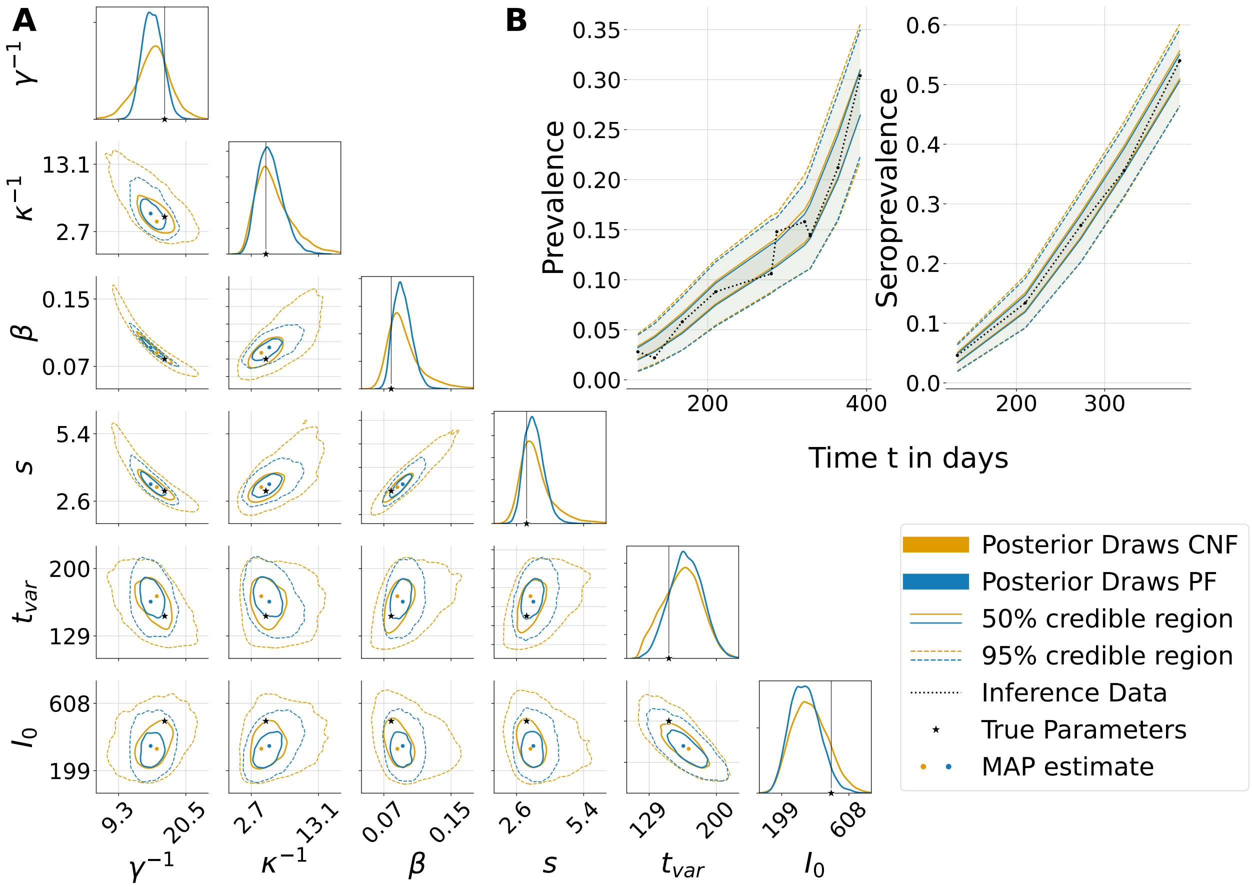

Figure S5.1: **Results of the two-variant SEIR model for *s-1-1-1*.**
A Posterior approximations from 10,000 samples. Contour gives the 50% (solid) and 95% (dashed) credible regions, coloured by method. Diagonals show the 1D marginals. Black stars mark the true parameters, coloured circles the joint MAP estimates. **B** Posterior predictive fit: bands give the 50% and 95% pointwise predictive intervals from the same samples (line styles as in **A**) with inference data shown as a dotted line.

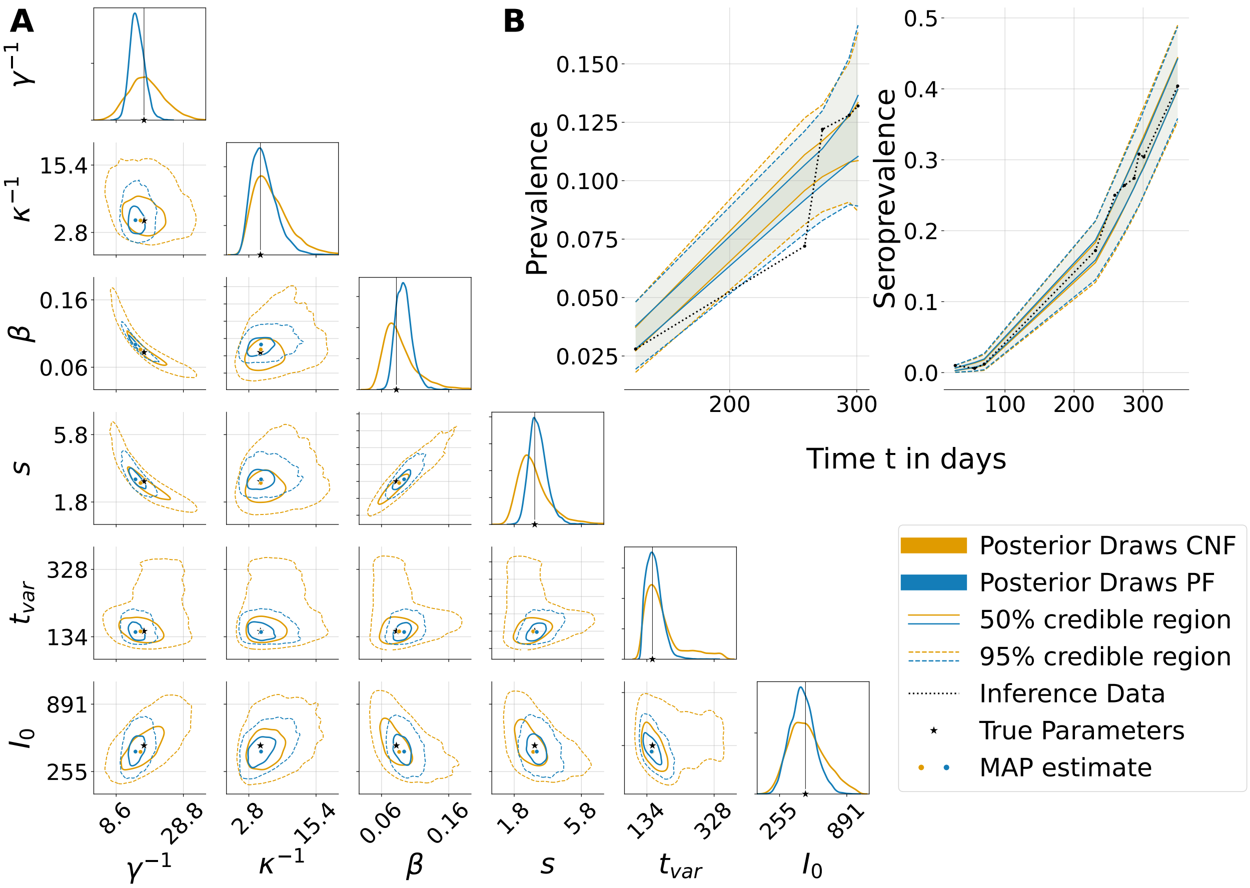

Figure S5.2: **Results of the two-variant SEIR model for *s-1-1-2*.**
A Posterior approximations from 10,000 samples. Contour gives the 50% (solid) and 95% (dashed) credible regions, coloured by method. Diagonals show the 1D marginals. Black stars mark the true parameters, coloured circles the joint MAP estimates. **B** Posterior predictive fit: bands give the 50% and 95% pointwise predictive intervals from the same samples (line styles as in **A**) with inference data shown as a dotted line.

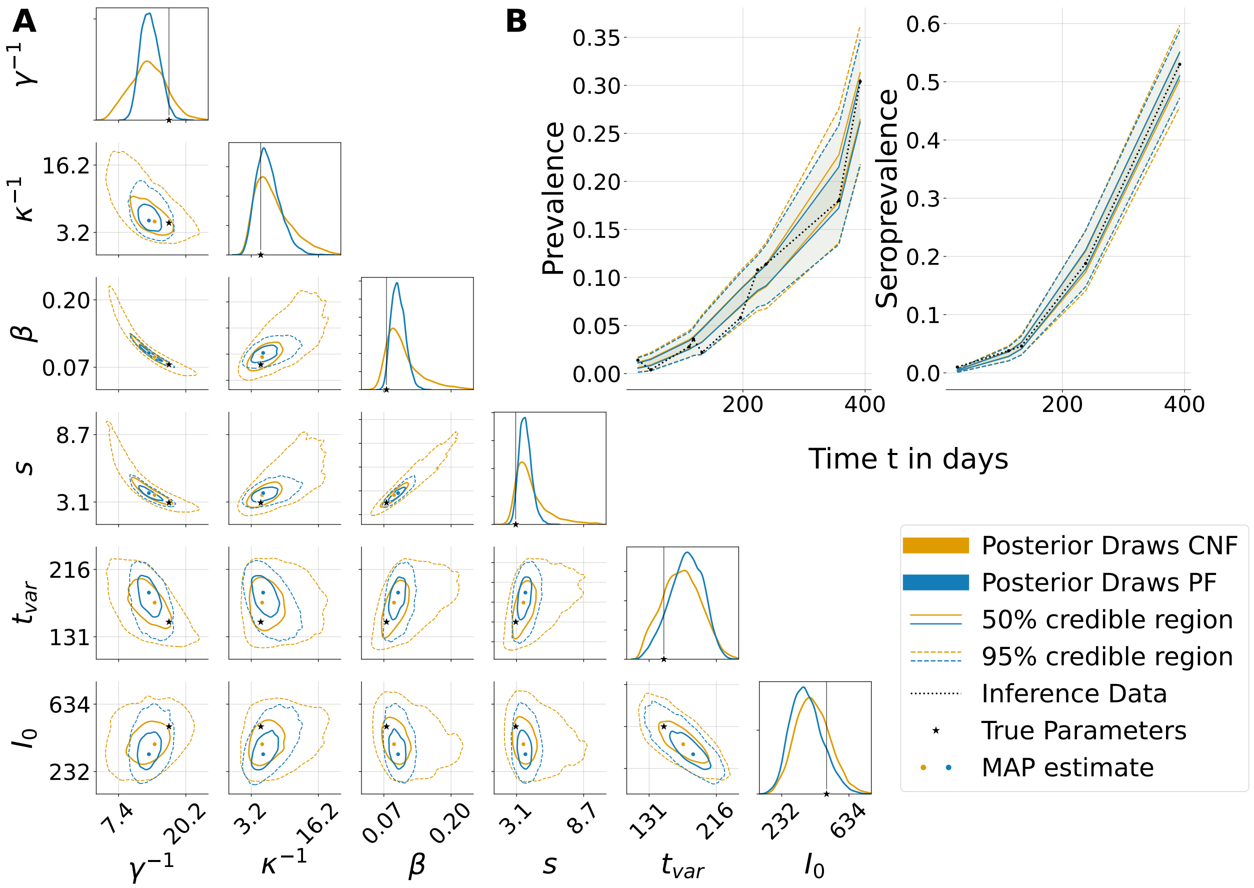

Figure S5.3: **Results of the two-variant SEIR model for *s-1-1-3*.**
A Posterior approximations from 10,000 samples. Contour gives the 50% (solid) and 95% (dashed) credible regions, coloured by method. Diagonals show the 1D marginals. Black stars mark the true parameters, coloured circles the joint MAP estimates. **B** Posterior predictive fit: bands give the 50% and 95% pointwise predictive intervals from the same samples (line styles as in **A**) with inference data shown as a dotted line.

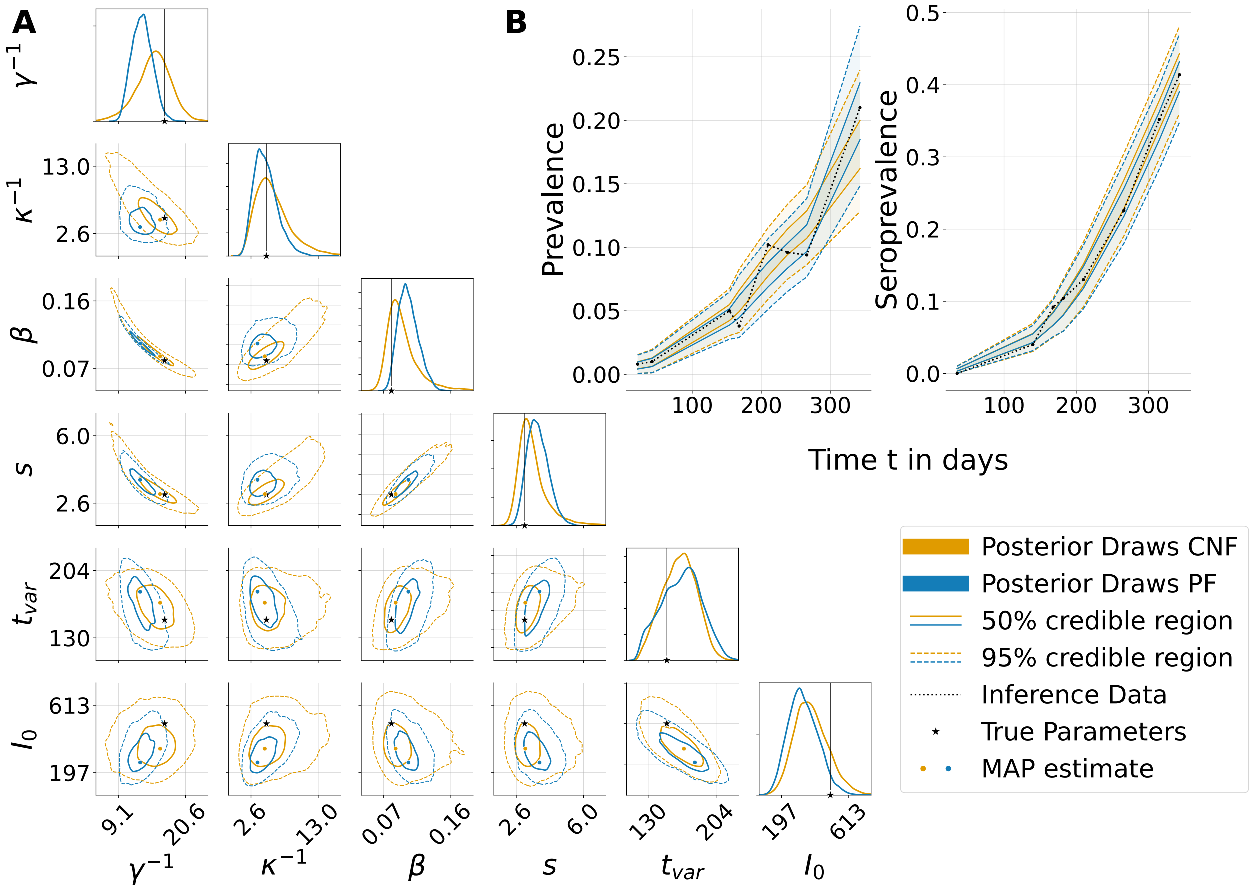

Figure S5.4: **Results of the two-variant SEIR model for *s-1-2-1*.**
A Posterior approximations from 10,000 samples. Contour gives the 50% (solid) and 95% (dashed) credible regions, coloured by method. Diagonals show the 1D marginals. Black stars mark the true parameters, coloured circles the joint MAP estimates. **B** Posterior predictive fit: bands give the 50% and 95% pointwise predictive intervals from the same samples (line styles as in **A**) with inference data shown as a dotted line.

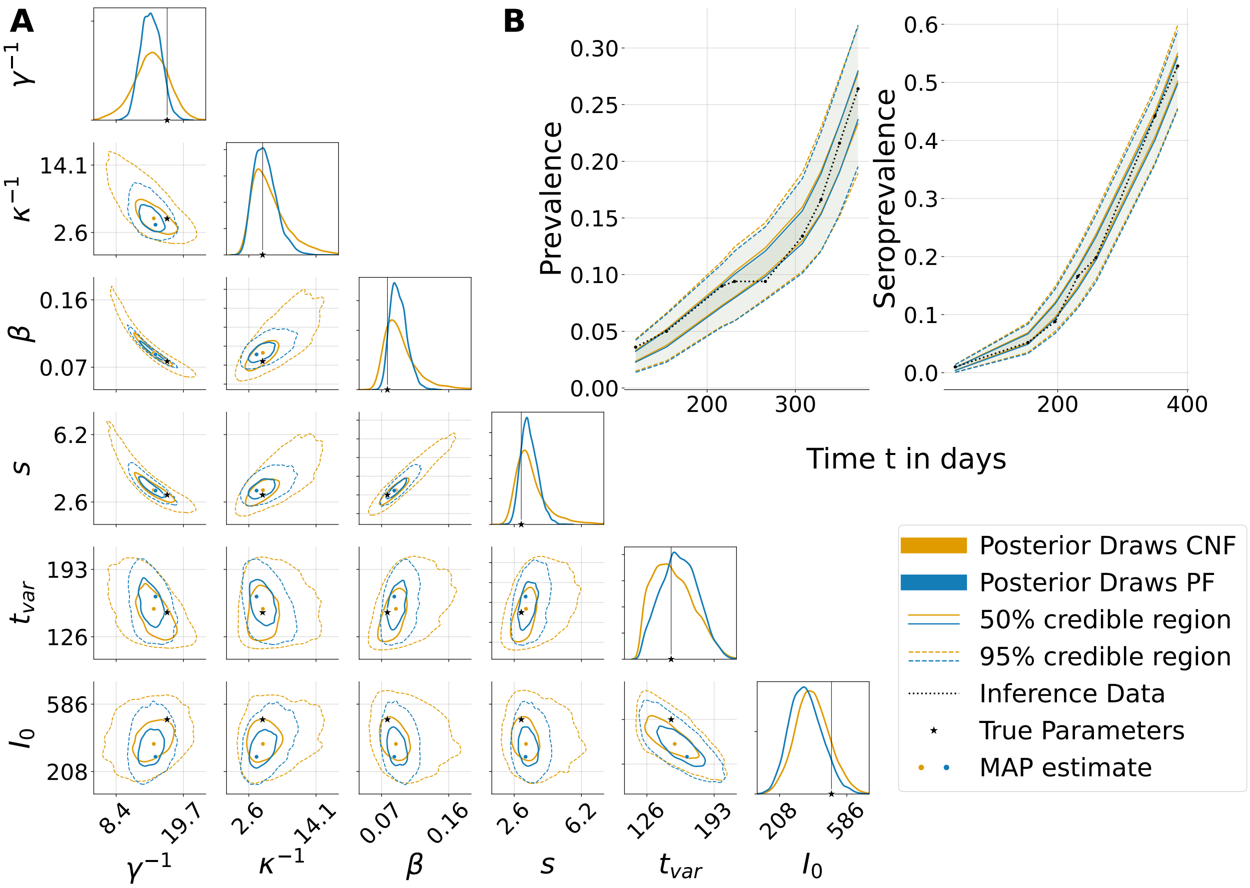

Figure S5.5: **Results of the two-variant SEIR model for *s-1-2-2*.**
A Posterior approximations from 10,000 samples. Contour gives the 50% (solid) and 95% (dashed) credible regions, coloured by method. Diagonals show the 1D marginals. Black stars mark the true parameters, coloured circles the joint MAP estimates. **B** Posterior predictive fit: bands give the 50% and 95% pointwise predictive intervals from the same samples (line styles as in **A**) with inference data shown as a dotted line.

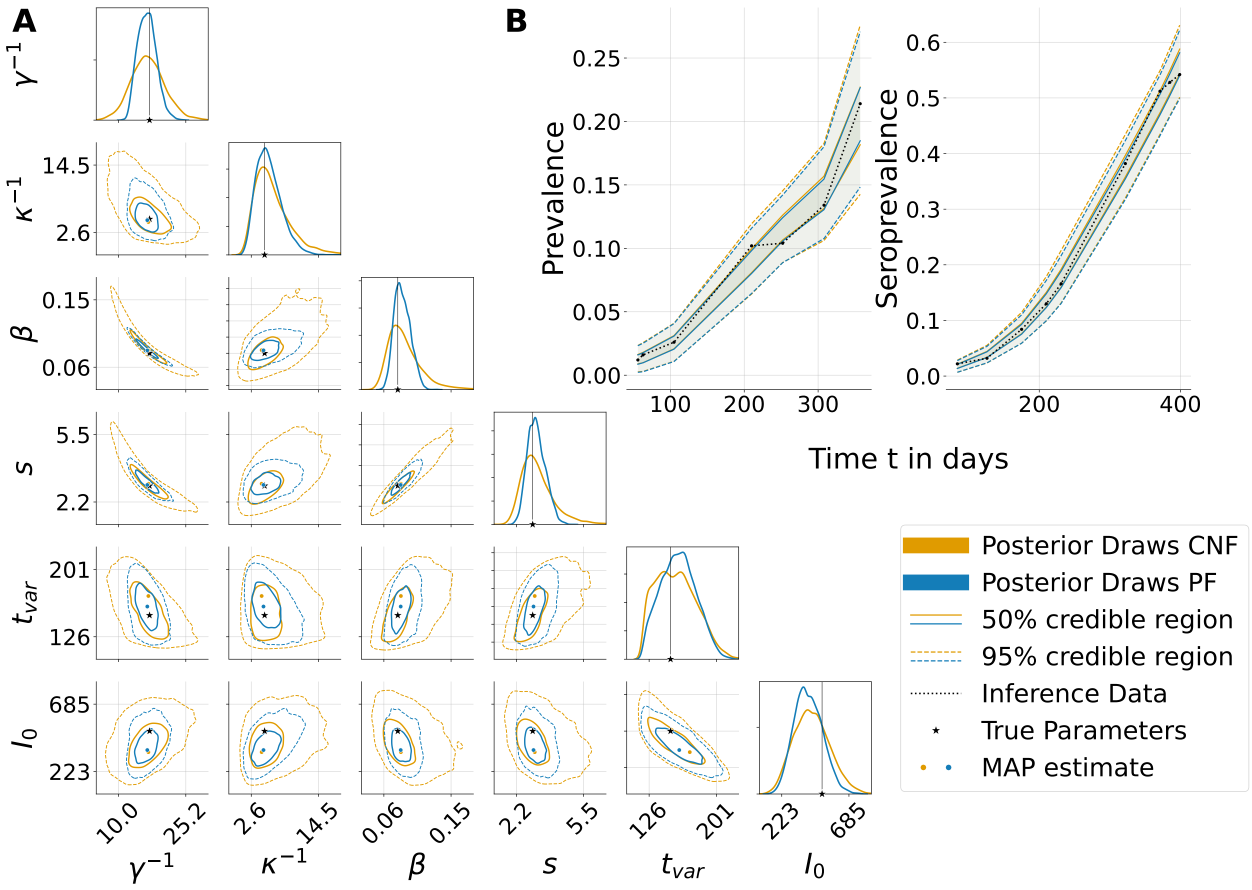

Figure S5.6: **Results of the two-variant SEIR model for *s-1-2-3*.**
A Posterior approximations from 10,000 samples. Contour gives the 50% (solid) and 95% (dashed) credible regions, coloured by method. Diagonals show the 1D marginals. Black stars mark the true parameters, coloured circles the joint MAP estimates. **B** Posterior predictive fit: bands give the 50% and 95% pointwise predictive intervals from the same samples (line styles as in **A**) with inference data shown as a dotted line.

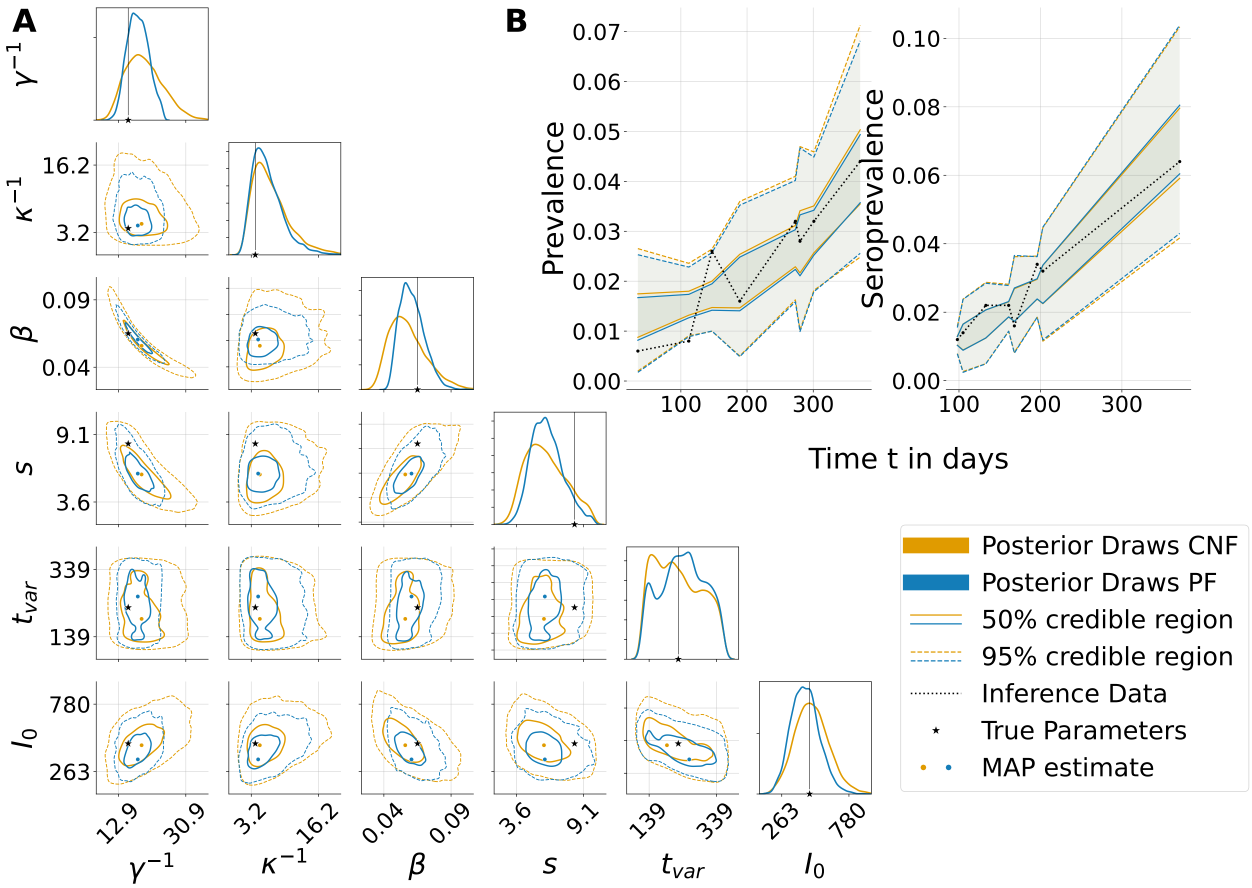

Figure S5.7: **Results of the two-variant SEIR model for *s-3*.**
A Posterior approximations from 10,000 samples. Contour gives the 50% (solid) and 95% (dashed) credible regions, coloured by method. Diagonals show the 1D marginals. Black stars mark the true parameters, coloured circles the joint MAP estimates. **B** Posterior predictive fit: bands give the 50% and 95% pointwise predictive intervals from the same samples (line styles as in **A**) with inference data shown as a dotted line.

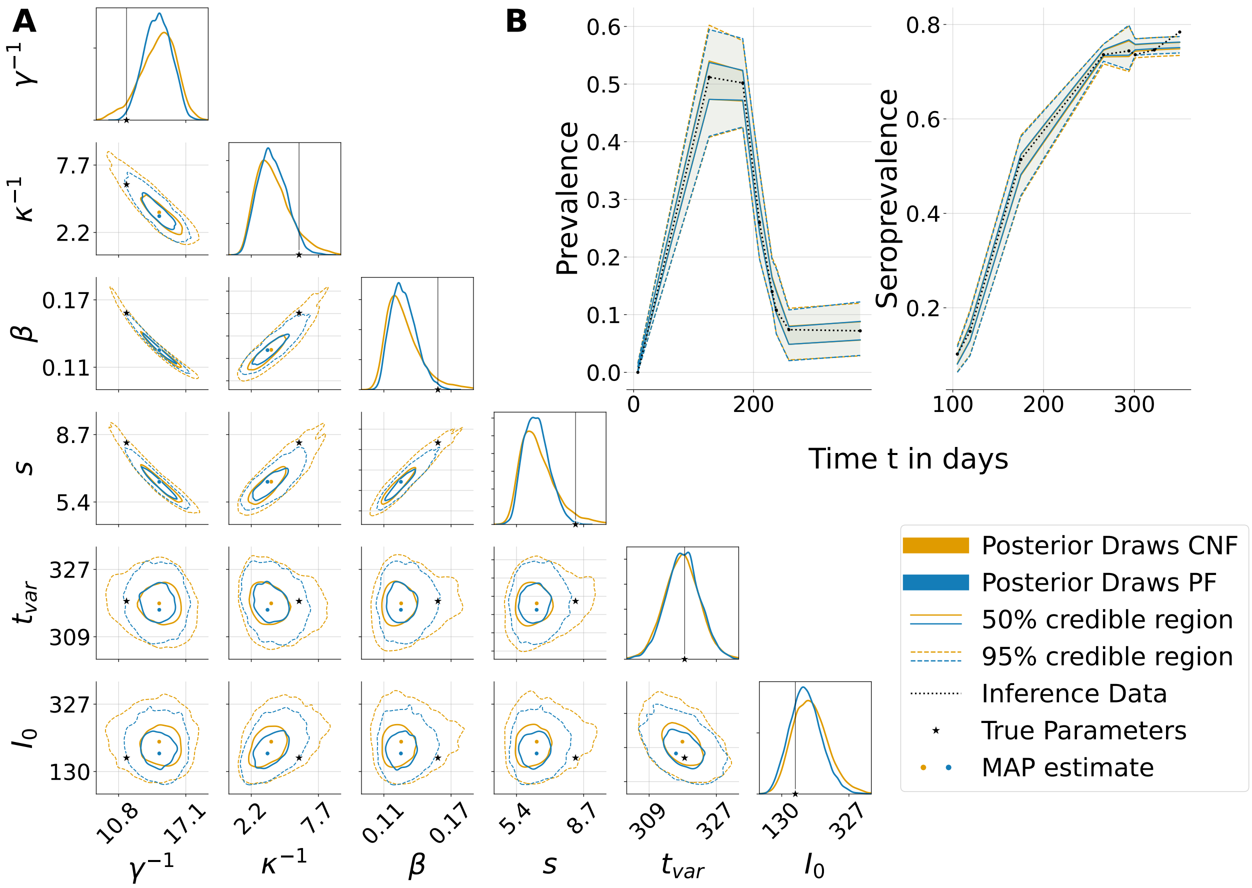

Figure S5.8: **Results of the two-variant SEIR model for *s-4*.**
A Posterior approximations from 10,000 samples. Contour gives the 50% (solid) and 95% (dashed) credible regions, coloured by method. Diagonals show the 1D marginals. Black stars mark the true parameters, coloured circles the joint MAP estimates. **B** Posterior predictive fit: bands give the 50% and 95% pointwise predictive intervals from the same samples (line styles as in **A**) with inference data shown as a dotted line.

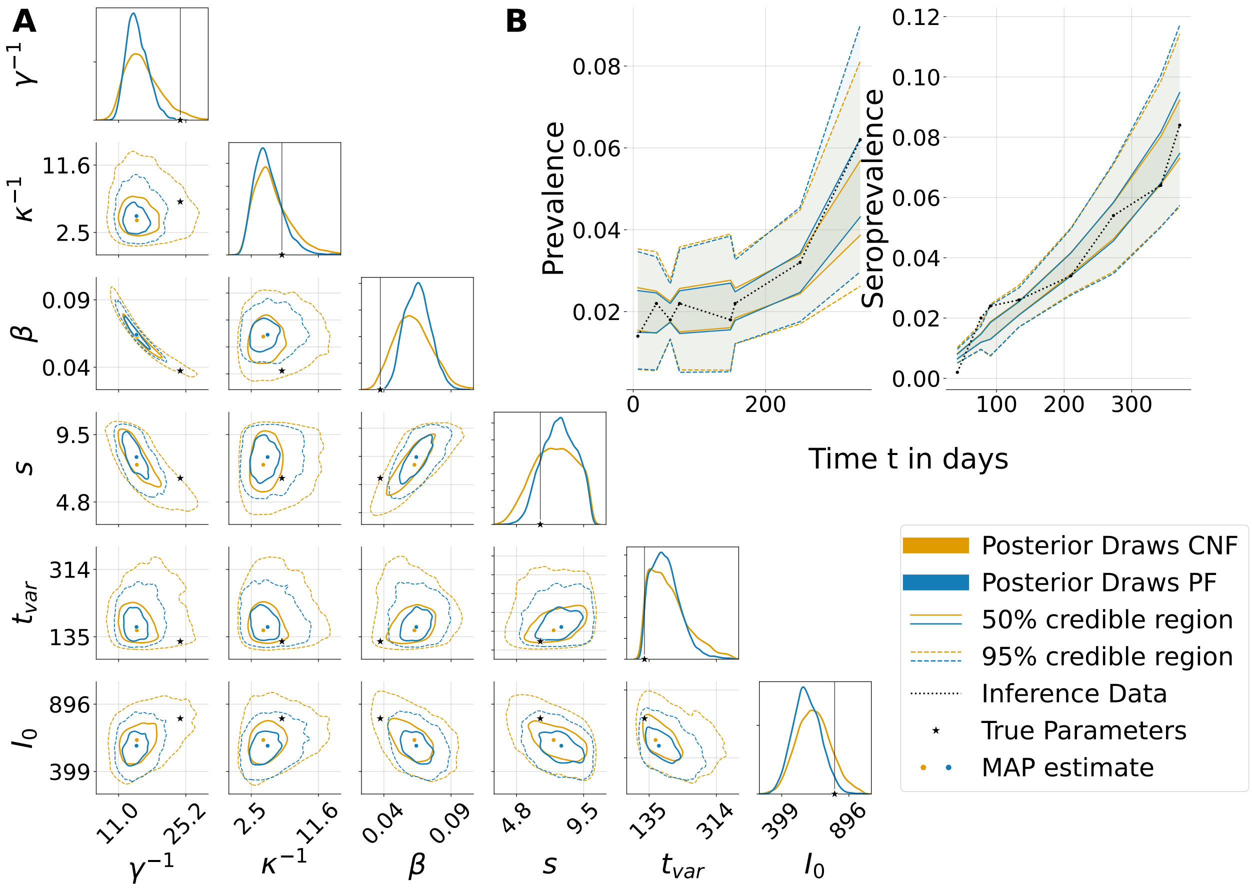

Figure S5.9: **Results of the two-variant SEIR model for *s-5*.**
A Posterior approximations from 10,000 samples. Contour gives the 50% (solid) and 95% (dashed) credible regions, coloured by method. Diagonals show the 1D marginals. Black stars mark the true parameters, coloured circles the joint MAP estimates. **B** Posterior predictive fit: bands give the 50% and 95% pointwise predictive intervals from the same samples (line styles as in **A**) with inference data shown as a dotted line.

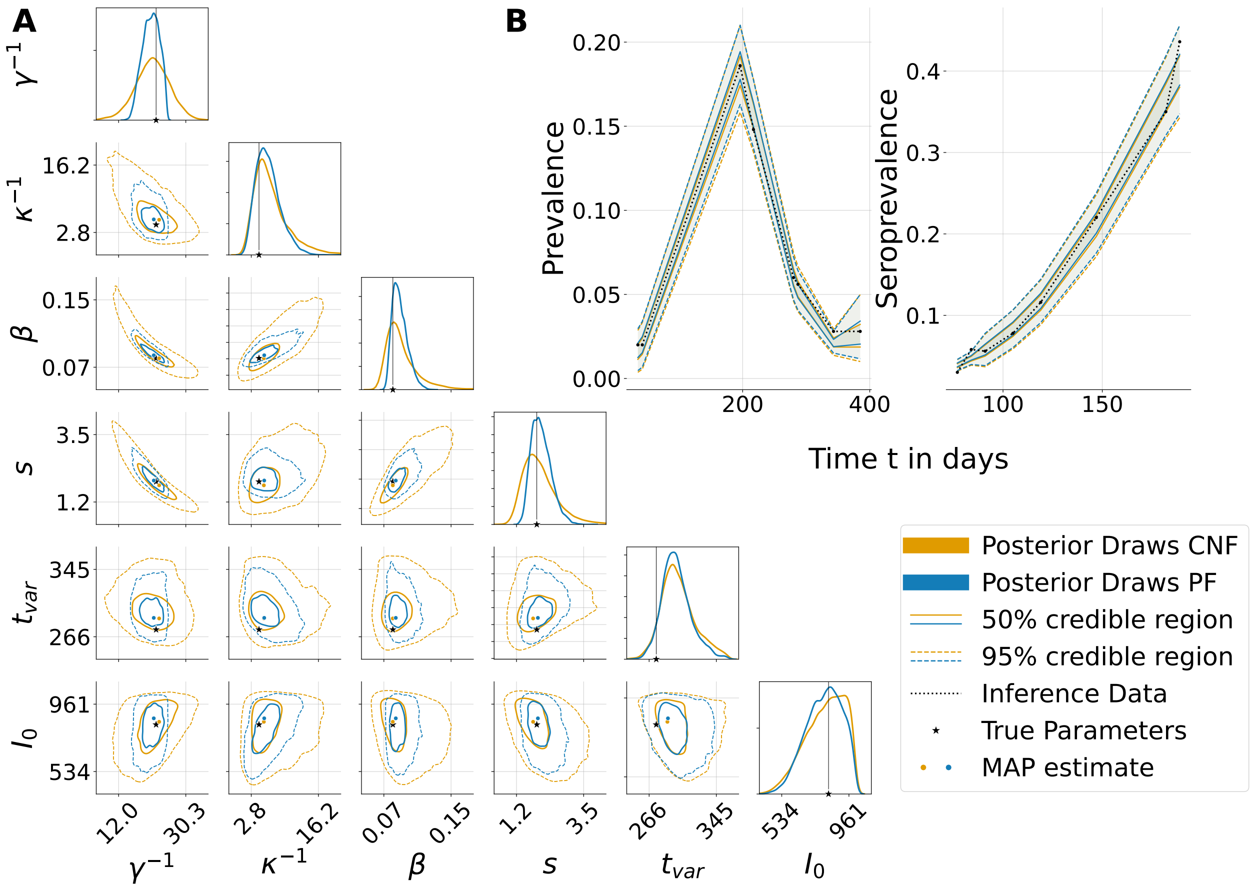

Figure S5.10: **Results of the two-variant SEIR model for *s-6*.**
A Posterior approximations from 10,000 samples. Contour gives the 50% (solid) and 95% (dashed) credible regions, coloured by method. Diagonals show the 1D marginals. Black stars mark the true parameters, coloured circles the joint MAP estimates. **B** Posterior predictive fit: bands give the 50% and 95% pointwise predictive intervals from the same samples (line styles as in **A**) with inference data shown as a dotted line.

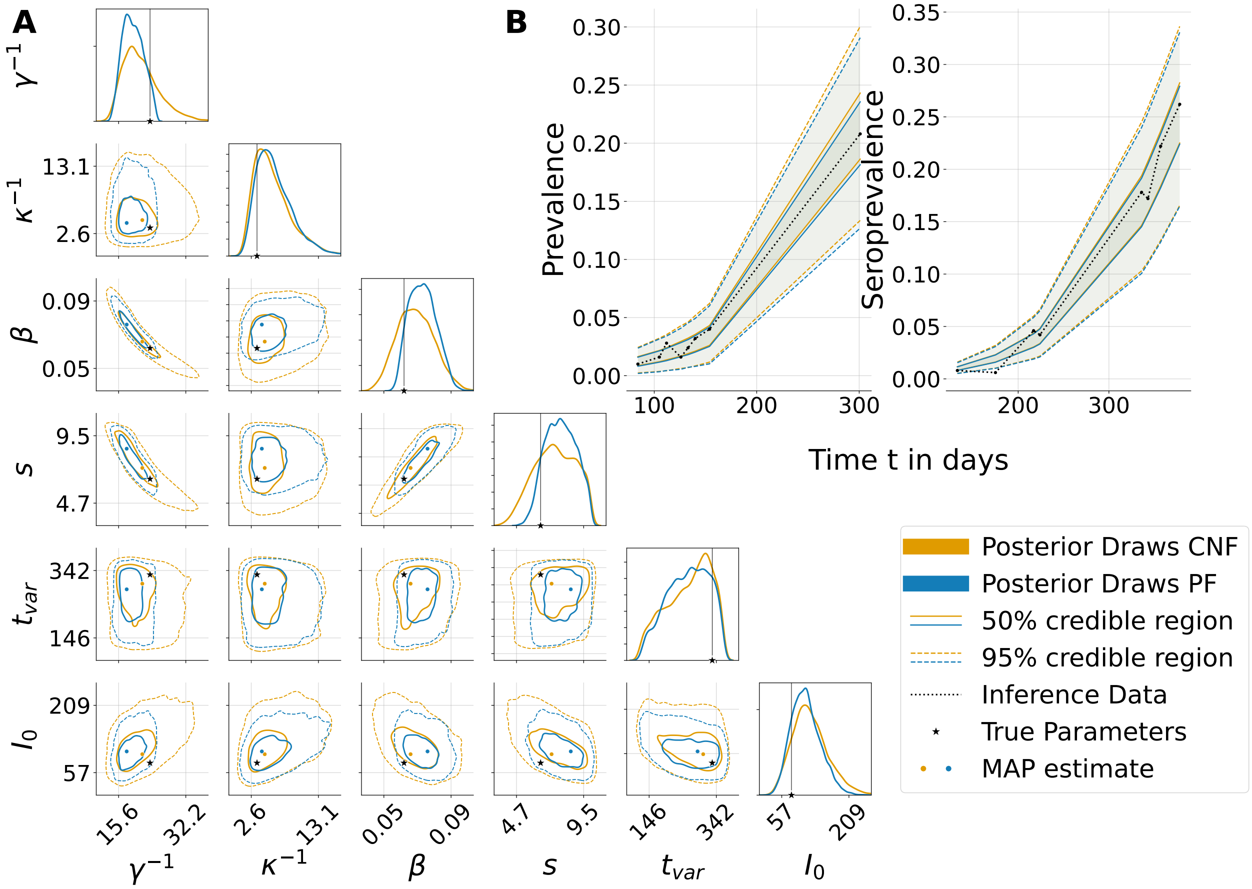

Figure S5.11: **Results of the two-variant SEIR model for *s*-7.**
A Posterior approximations from 10,000 samples. Contour gives the 50% (solid) and 95% (dashed) credible regions, coloured by method. Diagonals show the 1D marginals. Black stars mark the true parameters, coloured circles the joint MAP estimates. **B** Posterior predictive fit: bands give the 50% and 95% pointwise predictive intervals from the same samples (line styles as in **A**) with inference data shown as a dotted line.

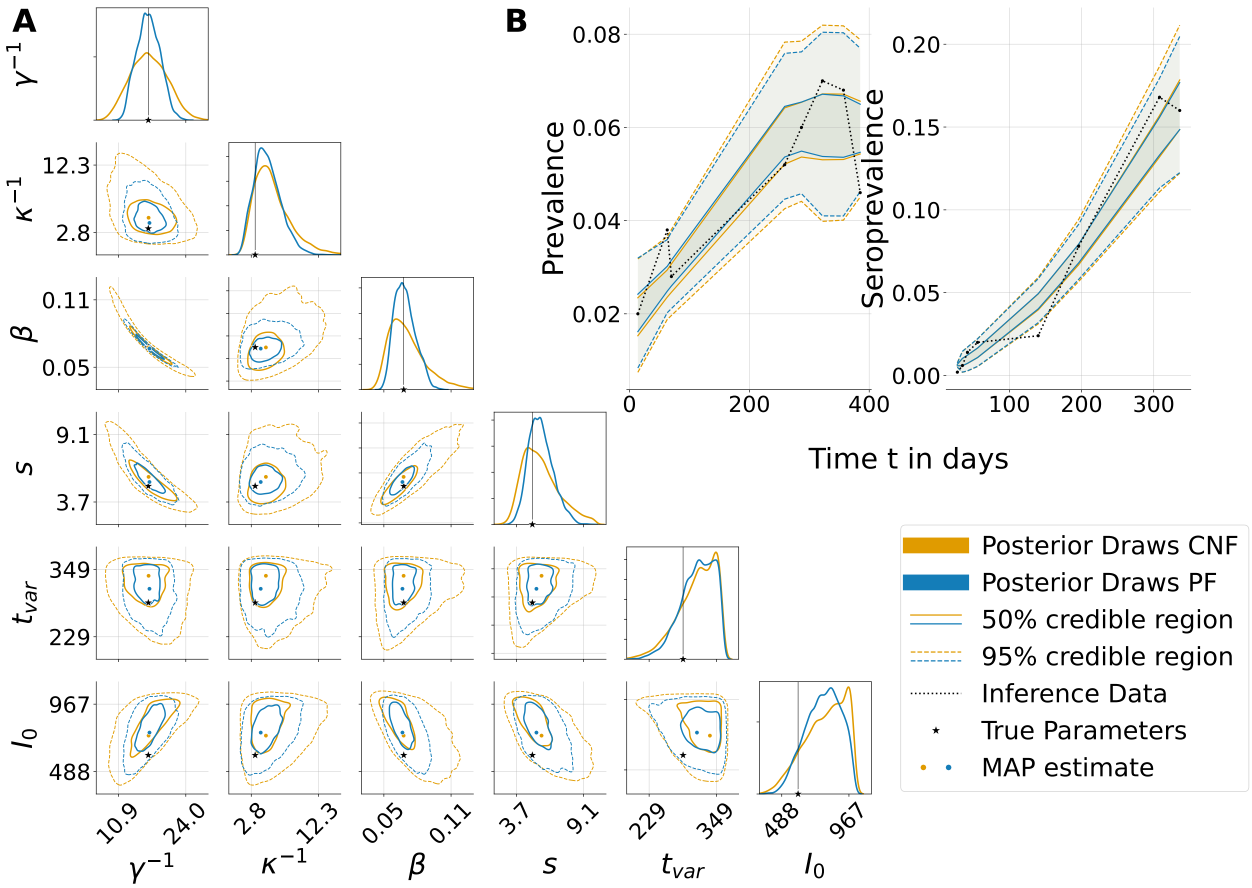

Figure S5.12: **Results of the two-variant SEIR model for *s-8*.**

A Posterior approximations from 10,000 samples. Contour gives the 50% (solid) and 95% (dashed) credible regions, coloured by method. Diagonals show the 1D marginals. Black stars mark the true parameters, coloured circles the joint MAP estimates. **B** Posterior predictive fit: bands give the 50% and 95% pointwise predictive intervals from the same samples (line styles as in **A**) with inference data shown as a dotted line.

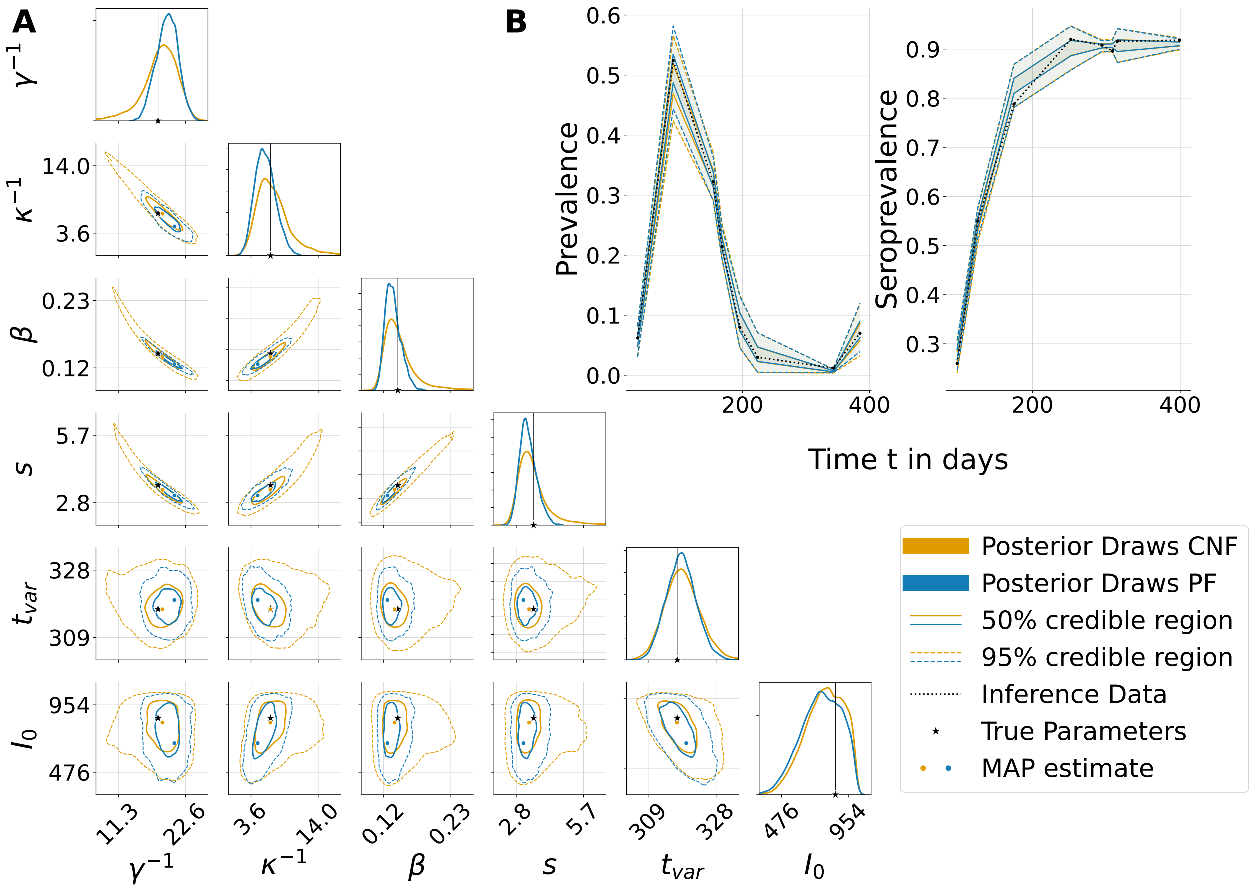

Figure S5.13: **Results of the two-variant SEIR model for *s-9*.**
A Posterior approximations from 10,000 samples. Contour gives the 50% (solid) and 95% (dashed) credible regions, coloured by method. Diagonals show the 1D marginals. Black stars mark the true parameters, coloured circles the joint MAP estimates. **B** Posterior predictive fit: bands give the 50% and 95% pointwise predictive intervals from the same samples (line styles as in **A**) with inference data shown as a dotted line.

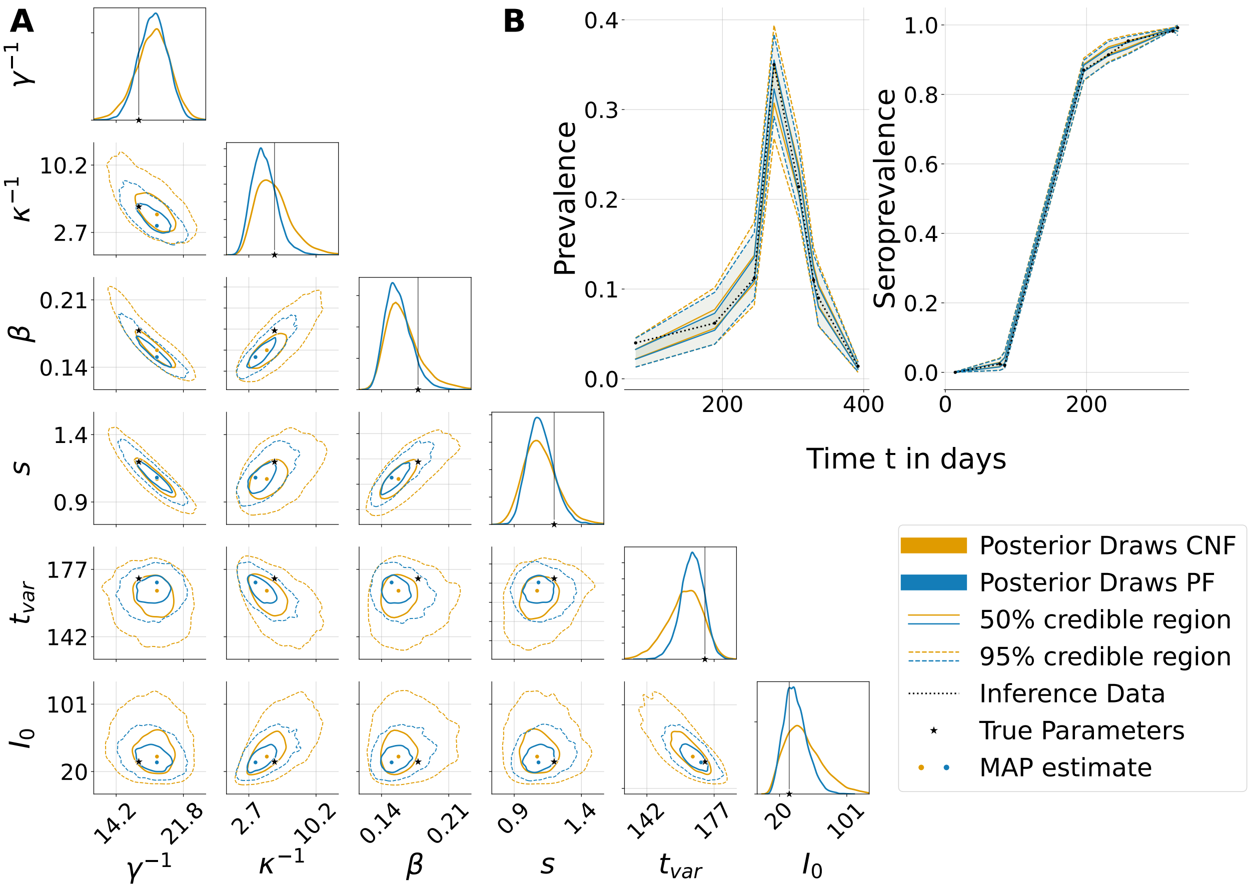

Figure S5.14: **Results of the two-variant SEIR model for *s-10*.**
A Posterior approximations from 10,000 samples. Contour gives the 50% (solid) and 95% (dashed) credible regions, coloured by method. Diagonals show the 1D marginals. Black stars mark the true parameters, coloured circles the joint MAP estimates. **B** Posterior predictive fit: bands give the 50% and 95% pointwise predictive intervals from the same samples (line styles as in **A**) with inference data shown as a dotted line.

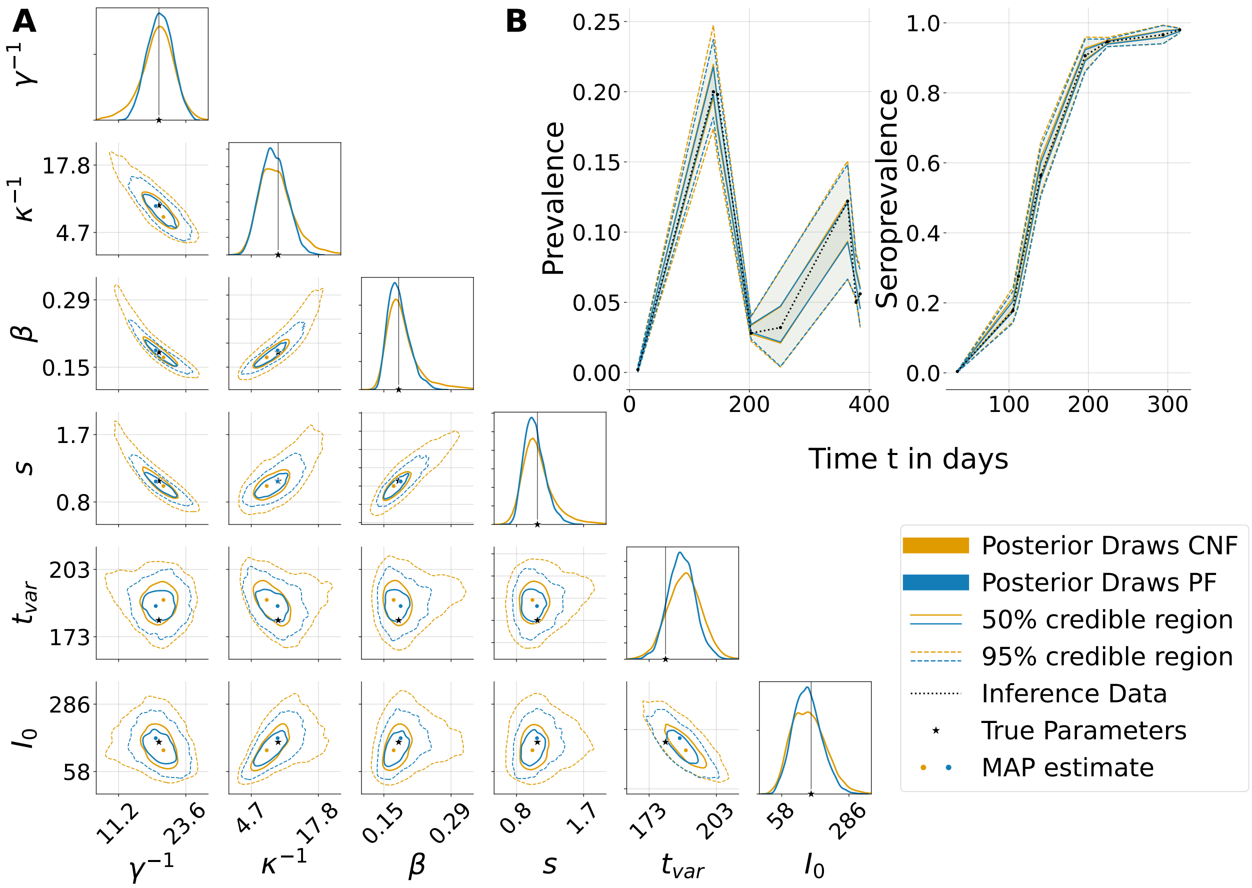

Figure S5.15: **Results of the two-variant SEIR model for *s-11*.**
A Posterior approximations from 10,000 samples. Contour gives the 50% (solid) and 95% (dashed) credible regions, coloured by method. Diagonals show the 1D marginals. Black stars mark the true parameters, coloured circles the joint MAP estimates. **B** Posterior predictive fit: bands give the 50% and 95% pointwise predictive intervals from the same samples (line styles as in **A**) with inference data shown as a dotted line.

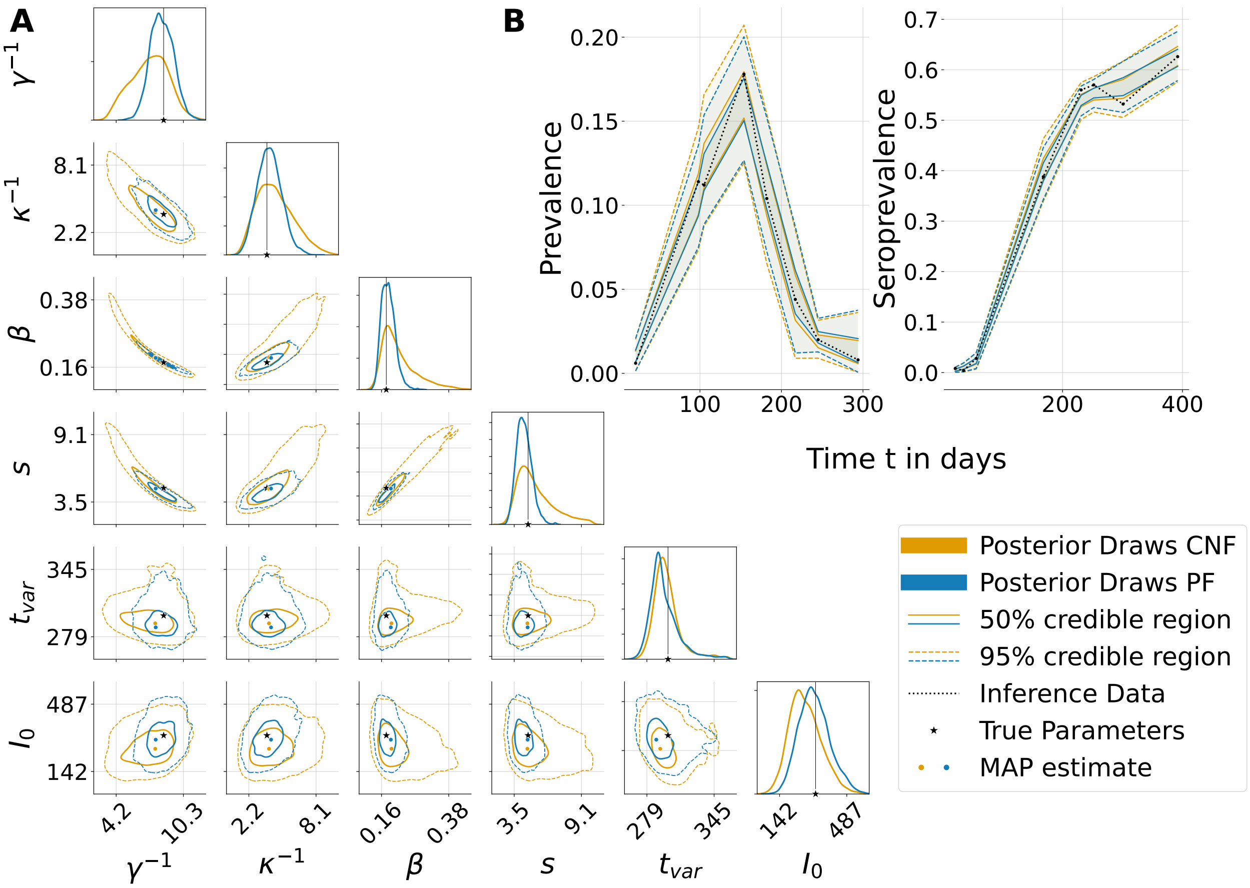

Figure S5.16: **Results of the two-variant SEIR model for *s-12*.**
A Posterior approximations from 10,000 samples. Contour gives the 50% (solid) and 95% (dashed) credible regions, coloured by method. Diagonals show the 1D marginals. Black stars mark the true parameters, coloured circles the joint MAP estimates. **B** Posterior predictive fit: bands give the 50% and 95% pointwise predictive intervals from the same samples (line styles as in **A**) with inference data shown as a dotted line.

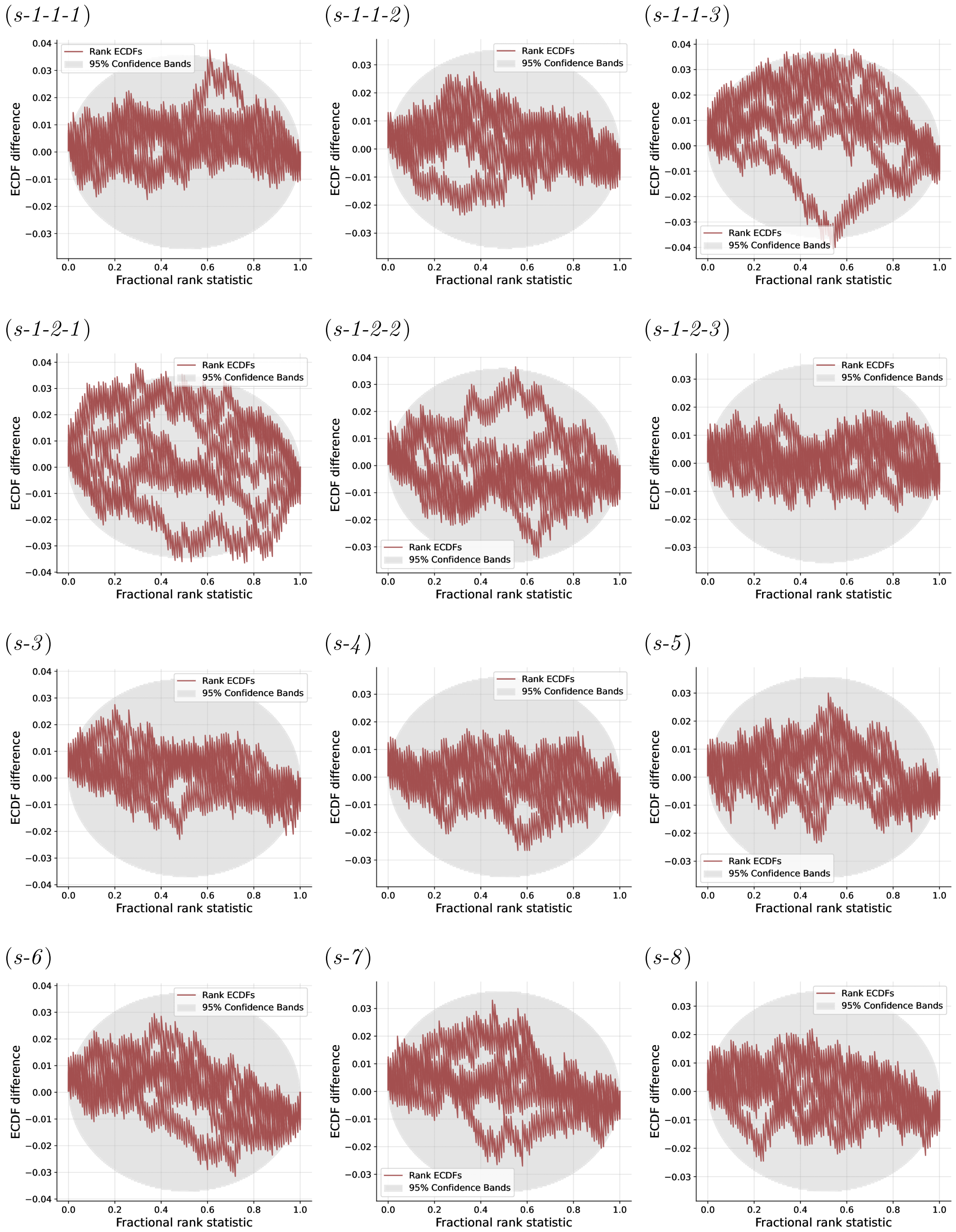
(s-1-1-1)
(s-1-1-2)
(s-1-1-3)
(s-1-2-1)
(s-1-2-2)
(s-1-2-3)
(s-3)
(s-4)
(s-5)
(s-6)
(s-7)
(s-8)
Rank ECDFs
95% Confidence Bands
ECDF difference
Fractional rank statistic

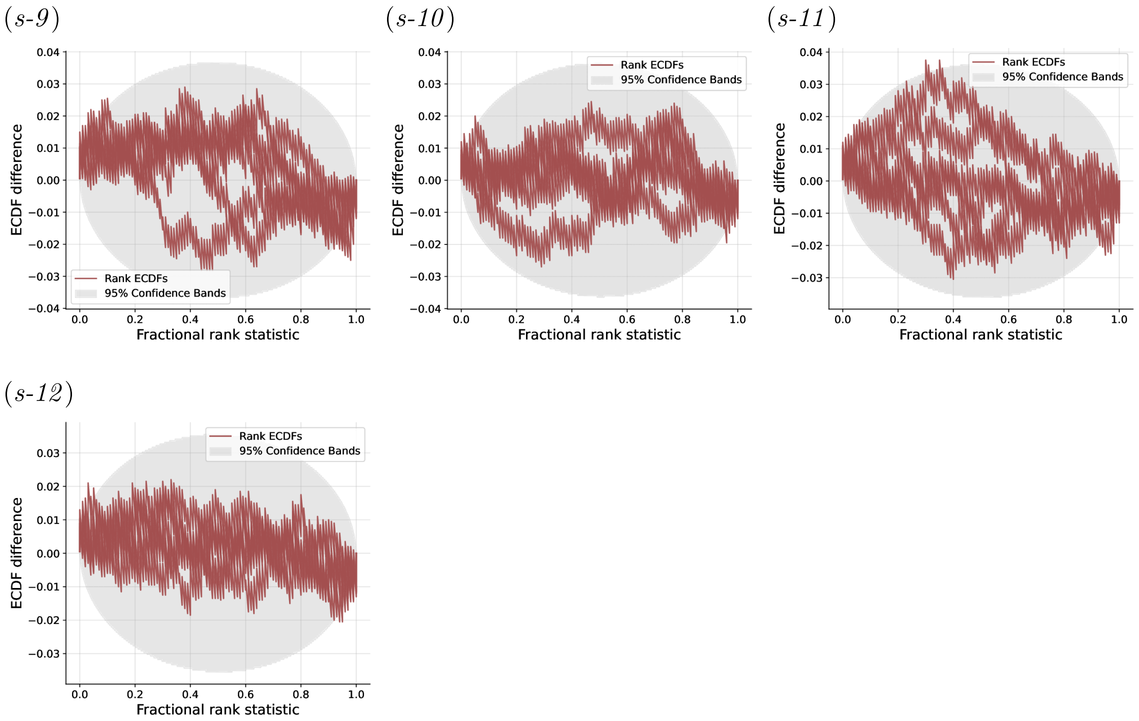

Figure S5.17: **ECDF Calibration plots for the full SEIR model on sparse datasets.**

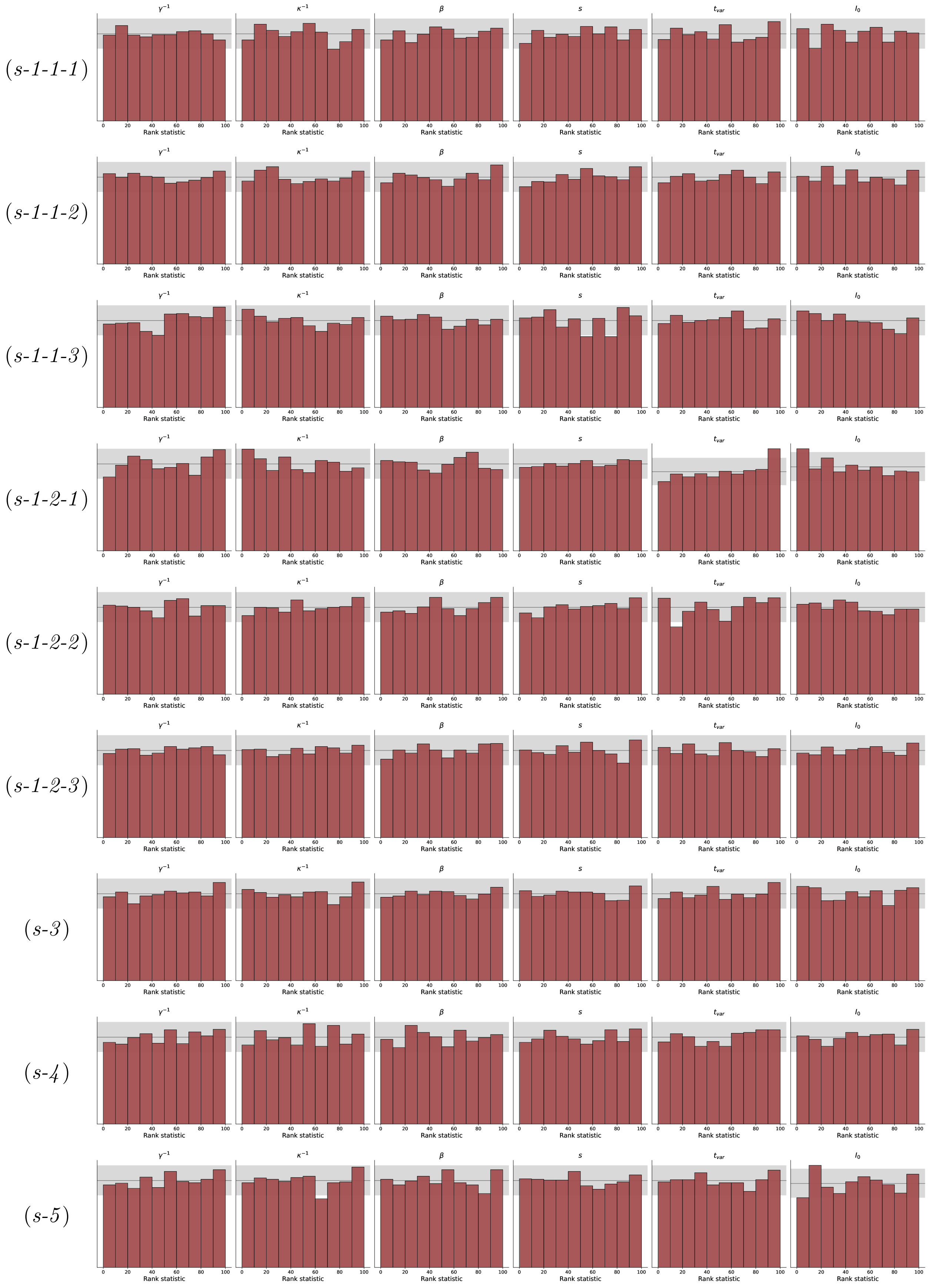
(s-1-1-1)
(s-1-1-2)
(s-1-1-3)
(s-1-2-1)
(s-1-2-2)
(s-1-2-3)
(s-3)
(s-4)
(s-5)
γ^{-1}
κ^{-1}
β
s
t_{var}
I_0
Rank statistic
0
20
40
60
80
100

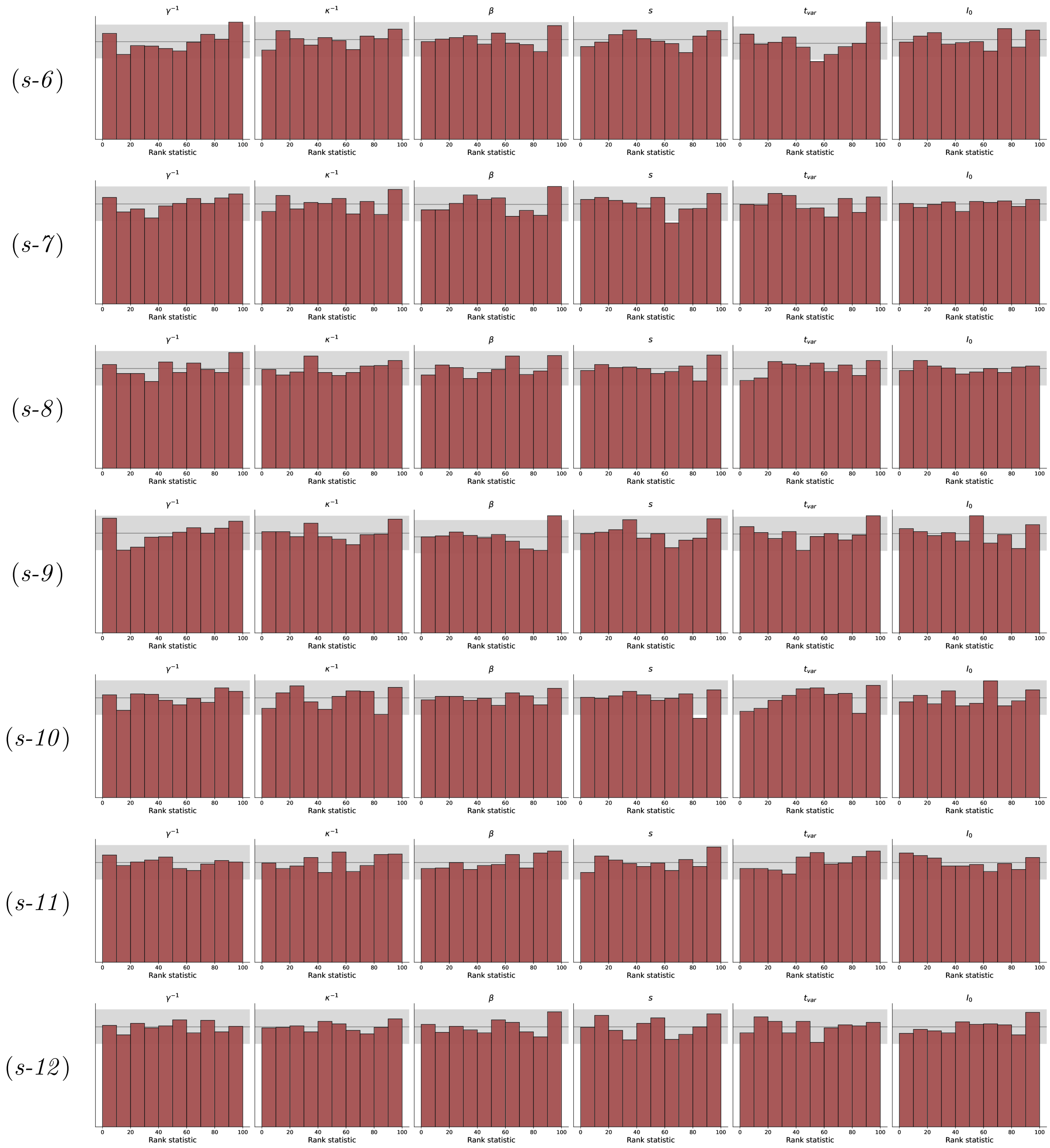

Figure S5.17: **SBC Histograms for the full SEIR model on sparse datasets.**

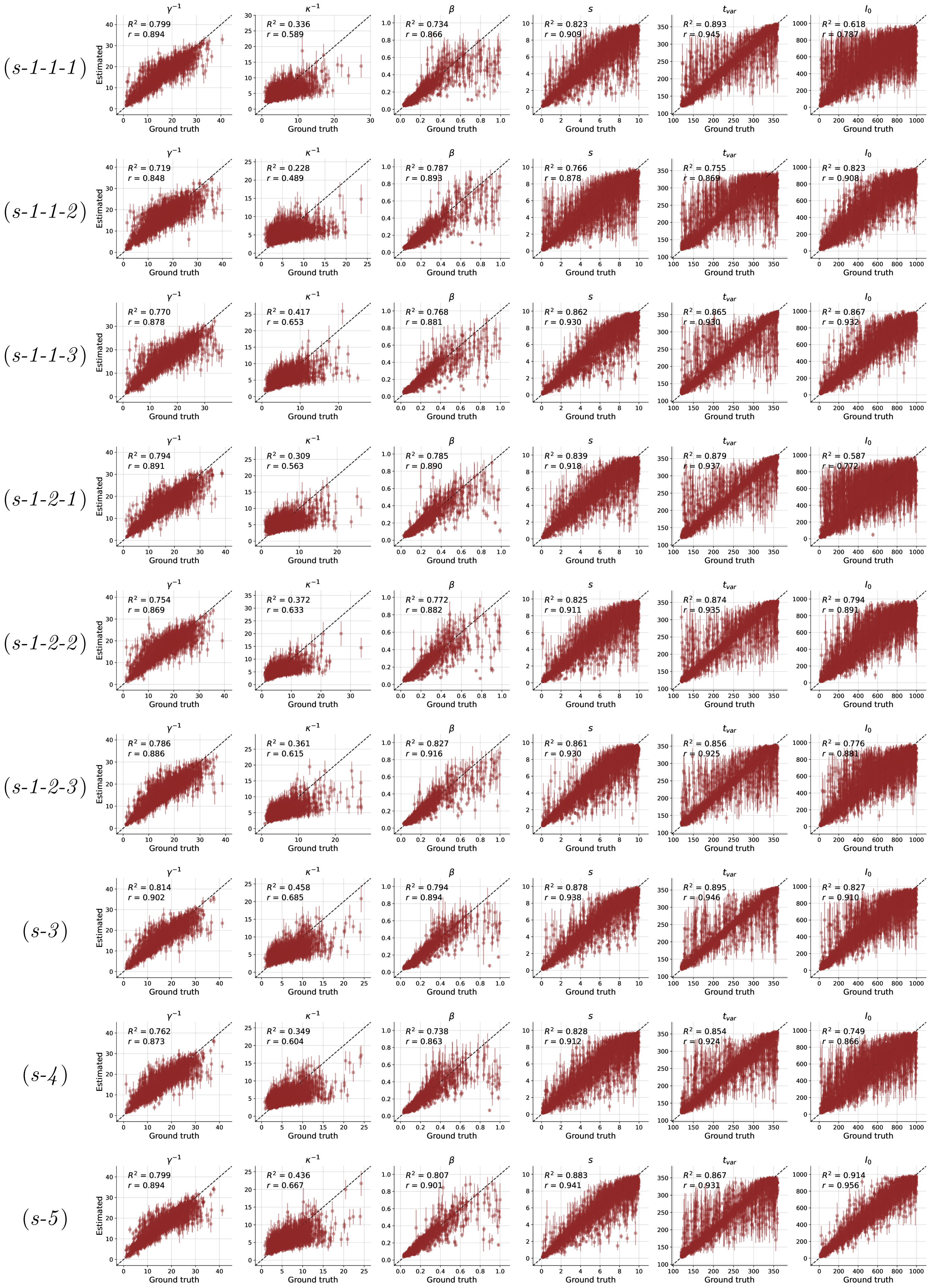

γ^{-1}
κ^{-1}
β
s
t_{var}
I_0
Estimated
Ground truth
(s-1-1-1)
R^2 = 0.799 r = 0.894
R^2 = 0.336 r = 0.589
R^2 = 0.734 r = 0.866
R^2 = 0.823 r = 0.909
R^2 = 0.893 r = 0.945
R^2 = 0.618 r = 0.787
(s-1-1-2)
R^2 = 0.719 r = 0.848
R^2 = 0.228 r = 0.489
R^2 = 0.787 r = 0.893
R^2 = 0.766 r = 0.878
R^2 = 0.755 r = 0.869
R^2 = 0.823 r = 0.908
(s-1-1-3)
R^2 = 0.770 r = 0.878
R^2 = 0.417 r = 0.653
R^2 = 0.768 r = 0.881
R^2 = 0.862 r = 0.930
R^2 = 0.865 r = 0.930
R^2 = 0.867 r = 0.932
(s-1-2-1)
R^2 = 0.794 r = 0.891
R^2 = 0.309 r = 0.563
R^2 = 0.785 r = 0.890
R^2 = 0.839 r = 0.918
R^2 = 0.879 r = 0.937
R^2 = 0.587 r = 0.772
(s-1-2-2)
R^2 = 0.754 r = 0.869
R^2 = 0.372 r = 0.633
R^2 = 0.772 r = 0.882
R^2 = 0.825 r = 0.911
R^2 = 0.874 r = 0.935
R^2 = 0.794 r = 0.891
(s-1-2-3)
R^2 = 0.786 r = 0.886
R^2 = 0.361 r = 0.615
R^2 = 0.827 r = 0.916
R^2 = 0.861 r = 0.930
R^2 = 0.856 r = 0.925
R^2 = 0.776 r = 0.881
(s-3)
R^2 = 0.814 r = 0.902
R^2 = 0.458 r = 0.685
R^2 = 0.794 r = 0.894
R^2 = 0.878 r = 0.938
R^2 = 0.895 r = 0.946
R^2 = 0.827 r = 0.910
(s-4)
R^2 = 0.762 r = 0.873
R^2 = 0.349 r = 0.604
R^2 = 0.738 r = 0.863
R^2 = 0.828 r = 0.912
R^2 = 0.854 r = 0.924
R^2 = 0.749 r = 0.866
(s-5)
R^2 = 0.799 r = 0.894
R^2 = 0.436 r = 0.667
R^2 = 0.807 r = 0.901
R^2 = 0.883 r = 0.941
R^2 = 0.867 r = 0.931
R^2 = 0.914 r = 0.956

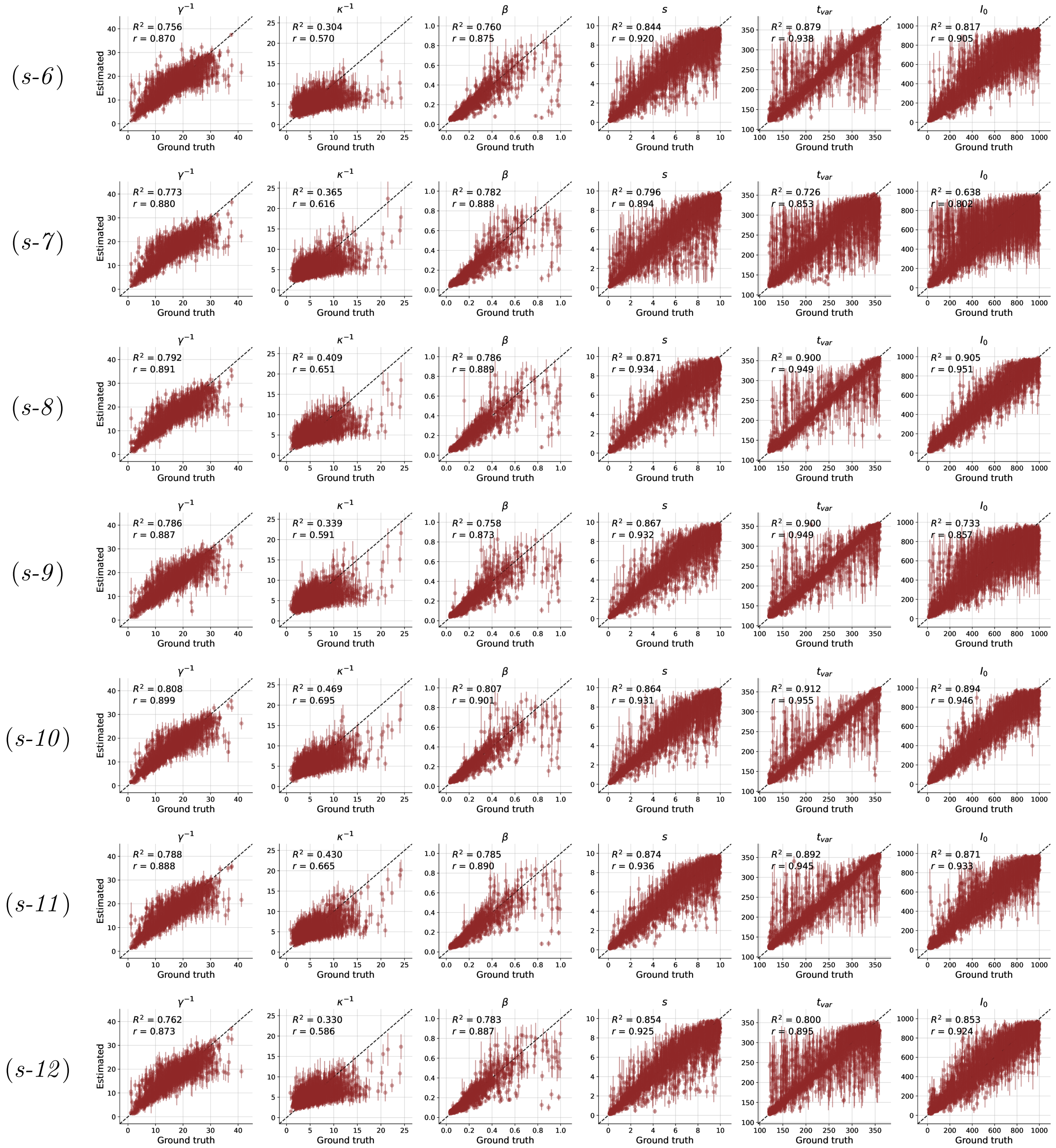

Figure S5.17: **Parameter recovery for the full SEIR model on sparse datasets.**

S5.B Supplementary Tables

Table S5.1: Posterior MAP estimates with 95% intervals for datasets *s-1-1-1* to *s-4*.

Dataset	Method	γ^{-1}	κ^{-1}	β	s	t_{var}	I_0
s-1-1-1	True	17.00	5.00	0.0800	3.00	150.0	500.0
	CNF	15.69 (9.10, 20.64)	4.29 (2.26, 13.35)	0.0871 (0.07, 0.15)	3.16 (2.4, 5.5)	171.0 (125.5, 201.7)	331.8 (184.8, 600.5)
	PF	14.65 (11.88, 18.28)	5.52 (2.52, 10.04)	0.0933 (0.08, 0.12)	3.29 (2.6, 4.2)	165.3 (135.0, 201.8)	349.4 (182.6, 529.2)
s-1-1-2	True	17.00	5.00	0.0800	3.00	150.0	500.0
	CNF	15.95 (8.33, 29.10)	5.06 (2.29, 15.87)	0.0841 (0.05, 0.16)	2.91 (1.6, 5.9)	149.5 (122.0, 334.5)	440.4 (233.9, 885.4)
	PF	14.44 (11.08, 19.55)	5.12 (2.16, 11.32)	0.0913 (0.07, 0.12)	3.12 (2.3, 4.4)	147.6 (122.0, 206.8)	443.2 (260.0, 717.3)
s-1-1-3	True	17.00	5.00	0.0800	3.00	150.0	500.0
	CNF	14.26 (6.58, 19.90)	5.27 (2.69, 16.47)	0.0945 (0.07, 0.21)	3.65 (2.7, 8.1)	174.5 (128.0, 218.8)	395.0 (231.7, 633.0)
	PF	13.17 (10.23, 17.06)	5.47 (2.74, 11.76)	0.1023 (0.08, 0.13)	3.81 (2.9, 5.0)	187.1 (135.3, 215.2)	335.1 (218.9, 585.1)
s-1-2-1	True	17.00	5.00	0.0800	3.00	150.0	500.0
	CNF	16.23 (8.78, 20.53)	4.76 (2.18, 13.49)	0.0854 (0.07, 0.16)	3.03 (2.4, 5.7)	168.7 (128.7, 197.1)	345.3 (197.0, 612.8)
	PF	12.82 (10.08, 16.60)	3.62 (2.07, 9.11)	0.1016 (0.08, 0.13)	3.74 (2.8, 4.8)	180.6 (125.0, 205.8)	259.9 (171.8, 525.3)
s-1-2-2	True	17.00	5.00	0.0800	3.00	150.0	500.0
	CNF	14.74 (7.97, 19.81)	5.05 (2.22, 14.33)	0.0913 (0.07, 0.17)	3.26 (2.4, 6.2)	153.6 (121.9, 195.4)	363.1 (210.8, 592.6)
	PF	15.03 (11.12, 17.62)	3.96 (2.28, 9.92)	0.0891 (0.08, 0.12)	3.23 (2.7, 4.4)	166.0 (124.9, 193.9)	290.7 (193.8, 547.7)
s-1-2-3	True	17.00	5.00	0.0800	3.00	150.0	500.0
	CNF	16.74 (9.38, 24.56)	4.48 (2.16, 14.86)	0.0838 (0.06, 0.15)	3.10 (2.1, 5.6)	171.6 (121.7, 202.5)	356.0 (213.4, 683.7)
	PF	16.49 (12.85, 20.78)	4.80 (2.32, 10.87)	0.0836 (0.07, 0.11)	3.04 (2.4, 4.1)	159.8 (123.7, 197.0)	370.2 (233.6, 602.9)
s-3	True	15.47	3.97	0.0655	8.40	226.0	477.3
	CNF	19.05 (11.70, 30.61)	4.84 (2.24, 16.72)	0.0561 (0.04, 0.09)	5.89 (3.4, 9.5)	192.4 (125.2, 351.1)	464.8 (254.9, 774.2)
	PF	18.03 (13.06, 23.75)	4.52 (2.20, 13.81)	0.0609 (0.05, 0.08)	5.97 (4.0, 9.0)	258.8 (125.7, 351.9)	356.1 (249.7, 670.0)
s-4	True	11.57	6.11	0.1605	8.34	318.0	169.6
	CNF	14.64 (10.67, 17.38)	3.83 (1.88, 7.52)	0.1276 (0.11, 0.17)	6.43 (5.3, 8.8)	318.1 (308.5, 327.9)	217.2 (125.3, 327.0)
	PF	14.65 (12.05, 16.83)	3.53 (1.93, 6.33)	0.1274 (0.11, 0.15)	6.42 (5.4, 7.7)	316.3 (309.0, 327.3)	182.7 (118.0, 295.9)

Table S5.2: Posterior MAP estimates with 95% intervals for datasets *s-5* to *s-12*.

Dataset	Method	γ^{-1}	κ^{-1}	β	s	t_{var}	I_0
s-5	True	24.05	6.63	0.0423	6.45	122.0	789.9
	CNF	14.87 (10.53, 25.23)	4.12 (1.90, 12.11)	0.0677 (0.04, 0.10)	7.40 (4.6, 9.9)	152.4 (122.5, 311.3)	631.4 (403.4, 898.7)
	PF	14.80 (11.28, 20.00)	4.70 (1.86, 9.28)	0.0691 (0.05, 0.09)	7.95 (5.6, 9.8)	161.2 (124.1, 270.3)	589.1 (394.6, 792.6)
s-6	True	22.21	4.37	0.0807	1.91	274.0	831.2
	CNF	23.04 (11.66, 30.11)	5.34 (2.28, 16.69)	0.0806 (0.07, 0.16)	1.79 (1.2, 3.7)	287.3 (265.1, 346.3)	848.9 (541.5, 987.1)
	PF	21.57 (16.38, 24.61)	5.41 (2.44, 11.69)	0.0843 (0.08, 0.11)	1.95 (1.5, 2.8)	287.9 (267.4, 337.8)	871.0 (569.5, 983.7)
s-7	True	23.34	3.52	0.0631	6.43	329.0	79.0
	CNF	21.44 (14.55, 32.04)	4.72 (2.04, 14.24)	0.0672 (0.05, 0.09)	7.22 (4.6, 9.9)	303.3 (133.4, 355.4)	98.7 (54.1, 215.2)
	PF	17.58 (14.81, 24.00)	4.27 (2.26, 13.45)	0.0777 (0.06, 0.09)	8.58 (5.9, 9.8)	286.9 (140.2, 353.7)	105.0 (55.1, 182.7)
s-8	True	16.66	3.35	0.0699	4.95	289.0	604.8
	CNF	16.71 (10.21, 23.84)	4.86 (2.19, 12.33)	0.0698 (0.05, 0.11)	5.69 (3.5, 9.1)	337.6 (233.4, 358.4)	743.8 (485.6, 994.6)
	PF	16.88 (12.65, 21.03)	4.13 (2.05, 9.20)	0.0688 (0.06, 0.09)	5.27 (3.9, 7.8)	314.5 (249.0, 357.2)	765.3 (535.4, 981.7)
s-9	True	17.98	6.59	0.1430	3.55	317.0	859.7
	CNF	18.72 (10.90, 23.01)	6.57 (3.21, 14.12)	0.1378 (0.12, 0.24)	3.36 (2.7, 5.9)	317.1 (309.7, 329.0)	828.2 (496.7, 986.9)
	PF	20.74 (15.93, 22.92)	4.59 (3.07, 9.05)	0.1263 (0.12, 0.16)	3.12 (2.7, 4.1)	319.7 (310.5, 326.6)	682.2 (466.5, 981.3)
s-10	True	16.77	5.57	0.1786	1.18	172.0	31.8
	CNF	18.82 (14.12, 21.96)	4.72 (2.50, 10.29)	0.1599 (0.14, 0.21)	1.04 (0.8, 1.4)	166.0 (141.6, 177.8)	38.1 (15.7, 103.9)
	PF	18.81 (15.23, 21.36)	3.46 (2.18, 7.57)	0.1534 (0.14, 0.19)	1.05 (0.9, 1.3)	170.4 (153.0, 176.7)	31.1 (16.6, 70.1)
s-11	True	18.63	9.93	0.1807	1.07	180.0	157.8
	CNF	19.46 (11.30, 23.90)	7.71 (4.34, 17.87)	0.1702 (0.15, 0.30)	1.00 (0.8, 1.7)	189.2 (173.9, 203.5)	130.0 (53.6, 271.5)
	PF	18.04 (14.41, 23.31)	9.83 (4.47, 14.07)	0.1848 (0.15, 0.23)	1.06 (0.8, 1.4)	186.5 (175.9, 199.7)	171.0 (60.6, 242.0)
s-12	True	8.51	3.80	0.1731	4.66	299.0	327.2
	CNF	7.75 (3.99, 10.39)	3.98 (1.98, 8.04)	0.1905 (0.14, 0.37)	4.60 (3.3, 9.2)	292.2 (279.0, 340.0)	258.6 (133.9, 450.9)
	PF	7.79 (6.40, 10.41)	4.16 (2.11, 6.19)	0.1882 (0.14, 0.23)	4.62 (3.3, 5.7)	288.3 (275.8, 336.6)	305.6 (180.5, 491.7)

Table S5.3: **Effective sample sizes (ESS) per parameter and model for sparse datasets.** ESS computed on the last 10,000 samples of the chains resulting from running the PF method on the two-variant SEIR model and using a maximum lag size of 250 for the autocorrelation.

**Dataset**	$\boldsymbol{\gamma^{-1}}$	$\boldsymbol{\kappa^{-1}}$	$\boldsymbol{\beta}$	**s**	$\mathbf{t}_{\text{var}}$	$\mathbf{I_0}$
s-1-1-1	313.9	536.5	302.4	364.4	1550.2	1641.1
s-1-1-2	208.8	938.9	211.3	261.2	907.0	757.0
s-1-1-3	292.1	771.5	287.9	304.3	914.6	1157.6
s-1-2-1	252.0	1121.2	258.3	308.7	463.0	587.4
s-1-2-2	326.5	874.5	303.6	380.3	738.2	1216.5
s-1-2-3	301.1	1234.2	309.9	343.2	760.9	873.2
s-3	578.3	1576.9	564.7	1850.8	831.4	1461.8
s-4	1346.6	1628.2	1306.1	2980.6	1634.9	3049.4
s-5	702.9	1822.5	703.3	1747.8	1224.0	1677.9
s-6	1217.9	1722.3	1169.8	1812.0	1434.3	2402.2
s-7	1461.4	1360.9	1285.3	1630.8	1450.7	1536.3
s-8	550.8	1523.8	560.7	1706.0	618.8	919.2
s-9	1788.5	1870.4	1717.0	2227.4	1761.5	2616.1
s-10	2545.4	2363.8	2274.5	2702.1	2709.2	2675.5
s-11	1183.7	1344.6	1100.9	2648.1	1369.7	2659.4
s-12	400.0	566.2	375.9	1292.8	448.7	1732.1

Table S5.4: **$\hat{\mathbf{R}}$ diagnostics for the SEIR model with sparse data.**

**Dataset**	$\boldsymbol{\gamma^{-1}}$	$\boldsymbol{\kappa^{-1}}$	$\boldsymbol{\beta}$	**s**	$\mathbf{t}_{\text{var}}$	$\mathbf{I_0}$
s-1-1-1	1.064	1.034	1.065	1.052	1.002	1.005
s-1-1-2	1.154	1.003	1.143	1.099	1.008	1.028
s-1-1-3	1.100	1.019	1.100	1.085	1.018	1.007
s-1-2-1	1.112	1.007	1.109	1.076	1.036	1.030
s-1-2-2	1.067	1.016	1.068	1.055	1.024	1.009
s-1-2-3	1.027	1.007	1.028	1.016	1.011	1.007
s-3	1.047	1.002	1.046	1.006	1.035	1.009
s-4	1.005	1.006	1.005	1.002	1.005	1.004
s-5	1.026	1.003	1.026	1.005	1.012	1.005
s-6	1.008	1.005	1.007	1.003	1.007	1.002
s-7	1.005	1.013	1.010	1.003	1.004	1.007
s-8	1.068	1.007	1.067	1.011	1.053	1.022
s-9	1.003	1.004	1.003	1.005	1.003	1.002
s-10	1.004	1.003	1.003	1.002	1.003	1.004
s-11	1.015	1.011	1.015	1.003	1.013	1.003
s-12	1.083	1.052	1.084	1.006	1.065	1.004
