# Supplementary material for: Assessment of simulation-based inference methods for stochastic compartmental models in epidemiological research
Source: PLoS One. 2026 Jul 13;21(7):e0353306. doi: 10.1371/journal.pone.0353306 (PMC13362117; doi:10.1371/journal.pone.0353306)
Supplement: S6 Results — (PDF) [file pone.0353306.s006.pdf]

S6 Supplementary Results Reparametrized SEIR-Model

Assessment of Simulation-based Inference Methods for Stochastic Compartmental Models in Epidemiological Research

Vincent Wieland[1,2,◑], Nils Waßmuth[1,2,3,◑], Lorenzo Contento[1], Martin Kühn[1,2,3], and Jan Hasenauer[1,2,*]

[1]Bonn Center for Mathematical Life Sciences, University of Bonn, Bonn, Germany
[2]Life and Medical Science Institute, University of Bonn, Bonn, Germany
[3]Institute of Software Technology, Department for High-Performance Computing, German Aerospace Center (DLR), Cologne, Germany
[◑]These authors contributed equally to the work.
[*]To whom correspondence should be addressed; jan.hasenauer@uni-bonn.de.

June 26, 2026

Contents

S6.A Supplementary Figures . . . 2
S6.B Supplementary Tables . . . 20

S6.A Supplementary Figures

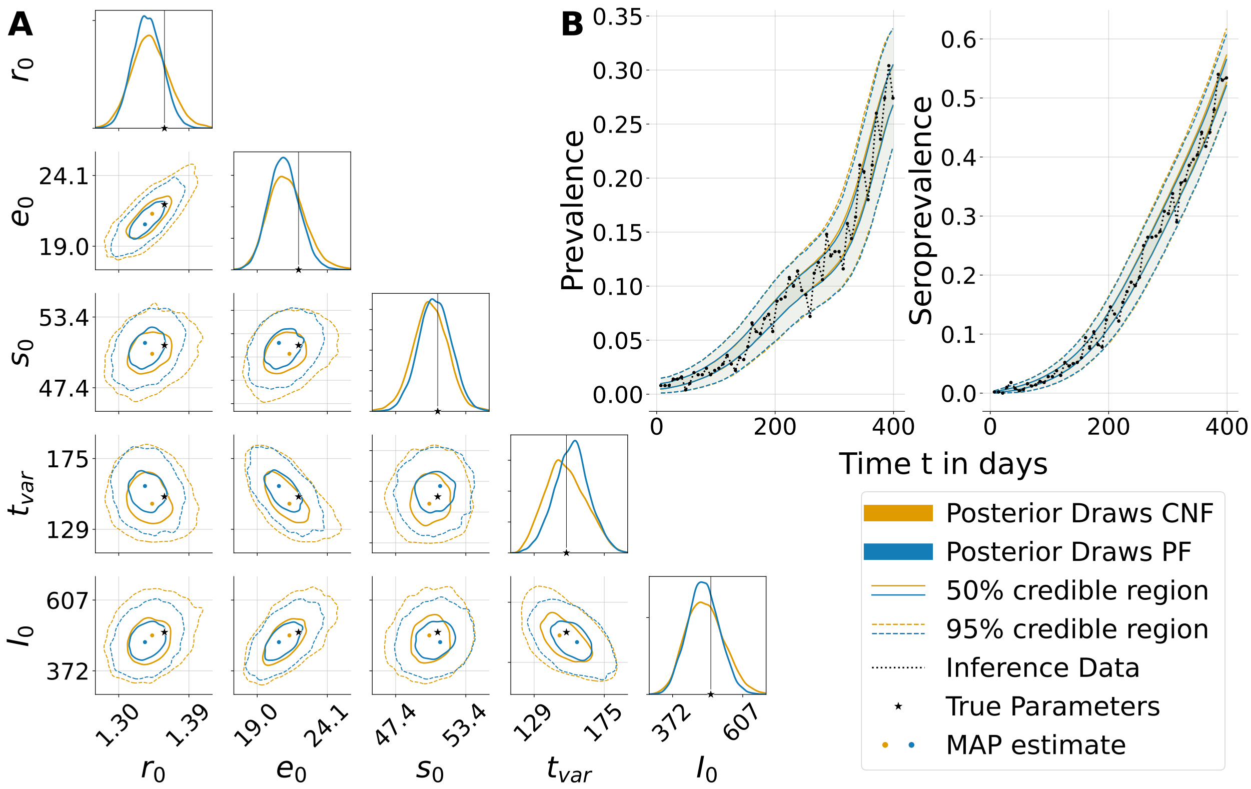

Figure S6.1: **Results of the reparametrized two-variant SEIR model for *d-1-1*.**
A Posterior approximations from 10,000 samples. Contour gives the 50% (solid) and 95% (dashed) credible regions, coloured by method. Diagonals show the 1D marginals. Black stars mark the true parameters, coloured circles the joint MAP estimates. **B** Posterior predictive fit: bands give the 50% and 95% pointwise predictive intervals from the same samples (line styles as in **A**) with inference data shown as a dotted line.

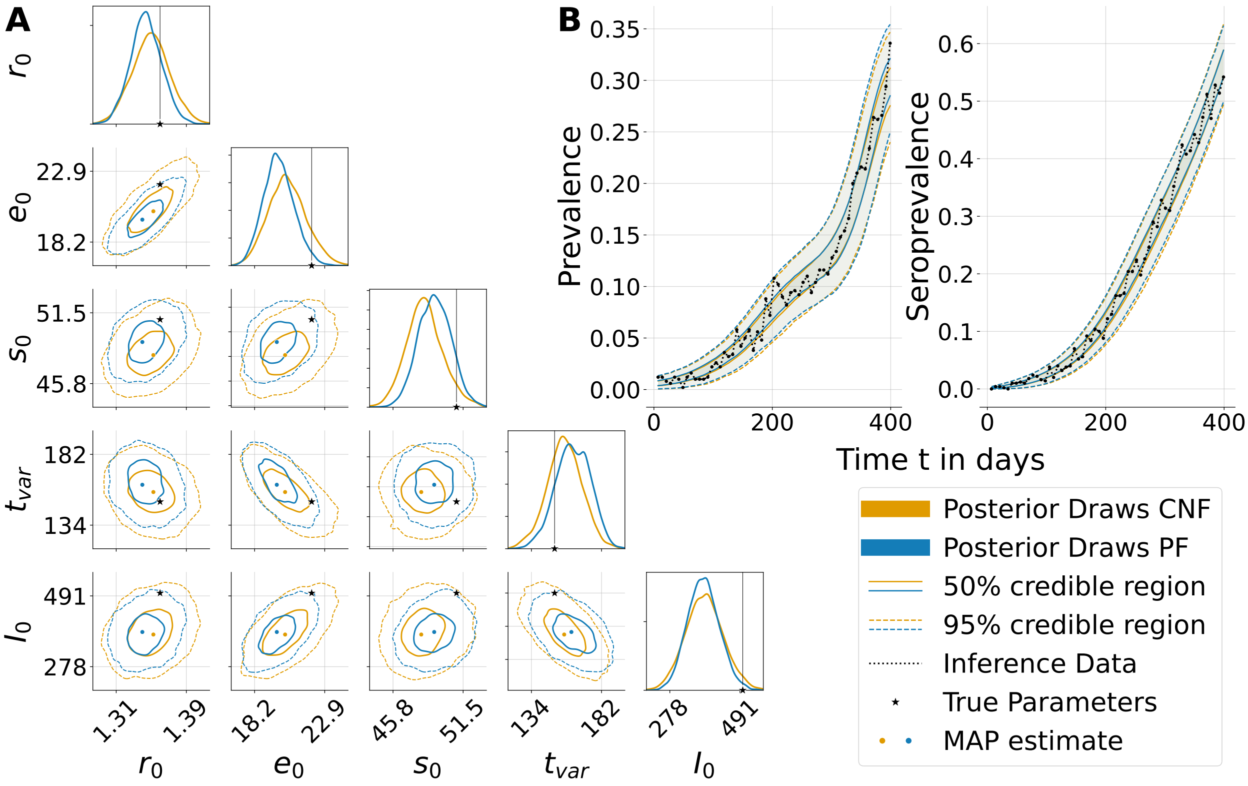

Figure S6.2: **Results of the reparametrized two-variant SEIR model for *d-1-2*.**
A Posterior approximations from 10,000 samples. Contour gives the 50% (solid) and 95% (dashed) credible regions, coloured by method. Diagonals show the 1D marginals. Black stars mark the true parameters, coloured circles the joint MAP estimates. **B** Posterior predictive fit: bands give the 50% and 95% pointwise predictive intervals from the same samples (line styles as in **A**) with inference data shown as a dotted line.

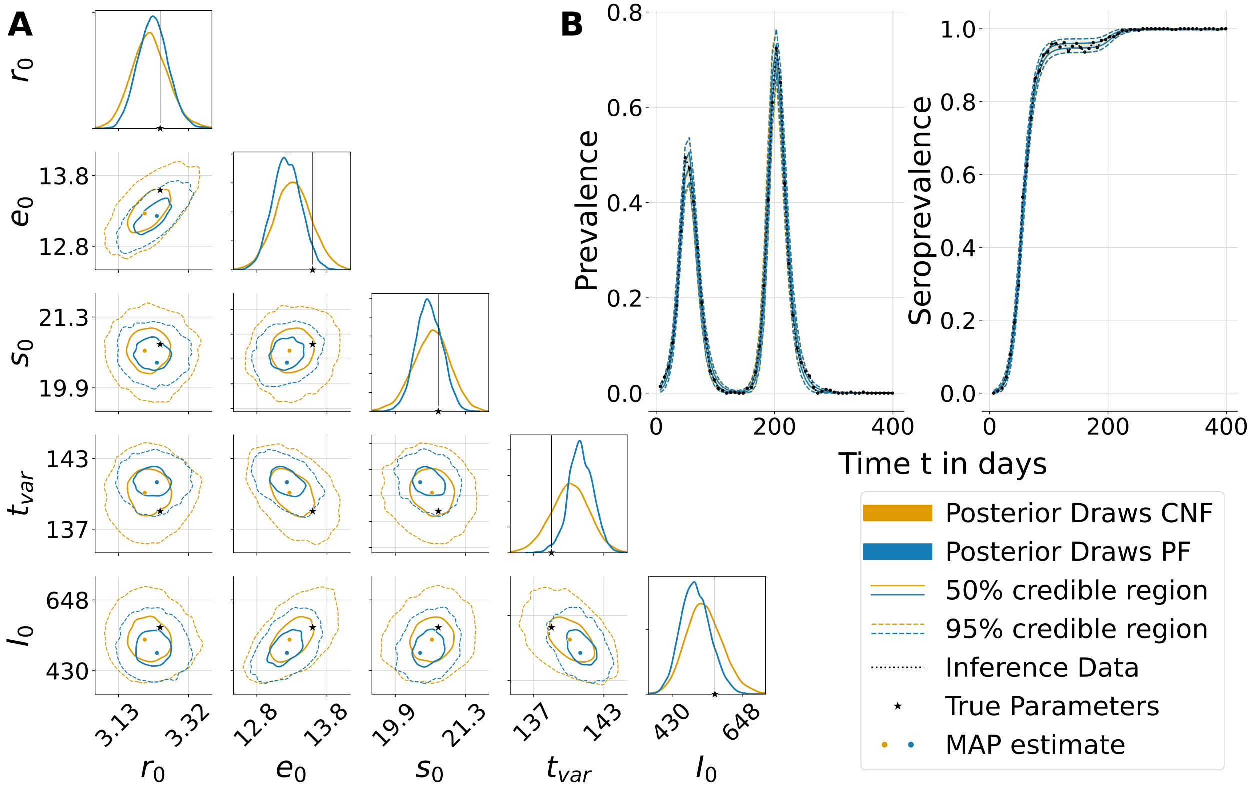

Figure S6.3: **Results of the reparametrized two-variant SEIR model for *r-1*.**
A Posterior approximations from 10,000 samples. Contour gives the 50% (solid) and 95% (dashed) credible regions, coloured by method. Diagonals show the 1D marginals. Black stars mark the true parameters, coloured circles the joint MAP estimates. **B** Posterior predictive fit: bands give the 50% and 95% pointwise predictive intervals from the same samples (line styles as in **A**) with inference data shown as a dotted line.

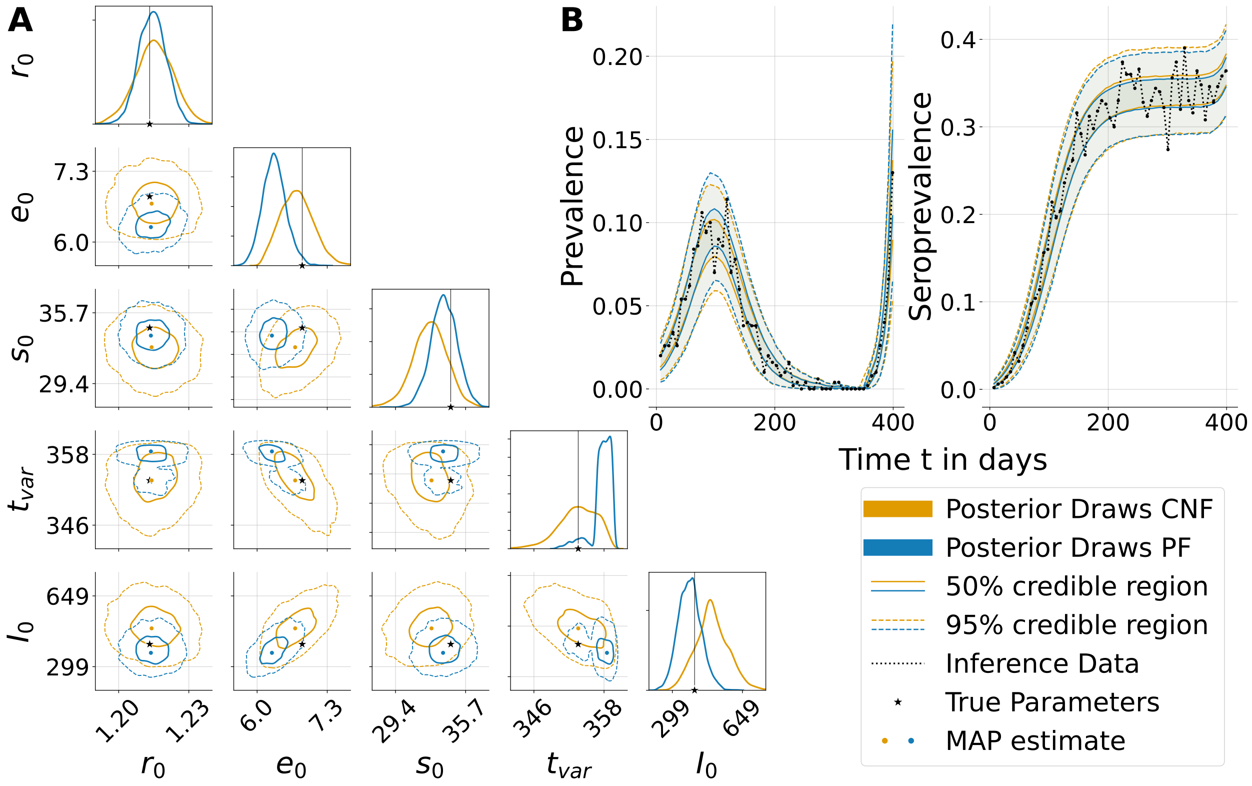

Figure S6.4: **Results of the reparametrized two-variant SEIR model for *r-2*.**
A Posterior approximations from 10,000 samples. Contour gives the 50% (solid) and 95% (dashed) credible regions, coloured by method. Diagonals show the 1D marginals. Black stars mark the true parameters, coloured circles the joint MAP estimates. **B** Posterior predictive fit: bands give the 50% and 95% pointwise predictive intervals from the same samples (line styles as in **A**) with inference data shown as a dotted line.

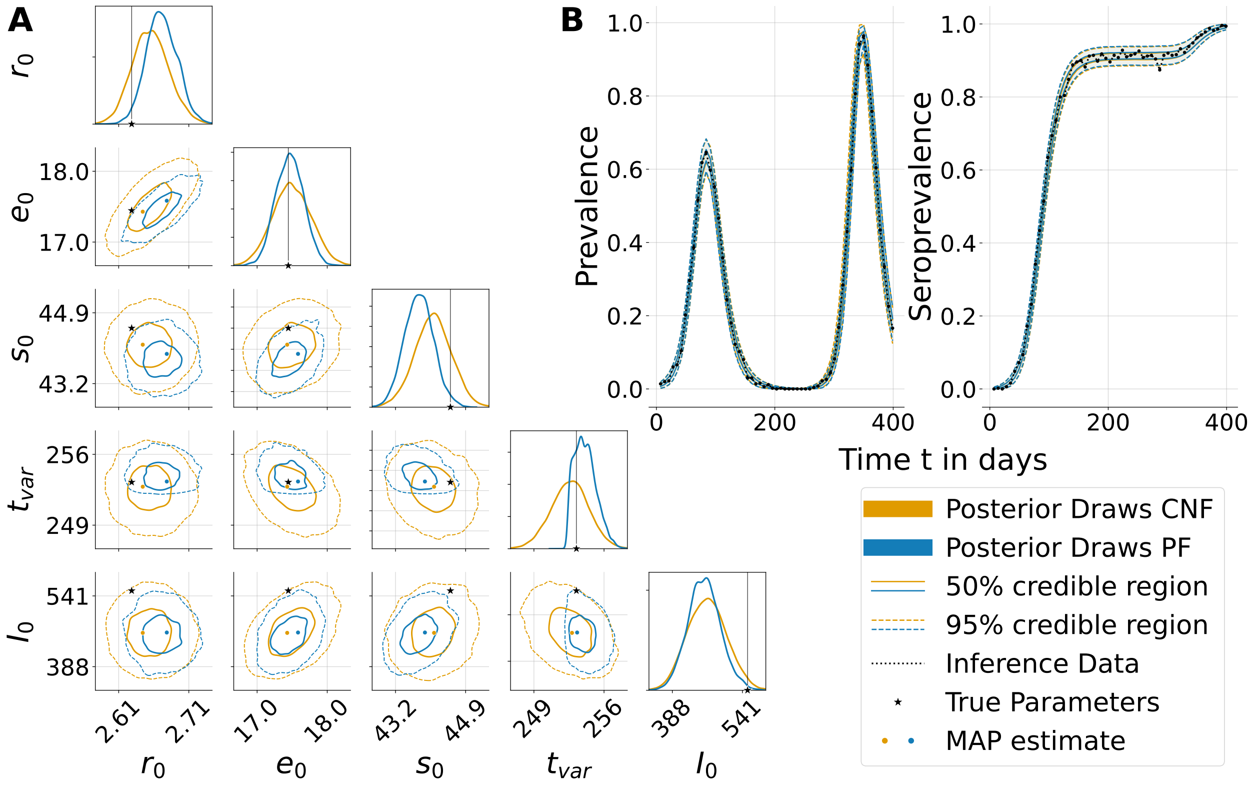

Figure S6.5: **Results of the reparametrized two-variant SEIR model for *r-3*.**
A Posterior approximations from 10,000 samples. Contour gives the 50% (solid) and 95% (dashed) credible regions, coloured by method. Diagonals show the 1D marginals. Black stars mark the true parameters, coloured circles the joint MAP estimates. **B** Posterior predictive fit: bands give the 50% and 95% pointwise predictive intervals from the same samples (line styles as in **A**) with inference data shown as a dotted line.

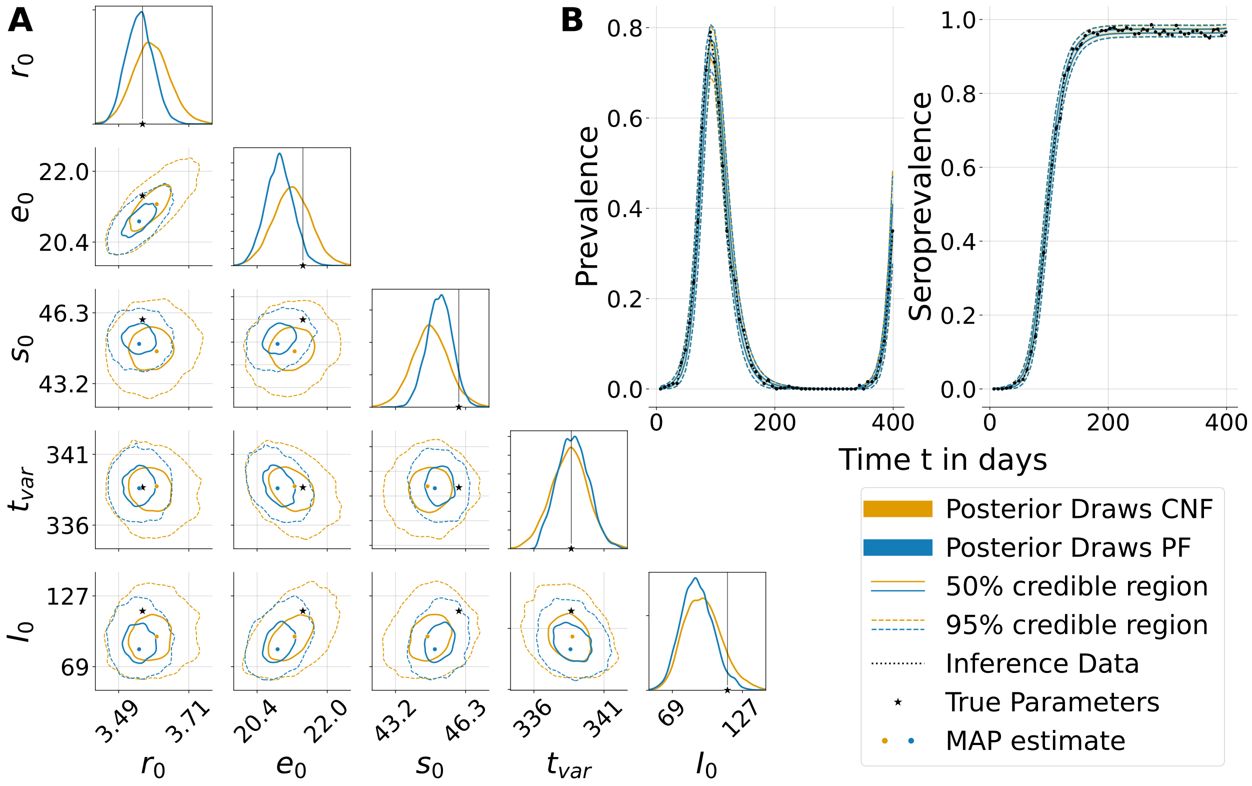

Figure S6.6: **Results of the reparametrized two-variant SEIR model for r-4.**
A Posterior approximations from 10,000 samples. Contour gives the 50% (solid) and 95% (dashed) credible regions, coloured by method. Diagonals show the 1D marginals. Black stars mark the true parameters, coloured circles the joint MAP estimates. **B** Posterior predictive fit: bands give the 50% and 95% pointwise predictive intervals from the same samples (line styles as in **A**) with inference data shown as a dotted line.

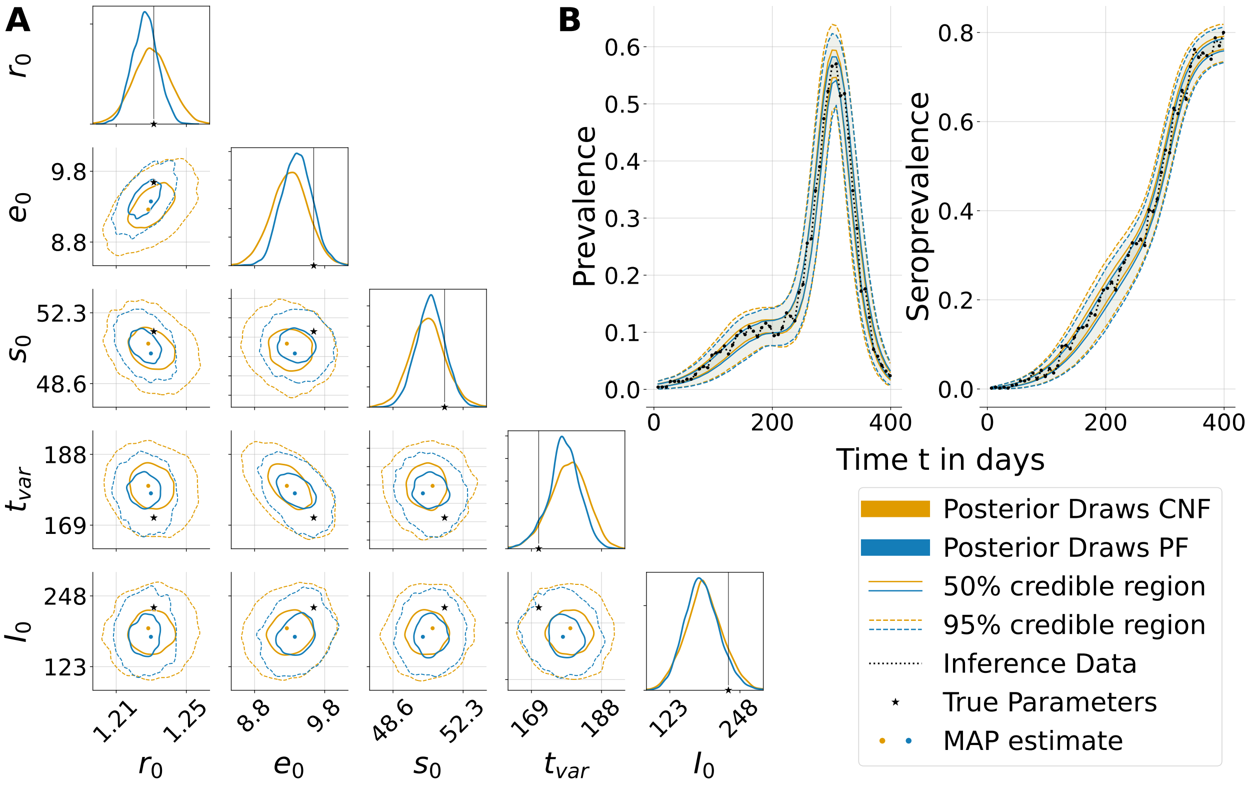

Figure S6.7: **Results of the reparametrized two-variant SEIR model for *r-5*.**
A Posterior approximations from 10,000 samples. Contour gives the 50% (solid) and 95% (dashed) credible regions, coloured by method. Diagonals show the 1D marginals. Black stars mark the true parameters, coloured circles the joint MAP estimates. **B** Posterior predictive fit: bands give the 50% and 95% pointwise predictive intervals from the same samples (line styles as in **A**) with inference data shown as a dotted line.

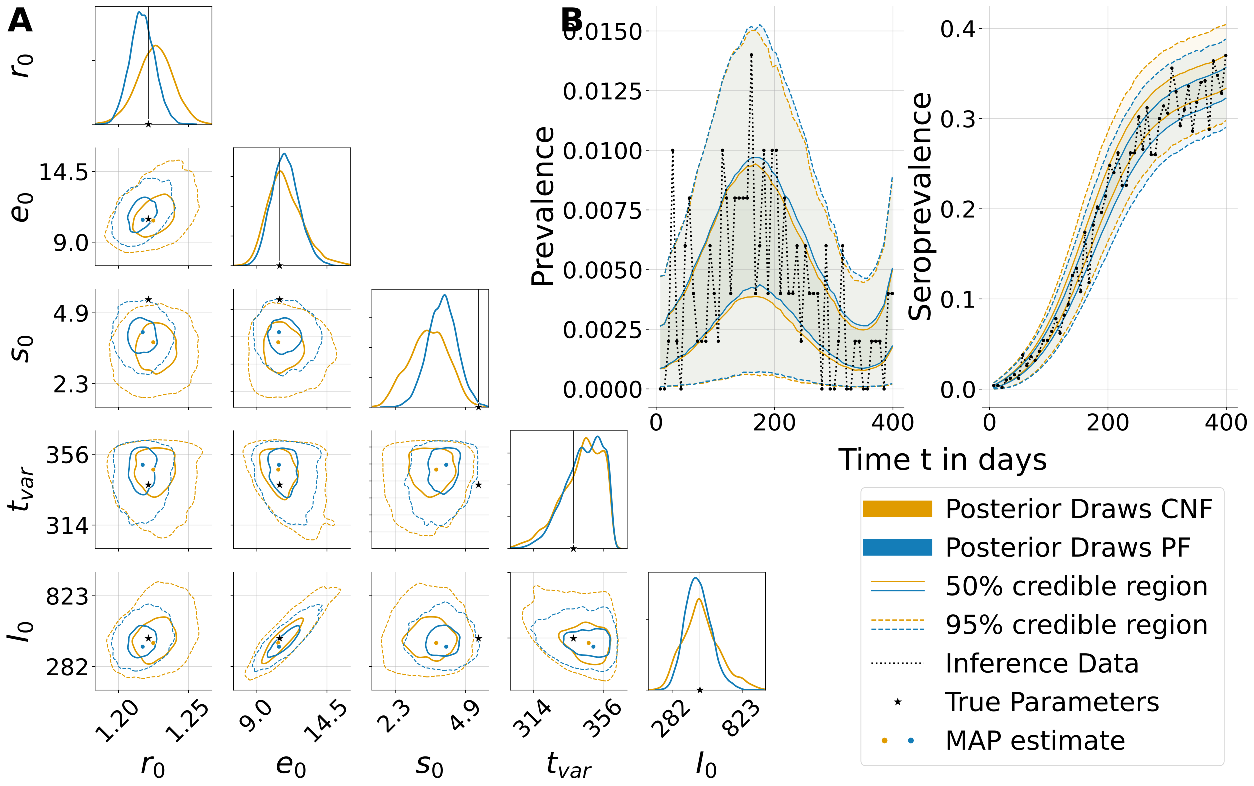

Figure S6.8: **Results of the reparametrized two-variant SEIR model for *r-6*.**
A Posterior approximations from 10,000 samples. Contour gives the 50% (solid) and 95% (dashed) credible regions, coloured by method. Diagonals show the 1D marginals. Black stars mark the true parameters, coloured circles the joint MAP estimates. **B** Posterior predictive fit: bands give the 50% and 95% pointwise predictive intervals from the same samples (line styles as in **A**) with inference data shown as a dotted line.

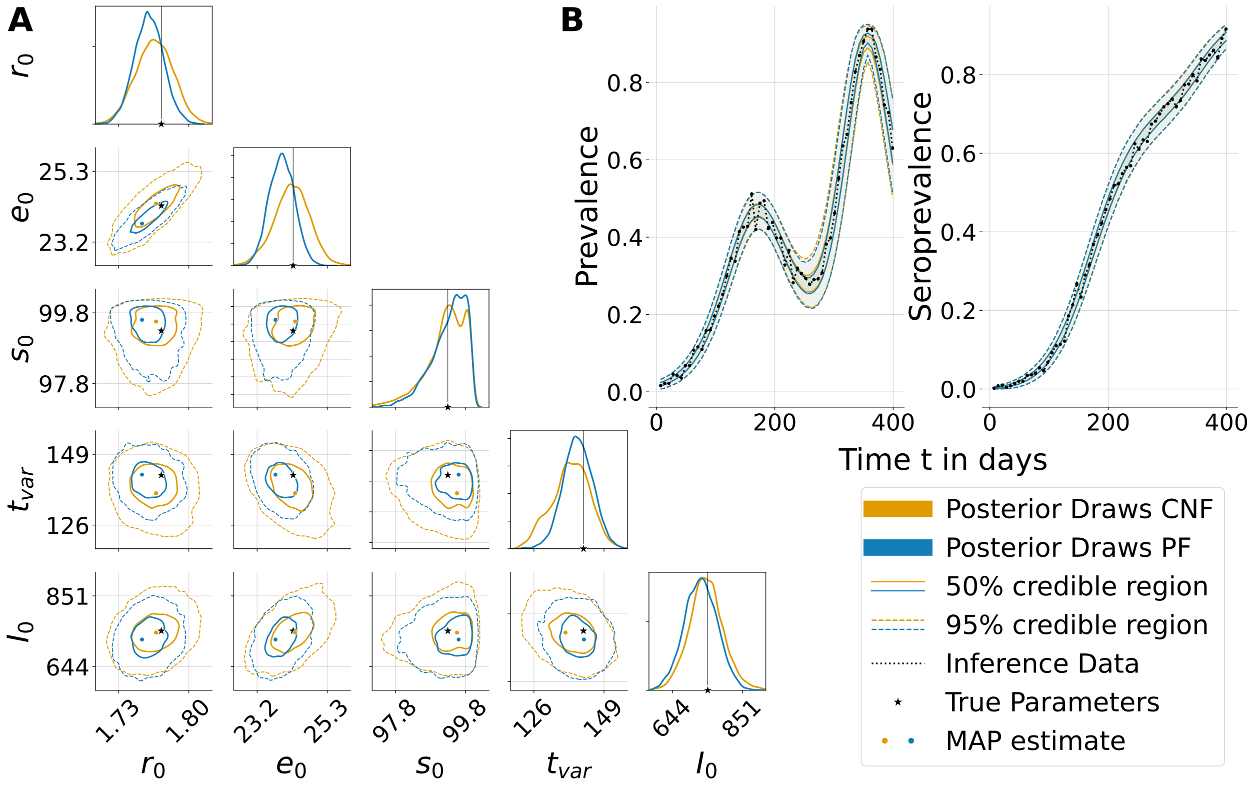

Figure S6.9: **Results of the reparametrized two-variant SEIR model for *r-7*.**
A Posterior approximations from 10,000 samples. Contour gives the 50% (solid) and 95% (dashed) credible regions, coloured by method. Diagonals show the 1D marginals. Black stars mark the true parameters, coloured circles the joint MAP estimates. **B** Posterior predictive fit: bands give the 50% and 95% pointwise predictive intervals from the same samples (line styles as in **A**) with inference data shown as a dotted line.

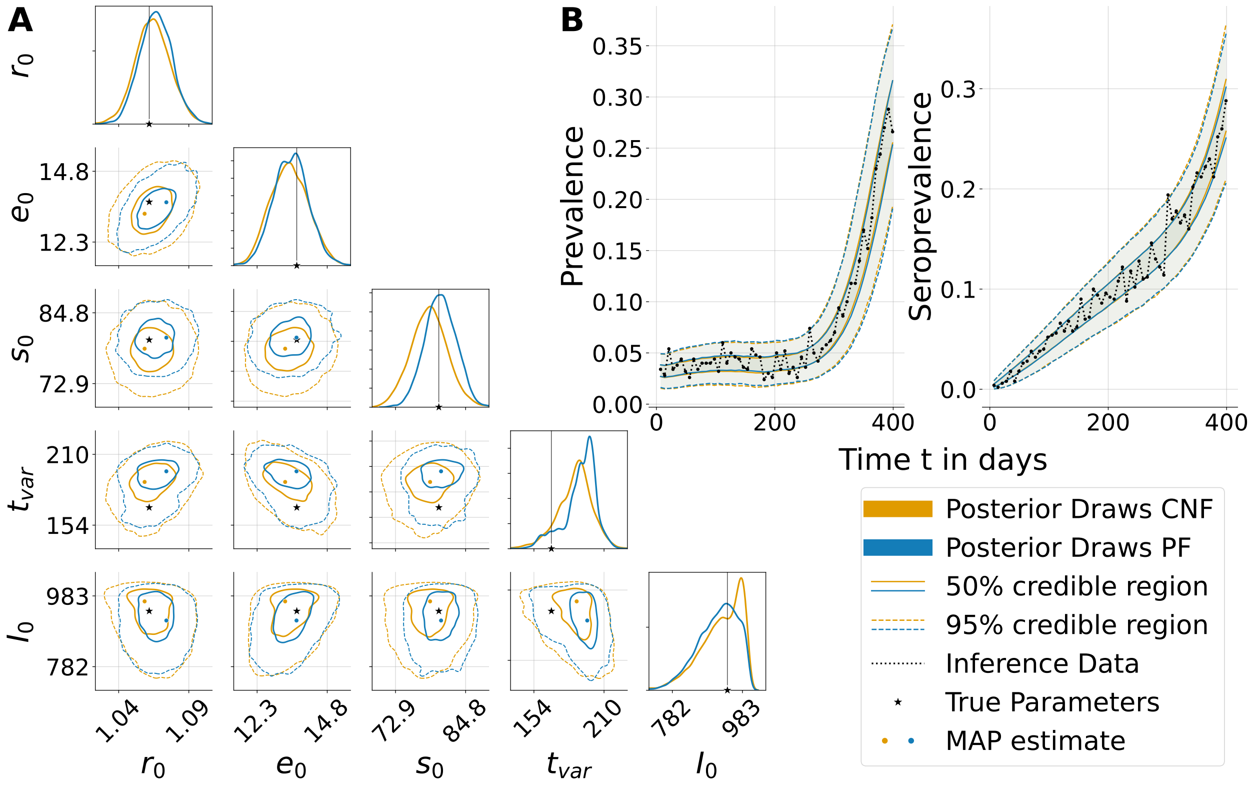

Figure S6.10: **Results of the reparametrized two-variant SEIR model for r-8.**
A Posterior approximations from 10,000 samples. Contour gives the 50% (solid) and 95% (dashed) credible regions, coloured by method. Diagonals show the 1D marginals. Black stars mark the true parameters, coloured circles the joint MAP estimates. **B** Posterior predictive fit: bands give the 50% and 95% pointwise predictive intervals from the same samples (line styles as in **A**) with inference data shown as a dotted line.

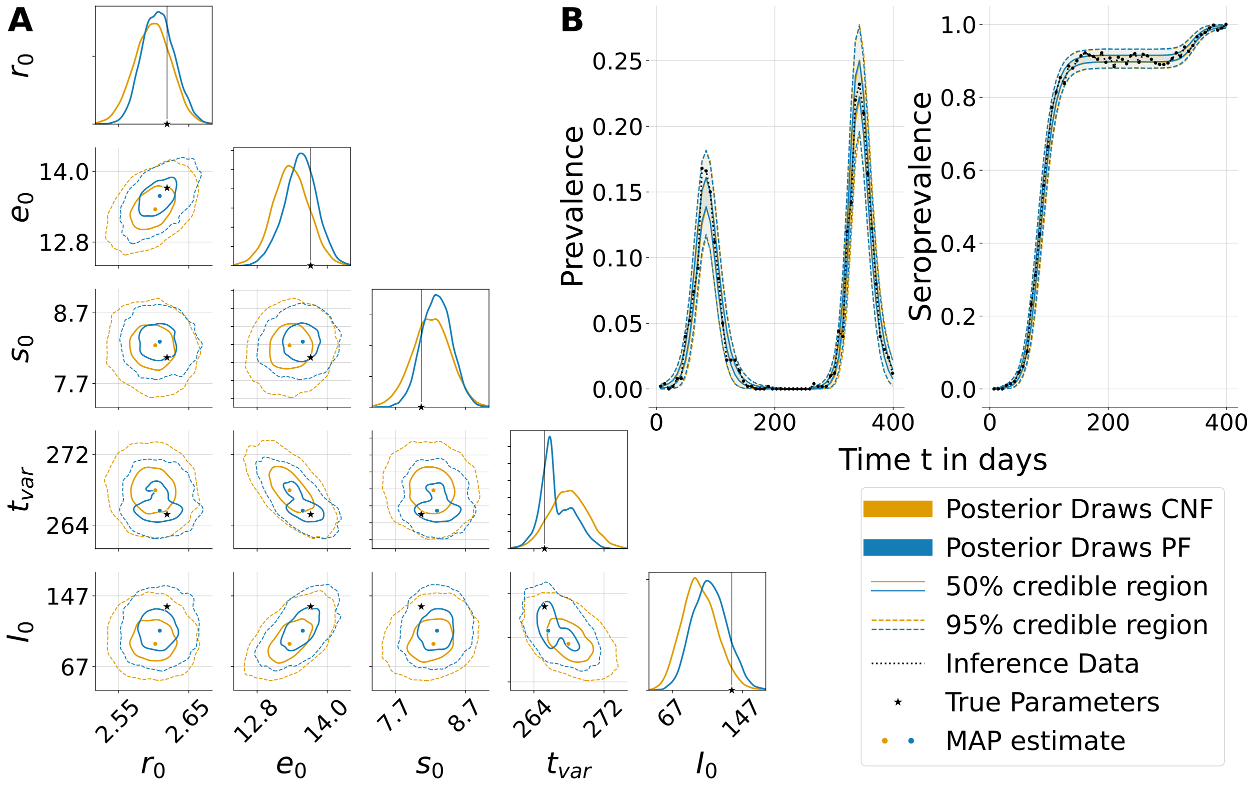

Figure S6.11: **Results of the reparametrized two-variant SEIR model for *r-9*.**
A Posterior approximations from 10,000 samples. Contour gives the 50% (solid) and 95% (dashed) credible regions, coloured by method. Diagonals show the 1D marginals. Black stars mark the true parameters, coloured circles the joint MAP estimates. **B** Posterior predictive fit: bands give the 50% and 95% pointwise predictive intervals from the same samples (line styles as in **A**) with inference data shown as a dotted line.

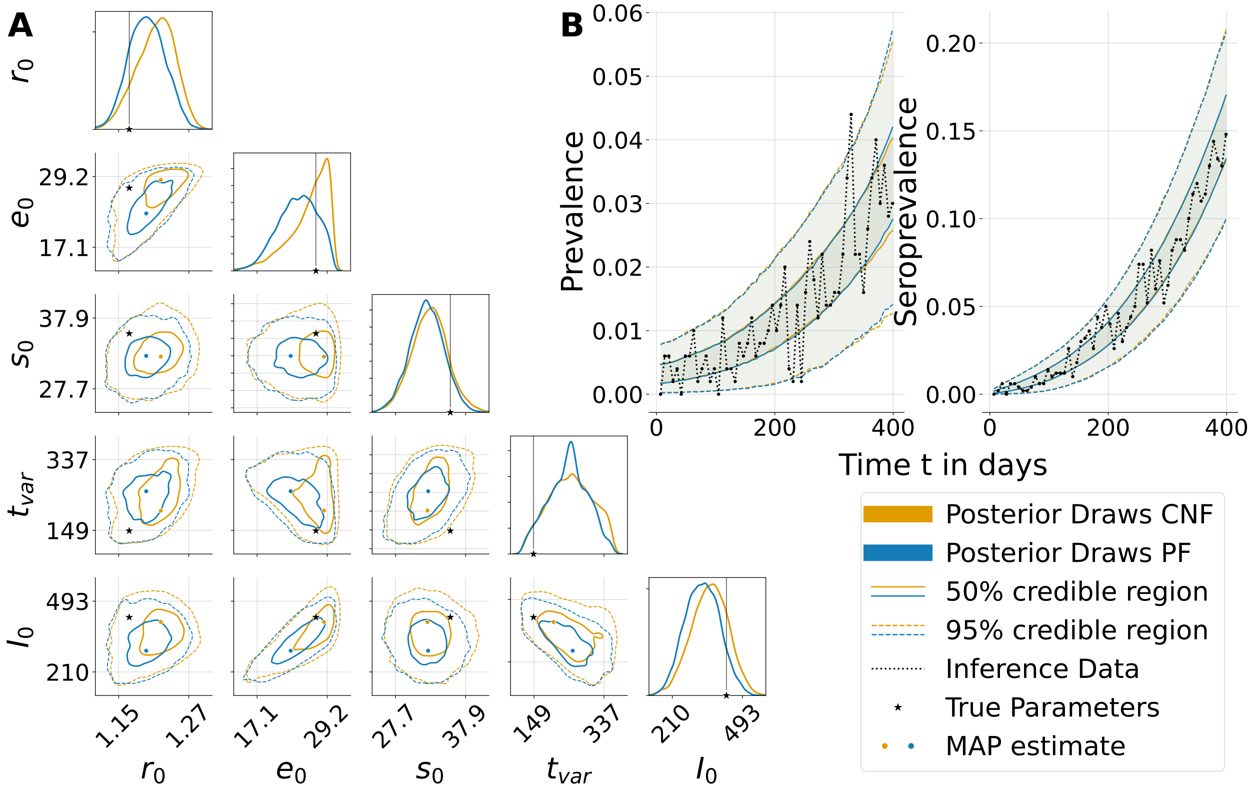

Figure S6.12: **Results of the reparametrized two-variant SEIR model for *r-10*.**
A Posterior approximations from 10,000 samples. Contour gives the 50% (solid) and 95% (dashed) credible regions, coloured by method. Diagonals show the 1D marginals. Black stars mark the true parameters, coloured circles the joint MAP estimates. **B** Posterior predictive fit: bands give the 50% and 95% pointwise predictive intervals from the same samples (line styles as in **A**) with inference data shown as a dotted line.

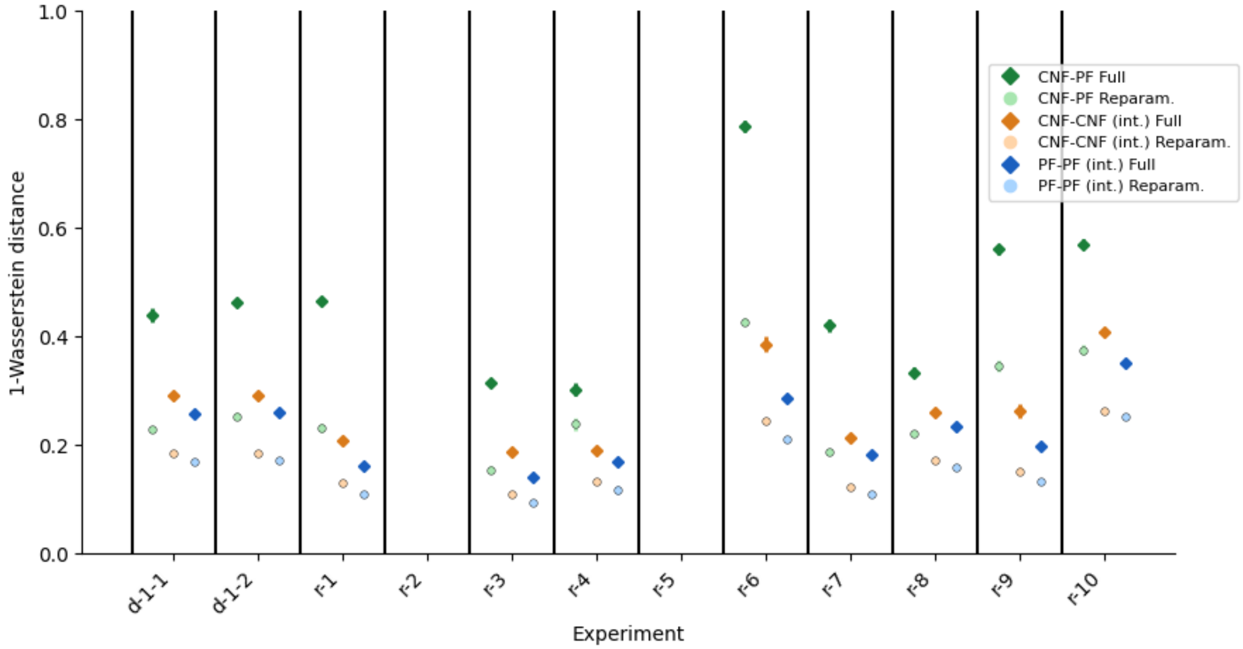

Figure S6.13: **Pairwise 1-Wasserstein distances for Full and Reparametrized variants of the SEIR2V model across CNF-PF, CNF-CNF (interior), and PF–PF (interior).** The plot shows how reparametrization affects Wasserstein distances across experiments. Interior distances quantify within-run Monte-Carlo variability.

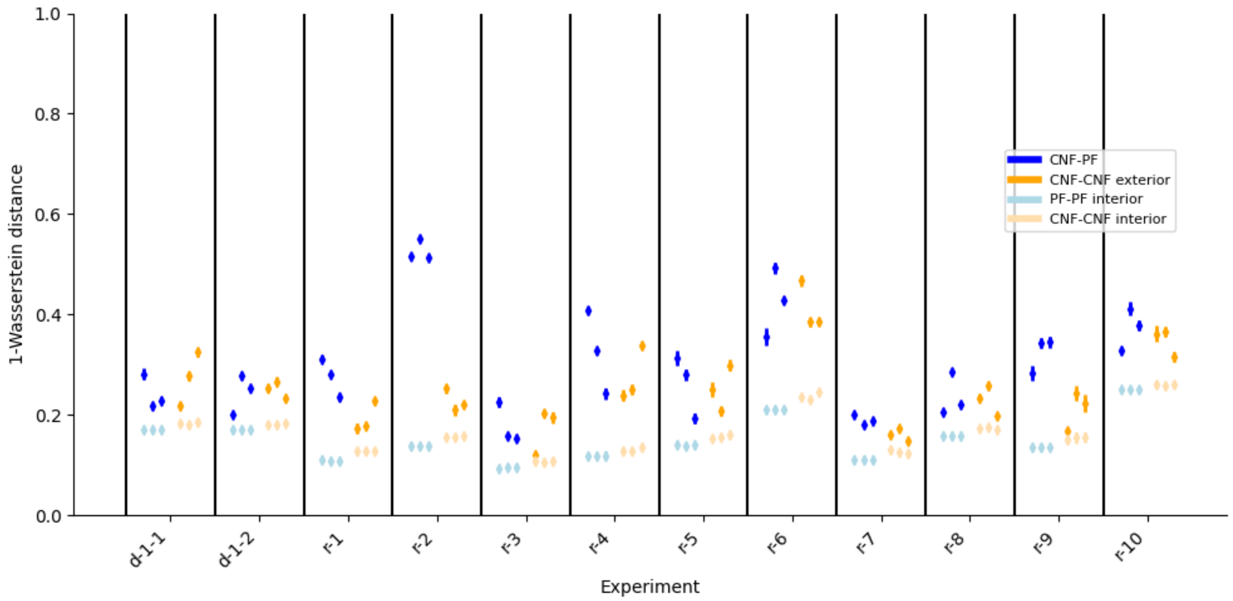

Figure S6.14: **Pairwise 1-Wasserstein distances between PF and CNF with multiple CNF reruns for the reparametrized two-variant model** Interior distances quantify within-run Monte-Carlo variability, while exterior distances capture variability across repeated CNF runs.

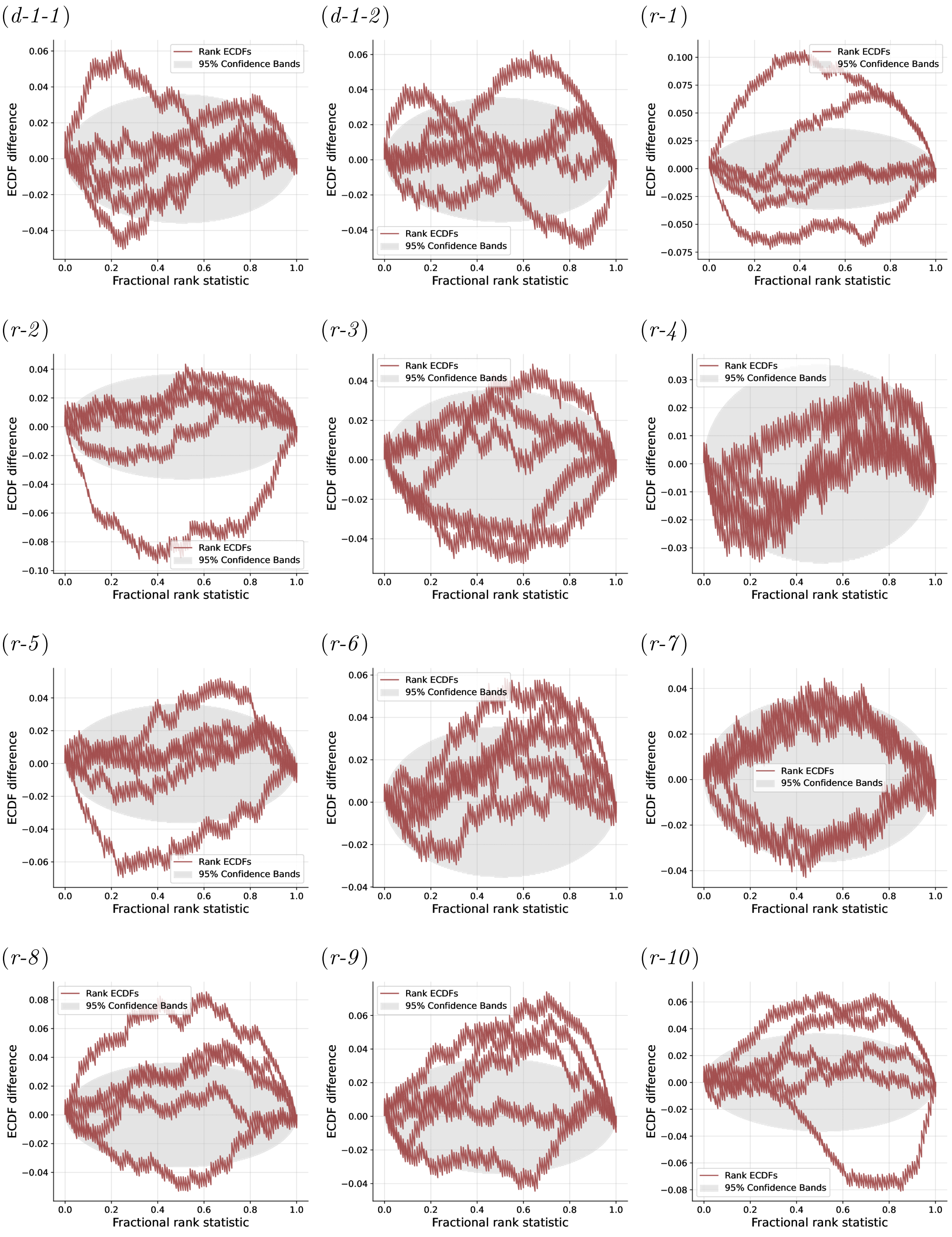

Figure S6.15: **ECDF Calibration plots for the reparametrized SEIR model and dense datasets.**

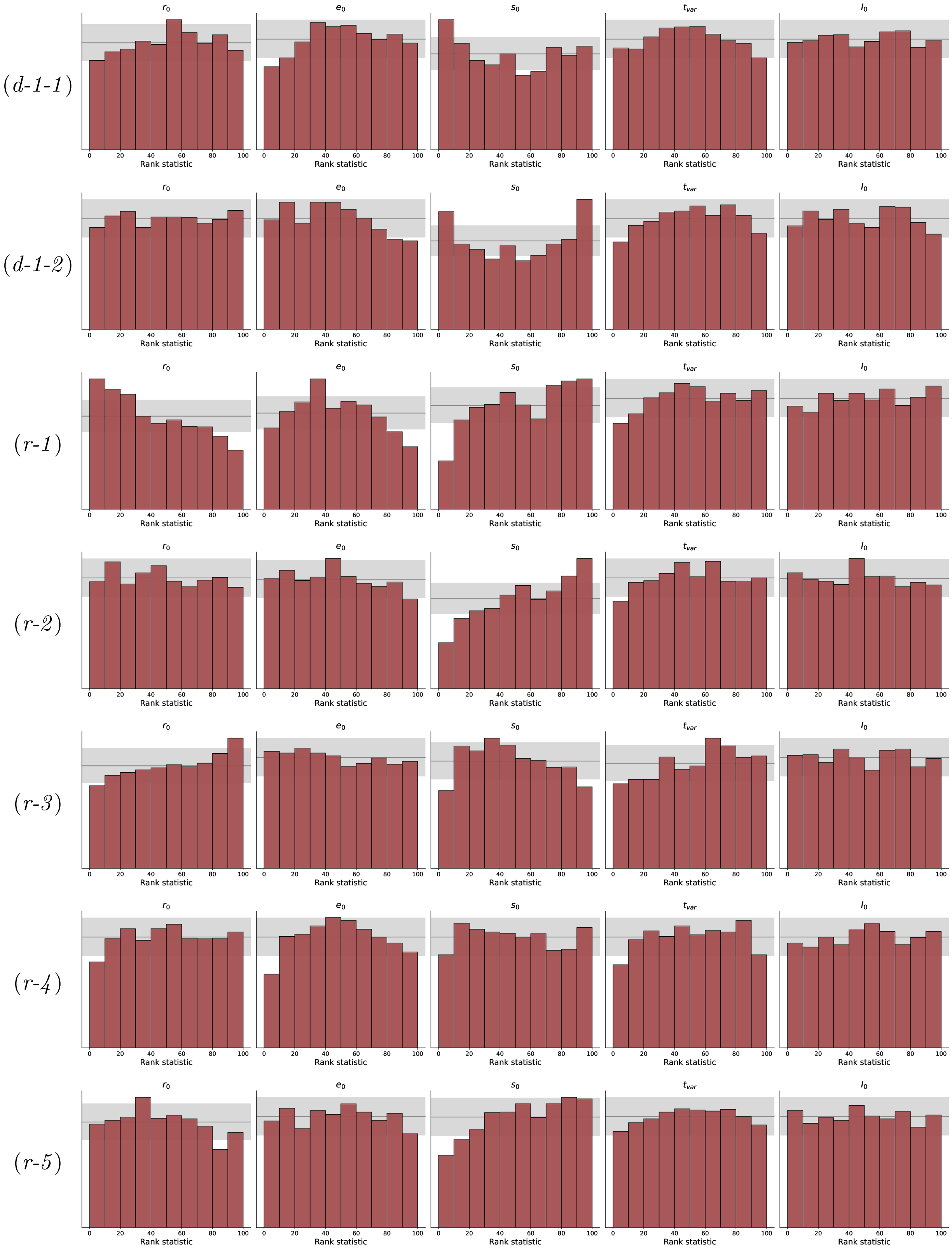

(d-1-1)
(d-1-2)
(r-1)
(r-2)
(r-3)
(r-4)
(r-5)
r_0
e_0
s_0
t_{var}
I_0
Rank statistic

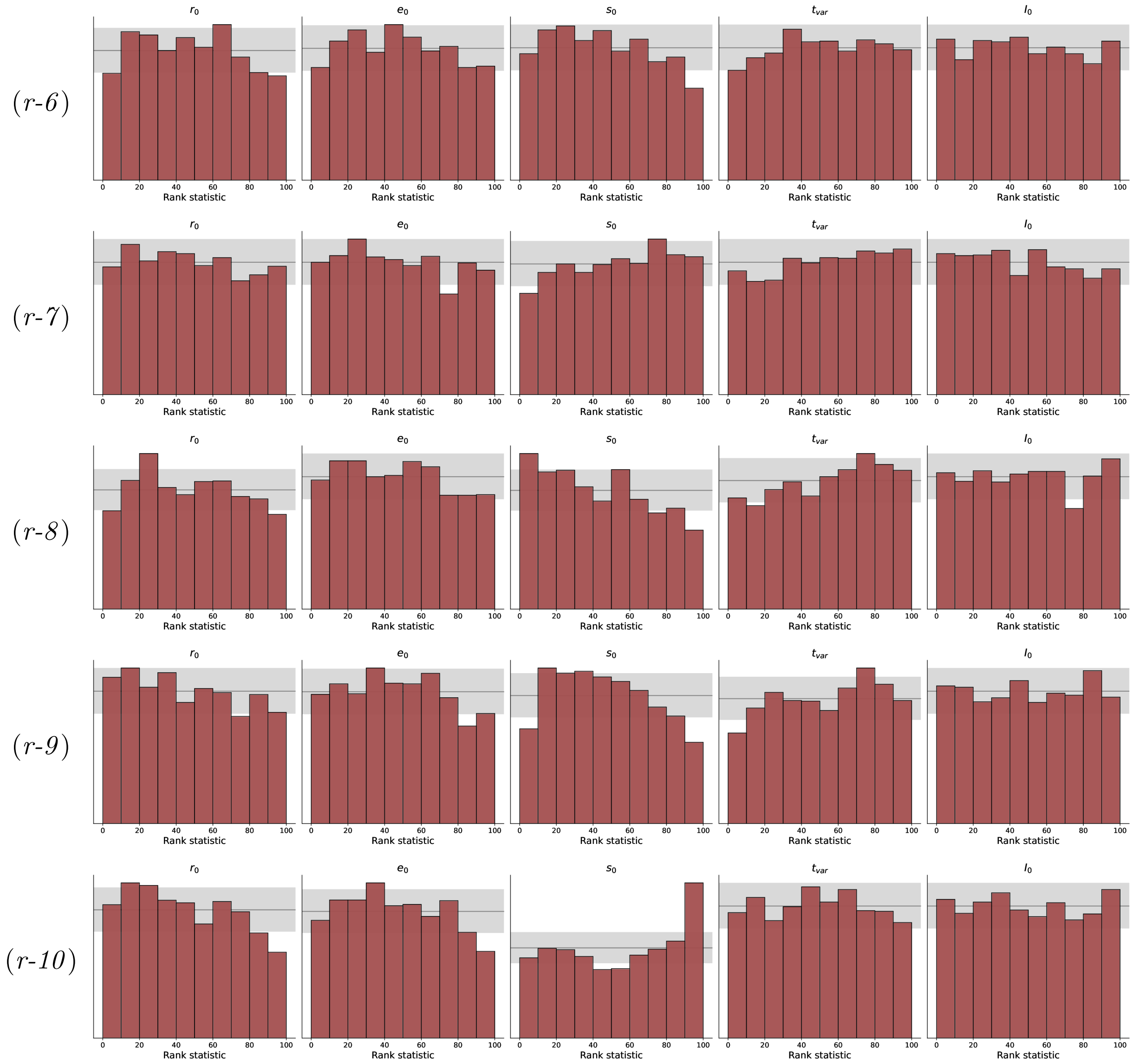

Figure S6.15: **SBC Histograms for the reparametrized SEIR model and dense datasets.**

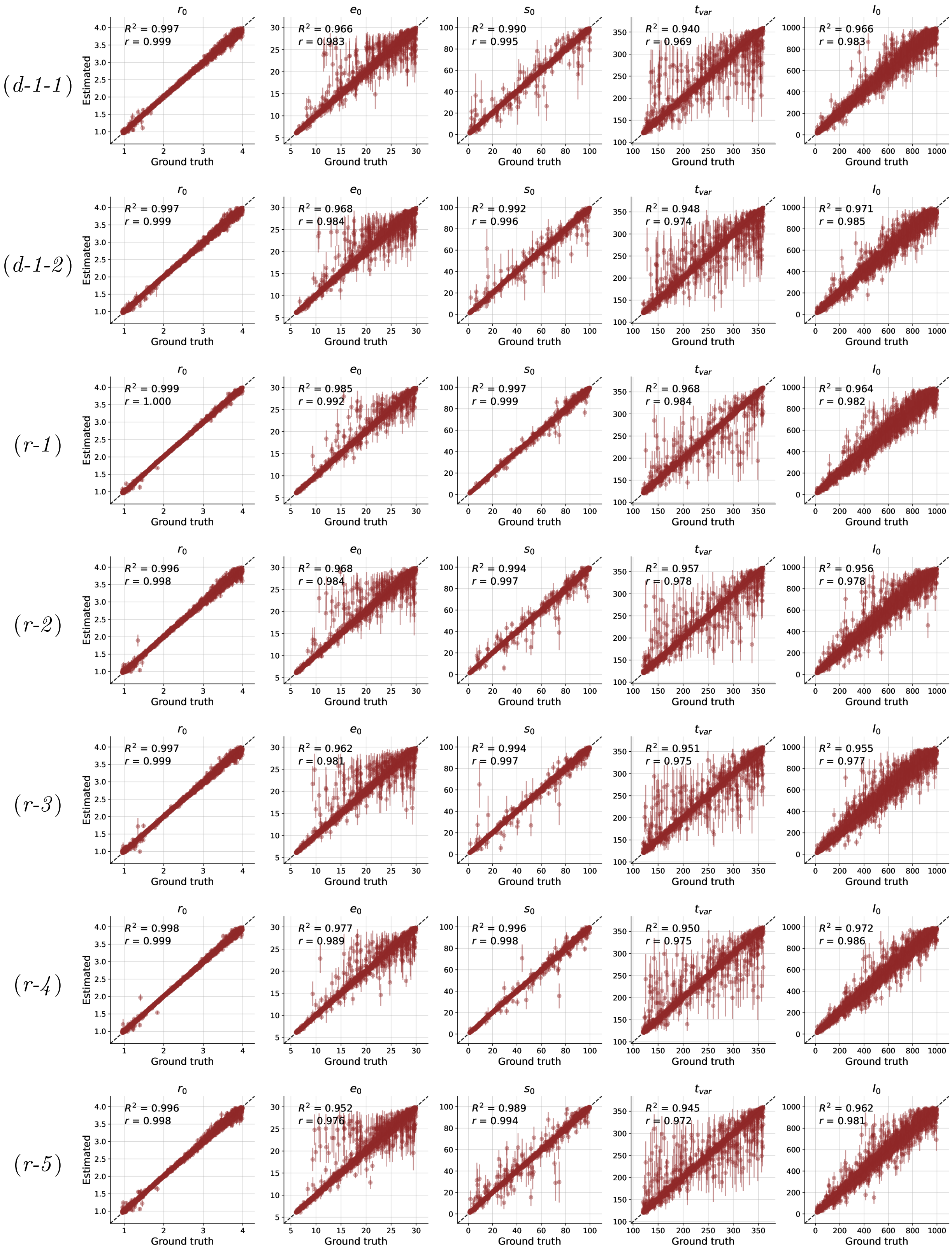
(d-1-1)
(d-1-2)
(r-1)
(r-2)
(r-3)
(r-4)
(r-5)
r_0
e_0
s_0
t_{var}
I_0
Estimated
Ground truth
(d-1-1) r_0: R^2 = 0.997, r = 0.999; e_0: R^2 = 0.966, r = 0.983; s_0: R^2 = 0.990, r = 0.995; t_{var}: R^2 = 0.940, r = 0.969; I_0: R^2 = 0.966, r = 0.983
(d-1-2) r_0: R^2 = 0.997, r = 0.999; e_0: R^2 = 0.968, r = 0.984; s_0: R^2 = 0.992, r = 0.996; t_{var}: R^2 = 0.948, r = 0.974; I_0: R^2 = 0.971, r = 0.985
(r-1) r_0: R^2 = 0.999, r = 1.000; e_0: R^2 = 0.985, r = 0.992; s_0: R^2 = 0.997, r = 0.999; t_{var}: R^2 = 0.968, r = 0.984; I_0: R^2 = 0.964, r = 0.982
(r-2) r_0: R^2 = 0.996, r = 0.998; e_0: R^2 = 0.968, r = 0.984; s_0: R^2 = 0.994, r = 0.997; t_{var}: R^2 = 0.957, r = 0.978; I_0: R^2 = 0.956, r = 0.978
(r-3) r_0: R^2 = 0.997, r = 0.999; e_0: R^2 = 0.962, r = 0.981; s_0: R^2 = 0.994, r = 0.997; t_{var}: R^2 = 0.951, r = 0.975; I_0: R^2 = 0.955, r = 0.977
(r-4) r_0: R^2 = 0.998, r = 0.999; e_0: R^2 = 0.977, r = 0.989; s_0: R^2 = 0.996, r = 0.998; t_{var}: R^2 = 0.950, r = 0.975; I_0: R^2 = 0.972, r = 0.986
(r-5) r_0: R^2 = 0.996, r = 0.998; e_0: R^2 = 0.952, r = 0.976; s_0: R^2 = 0.989, r = 0.994; t_{var}: R^2 = 0.945, r = 0.972; I_0: R^2 = 0.962, r = 0.981

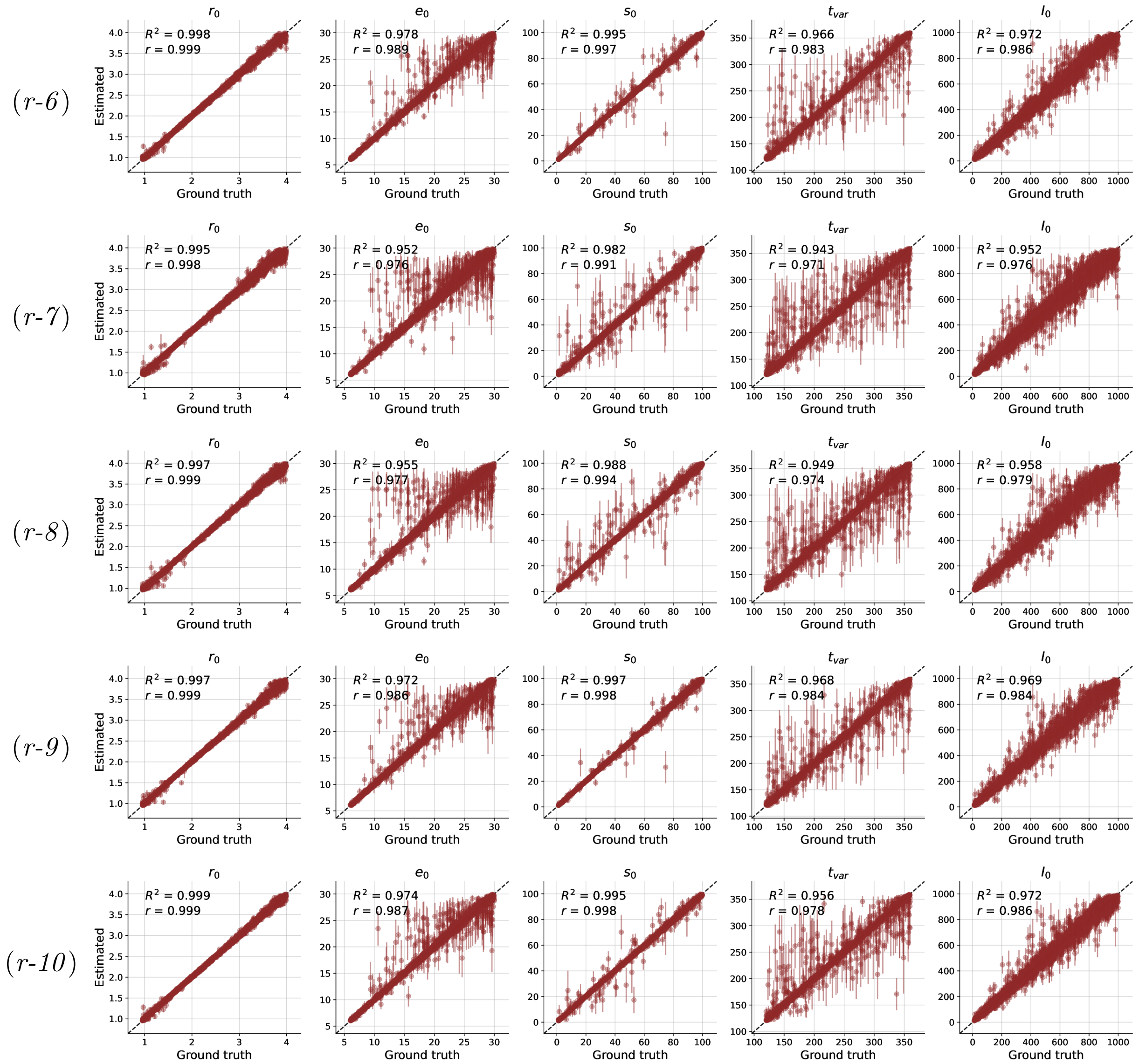

Figure S6.15: **Parameter recovery for the reparametrized SEIR model and dense datasets.**

S6.B Supplementary Tables

Dataset	Method	r_0	e_0	s_0	t_{var}	I_0
d-1-1	True	1.360	22.00	51.00	150.0	500.0
	CNF	1.344 (1.297, 1.391)	21.33 (18.79, 24.09)	50.27 (47.37, 53.36)	145.5 (126.2, 175.8)	489.7 (369.2, 612.6)
	PF	1.335 (1.303, 1.375)	20.57 (18.88, 23.19)	51.21 (48.01, 53.47)	156.9 (131.0, 176.0)	467.3 (374.5, 580.7)
d-1-2	True	1.360	22.00	51.00	150.0	500.0
	CNF	1.352 (1.306, 1.393)	20.24 (18.02, 23.00)	48.11 (45.53, 51.62)	156.5 (132.2, 179.6)	375.1 (273.7, 500.8)
	PF	1.339 (1.308, 1.380)	19.69 (17.94, 21.93)	49.17 (46.62, 51.86)	161.3 (138.9, 183.6)	382.7 (290.3, 478.9)
r-1	True	3.239	13.63	20.80	138.0	563.0
	CNF	3.197 (3.117, 3.316)	13.30 (12.84, 13.87)	20.67 (19.87, 21.36)	140.2 (137.2, 143.1)	525.6 (423.9, 652.5)
	PF	3.230 (3.142, 3.303)	13.27 (12.88, 13.66)	20.43 (20.08, 21.12)	141.0 (139.1, 142.9)	484.5 (417.5, 600.2)
r-2	True	1.213	6.83	34.36	353.0	410.5
	CNF	1.215 (1.196, 1.235)	6.70 (6.19, 7.38)	32.64 (29.24, 35.42)	353.9 (346.2, 359.0)	488.8 (344.8, 667.2)
	PF	1.214 (1.201, 1.229)	6.27 (5.91, 6.78)	33.67 (31.39, 36.07)	358.9 (352.5, 359.9)	367.8 (280.9, 504.5)
r-3	True	2.625	17.48	44.50	252.0	551.7
	CNF	2.642 (2.602, 2.711)	17.46 (16.99, 18.06)	44.11 (43.21, 44.94)	252.4 (248.4, 255.9)	461.0 (384.7, 547.7)
	PF	2.679 (2.627, 2.718)	17.62 (17.12, 17.88)	43.89 (43.08, 44.44)	252.9 (252.1, 256.1)	461.7 (390.0, 530.5)
r-4	True	3.566	21.43	45.99	338.0	114.4
	CNF	3.609 (3.486, 3.708)	21.24 (20.32, 22.05)	44.59 (43.10, 46.40)	338.8 (335.5, 341.5)	93.4 (66.9, 128.6)
	PF	3.556 (3.481, 3.639)	20.84 (20.26, 21.50)	44.92 (44.01, 46.20)	338.6 (336.5, 341.3)	83.0 (64.9, 117.7)
r-5	True	1.235	9.61	51.29	171.0	227.2
	CNF	1.232 (1.213, 1.255)	9.26 (8.81, 9.77)	50.67 (48.61, 52.31)	179.8 (168.4, 188.8)	190.6 (118.2, 252.6)
	PF	1.233 (1.216, 1.244)	9.37 (9.02, 9.79)	50.16 (49.09, 52.02)	177.7 (168.4, 186.2)	175.5 (123.6, 244.9)
r-6	True	1.217	10.79	5.37	337.0	497.2
	CNF	1.221 (1.195, 1.247)	10.68 (8.96, 14.43)	3.80 (2.11, 4.82)	346.7 (313.2, 359.0)	462.1 (265.1, 835.5)
	PF	1.213 (1.195, 1.231)	10.74 (9.30, 13.43)	4.17 (2.96, 5.08)	349.5 (319.6, 359.3)	433.7 (306.0, 700.3)
r-7	True	1.769	24.29	99.31	142.0	748.2
	CNF	1.764 (1.727, 1.797)	24.35 (23.12, 25.28)	99.57 (97.75, 99.96)	136.1 (125.2, 149.0)	743.3 (645.9, 849.1)
	PF	1.750 (1.730, 1.785)	23.75 (23.20, 24.69)	99.62 (98.10, 99.98)	142.1 (130.3, 149.5)	723.0 (631.9, 821.1)
r-8	True	1.062	13.71	80.25	167.0	940.2
	CNF	1.058 (1.036, 1.092)	13.30 (12.21, 14.77)	78.77 (72.62, 84.95)	187.8 (154.8, 211.2)	967.8 (780.8, 996.4)
	PF	1.076 (1.043, 1.093)	13.70 (12.33, 14.72)	80.62 (75.43, 85.60)	196.2 (158.8, 210.4)	913.3 (783.6, 995.5)
r-9	True	2.621	13.69	8.07	265.0	135.1
	CNF	2.604 (2.549, 2.651)	13.33 (12.73, 13.96)	8.24 (7.67, 8.73)	267.8 (263.5, 272.3)	93.0 (62.7, 140.3)
	PF	2.611 (2.566, 2.654)	13.55 (12.94, 14.04)	8.29 (7.88, 8.69)	265.4 (263.0, 270.5)	107.9 (74.3, 149.2)
r-10	True	1.166	27.22	35.70	147.0	428.9
	CNF	1.223 (1.141, 1.274)	28.61 (17.05, 29.90)	32.38 (27.49, 38.11)	201.4 (138.2, 350.9)	409.8 (212.3, 499.3)
	PF	1.197 (1.137, 1.260)	22.90 (17.25, 29.54)	32.48 (27.34, 37.05)	253.0 (136.7, 339.0)	294.9 (200.9, 467.6)

Table S6.1: Posterior MAP estimates with 95% intervals for the reparametrized SEIR2V model.

Table S6.2: **Effective sample sizes (ESS) per parameter and model.** ESS computed on the last 10,000 samples of the chains resulting from running the PF method on the reparametrized two-variant SEIR model and using a maximum lag size of 250 for the autocorrelation.

**Dataset**	$\mathbf{r_0}$	$\mathbf{e_0}$	$\mathbf{s_0}$	$\mathbf{t_{var}}$	$\mathbf{I_0}$
d-1-1	2409.8	2453.8	2370.5	2276.2	2382.2
d-1-2	2675.0	2413.2	2495.5	2537.6	2571.3
r-1	4104.6	4182.8	3869.8	3936.5	3924.8
r-2	2347.3	2342.5	2624.4	1185.0	2554.2
r-3	3522.5	3357.1	3486.6	3563.4	3688.1
r-4	4017.0	4069.4	4030.9	3856.0	3892.9
r-5	3748.5	3306.9	3285.5	3515.8	3800.7
r-6	3383.7	3254.2	3251.3	3147.4	3304.3
r-7	3142.5	3307.9	2932.6	3445.8	3639.8
r-8	3054.0	2826.0	3278.7	2758.7	2658.6
r-9	3566.7	3242.8	3289.5	3309.4	3212.1
r-10	3009.9	3090.7	2966.8	3003.5	3326.2

Table S6.3: **$\hat{\mathbf{R}}$ diagnostics for the reparametrized SEIR model.**

**Dataset**	$\mathbf{r_0}$	$\mathbf{e_0}$	$\mathbf{s_0}$	$\mathbf{t_{var}}$	$\mathbf{I_0}$
d-1-1	1.002	1.001	1.005	1.003	1.002
d-1-2	1.002	1.002	1.002	1.002	1.002
r-1	1.004	1.003	1.001	1.002	1.004
r-2	1.001	1.005	1.001	1.009	1.005
r-3	1.002	1.001	1.002	1.002	1.001
r-4	1.004	1.003	1.001	1.003	1.001
r-5	1.003	1.001	1.003	1.002	1.001
r-6	1.002	1.002	1.002	1.002	1.001
r-7	1.002	1.003	1.001	1.003	1.001
r-8	1.002	1.002	1.003	1.002	1.003
r-9	1.002	1.002	1.003	1.002	1.002
r-10	1.001	1.003	1.002	1.003	1.005
