# Supplementary material for: Assessment of simulation-based inference methods for stochastic compartmental models in epidemiological research
Source: PLoS One. 2026 Jul 13;21(7):e0353306. doi: 10.1371/journal.pone.0353306 (PMC13362117; doi:10.1371/journal.pone.0353306)
Supplement: S7 Results — (PDF) [file pone.0353306.s007.pdf]

S7 Supplementary Results Comparison Full SEIR-Model with Reparametrized Model

Assessment of Simulation-based Inference Methods for Stochastic Compartmental Models in Epidemiological Research

Vincent Wieland[1,2,◕], Nils Waßmuth[1,2,3,◕], Lorenzo Contento[1], Martin Kühn[1,2,3], and Jan Hasenauer[1,2,*]

[1]Bonn Center for Mathematical Life Sciences, University of Bonn, Bonn, Germany
[2]Life and Medical Science Institute, University of Bonn, Bonn, Germany
[3]Institute of Software Technology, Department for High-Performance Computing, German Aerospace Center (DLR), Cologne, Germany
[◕]These authors contributed equally to the work.
[*]To whom correspondence should be addressed; jan.hasenauer@uni-bonn.de.

June 26, 2026

Contents

S7.A Supplementary Figures 2
S7.B Supplementary Tables 19

S7.A Supplementary Figures

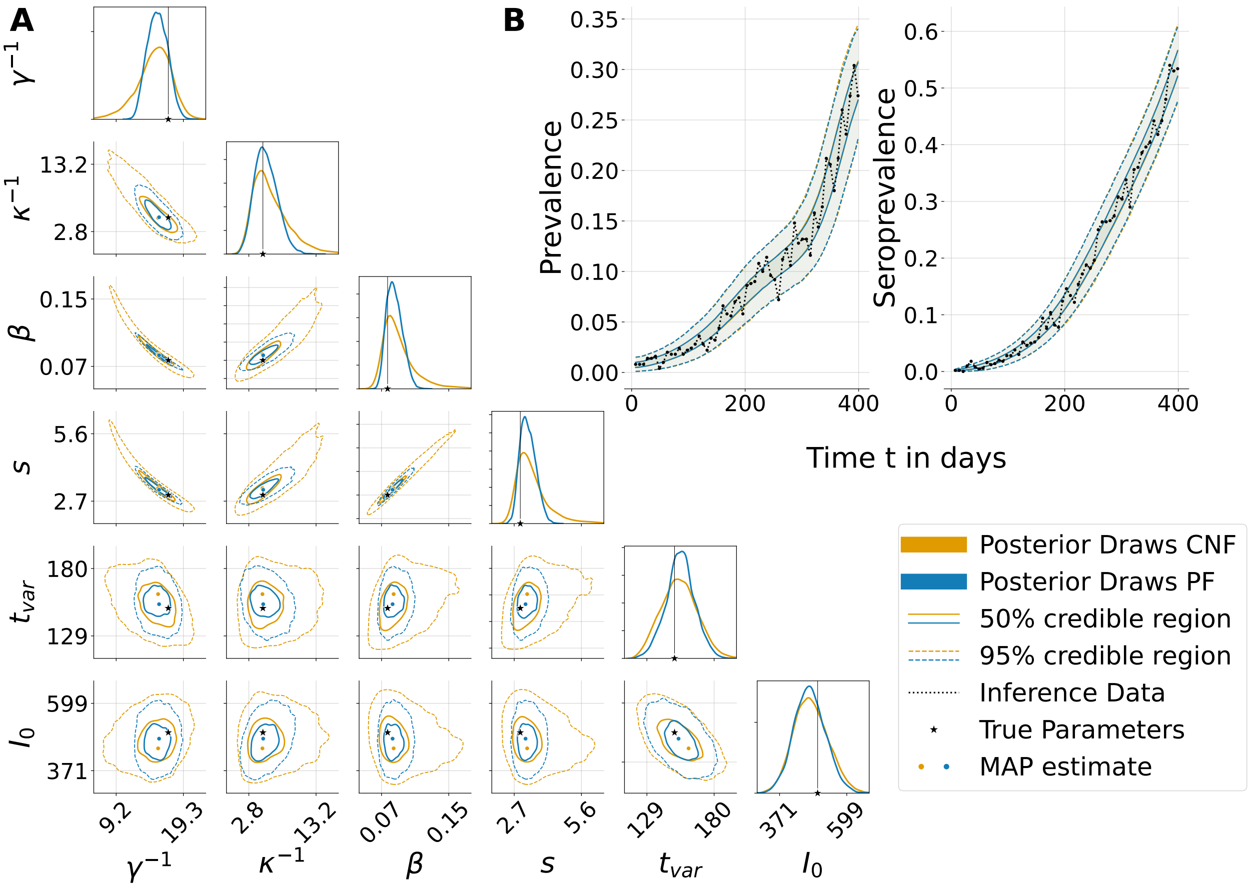

Figure S7.1: **Results of the two-variant SEIR model for *d-1-1*.**
A Posterior approximations from 10,000 samples. Contour gives the 50% (solid) and 95% (dashed) credible regions, coloured by method. Diagonals show the 1D marginals. Black stars mark the true parameters, coloured circles the joint MAP estimates. **B** Posterior predictive fit: bands give the 50% and 95% pointwise predictive intervals from the same samples (line styles as in **A**) with inference data shown as a dotted line.

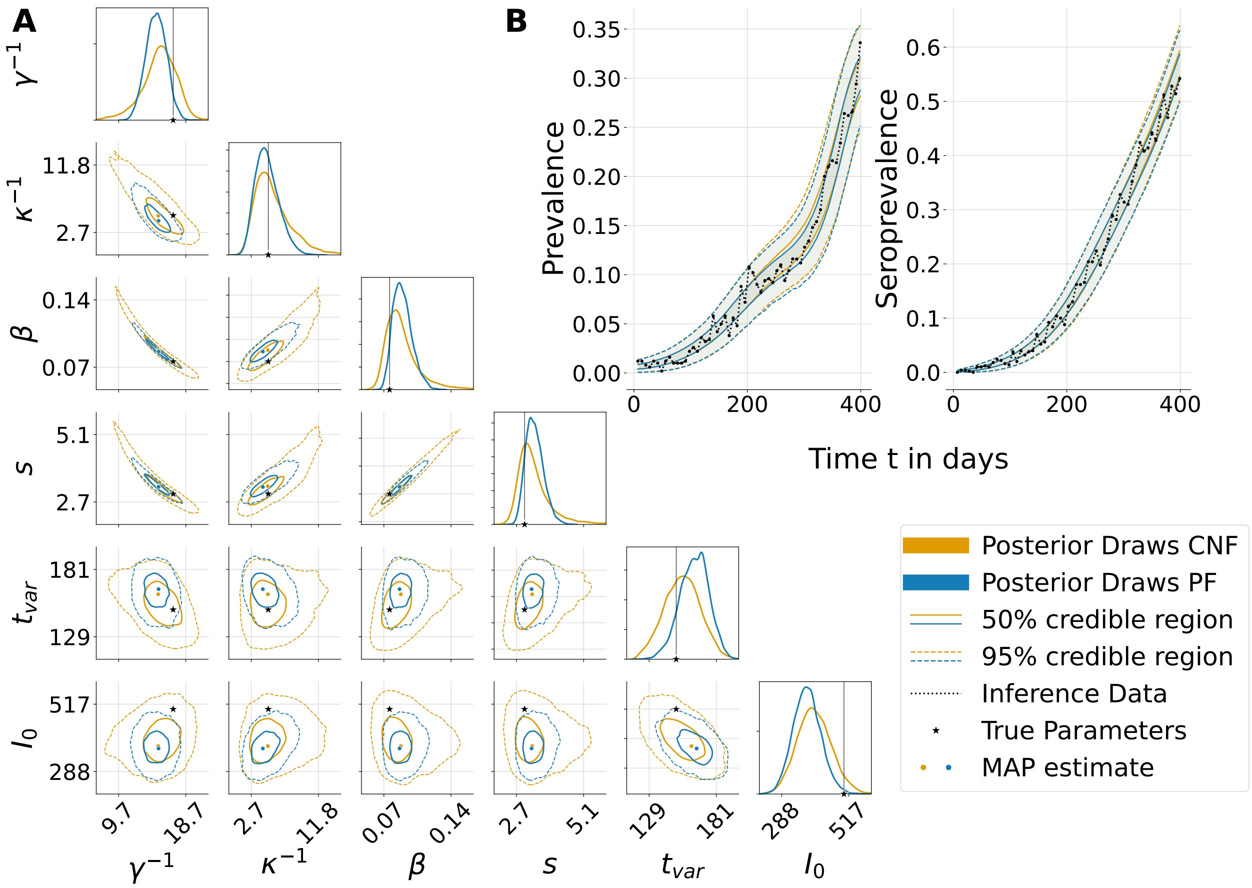

Figure S7.2: **Results of the two-variant SEIR model for *d-1-2*.**
A Posterior approximations from 10,000 samples. Contour gives the 50% (solid) and 95% (dashed) credible regions, coloured by method. Diagonals show the 1D marginals. Black stars mark the true parameters, coloured circles the joint MAP estimates. **B** Posterior predictive fit: bands give the 50% and 95% pointwise predictive intervals from the same samples (line styles as in **A**) with inference data shown as a dotted line.

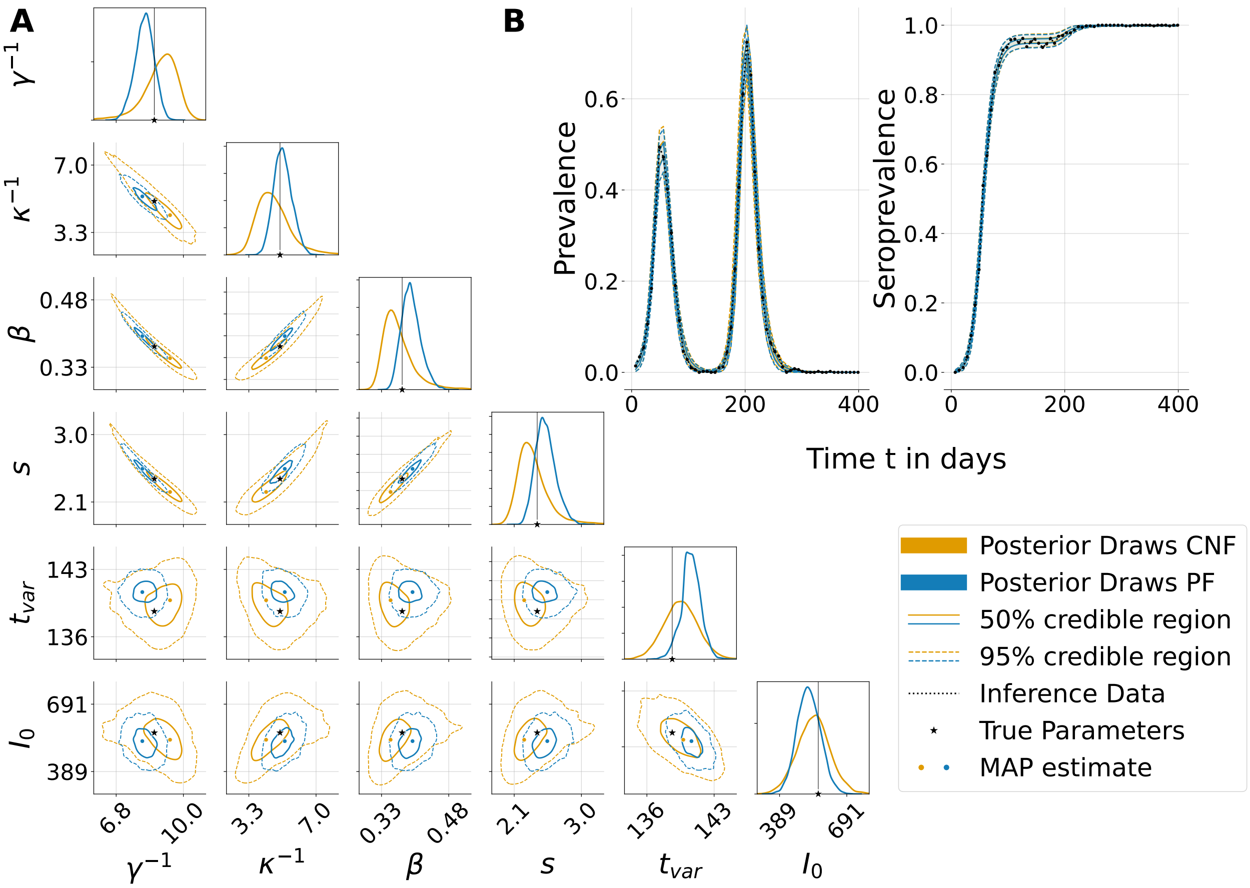

Figure S7.3: **Results of the two-variant SEIR model for *r-1*.**
A Posterior approximations from 10,000 samples. Contour gives the 50% (solid) and 95% (dashed) credible regions, coloured by method. Diagonals show the 1D marginals. Black stars mark the true parameters, coloured circles the joint MAP estimates. **B** Posterior predictive fit: bands give the 50% and 95% pointwise predictive intervals from the same samples (line styles as in **A**) with inference data shown as a dotted line.

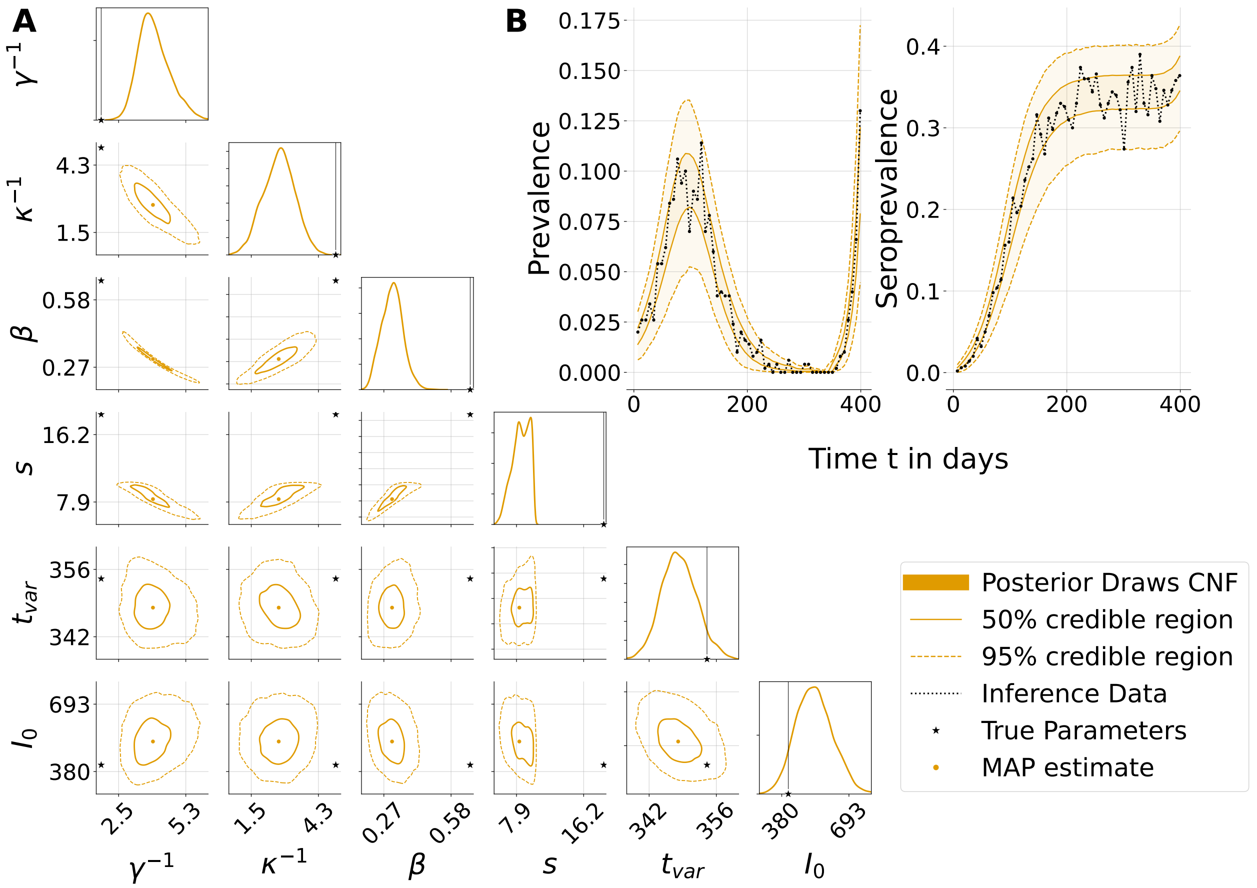

Figure S7.4: **Results of the two-variant SEIR model for *r-2*.**
A Posterior approximations from 10,000 samples. Contour gives the 50% (solid) and 95% (dashed) credible regions, coloured by method. Diagonals show the 1D marginals. Black stars mark the true parameters, coloured circles the joint MAP estimates. **B** Posterior predictive fit: bands give the 50% and 95% pointwise predictive intervals from the same samples (line styles as in **A**) with inference data shown as a dotted line.

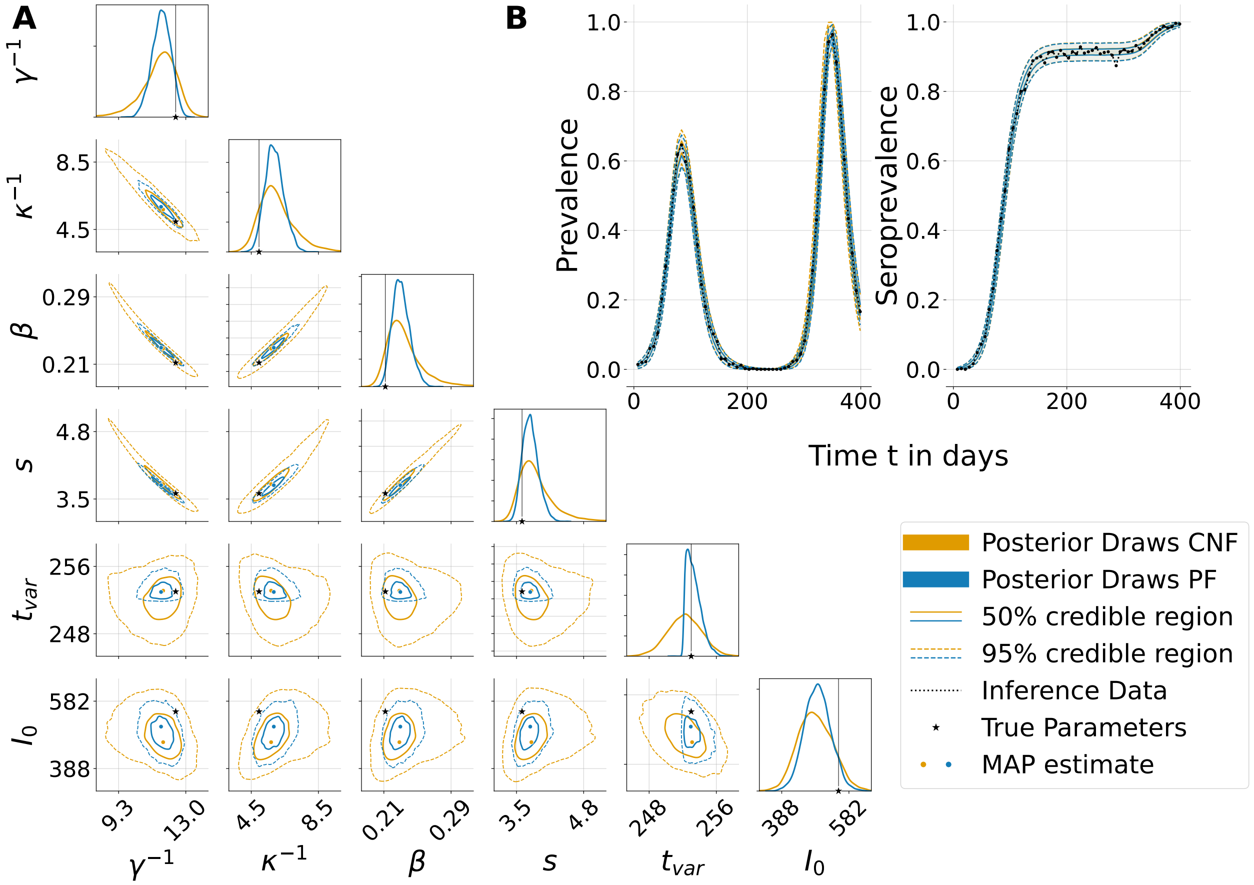

Figure S7.5: **Results of the two-variant SEIR model for *r-3*.**
A Posterior approximations from 10,000 samples. Contour gives the 50% (solid) and 95% (dashed) credible regions, coloured by method. Diagonals show the 1D marginals. Black stars mark the true parameters, coloured circles the joint MAP estimates. **B** Posterior predictive fit: bands give the 50% and 95% pointwise predictive intervals from the same samples (line styles as in **A**) with inference data shown as a dotted line.

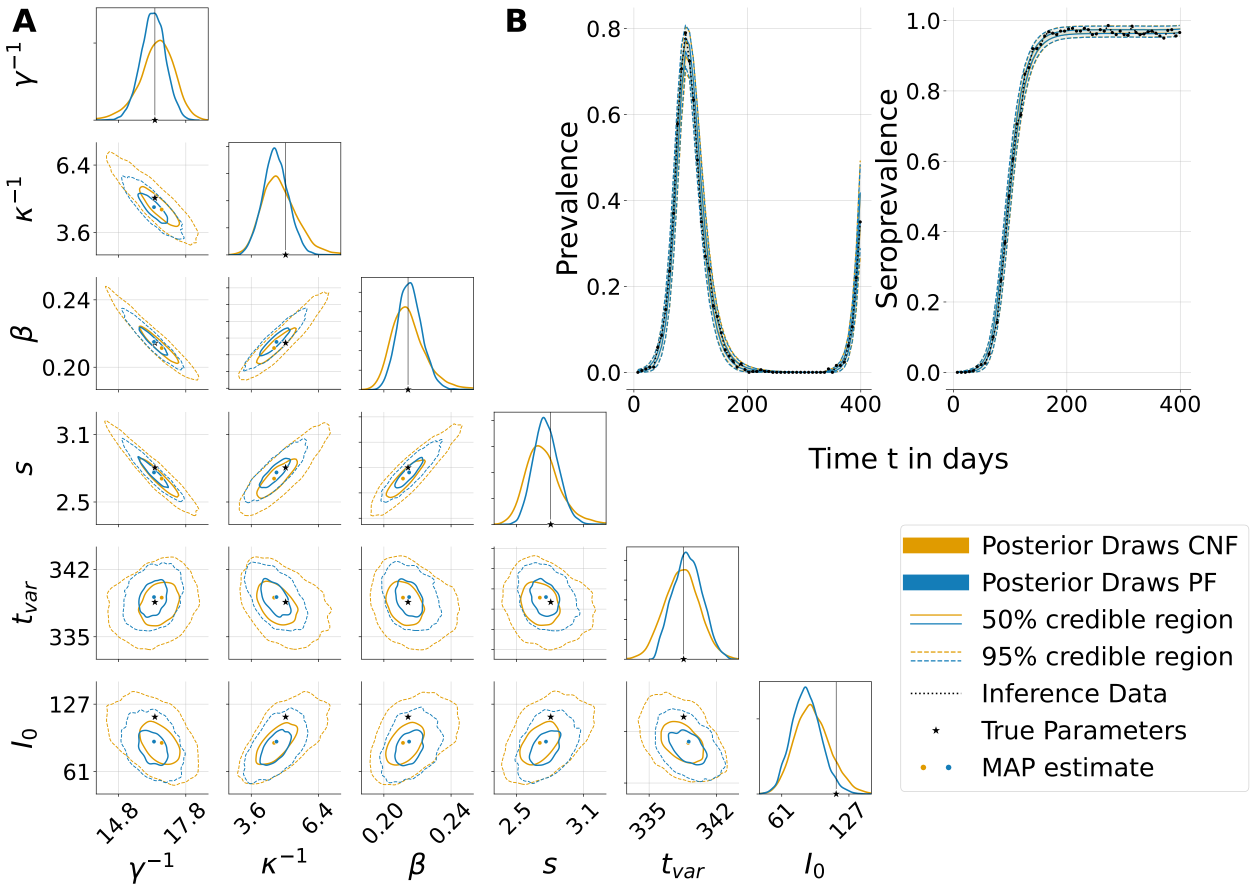

Figure S7.6: **Results of the two-variant SEIR model for *r-4*.**
A Posterior approximations from 10,000 samples. Contour gives the 50% (solid) and 95% (dashed) credible regions, coloured by method. Diagonals show the 1D marginals. Black stars mark the true parameters, coloured circles the joint MAP estimates. **B** Posterior predictive fit: bands give the 50% and 95% pointwise predictive intervals from the same samples (line styles as in **A**) with inference data shown as a dotted line.

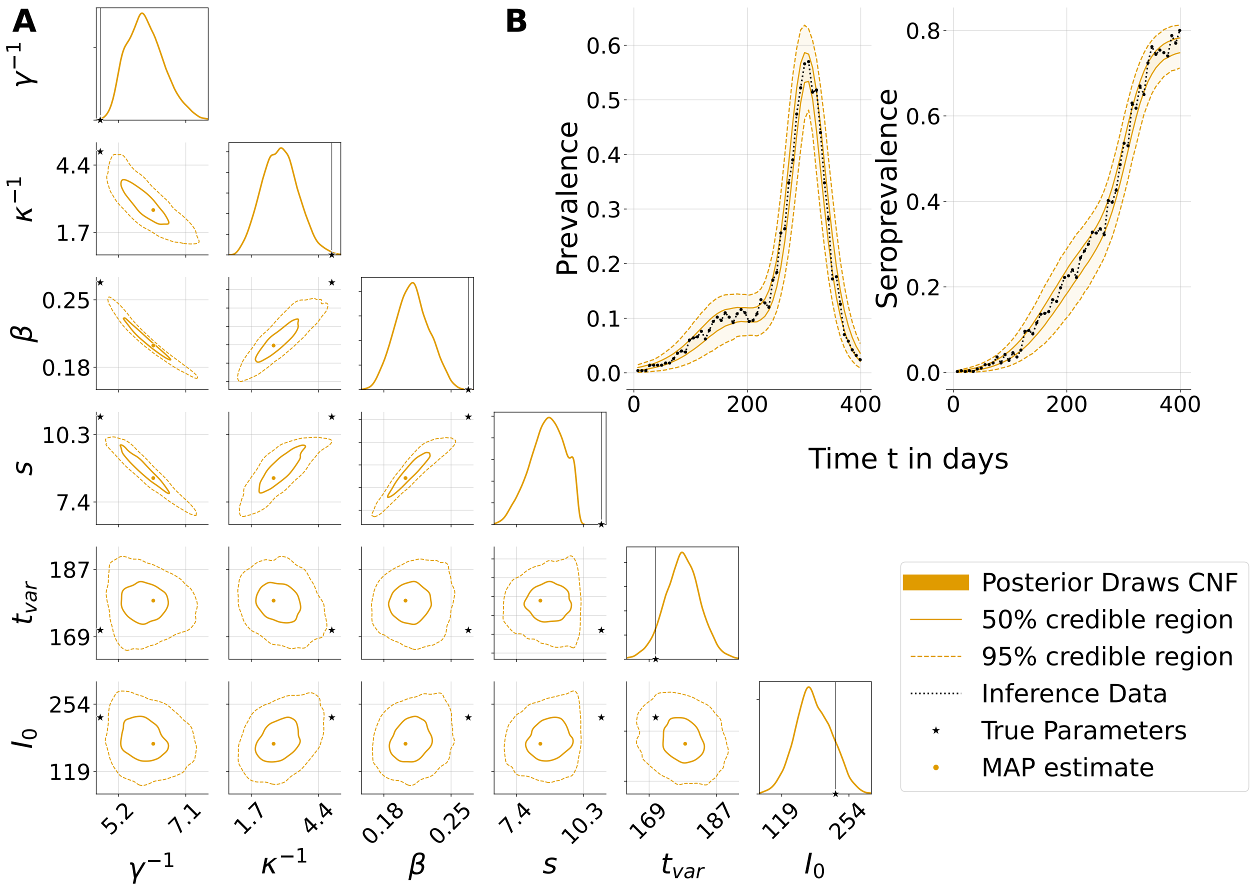

Figure S7.7: **Results of the two-variant SEIR model for *r-5*.**
A Posterior approximations from 10,000 samples. Contour gives the 50% (solid) and 95% (dashed) credible regions, coloured by method. Diagonals show the 1D marginals. Black stars mark the true parameters, coloured circles the joint MAP estimates. **B** Posterior predictive fit: bands give the 50% and 95% pointwise predictive intervals from the same samples (line styles as in **A**) with inference data shown as a dotted line.

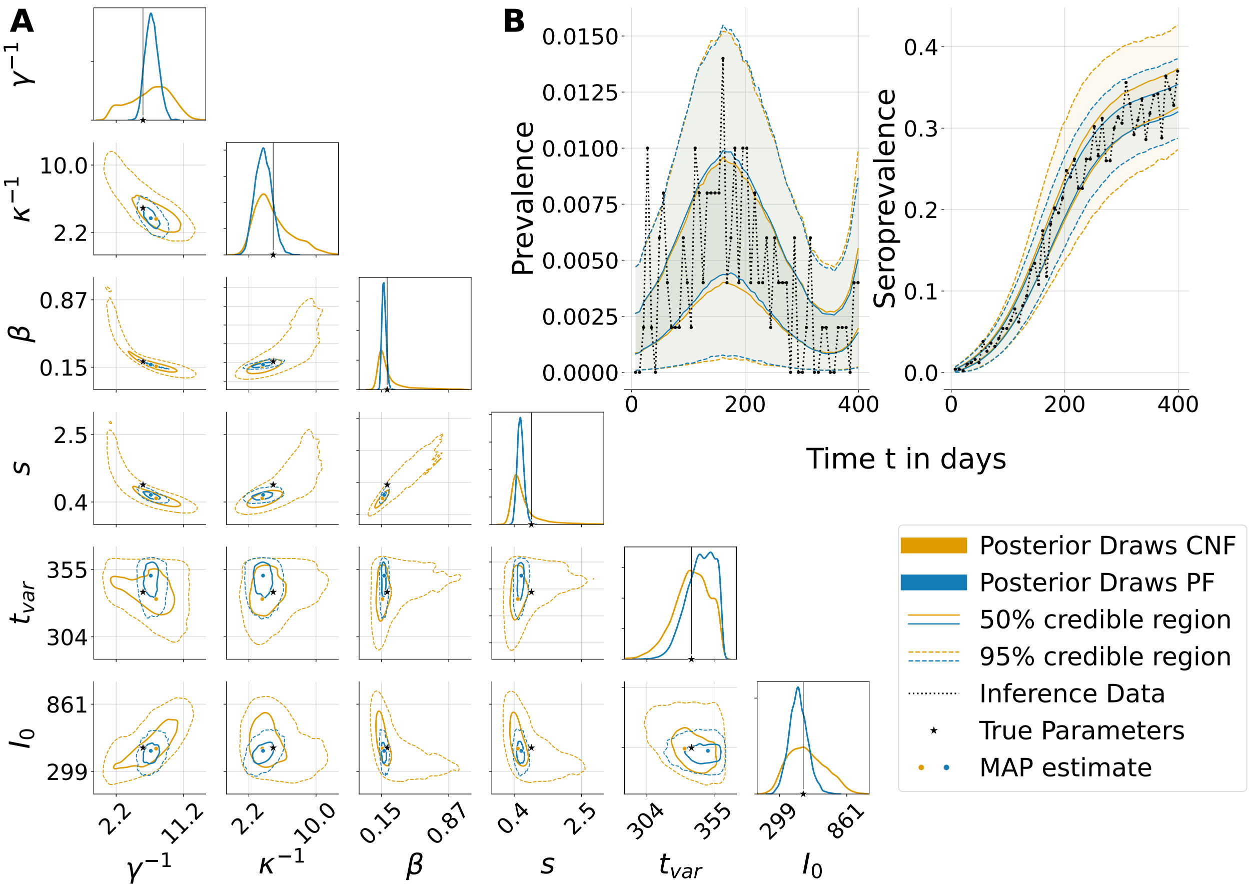

Figure S7.8: **Results of the two-variant SEIR model for *r-6*.**
A Posterior approximations from 10,000 samples. Contour gives the 50% (solid) and 95% (dashed) credible regions, coloured by method. Diagonals show the 1D marginals. Black stars mark the true parameters, coloured circles the joint MAP estimates. **B** Posterior predictive fit: bands give the 50% and 95% pointwise predictive intervals from the same samples (line styles as in **A**) with inference data shown as a dotted line.

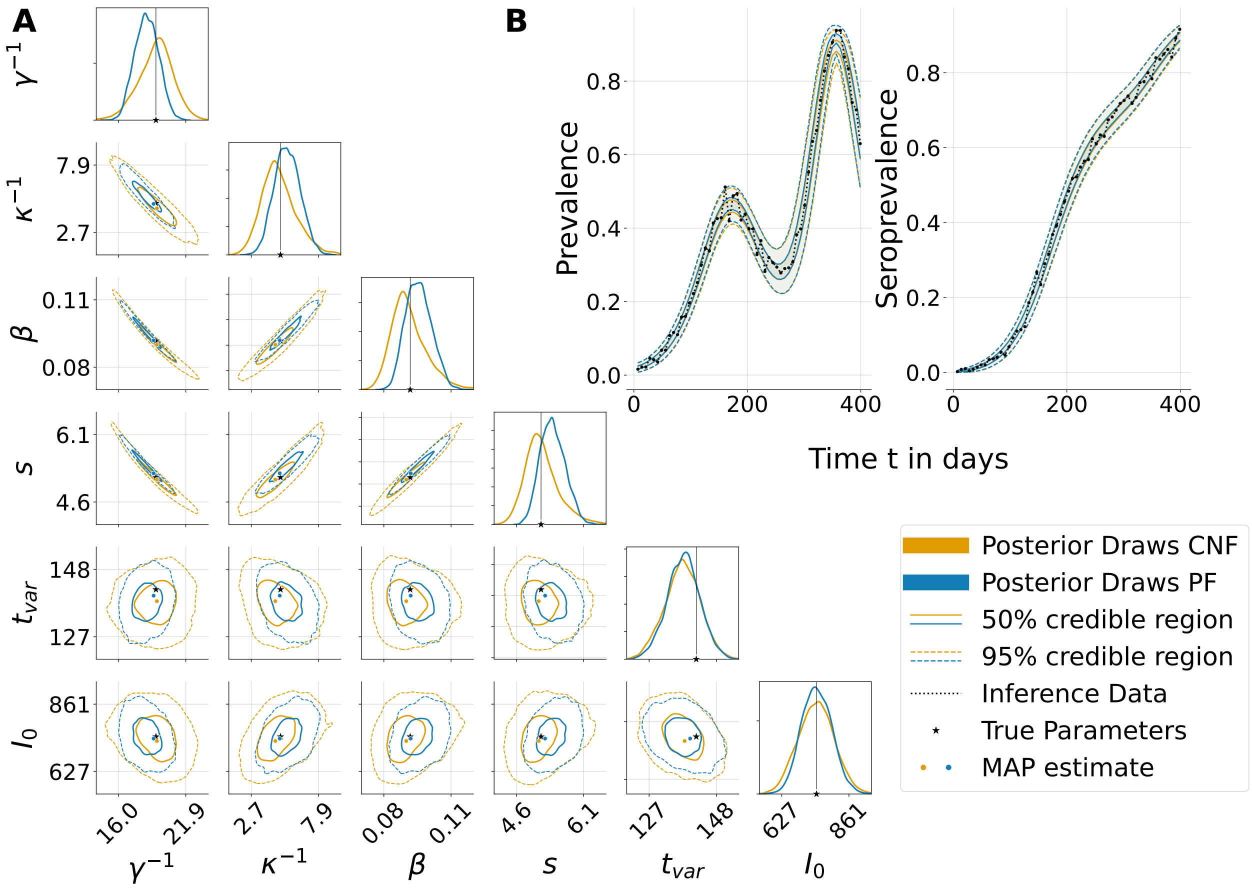

Figure S7.9: **Results of the two-variant SEIR model for r-7.**
A Posterior approximations from 10,000 samples. Contour gives the 50% (solid) and 95% (dashed) credible regions, coloured by method. Diagonals show the 1D marginals. Black stars mark the true parameters, coloured circles the joint MAP estimates. **B** Posterior predictive fit: bands give the 50% and 95% pointwise predictive intervals from the same samples (line styles as in **A**) with inference data shown as a dotted line.

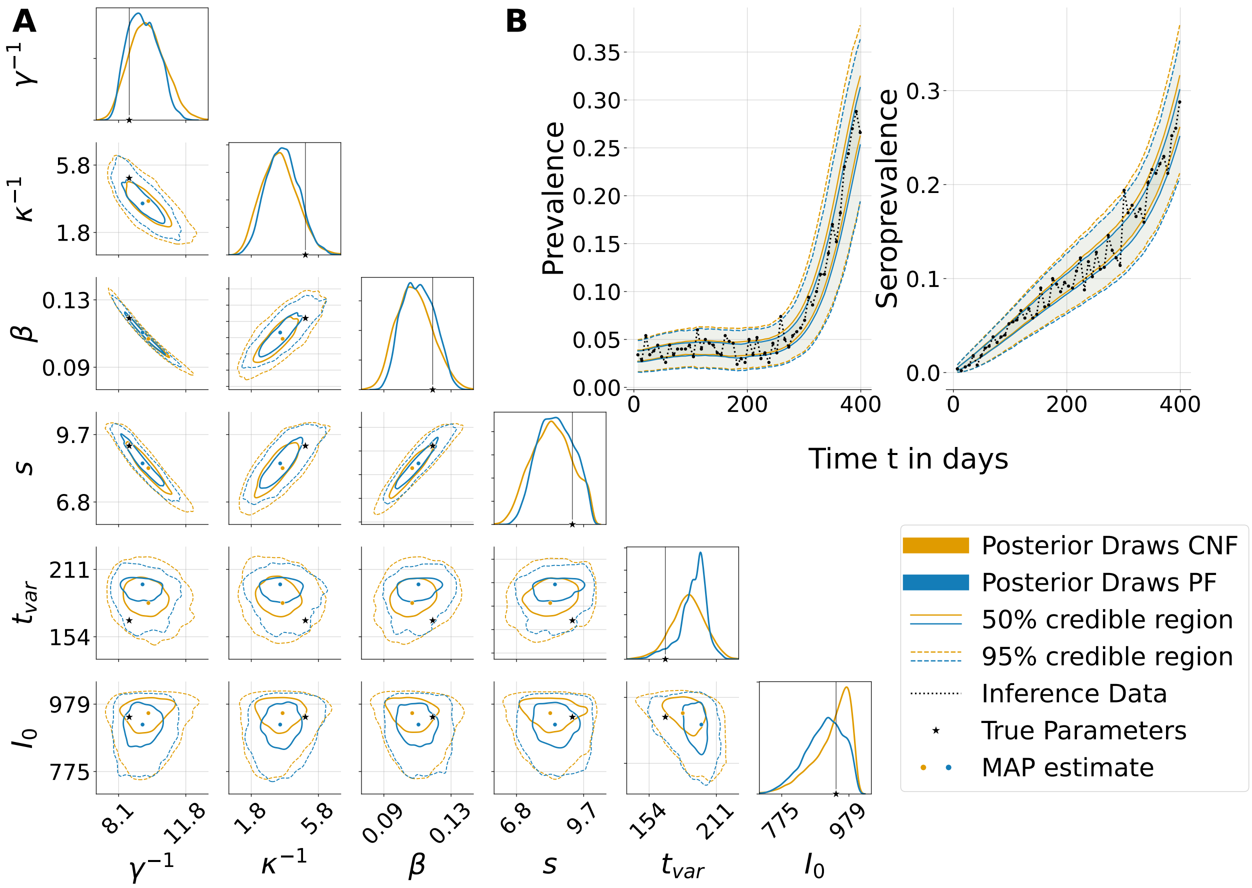

Figure S7.10: **Results of the two-variant SEIR model for *r-8*.**

A Posterior approximations from 10,000 samples. Contour gives the 50% (solid) and 95% (dashed) credible regions, coloured by method. Diagonals show the 1D marginals. Black stars mark the true parameters, coloured circles the joint MAP estimates. **B** Posterior predictive fit: bands give the 50% and 95% pointwise predictive intervals from the same samples (line styles as in **A**) with inference data shown as a dotted line.

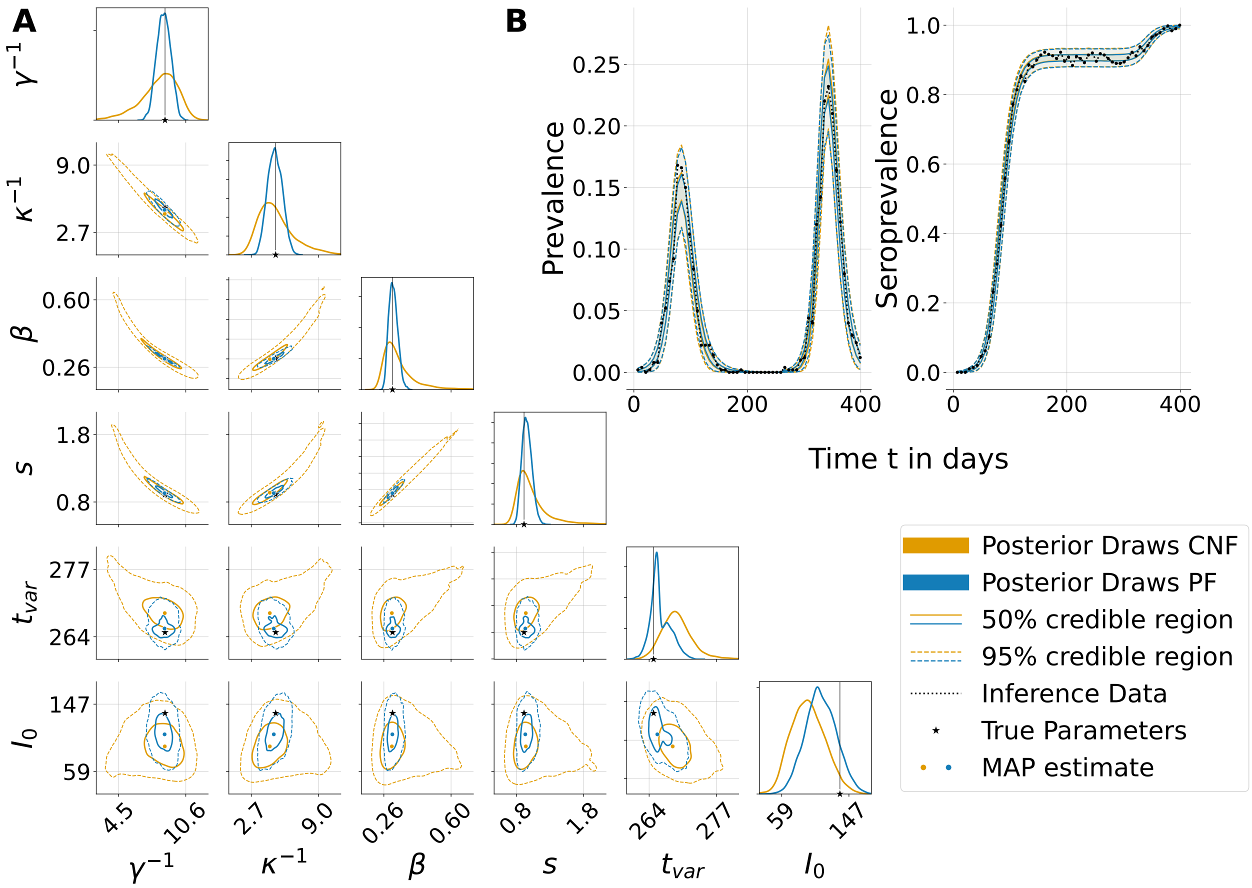

Figure S7.11: **Results of the two-variant SEIR model for *r-9*.**

A Posterior approximations from 10,000 samples. Contour gives the 50% (solid) and 95% (dashed) credible regions, coloured by method. Diagonals show the 1D marginals. Black stars mark the true parameters, coloured circles the joint MAP estimates. **B** Posterior predictive fit: bands give the 50% and 95% pointwise predictive intervals from the same samples (line styles as in **A**) with inference data shown as a dotted line.

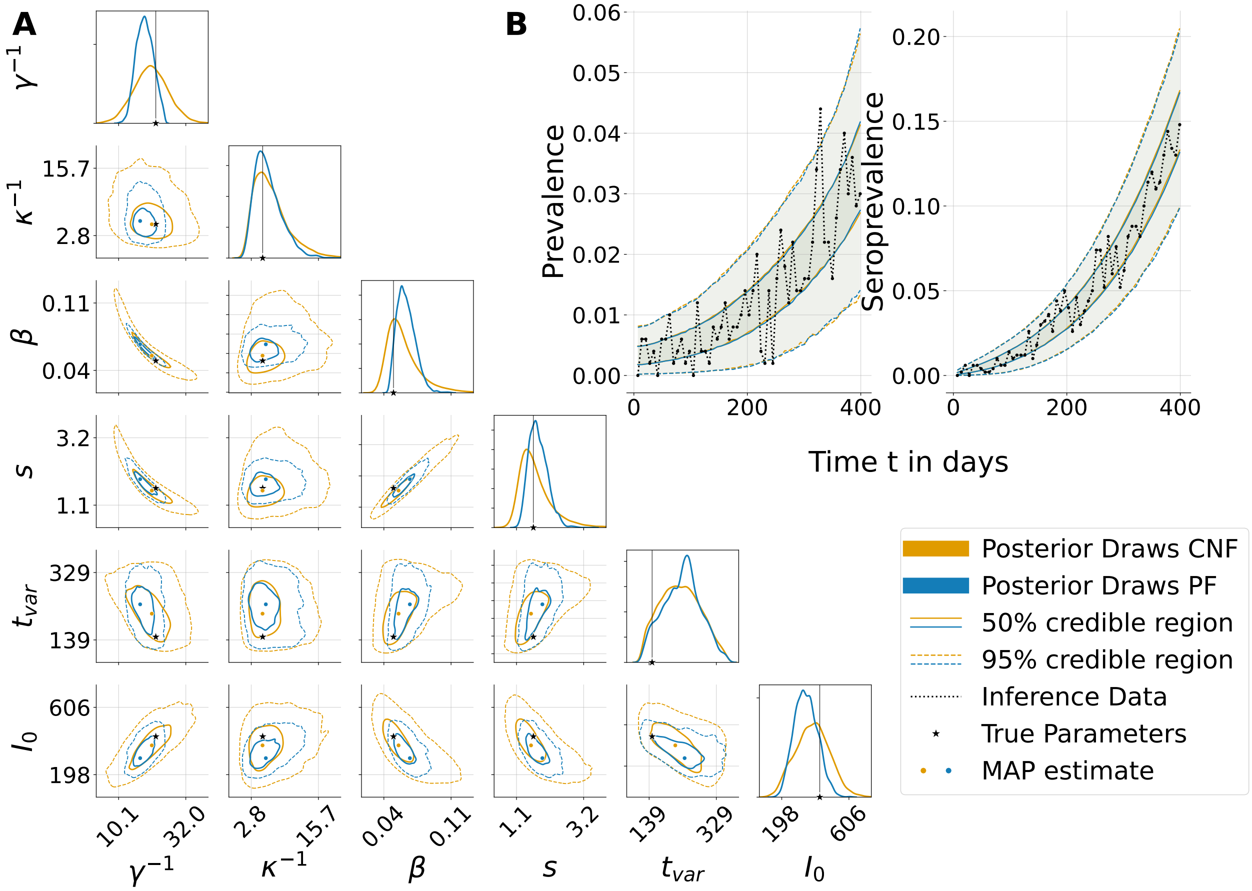

Figure S7.12: **Results of the two-variant SEIR model for *r-10*.**
A Posterior approximations from 10,000 samples. Contour gives the 50% (solid) and 95% (dashed) credible regions, coloured by method. Diagonals show the 1D marginals. Black stars mark the true parameters, coloured circles the joint MAP estimates. **B** Posterior predictive fit: bands give the 50% and 95% pointwise predictive intervals from the same samples (line styles as in **A**) with inference data shown as a dotted line.

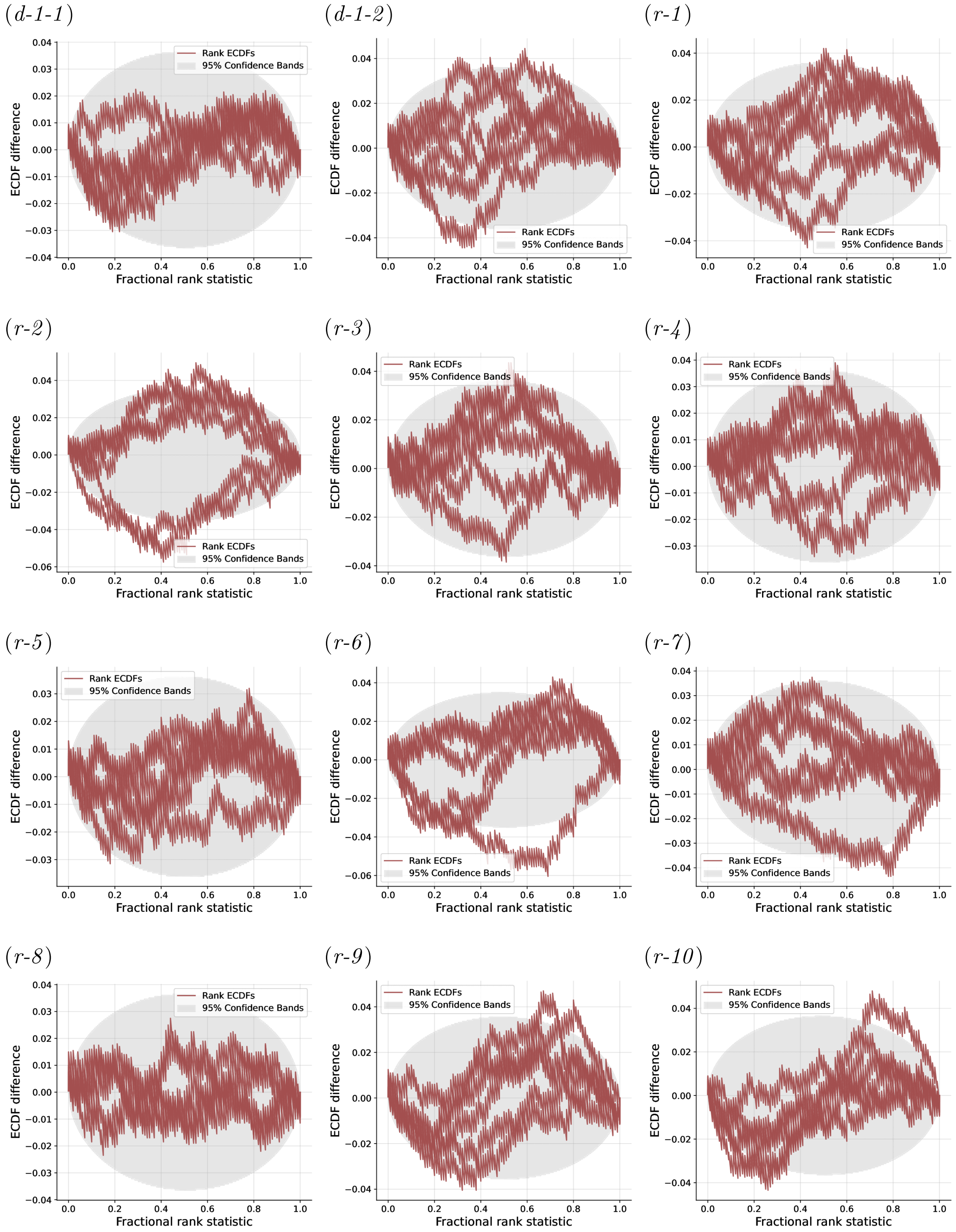

Figure S7.13: **ECDF Calibration plots for the full SEIR model with datasets for the reparametrized model.**

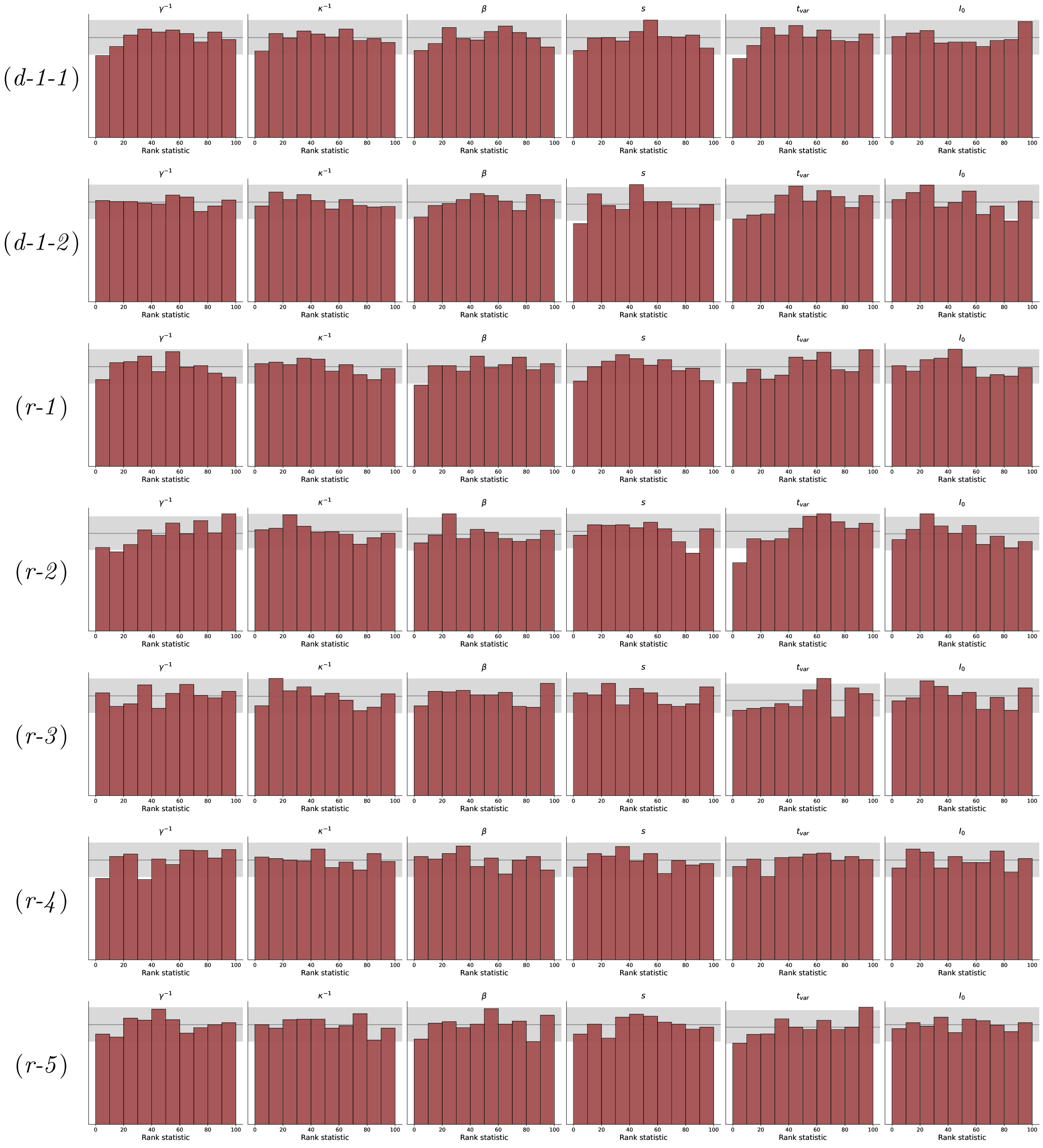
(d-1-1)
(d-1-2)
(r-1)
(r-2)
(r-3)
(r-4)
(r-5)
γ⁻¹
κ⁻¹
β
s
t_var
I_0
0 20 40 60 80 100
Rank statistic

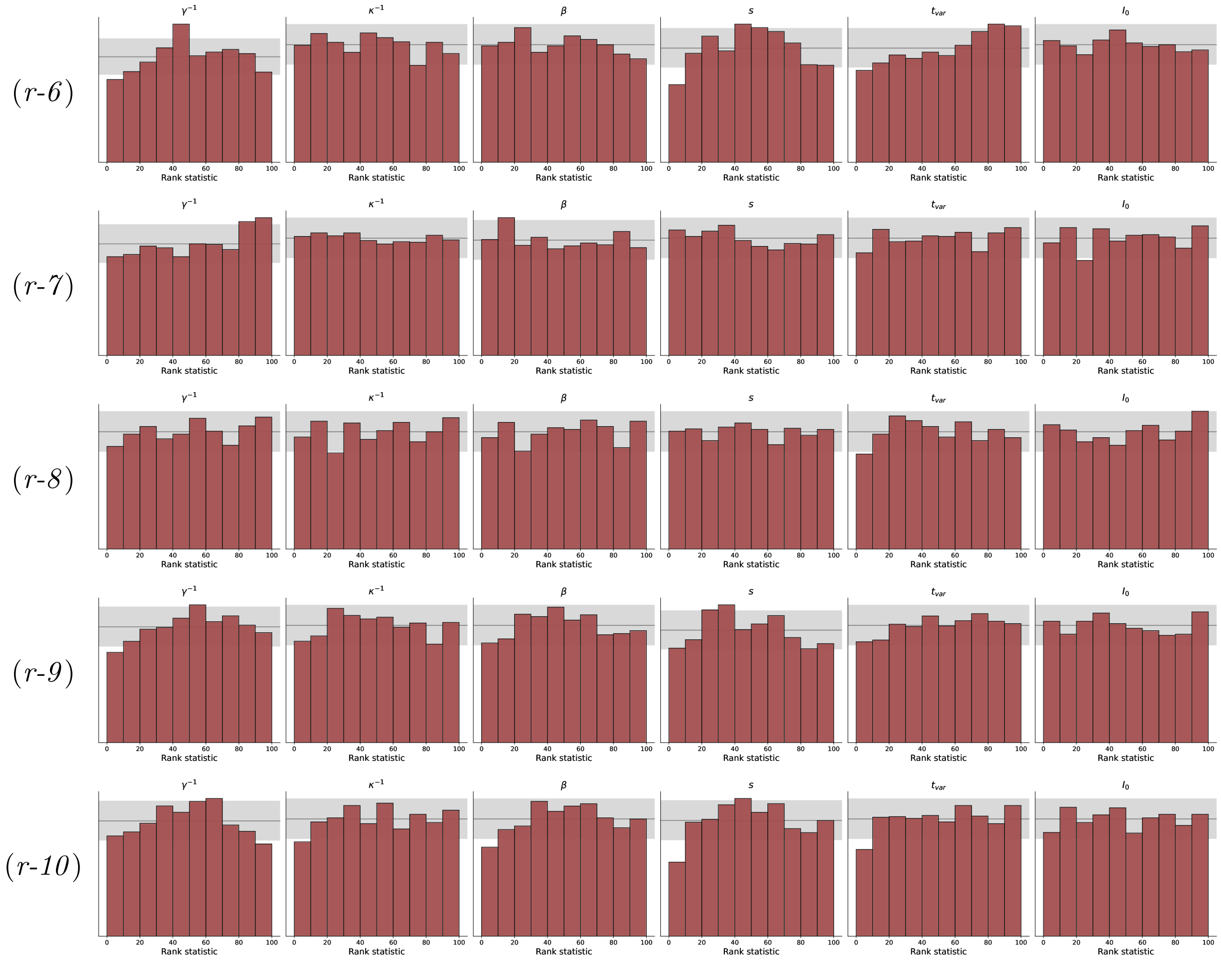

Figure S7.13: **SBC Histograms for the full SEIR model and datasets for the reparametrized model.**

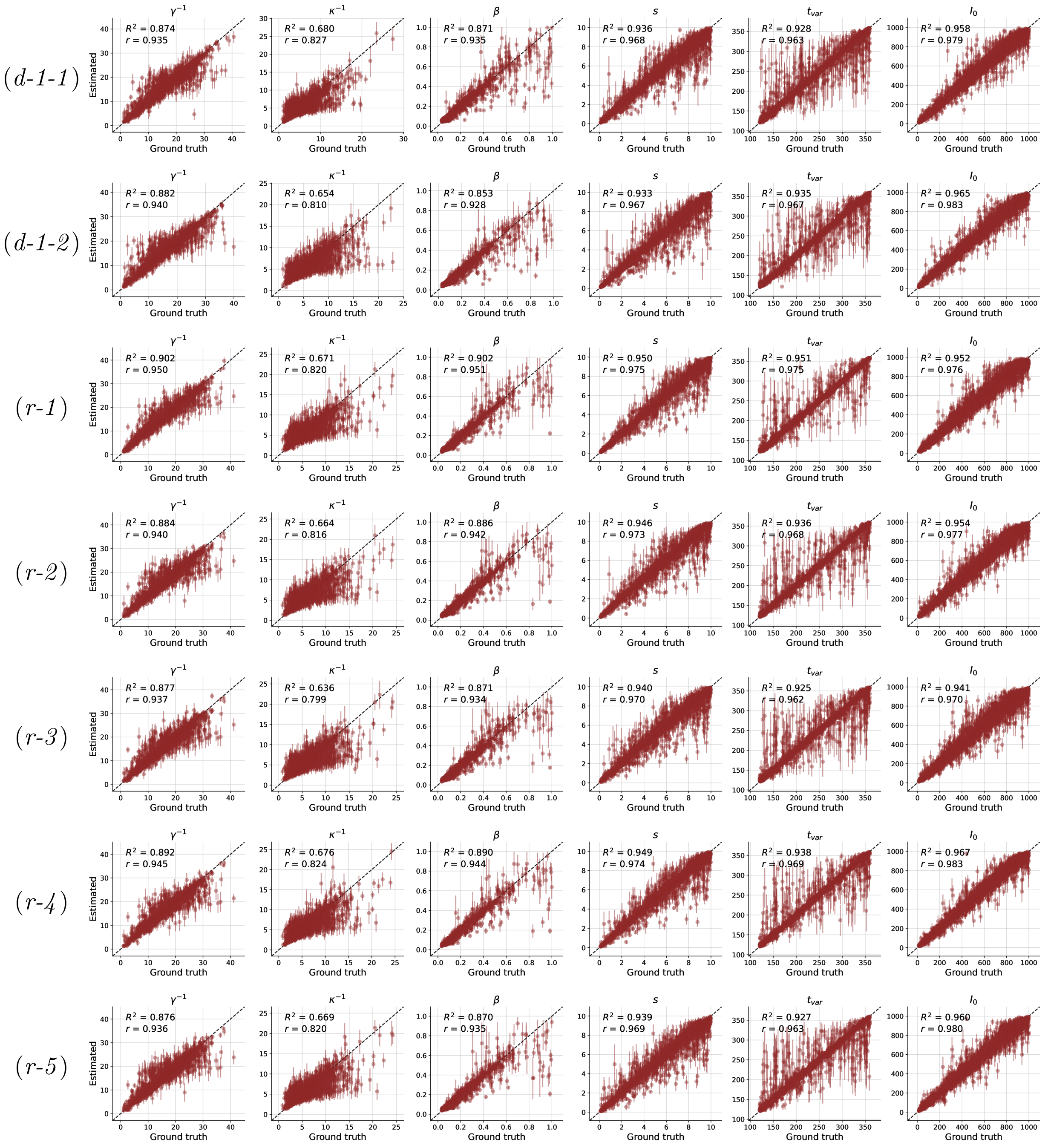
(d-1-1)
γ^{-1}
R^2 = 0.874
r = 0.935
κ^{-1}
R^2 = 0.680
r = 0.827
β
R^2 = 0.871
r = 0.935
s
R^2 = 0.936
r = 0.968
t_{var}
R^2 = 0.928
r = 0.963
I_0
R^2 = 0.958
r = 0.979
(d-1-2)
R^2 = 0.882
r = 0.940
R^2 = 0.654
r = 0.810
R^2 = 0.853
r = 0.928
R^2 = 0.933
r = 0.967
R^2 = 0.935
r = 0.967
R^2 = 0.965
r = 0.983
(r-1)
R^2 = 0.902
r = 0.950
R^2 = 0.671
r = 0.820
R^2 = 0.902
r = 0.951
R^2 = 0.950
r = 0.975
R^2 = 0.951
r = 0.975
R^2 = 0.952
r = 0.976
(r-2)
R^2 = 0.884
r = 0.940
R^2 = 0.664
r = 0.816
R^2 = 0.886
r = 0.942
R^2 = 0.946
r = 0.973
R^2 = 0.936
r = 0.968
R^2 = 0.954
r = 0.977
(r-3)
R^2 = 0.877
r = 0.937
R^2 = 0.636
r = 0.799
R^2 = 0.871
r = 0.934
R^2 = 0.940
r = 0.970
R^2 = 0.925
r = 0.962
R^2 = 0.941
r = 0.970
(r-4)
R^2 = 0.892
r = 0.945
R^2 = 0.676
r = 0.824
R^2 = 0.890
r = 0.944
R^2 = 0.949
r = 0.974
R^2 = 0.938
r = 0.969
R^2 = 0.967
r = 0.983
(r-5)
R^2 = 0.876
r = 0.936
R^2 = 0.669
r = 0.820
R^2 = 0.870
r = 0.935
R^2 = 0.939
r = 0.969
R^2 = 0.927
r = 0.963
R^2 = 0.960
r = 0.980
Estimated
Ground truth

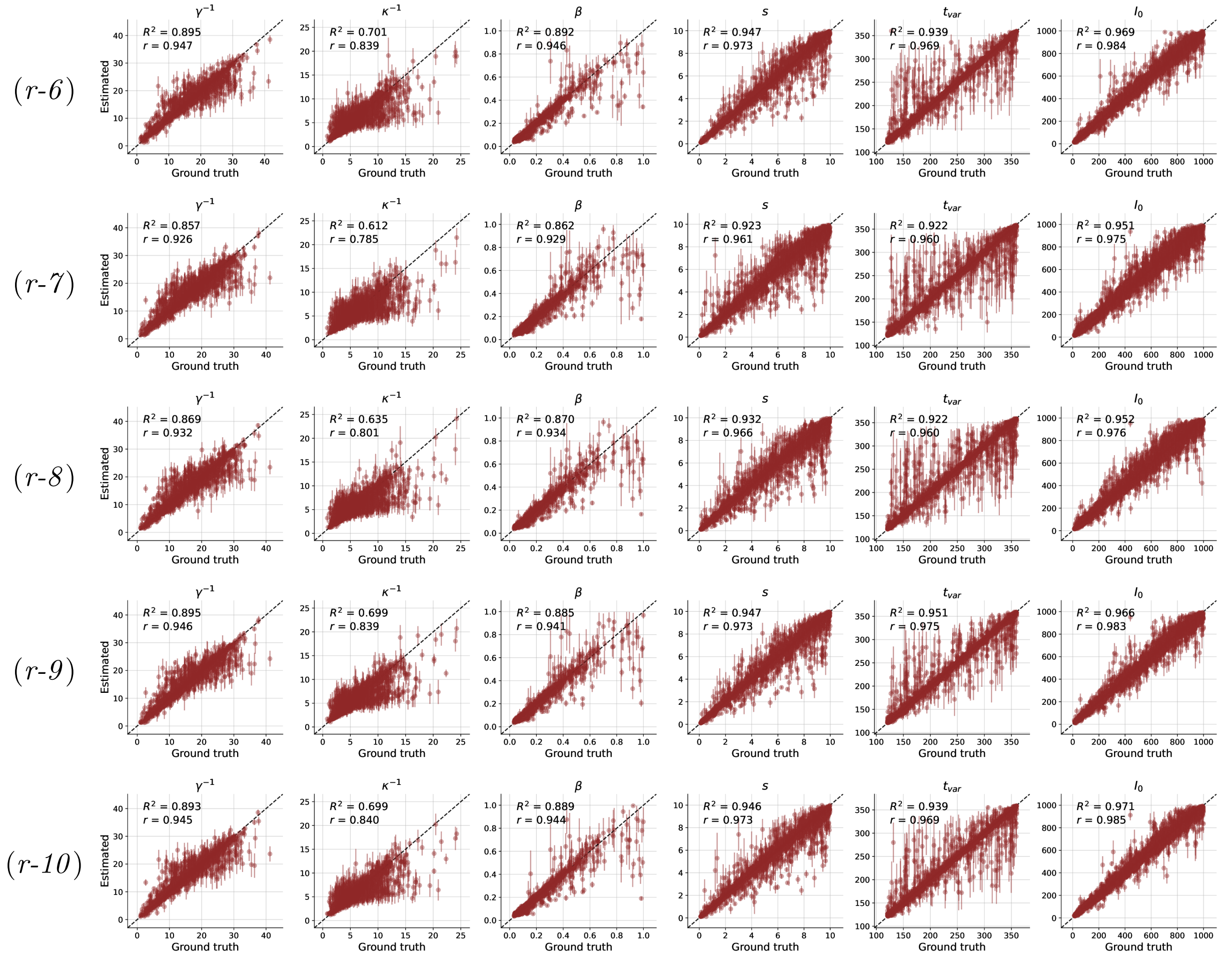

Figure S7.13: **Parameter recovery for the full SEIR model and datasets for the reparametrized model.**

S7.B Supplementary Tables

Table S7.1: Posterior MAP estimates with 95% intervals for the full SEIR2V model with data for the reparametrized model.

Dataset	Method	γ^{-1}	κ^{-1}	β	s	t_{var}	I_0
d-1-1	True	17.00	5.00	0.0800	3.00	150.0	500.0
	CNF	15.47 (8.83, 19.26)	4.93 (2.42, 13.54)	0.0866 (0.07, 0.15)	3.29 (2.6, 5.8)	160.6 (128.5, 180.8)	446.3 (370.1, 598.2)
	PF	15.62 (12.65, 18.29)	5.04 (2.45, 9.18)	0.0855 (0.07, 0.11)	3.22 (2.8, 4.0)	153.0 (132.8, 175.5)	479.2 (371.7, 581.0)
d-1-2	True	17.00	5.00	0.0800	3.00	150.0	500.0
	CNF	15.00 (9.86, 19.00)	4.95 (2.24, 11.77)	0.0902 (0.07, 0.14)	3.28 (2.6, 5.1)	161.7 (128.3, 180.7)	374.7 (291.7, 519.0)
	PF	15.04 (12.10, 17.34)	4.26 (2.22, 8.39)	0.0890 (0.08, 0.11)	3.25 (2.8, 4.0)	165.6 (141.1, 183.6)	366.6 (285.9, 470.9)
r-1	True	8.63	5.00	0.3754	2.41	138.0	563.0
	CNF	9.37 (6.76, 10.13)	4.22 (3.18, 7.26)	0.3488 (0.32, 0.48)	2.23 (2.1, 3.0)	139.9 (136.2, 143.3)	531.5 (398.0, 689.9)
	PF	8.07 (7.27, 9.00)	5.27 (4.23, 6.20)	0.3992 (0.36, 0.44)	2.55 (2.3, 2.8)	140.8 (138.6, 142.7)	525.4 (420.0, 625.7)
r-2	True	1.83	5.00	0.6617	18.74	353.0	410.5
	CNF	3.94 (2.93, 5.45)	2.63 (1.36, 3.90)	0.3114 (0.22, 0.41)	8.22 (6.2, 9.9)	348.2 (341.7, 355.7)	519.4 (374.4, 706.0)
r-3	True	12.48	5.00	0.2104	3.57	252.0	551.7
	CNF	11.77 (9.06, 13.11)	5.72 (4.43, 8.84)	0.2273 (0.20, 0.30)	3.75 (3.4, 4.9)	252.9 (248.2, 255.9)	463.6 (394.0, 584.3)
	PF	11.66 (10.62, 12.65)	5.88 (4.91, 7.08)	0.2284 (0.21, 0.25)	3.73 (3.5, 4.1)	252.8 (252.1, 255.1)	508.4 (419.0, 566.2)
r-4	True	16.43	5.00	0.2171	2.80	338.0	114.4
	CNF	16.74 (14.59, 17.93)	4.52 (3.54, 6.48)	0.2140 (0.20, 0.24)	2.71 (2.5, 3.1)	339.1 (335.2, 341.9)	89.1 (60.2, 127.7)
	PF	16.39 (15.21, 17.44)	4.62 (3.59, 5.62)	0.2177 (0.20, 0.23)	2.76 (2.6, 3.0)	339.2 (336.5, 341.8)	90.3 (59.4, 114.8)
r-5	True	4.61	5.00	0.2678	11.12	171.0	227.2
	CNF	6.17 (5.03, 7.15)	2.59 (1.53, 4.36)	0.1992 (0.17, 0.24)	8.42 (7.1, 10.0)	178.9 (168.6, 187.7)	174.7 (114.2, 256.2)
r-6	True	5.79	5.00	0.2102	0.93	337.0	497.2
	CNF	7.54 (1.57, 11.34)	3.73 (2.02, 10.09)	0.1594 (0.11, 0.78)	0.50 (0.3, 2.3)	332.5 (308.4, 358.4)	490.4 (275.8, 837.1)
	PF	6.83 (5.42, 8.72)	3.82 (2.12, 5.80)	0.1775 (0.14, 0.22)	0.61 (0.4, 0.8)	349.9 (322.6, 359.2)	471.7 (313.2, 619.4)
r-7	True	19.29	5.00	0.0917	5.15	142.0	748.2
	CNF	19.37 (15.98, 22.06)	4.60 (2.57, 7.94)	0.0902 (0.08, 0.11)	5.10 (4.5, 6.2)	138.0 (126.1, 148.5)	733.2 (627.0, 869.7)
	PF	19.08 (16.59, 20.44)	4.94 (3.65, 7.45)	0.0919 (0.09, 0.11)	5.24 (4.9, 6.0)	140.0 (127.3, 148.1)	741.6 (650.2, 855.0)
r-8	True	8.71	5.00	0.1219	9.21	167.0	940.2
	CNF	9.76 (7.99, 11.71)	3.64 (1.58, 5.64)	0.1093 (0.09, 0.13)	8.28 (6.8, 9.9)	182.7 (156.3, 214.3)	952.2 (780.0, 996.3)
	PF	9.44 (8.11, 11.06)	3.49 (1.86, 5.56)	0.1132 (0.10, 0.13)	8.48 (7.1, 9.8)	198.6 (160.7, 210.1)	917.4 (777.1, 993.0)
r-9	True	8.69	5.00	0.3018	0.93	265.0	135.1
	CNF	8.66 (4.22, 10.76)	4.45 (2.60, 9.26)	0.2985 (0.24, 0.62)	0.96 (0.8, 1.9)	268.6 (264.4, 276.5)	92.0 (55.2, 138.6)
	PF	8.65 (7.38, 9.74)	4.83 (3.66, 6.25)	0.3009 (0.27, 0.35)	0.95 (0.8, 1.1)	265.7 (263.1, 270.4)	107.6 (71.2, 151.0)
r-10	True	22.22	5.00	0.0525	1.61	147.0	428.9
	CNF	20.92 (10.03, 31.75)	4.97 (2.23, 16.04)	0.0577 (0.04, 0.12)	1.53 (1.0, 3.2)	213.2 (130.0, 336.4)	375.4 (192.1, 587.5)
	PF	17.12 (13.61, 24.04)	5.60 (2.28, 12.29)	0.0694 (0.05, 0.09)	1.90 (1.3, 2.4)	239.6 (134.4, 330.9)	298.2 (219.4, 489.1)

Table S7.2: **Effective sample sizes (ESS) per parameter and model.** ESS computed on the last 10,000 samples of the chains resulting from running the PF method on the full two-variant SEIR model with datasets for the reparametrized model and using a maximum lag size of 250 for the autocorrelation.

**Dataset**	$\boldsymbol{\gamma^{-1}}$	$\boldsymbol{\kappa^{-1}}$	$\boldsymbol{\beta}$	**s**	$\mathbf{t}_{\text{var}}$	**I0**
d-1-1	501.6	658.8	514.8	528.8	1174.2	1537.4
d-1-2	436.2	539.8	401.8	428.6	1493.4	1768.6
r-1	2311.4	2677.6	2238.3	3195.0	2227.8	3110.6
r-3	1548.5	1579.2	1494.0	2699.0	1491.8	2661.4
r-4	3040.3	2889.0	2968.4	3225.8	3037.0	2946.2
r-6	288.1	633.9	283.2	2000.6	524.8	1293.5
r-7	1777.9	1590.3	1786.5	2162.0	1777.5	2536.1
r-8	1140.3	1729.7	1196.5	1761.4	1260.4	2053.0
r-9	1032.7	1020.9	1011.3	2564.6	1120.1	2106.1
r-10	442.8	1415.0	418.2	1587.0	487.5	981.4

Table S7.3: **$\hat{\mathbf{R}}$ diagnostics for the full SEIR model with data for the reparametrized model.**

**Dataset**	$\boldsymbol{\gamma^{-1}}$	$\boldsymbol{\kappa^{-1}}$	$\boldsymbol{\beta}$	**s**	$\mathbf{t}_{\text{var}}$	**I0**
d-1-1	1.023	1.010	1.022	1.022	1.007	1.005
d-1-2	1.025	1.014	1.024	1.021	1.004	1.002
r-1	1.004	1.003	1.004	1.002	1.004	1.004
r-3	1.007	1.007	1.007	1.003	1.007	1.005
r-4	1.001	1.001	1.001	1.002	1.001	1.003
r-6	1.185	1.060	1.186	1.009	1.071	1.019
r-7	1.008	1.007	1.008	1.003	1.008	1.005
r-8	1.016	1.014	1.016	1.005	1.016	1.001
r-9	1.018	1.018	1.019	1.002	1.014	1.005
r-10	1.051	1.006	1.050	1.005	1.043	1.015
