# Supplementary material for: Assessment of simulation-based inference methods for stochastic compartmental models in epidemiological research
Source: PLoS One. 2026 Jul 13;21(7):e0353306. doi: 10.1371/journal.pone.0353306 (PMC13362117; doi:10.1371/journal.pone.0353306)
Supplement: S8 Results — (PDF) [file pone.0353306.s008.pdf]

S8 Supplementary Results SEIR-Model with Real Data
Assessment of Simulation-based Inference Methods for Stochastic Compartmental Models in Epidemiological Research

Vincent Wieland[1,2,◕], Nils Waßmuth[1,2,3,◕], Lorenzo Contento[1], Martin Kühn[1,2,3], and Jan Hasenauer[1,2,*]

[1]Bonn Center for Mathematical Life Sciences, University of Bonn, Bonn, Germany
[2]Life and Medical Science Institute, University of Bonn, Bonn, Germany
[3]Institute of Software Technology, Department for High-Performance Computing, German Aerospace Center (DLR), Cologne, Germany
◕These authors contributed equally to the work.
*To whom correspondence should be addressed; jan.hasenauer@uni-bonn.de.

June 26, 2026

Contents

S8.A Supplementary Figures 2
S8.B Supplementary Tables 4

S8.A Supplementary Figures

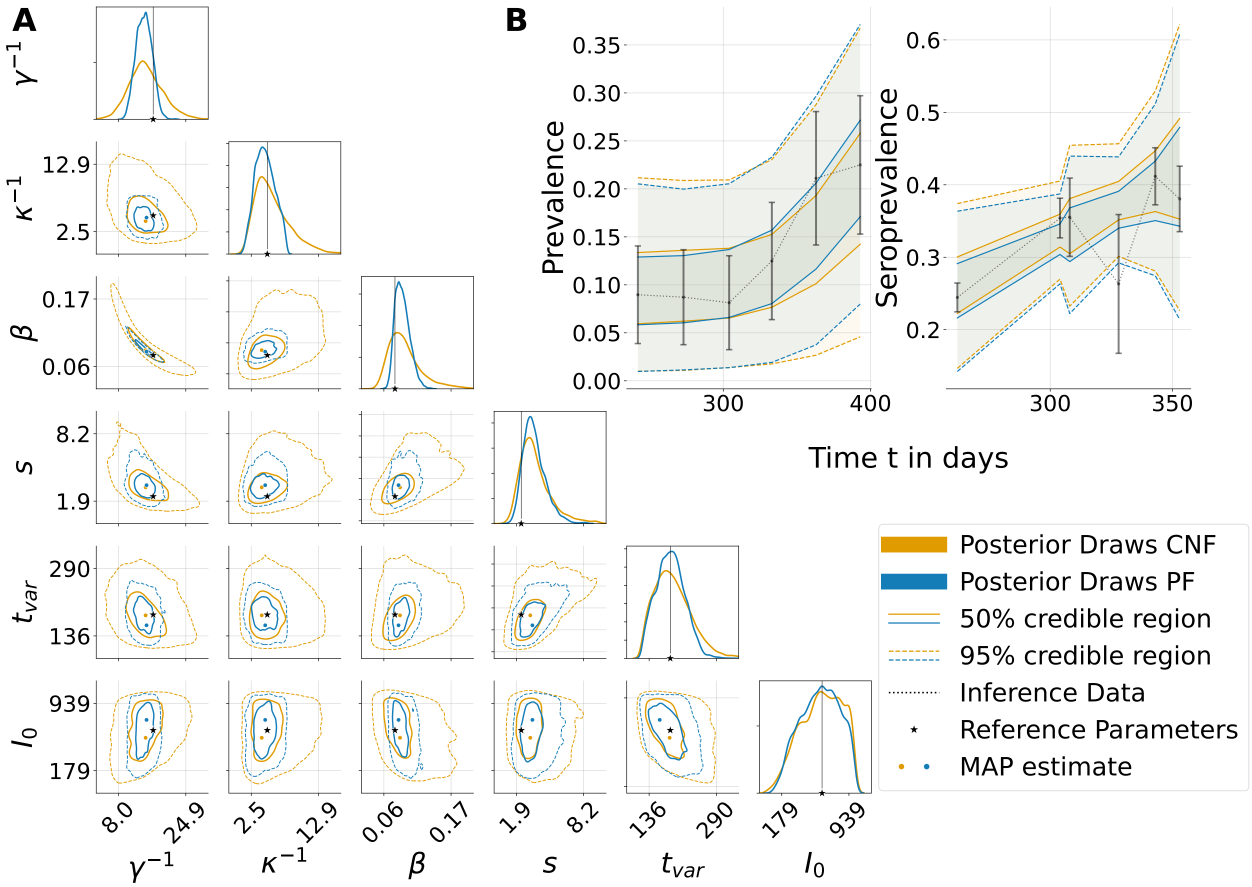

Figure S8.1: **Results of the two-variant SEIR model for *ethiopia*.**
A Posterior approximations from 10,000 samples. Contours give the 50% (solid) and 95% (dashed) credible regions, coloured by method. Diagonals show the 1D marginals. Black stars mark the publication parameters, coloured circles the joint MAP estimates. **B** Posterior predictive fit: bands give the 50% and 95% pointwise predictive intervals from the same samples (line styles as in **A**). The inference data (published data from [1]) are shown as a dotted line with error bars.

(*eth.*)

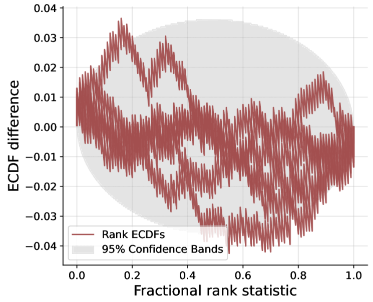

Figure S8.2: **ECDF Calibration plots for the full two-variant SEIR model with dataset *ethiopia*.**

(*eth.*)

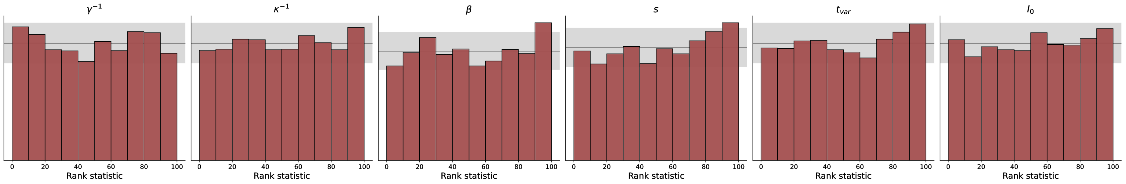

Figure S8.3: **SBC Histograms for the full two-variant SEIR model with dataset *ethiopia*.**

(*eth.*)

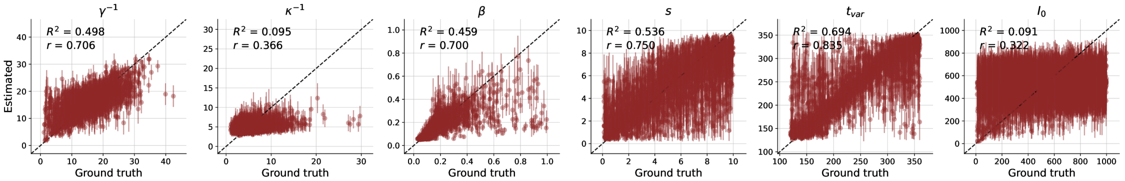

Figure S8.4: **Parameter recovery for the full two-variant SEIR model with dataset *ethiopia*.**

S8.B Supplementary Tables

Table S8.1: Posterior MAP estimates with 95% intervals for real-world data.

Dataset	Method	γ^{-1}	κ^{-1}	β	s	t_{var}	I_0
eth.	Pub.	16.7	5.0	0.08	2.3	184.5	635.0
	CNF	14.80 (7.23, 24.19)	4.14 (2.04, 12.95)	0.0882 (0.06, 0.18)	3.16 (1.5, 7.6)	183.0 (126.4, 290.3)	550.2 (173.0, 983.0)
	PF	15.03 (10.66, 18.22)	4.67 (2.00, 7.54)	0.0858 (0.07, 0.12)	3.36 (1.9, 6.0)	160.4 (128.0, 248.5)	752.1 (205.6, 972.4)

Table S8.2: **Effective sample sizes (ESS) per parameter and model for the real dataset.** ESS computed on the last 10,000 samples of the chains resulting from running the PF method on the two-variant SEIR model and using a maximum lag size of 250 for the autocorrelation.

**Dataset**	$\boldsymbol{\gamma^{-1}}$	$\boldsymbol{\kappa^{-1}}$	$\boldsymbol{\beta}$	**s**	$\mathbf{t}_{\text{var}}$	$\mathbf{I_0}$
eth.	423.8	1312.0	407.3	959.2	798.6	734.5

Table S8.3: **$\hat{\mathbf{R}}$ diagnostics for the SEIR model with real data.**

**Dataset**	$\boldsymbol{\gamma^{-1}}$	$\boldsymbol{\kappa^{-1}}$	$\boldsymbol{\beta}$	**s**	$\mathbf{t}_{\text{var}}$	$\mathbf{I_0}$
eth.	1.034	1.004	1.033	1.007	1.010	1.006

References

[1] Gudina EK, Ali S, Girma E, Gize A, Tegene B, Hundie GB, et al. Seroepidemiology and model-based prediction of SARS CoV 2 in Ethiopia: longitudinal cohort study among front-line hospital workers and communities. The Lancet Global Health. 2021;9(11):e1517-27. doi:10.1016/S2214-109X(21)00386-7.
