# Supplementary material for: Assessment of simulation-based inference methods for stochastic compartmental models in epidemiological research
Source: PLoS One. 2026 Jul 13;21(7):e0353306. doi: 10.1371/journal.pone.0353306 (PMC13362117; doi:10.1371/journal.pone.0353306)
Supplement: Attachment — Submitted filename: Review.pdf [file pone.0353306.s009.pdf]

Review for
„Assessment of Simulation-based Inference Methods for Stochastic Compartmental Models“

Summary

The authors study parameter inference for stochastic epidemic models using two probabilistic approaches. Focusing on stochastic SIR and extended SEIR models, they assess how particle filtering (PF) and conditional normalizing flows (CNF) approximate the posterior distribution. The methods are first evaluated on synthetic data and subsequently applied to real-world epidemiological observations from Ethiopia.

General Comments

The authors do a good job in setting the stage and introducing the problem and explaining the different SIR models. However their evaluation lacks in depth and most claims are not supported by the presented analysis. Their whole evaluation is based on very few (2-6) synthetic observation. But, as they even shown in the parameter recovery plots (e.g. Fig. S24), performance can vary vastly for different parameter settings. This should be taken into account and all analysis should be performed on an adequate set of (synthetic) datasets, commonly chosen as simulations based on samples from the prior.

While benchmarking likelihood-free inference methods is challenging, there are nevertheless tools to investigate the results in more depth. For the SIR model, I assume that the likelihood is even tractable and standard MCMC approaches could serve as a baseline to which the full posterior can be compared to (e.g. in terms of KL divergence, or classifier-two-sample test, or other measures). The authors oversimplify the comparison by mainly reporting RMSE, which can be done for the posterior predictive, bur which falls short in the parameter space, where only the MAP is considered. While SBC is an appropriate measure, the authors don’t take the failure in the simple SIR modle properly into account, and again refer to the predictive performance. However, a correct *posterior* should be the target of the inverse modelling approach. The claims of to wide or narrow posteriors (e.g. l.540) is therefore not supported by the analysis, as they never compare against a proper reference posterior, and is more anecdotally based on very few observations. Same holds true for other claims and their interpretations in their result sections are questionable and not backed up by proper analysis.

On a conceptual level, the authors claim to use an amortized inference method (CNF), however they train it from scratch for every observation (if I understood it correctly). While this might make sense for different data shapes (dense vs sparse data), it is unclear why this is done even in same simulator and data settings.

On a technical levels, a lot of information is missing, e.g. details to subsample the sparse datasets (e.g. data shapes), exact usage of prior (and confusing „upper“ and „lower“ limits), the exact evaluation schemes, the reparametrization (which fixes on parameter (?) and therefore changes the problem) etc. Also CNFs are trained for a fixed, limited number of epochs with a small network capacity (in terms of number of summary statistics / hidden layer size). This can clearly decrease the performance and additional control experiments should be performed and the convergence of the networks investigated.

Overall, while it looks like the authors have built a nice set-up to solve the inverse problem for stochastic versions of the SIR model, their evaluation pipeline is limited and their claims are not properly backed-up by the presented experiments. Therefore I recommend to reject the paper. Below I attach more specific remarks, which may help the authors to improve their manuscript.

Specific Remarks:

1. The intro to SBI is quite rough and can be a bit more elaborated. Additionally, it misses credits to some of the foundational works on SBI, e.g. the whole work from the *sbi* community is not mentioned (Boelts et al. 2024). Some specific points:
    A. l. 88: neural posterior methods are only one class of SBI methods. See Cranmer et al. or Deistler et al. (2025) for a categorization. Consequently l. 88 ff are not a complete picture of the SBI methods.
    B. l. 90 ff: Missing/wrong citation. Papamakarios et al. (2016) were the first in this line of work, and importantly Pampamakarios et al. (2021) (or any of his preceding papers) which invented and developed NFs.
    C. l. 95 ff: There has been (by today rather outdated) effort to benchmark different ABC/ SBI methods, which even covers a version of an SIR model and SMC-ABC (Lueckmann et al. 2021). So this claim is a bit misleading.
    D. The missing credits also holds true for Section 3.2.
2. Title is a bit misleading and should mention some SIR/epistemic model.
3. l. 125: While it is intuitively clear what is happening, the formal description of a compartmental model could be improved.
4. Fig. 1 a,b: Caption should be (almost) self-containing, e.g. explain *SIER, wt, var.*
5. l. 168: Why is kappa fixed? Isn’t this a free parameter of the model which is inferred from data?
6. Tab. 2: Variant recovery: where does the 1.35 come from? This is implementing the „prolonged infectious period“, but shouldn’t this also be inferred from data?
7. l. 204: Typically Bayesian inference is interested in the posterior distribution rather than the „best explain[ing]“ parameters. Which is explained later. The authors should adapt also here.
8. l. 233: Again „conditional formalizing flows“ seems to be restrictive here, as there are many other conditional density estimators. The authors don’t have to explain them all, but at least mention that they are only considering this specific sub-class.
9. Algo 2: Best practice is to optimize the DNF until convergence on a validation dataset, not for a fixed number of epochs T.
10. Fig. S3, b,d: Missing y-label. For how many particles is the ancestor tree? C: title cut off.
11. l. 355ff: CNF: the presented setting might be sub-optimal. Can the authors ensure that the network did already converge after 100 epochs? A latent dimension (dim of summary stats) of 2n+2 seems quite small, as the data (observations) are compressed into this dimension. Also, even a single Gaussian has (n^2+n)/2 free parameters. However, at this point it is unclear to me how large the observation space is for the different observational settings.
12. l. 360 ff: Handling missing data: What is the reasoning of implementing an approach for missing and sparse data if the neural networks are only trained on this specific pattern? Or did I misunderstood l. 365? For similarly structured training and test data, no additional processing is necessary.
13. l. 378: For CNF the MAP can directly be obtained from the trained network by maximizing the (log-) posterior probability, which is not introducing an additional approximation via kernels.
14. L 402. Referring to (almost) all suppl. tables in one place doesn’t make sense.

15. Section 5: It is not clear at this point what the dimension of the observation space is in the different settings.
16. Fig. 2: Ground-truth parameter should also be included into the 2d marginal.
17. Fig. 4: it is common to also scale the 2d marginals to the prior bounds/ meaningful prior support regions. Otherwise the information gain across dimensions is hard to judge.
18. Suppl. Figures should be arranged according to the main text.
19. L. 451: should reference to Fig. S25
20. Fig. 6 „True parameter“ is misleading. There is no true parameter here. What are the black uncertainty intervals in B? These are not defined.
21. L. 616: for flow matching Wildberger et al. (2023) might be of interest for the authors.
22. L. 558: There are already a bunch of SBI methods dealing with these flexible settings, e.g. Gloeckler et al. (2024) or Moss et al. (2023). While I included some references below, I don't expect the authors to cite them in their manuscript. But I want to make them aware of research related to their topic, which might be of interest.
23. Supporting Tables need a thorough proofreading. There is a lot of missing/repeated information. They should also be arranged in the order as appearing in main.
24. Tab. S20: „Dense 1-1“ and „Dense 1-2“ are the same, but information for „Dense 2-1“ is missing?
25. Table S15: all values are the same. What is the subsampling ratio?
26. Table S27: What are the different priors referring to? The computation time comparison is not fair, as different resources are used.
27. Table S3: what are the upper and lower bounds for beta? Why are they dependent on gamma and why is the prior then Unif(0,1)? Please clarify.
28. Evaluation:
    A. results solely rely on two (!) parameter settings for each model.
    B. l. 381: RMSE is used in parameter and data space. While this is fine conceptually (but see comments above), the authors should make this more explicit.
    C. SBC plots are just provided in the supplement without e.g. explaining what ECDF means. SBC is clearly off for SIR, but in different ways for different parameters. Why? There seems to be an oscillating behavior in the SBC plots, why? A deeper analysis is missing here.
    D. RMSE should include some measure of uncertainty, e.g. std. or quantiles.

References

- *Papamakarios, G. and Murray, I. Fast ε-free inference of simulation models with Bayesian conditional density estimation. Advances in neural information processing systems, 29, 2016.*
- *Deistler, M., Boelts, J., Steinbach, P., Moss, G., Moreau, T., Gloeckler, M., Rodrigues, P. L., Linhart, J., Lappalainen,, . K., Miller, B. K., et al. Simulation-based inference: A practical guide. arXiv preprint arXiv:2508.12939, 2025.*
- *Papamakarios G, Nalisnick E, Rezende DJ, Mohamed S, Lakshminarayanan B. Normalizing flows for probabilistic modeling and inference. Journal of Machine Learning Research. 2021;22(57):1-64.*
- *Lueckmann JM, Boelts J, Greenberg D, Goncalves P, Macke J. Benchmarking simulation-based inference. InInternational conference on artificial intelligence and statistics 2021 Mar 18 (pp. 343-351). PMLR.*

- *Boelts J, Deistler M, Gloeckler M, Tejero-Cantero Á, Lueckmann JM, Moss G, Steinbach P, Moreau T, Muratore F, Linhart J, Durkan C. sbi reloaded: a toolkit for simulation-based inference workflows. arXiv preprint arXiv:2411.17337. 2024 Nov 26.*
- *Wildberger J, Dax M, Buchholz S, Green S, Macke JH, Schölkopf B. Flow matching for scalable simulation-based inference. Advances in Neural Information Processing Systems. 2023 Dec 15;36:16837-64.*
- *Gloeckler M, Deistler M, Weilbach C, Wood F, Macke JH. All-in-one simulation-based inference. arXiv preprint arXiv:2404.09636. 2024 Apr 15.*
- *Moss G, Muhle LS, Drews R, Macke JH, Schröder C. FNOPE: Simulation-based inference on function spaces with Fourier Neural Operators. arXiv preprint arXiv:2505.22573. 2025 May 28.*
