# Supplementary material for: Assessment of simulation-based inference methods for stochastic compartmental models in epidemiological research
Source: PLoS One. 2026 Jul 13;21(7):e0353306. doi: 10.1371/journal.pone.0353306 (PMC13362117; doi:10.1371/journal.pone.0353306)
Supplement: Attachment — Submitted filename: Response to Reviewers.pdf [file pone.0353306.s013.pdf]

Author’s response to reviewers’ comments

The authors thank the reviewers and the editor for their constructive comments. Based on their remarks, we have updated the manuscript. We addressed all of their comments and tried to resolve the raised issues. The following points were changed in the manuscript:

- We added a new small example model, the SIS-model, into the considered experimental setups.
- We computed a baseline posterior for the SIS and the SIR model using state-of-the-art Hamiltonain Monte Carlo sampling and compared both methods against this baseline.
- We introduce the Wasserstein distance to evaluate the difference of estimated posterior distributions in the parameter space.
- We changed the RMSE to a proper energy score to evaluate the posterior predictive performance.
- We grounded the assessment on more than 4-times the number of simulated datasets.
- We streamlined the introduction of stochastic compartmental models and added more details on the PMMH algorithm.
- We added more details for the data generating process and used evaluation metrics.

In addition to these changes in the content, we updated the structure and the section titles as well as the title of our proposed work:

- Manuscript title was changed to ”Assessment of Simulation-based Inference Methods for Stochastic Compartmental Models in Epidemiological Research”
- Splitting the supplementary information into several section corresponding to the different result sections.
- Combined several figures and removed redundant supplementary tables.

Our point-by-point responses to the single points raised by the reviewers are provided below.

- Response to Reviewer 1
- Response to Reviewer 2

The teal boxes contain the reviewers’ comments, while the yellow boxes contain the changes made in the manuscript. Within the yellow boxes, we have marked the changed part in red. To provide some context, the comments (teal) are surrounded by unchanged text (black).

Response to Reviewer 1

Comment 1: Page 2, lines 78–79: In pseudo-marginal MCMC (PMMH), particle filters are used to estimate the likelihood, not to propose parameters. The wording in this sentence should be revised accordingly.

Answer: We thank the reviewer for pointing out the misleading formulation and changed the wording accordingly.

Changes in the manuscript (Section 1):

These methods, also known as particle filters (PF), have been used to produce an unbiased likelihood estimate for ~~generate parameter proposals of~~ an MCMC algorithm,

Comment 2: Normal approximation of the Bernoulli distribution (A.2 Supplementary information): How is the parameter p (success probability) chosen in the normal approximation used to compute the standard deviation in each case? It would be helpful to report the resulting standard deviation for each analysis conducted. Additionally, how do the authors handle numerical instability in situations where the case count takes the value zero?

Answer: We thank the reviewer for raising this point. In all analyses, the success probability p used in the normal approximation is taken directly from the generative model: for serological observations, p corresponds to the true underlying seroprevalence at the observation time; for case counts, p corresponds to the model-implied infection probability. Thus, the standard deviation $\sqrt{np(1-p)}$ is fully determined by the known population size n and the model-generated value of p.
To avoid numerical instabilities and a degenerative behavior in Particle Filters, we discarded trajectories where the variance died out completely.

Changes to Section 2:

From each of the ~~two~~ stochastic compartmental models we generated ~~synthetical~~synthetic data emulating the time course of a disease outbreak. We generated dense datasets with observations every seven or ten days. We used two types of parameter regimes:

1. hand-selected values chosen to represent plausible epidemiological regimes, and
2. parameters sampled directly from the corresponding priors as an unbiased baseline

Prior draws that generated trajectories with collapsed variance were discarded and resampled to maintain numerical stability of the particle filter. Additionally, to create realistic, heterogeneous observation schedules, we randomly subsampled the dense time series into sparse datasets reducing the number of time points from 57 to fewer than 15 per observable. We also introduced partial missingness in the sparse datasets, where only one of the two observables was available at certain time points. ~~Details of the data generation and different datasets used are outlined in Supplementary Information S1.3.2.~~Details on the computation of measurement noise and data generation are provided in the **Models** section of the Supplementary Information (Section S1.3.)

Comment 3: Section 3.1 (Particle Markov Chain Monte Carlo Methods): Although this section is entitled Particle Markov Chain Monte Carlo Methods, it mainly describes the particle filter, with only a brief mention of how it is embedded within the Metropolis–Hastings algorithm. The authors could improve clarity by including pseudo-code for the PMMH algorithm (with acceptance probability), explicitly showing how the likelihood is replaced by its unbiased estimate in Equation (5).

Answer: We thank the reviewer for this advise. The use of the unbiased likelihood estimate in the Metropolis-Hastings algorithm is an important part of the method. However, we think the particle filter itself is the core aspect of the "simulation-based" and less known to readers. Therefore, we added the pseudo-code of the full PMMH algorithm in the supplementary information. This keeps the main part of the manuscript streamlined and avoids too much overhead, by showing two pseudo-code algorithms for one method, while providing all details to the interested reader.

Changes in the manuscript (Section 3.1):

The full PMMH-algorithm is provided in the Supplementary Information A.4.

Changes in the manuscript (Supplementary Information):

S1.1.1 PMMH algorithm

Particle Filters are a general tool to replace the intractable likelihood $p(y_{t_0:t_M}|\theta)$ by an unbiased estimate $\hat{p}(y_{t_0:t_M}|\theta)$ inside a Metropolis-Hastings (MH) algorithm by using the acceptance probability

$$\min\left\{1, \frac{\pi(\theta^k)}{\pi(\theta^{k-1})}\frac{\hat{p}(y|\theta^k)}{\hat{p}(y|\theta^{k-1})}\frac{q(\theta^{k-1}|\theta^k)}{q(\theta^k|\theta^{k-1})}\right\}, \tag{1}$$

where $q(\cdot|\theta)$ denotes the parameter proposal distribution from the MH algorithm.
The PMMH algorithm with a generic Particle Filter is shown in pseudocode in Algorithm 1, where we denote the estimate to the likelihood obtained from a particle filter by $L_{t_M}^N(\theta)$.

PMMH

Require: parameter space Θ, number of iterations K, parameter proposal distribution $q(\cdot|\theta)$, initial distributions $\pi(x_0), \pi(\theta)$, Particle Filter and necessary input for it (e.g., number of particles N, observations $y_{t_0:t_M}$)

Sample $\theta^0 \sim \pi(\theta)$
Run Particle Filter to generate variables $L_{t_m}(\theta_0)$
for $k = 1$ to K **do**
  Sample $\tilde{\theta} \sim Q_\Theta(\theta^{k-1})$
  Run Particle Filter to generate variables $L_{t_M}(\tilde{\theta})$
  Sample $u \sim \mathcal{U}([0,1])$
  Set $v = \log[r_{PMMH}(\theta^{k-1}, \tilde{\theta})]$ with
  $r_{PMMH}(\theta^{k-1}, \tilde{\theta}) := \frac{\pi(\tilde{\theta}) L^N_{t_M}(\tilde{\theta}) q(\theta^{k-1}|\tilde{\theta})}{\pi(\theta^{k-1}) L^N_{t_M}(\theta^{k-1}) q(\tilde{\theta}|\theta^{k-1})}$
  if $\log(u) \leq v$ **then**
    Set $\theta^k := \tilde{\theta}$
  else
    Set $\theta^k := \theta^{k-1}$
  end if
end for

Comment 4: Algorithm 1, Step 6: No information is given about the effective sample size (ESS), although this is discussed in the supplementary material. A brief explanation or reference in the main text would be helpful.

Answer: We added a reference to the corresponding section in the supplementary information.

Changes in the manuscript (Section 3.1):

In practice, the resampling is not done in every iteration, but only if the variability of the weights becomes too large, as measured by the effective sample size (for more details see Supplementary Information S1.1.2.2.

Comment 5: Algorithm 1, Step 7: In the resampling step, the weights should be $W_{t_{i-1}}$, since W_{t_i} is not yet available at that stage.

Answer: We thank the reviewer for catching this typo and fixed it accordingly in the main manuscript.

Comment 6: RMSE results in Tables 5–6 and supplementary tables: In some cases, the RMSE of incidence/prevalence computed using the known ground-truth parameters is higher than that obtained using PF or CNF estimates in the simulated data, which appears counterintuitive. Could the authors explain why this occurs? Additionally, how exactly is the RMSE computed when using the true parameters?

Answer: We thank the reviewer for raising this point. The observation that the RMSE obtained using the true parameters can occasionally be higher than the RMSE obtained from PF or CNF estimates is not contradictory. In our simulation setup, the "true-parameter RMSE" is computed by drawing 10,000 stochastic trajectories from the model using the ground-truth parameters and averaging their RMSE relative to the realized dataset. Because the epidemic trajectories are stochastic, the ground-truth parameters do not necessarily generate trajectories that are

closest to the particular realization used for inference. In some cases, PF or CNF may return parameter estimates that, by chance, produce trajectories that align more closely with the specific observed realization, even though these estimates are not closer to the underlying generative parameters.

This effect is a known limitation of RMSE-based comparisons in stochastic dynamical systems, as RMSE measures closeness to a single realization rather than to the underlying data-generating mechanism. For this reason, we have replaced RMSE with the energy score of the posterior predictive distribution, which evaluates predictive accuracy in a principled, distribution-aware manner. The Supplementary Information now includes a description of the energy score and the Monte Carlo error of its numerical estimate.

Comment 7: Figure 6 caption: The term “true parameters” is not appropriate, as true parameter values are not available for real data. The authors could instead use “reference parameters”.

Answer: We thank the reviewer for pointing is out. The term ”true parameter” is indeed misleading. We changed this to ”Publication Parameter” to make clear that we compare against the value from the reference publication. We also defined the uncertainty intervals in the figure caption.

Changes in Section 5:
Evaluation of the posterior approximations for the two-variant SEIR model with real data.
A Posterior approximations based on 10,000 samples, ~~true parameters~~ publication parameters and their reported standard deviation are indicated by bold black lines and joint MAP estimates appear in a darker shade. **B** Model fit with percentiles (50%, 90%, 95%) based on simulating the 10,000 samples.

Comment 8: Page 18, lines 580–593 (computational time): CPU times are only reported for the SIR model. Providing CPU times for the SEIR model (with two variants), both for simulated and real data, would further support the claims made in this paragraph.

Answer: Thank you for this suggestion. We reported accumulated CPU-times for the SIR and also SEIR model with two variants in Table S27 in the Supplementary Information and referenced this in the corresponding results sections for the SIR and SEIR model.

Comment 9: Prior distribution for the SIR model (Table S3): The prior specification for β is confusing. The table states that the prior is $\mathcal{U}(0,1)$, but also indicates a lower bound of $0.95 \times \gamma$. Since the lower bound of γ is 1, this would imply $\beta \sim \mathcal{U}(0.95, 1)$. On the other hand, if the prior is truly $\mathcal{U}(0,1)$, it is unclear how a MAP (PF or CNF) estimate of approximately 0.1 (which is less than $0.95 \times \gamma$) is obtained. My guess is that the lower and upper bounds refer to the normal prior mentioned in Table S27. The authors should clearly define and reconcile these prior specifications.

Answer: We thank the reviewer for pointing out this confusion. The issue arises from a notational mismatch: as γ is restrained to the interval $[1, 30]$, the lower bound for β is $0.95/30 \approx 0.03$. Our intention was to use uninformed priors, but in the considered models a large portion of the naive prior support leads to unrealistic or uninformative trajectories. In particular, the effective reproduction number $R_0 = \beta\gamma^{-1}$ becomes either extremely small or extremely large for many combinations of β and γ^{-1}, which results in trajectories that are either almost flat or explode unrealistically. To avoid placing prior mass on such pathological regimes, we imposed simple constraints on β that depend on γ^{-1}, ensuring that R_0 remains within a plausible epidemiological range.
The prior is therefore written as Unif(0,1) to indicate that we do not impose any additional shape or information beyond these feasibility constraints; the effective bounds shown in Table S3 arise solely from enforcing biologically

reasonable values of R_0.
For the SEIR2V model, we additionally restrained γ^{-1} to be greater than one for numerical stability. This is also consistent with domain knowledge, we deemed an infectious period shorter than one day as epidemiologically implausible.
We incorporated this in the manuscript as follows:

Changes in Supplementary Information:

S1.3.1 Prior Bounds and Feasibility Constraints

In addition to the stated priors, we impose simple feasibility constraints to ensure that simulated trajectories remain within a plausible epidemiological range. These constraints are based on the basic reproduction number $R_0 = \beta\gamma^{-1}$ which becomes unrealistically small or large for many naive combinations of β and γ^{-1}. To avoid such pathological regimes, we restrict the admissible values of β for a given γ^{-1} by repeatedly resampling β until the resulting R_0 lies within a reasonable range.
We additionally constrain the infectious period γ^{-1} to be greater than one day. This avoids numerical instability in the simulation and is consistent with epidemiological knowledge, as infectious periods shorter than one day are implausible for the diseases considered here.

Response to Reviewer 2

1. The authors do a good job in setting the stage and introducing the problem and explaining the different SIR models. However their evaluation lacks in depth and most claims are not supported by the presented analysis. Their whole evaluation is based on very few (2-6) synthetic observation. But, as they even shown in the parameter recovery plots (e.g. Fig. S24), performance can vary vastly for different parameter settings. This should be taken into account and all analysis should be performed on an adequate set of (synthetic) datasets, commonly chosen as simulations based on samples from the prior. This should be taken into account and all analysis should be performed on an adequate set of (synthetic) datasets, commonly chosen as simulations based on samples from the prior. While benchmarking likelihood-free inference methods is challenging, there are nevertheless tools to investigate the results in more depth.
For the SIR model, I assume that the likelihood is even tractable and standard MCMC approaches could serve as a baseline to which the full posterior can be compared to (e.g. in terms of KL divergence, or classifier-two-sample test, or other measures)

Answer: We thank the reviewer for this comment and suggestion how to improve the reliability of our results. Indeed, a broader synthetic evaluation and, where possible, comparison to a tractable likelihood would strengthen the analysis. Below we clarify the constraints of our setting and the concrete additions we included in the revised manuscript in 5 points.

1. **Tractability of the likelihood**
   Due to the stochastic nature, the likelihood of SDE-based compartmental models requires integration over Brownian motions and is therefore not tractable. Even the standard SIR model induces a two-dimensional Fokker–Planck equation on a triangular domain with state-dependent diffusion and reflecting boundaries. This cannot be solved analytically and numerical solutions (using the Python package FiPy) of the Fokker–Planck equation were also infeasible. Although FiPy provides implicit and semi-implicit solvers that are not subject to the classical explicit CFL stability restriction, resolving the PDE still requires a fine spatial grid due to the state-dependent diffusion and sharp gradients near the boundaries of the triangular domain. However, reducing the spatial grid size Δx effectively forces a reduction in the time-step size Δt as large Δt leads to severe temporal discretization error and produces poorly conditioned linear systems, causing the solver to diverge or stagnate. In practice, the required spatio-temporal resolution is prohibitively fine, making repeated numerical solutions (to solve over the full prior support) far too expensive for use within our inference pipeline. As such that we could not find a closed-form transition density. Consequently, exact-likelihood MCMC is not feasible.

2. **Baseline posterior**
   To circumvent the use of the exact marginal likelihood, we treat the diffusion path between observation times as missing data and approximate it using fine time-discretization in the Euler-Maruyama scheme. We then applied Hamiltonian Monte-Carlos on the resulting joint density over parameters and latent path increments and use this as a baseline posterior distribution, to which we can compare both methods.

Changes in Section 5:
~~We begin with the standard SIR model (Section 2.2) using a synthetic prior to validate both methods.~~ To assess the performance of both inference methods against a baseline posterior distribution, we use a standard SIS (Section 2.1) and SIR (Section 2.2) model with a synthetic prior. The baseline posterior was computed on a finely discretized version of the SDE using a state-of-the-art Hamiltonian Monte Carlo sampler [1](Supplementary Information S1.1.4).

Changes in Supplementary Information:

S1.1.4 HMC for SDE-based models

Likelihood-based inference for discretely observed diffusion processes is challenging because the likelihood depends on the transition density, which is rarely available in closed form for nonlinear, multivariate SDEs [2]. As a consequence, "direct" MCMC on the exact marginal likelihood is typically infeasible. A standard remedy is *data augmentation* in which the unobserved diffusion path between observation times is treated as missing data and approximated by a time discretization [3, 4]. This produces an explicit (approximate) joint density over parameters and latent path increments, enabling proper MCMC—and in particular gradient-based methods such as Hamiltonian Monte Carlo (HMC)—to be applied. Different strategies to propose diffusion path segments are discussed in [5] and more recently, improved bridge constructs and guided proposals were developed [6, 7]. For the SDE model at hand we used the transition densities from the Euler-Maruyama scheme on a fine-time discretization. We model the epidemic dynamics in terms of population fractions $x(t)$ (e.g. $x(t) = (s(t), i(t))^\top$ for SIS or $x(t) = (s(t), i(t), r(t))^\top$ for SIR) as an Itô SDE

$$dx(t) \;=\; f\big(x(t), \theta\big)\, dt \;+\; G\big(x(t), \theta\big)\, dB(t), \tag{2}$$

where θ contains epidemiological parameters (e.g. infection rate β, recovery rate γ), and $B(t)$ is a standard Brownian motion of suitable dimension.

The system is observed at irregular times $t_1 < \cdots < t_m$. First, we construct an augmented time grid $\tau_1 < \tau_2 < \cdots < \tau_K$ that contains all observation times and (optionally) intermediate points such that $\Delta\tau_k = \tau_{k+1} - \tau_k \le h_{\max}$. On this grid we approximate (2) with the Euler–Maruyama (EM) scheme:

$$x_{k+1} \;=\; x_k \;+\; f(x_k, \theta)\, \Delta\tau_k \;+\; G(x_k, \theta)\, \Delta B_k, \qquad \Delta B_k \sim \mathcal{N}(0, \Delta\tau_k I). \tag{3}$$

Writing $\Delta B_k = \sqrt{\Delta\tau_k}\, \varepsilon_k$ with $\varepsilon_k \sim \mathcal{N}(0, I)$ yields

$$x_{k+1} \;=\; x_k \;+\; f(x_k, \theta)\, \Delta\tau_k \;+\; G(x_k, \theta)\, \sqrt{\Delta\tau_k}\, \varepsilon_k. \tag{4}$$

This reparameterization makes the driving noise $\varepsilon_{1:K-1}$ explicit and i.i.d. standard Normal, so the latent path $\{x_k\}_{k=1}^K$ becomes a deterministic function of the initial condition, parameters θ, and the innovation sequence $\varepsilon_{1:K-1}$. The resulting posterior is an *approximation* to the continuous-time diffusion posterior whose accuracy improves as $h_{\max} \to 0$ [4] and crucially, for fixed (θ, ε) the latent path and the log posterior are deterministic, avoiding random simulation inside the likelihood [4].

We sample from this posterior using HMC with the No-U-Turn Sampler (NUTS) [1] as implemented in *Turing.jl*[8]. Gradients of the log posterior are obtained via automatic differentiation through the EM recursion and observation likelihood. Moreover, we ensured the convergence of the HMC chains by using standard MCMC convergence criteria as the Gelman-Rubin $\hat{R}$ statistic, autocorrelation and effective sample size.

3. **SIS model**
   Due to the already complex likelihood structure of the SIR-model, we added a more simple model to our assessment. For this we chose the the basic SIS-model and compared both simulation-based methods against the baseline HMC posterior on it. Although the associated Fokker–Planck equation is one-dimensional, numerical solutions remain too expensive to construct a likelihood-based posterior baseline. Nevertheless, due to the reduced model complexity, the HMC-based baseline posterior is more trustworthy and serves as a useful benchmark for evaluating the performance of the simulation-based methods.

Changes in Section 2:

2.1 SIS Model

The simplest compartmental model is the SIS-model [9] consisting of one *Susceptible* compartment S and one *Infectious* compartment I, where people can transition between them (Figure 1(a)). This model can be used for infectious diseases that do not confer any long-lasting immunity, such as a common cold or influenza. Following simple mass-action incidence individuals get infected with a rate βSI and leave the infective class at rate γI (Table 1).

Table 1: Description of the SIS model. Table including all events, such as initialization and transition of individuals, with their respective rate and model parameters.

**Event**	**Description**	**Rate**	**Parameter**
Initialization	Set initial number of infected individuals	-	I_0
Infection	$\mathrm{S} \rightarrow \mathrm{I}$	$\beta \frac{\mathrm{S}(t)\mathrm{I}(t)}{\mathrm{N}(t)}$	β
Recovery	$\mathrm{I} \rightarrow \mathrm{S}$	$\gamma I(t)$	γ^{-1}

Changes to Section 5 (Addition of SIS)

5.1 CNF and PF Agree With A Reference Posterior For The SIS Model

We evaluated convergence and posterior estimates for the SIS model using CNF, PF, and a Hamiltonian Monte Carlo (HMC) baseline applied to a finely discretized version of the SDE. Across 10 synthetic dense datasets, all methods achieved excellent marginal coverage and produced highly consistent Wasserstein distances. Two datasets (sis-2 and sis-4) exhibited unusually large distances and were excluded from the aggregate analysis but even for these outliers, visual posterior differences remained minor.

After exclusion, the in-distribution Monte Carlo baseline (HMC–HMC) was small, with values ranging from 0.056 to 0.119. Between-method distances were only moderately larger: CNF–PF ranged from 0.063 to 0.156, CNF–HMC ranged from 0.065 to 0.176 and PF–HMC ranged from 0.058 to 0.124. Interpreted on the log-parameter scale, these values correspond to average multiplicative shifts of roughly $6\% - 13\%$ for the baseline and $6\% - 17\%$, $7\% - 19\%$, and $6\% - 13\%$ for the between-method comparisons, respectively. These results indicate that systematic differences between inference algorithms are small, consistent with the strong visual overlap in marginal and joint posteriors (Figure 2A). Posterior predictive simulations from all three methods reproduced the observed epidemic trajectories with comparable accuracy (Figure 2B), as evidenced by consistent energy scores computed from the posterior predictive distributions of the methods and the ground truth (Table 2).

In terms of sampling behavior, PF showed excellent convergence, with ESS values above 2000 and $\hat{\mathbf{R}}$ near 1. CNF showed good calibration in simulation-based calibration diagnostics, with near-uniform rank histograms and accurate parameter recovery. Overall, both PF and CNF matched the predictive and inferential performance of the HMC reference.

Full diagnostics, including ESS, $\hat{\mathbf{R}}$, Wasserstein distance results, calibration plots, and parameter-recovery analyses are provided in Supplementary Information S2.

Table 2: Predictive Energy Score for the SIS model.

	sis-1	*sis-2*	*sis-3*	*sis-4*	*sis-5*
True	0.034	0.051	0.028	0.019	0.024
CNF	0.035	0.046	0.025	0.018	0.024
PF	0.034	0.046	0.025	0.018	0.023
HMC	0.035	0.046	0.025	0.018	0.023
	sis-6	*sis-7*	*sis-8*	*sis-9*	*sis-10*
True	0.041	0.025	0.045	0.042	0.041
CNF	0.040	0.027	0.044	0.035	0.040
PF	0.037	0.022	0.042	0.031	0.040
HMC	0.037	0.022	0.042	0.031	0.039

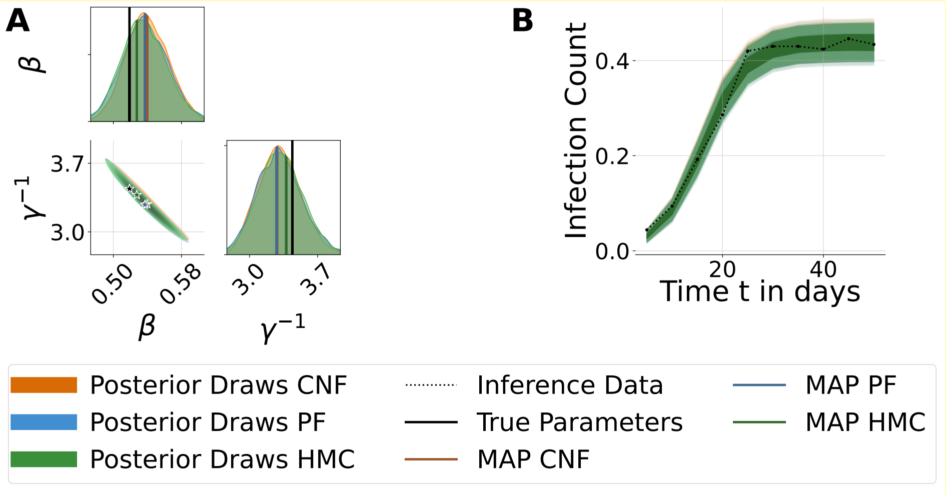

Figure 2: **Evaluation of the posterior approximations for the SIS model**
A Posterior approximations based on 10,000 samples, true parameters are indicated by bold black lines and joint MAP estimates appear in a darker shade. **B** Model fit with percentiles (50%, 90%, 95%) based on simulating the 10,000 samples.

4. **Number of synthetic datasets.**
We agree that performance varies across parameter settings and that a wider sweep over the prior is desirable. Such a sweep is limited by the computational cost of particle filtering ($\approx$ 2000 CPU-minutes per run), which constrained large-scale prior-predictive evaluations. In the revision, we increase the number of synthetic datasets for all models to more than ten, ensuring that variability across the prior is better represented.

Changes to Section 2:
From each of the ~~two~~ stochastic compartmental models we generated synthetical data emulating the time course of a disease outbreak. We generated dense datasets with observations every seven or ten days. To achieve unbiased data, we sampled the necessary parameters from the relevant priors. We discarded trajectories where variance died out completely to guarantee numerical stability for PF. Additionally, to create realistic, heterogeneous observation schedules, we randomly subsample the dense time series into sparse datasets reducing the number of time points from 57 to fewer than 15 per observable. We also introduced partial missingness in the sparse datasets, where only one of the two observables was available at certain time points. Details of the data generation and different datasets used are outlined in Supplementary Information S1.3.2.

Changes to Section 4:
For the comparison of both methods we used visual and quantitative measures. The visual comparison using violin and density plots of the marginal posteriors allows comparison of the shape and variability of the posterior approximations. This is complemented by a visual comparison of the model fit. For the evaluations we draw 10,000 parameter vector samples from each approximate posterior distribution and, for each parameter vector, simulate an observable trajectory to create an ensemble. We then plot the central 50%, 90%, and 95% intervals of the ensemble against the data. These are independent posterior predictive samples, obtained by evaluating $p(y \mid \theta)$ for draws $\theta \sim p(\theta \mid \mathcal{D}^*)$. As an additional qualitative indicator, we report the maximum a-posteriori (MAP) estimate computed via Gaussian kernel density estimation using Silverman's rule [10], which provides a consistent definition across methods. For a quantitative measure of inference accuracy, we compute the maximum a-posteriori (MAP) estimate in the parameter space via Gaussian kernel density estimation using Silverman's rule [10]we first assess marginal coverage by reporting how frequently the true generative parameters fall within the 95% marginal posterior intervals across datasets. We also quantify posterior differences using the 1-Wasserstein distance in log-parameter space. All Wasserstein distances are obtained by repeatedly subsampling 1,000 draws per posterior, and we report in-distribution values to characterize the Monte Carlo error of this procedure. Heuristically, the 1-Wasserstein distance in log-parameter space measures the average multiplicative shift required to transform one posterior into another, providing an interpretable notion of scale-adjusted discrepancy. Concretely, we compute these distances in three settings:

- **Baseline comparisons:** when a baseline posterior is available, we measure the Wasserstein distance between each method and the baseline and compare it to the corresponding in-distribution reference.
- **Model-parameterization comparison:** for the full and reparametrized SEIR2V models, we evaluate how the Wasserstein distance changes when keeping the dataset fixed, again reporting the in-distribution values as a baseline.
- **Within-method variability:** for one representative parameter set, we assess the variability of each method by computing Wasserstein distances between multiple PF runs or multiple CNF trainings.

Finally, we assess predictive accuracy via the energy score of the posterior predictive distributions. as well as the root-mean squared error (RMSE) Specifically, the RMSE was calculated for trajectories simulated from each of the 10,000 posterior samples and then averaged, providing an estimate of the expected RMSE under the posterior distribution. For synthetic experiments, the RMSEpredictive energy score was also computed with respect to the true parameters used to generate the data. For the real-data experiment, true parameters are not available. Instead, we report RMSEthe energy score relative to published parameter estimates from [11], which serve as a reference baseline.

2. The authors oversimplify the comparison by mainly reporting RMSE, which can be done for the posterior predictive, but which falls short in the parameter space, where only the MAP is considered. While SBC is an appropriate measure, the authors don't take the failure in the simple SIR model properly into account, and again refer to the predictive performance. However, a correct posterior should be the target of the inverse modeling approach. The claims of to wide or narrow posteriors (e.g. l.540) is therefore not supported by the analysis, as they never compare against a proper reference posterior, and is more anecdotally based on very few observations. Same holds true for other claims and their interpretations in their result sections are questionable and not backed up by proper analysis.

Answer: We thank the reviewer for suggesting further enhancements of our assessment. We agree that evaluating

likelihood-free inference requires going beyond MAP estimates and RMSE, and that the posterior (and not only its predictive implications) is the primary target. Below we clarify how the revised manuscript addresses these concerns by using baseline posterior distributions for the SIS and SIR model, a Wasserstein distance between posterior distributions in the parameter space and exchanging the RMSE by a proper energy score.

1. **Posterior comparisons.**
   In the original submission, RMSE was used only for posterior-predictive evaluation, and MAP estimates were reported in parameter space. We agree that this is insufficient for assessing posterior quality. In the revision, we therefore:

   - replace RMSE with the multivariate energy score for posterior-predictive evaluation, see for example Table 6 below
   - compute 1-Wasserstein distances between posterior samples (PF, CNF, HMC) on the log-scale to quantify discrepancies directly in parameter space, see example Figure S3.13 below
   - complement these metrics with ESS, $\hat{\mathbf{R}}$ and SBC to jointly assess sampling quality and calibration.

Changes to Section 5.2:
Marginal and joint posterior distributions overlapped closely (Figure3A) with CNF exhibiting a small systematic offset to HMC and PF, which we attribute to its mild posterior miscalibration, consistent with its mild posterior miscalibration discussed below. This pattern is reflected in the Wasserstein distances. The in-distribution Monte Carlo baseline (HMC–HMC) ranged from 0.034 to 0.096. Between-method distances were modestly larger: CNF–PF ranged from 0.063 to 0.165, CNF–HMC from 0.076 to 0.183 and PF–HMC ranged from 0.048 to 0.125. Interpreted on the log-parameter scale, these values correspond to average multiplicative shifts of approximately 3–10% for the baseline and 6–18%, 8–20%, and 5–13% for the between-method comparisons, respectively. Detailed results are provided in Supplementary Figure S3.13. Posterior predictive checks showed that epidemic trajectories generated from ~~either method~~all three methods fit the observed data equally well (Figure 3B)~~. MAP estimates from CNF and PF were nearly identical and close to the true parameters, with RMSEs for infection counts and seroprevalence matching those obtained from simulations with the ground truth~~ , as evidenced by the close agreement in predictive energy scores (Table 6).

Table 6: Predictive Energy Score for the SIR model.

	sir-1	*sir-2*	*sir-3*	*sir-4*	*sir-5*	*sir-6*
True	0.046	0.057	0.032	0.020	0.052	0.021
CNF	0.046	0.040	0.033	0.033	0.052	0.018
PF	0.045	0.039	0.033	0.025	0.054	0.020
HMC	0.045	0.039	0.032	0.024	0.054	0.021

	sir-7	*sir-8*	*sir-9*	*sir-10*	*sir-11*	*sir-12*
True	0.050	0.054	0.056	0.071	0.061	0.073
CNF	0.048	0.053	0.057	0.069	0.061	0.066
PF	0.047	0.052	0.057	0.070	0.062	0.060
HMC	0.047	0.053	0.057	0.069	0.061	0.060

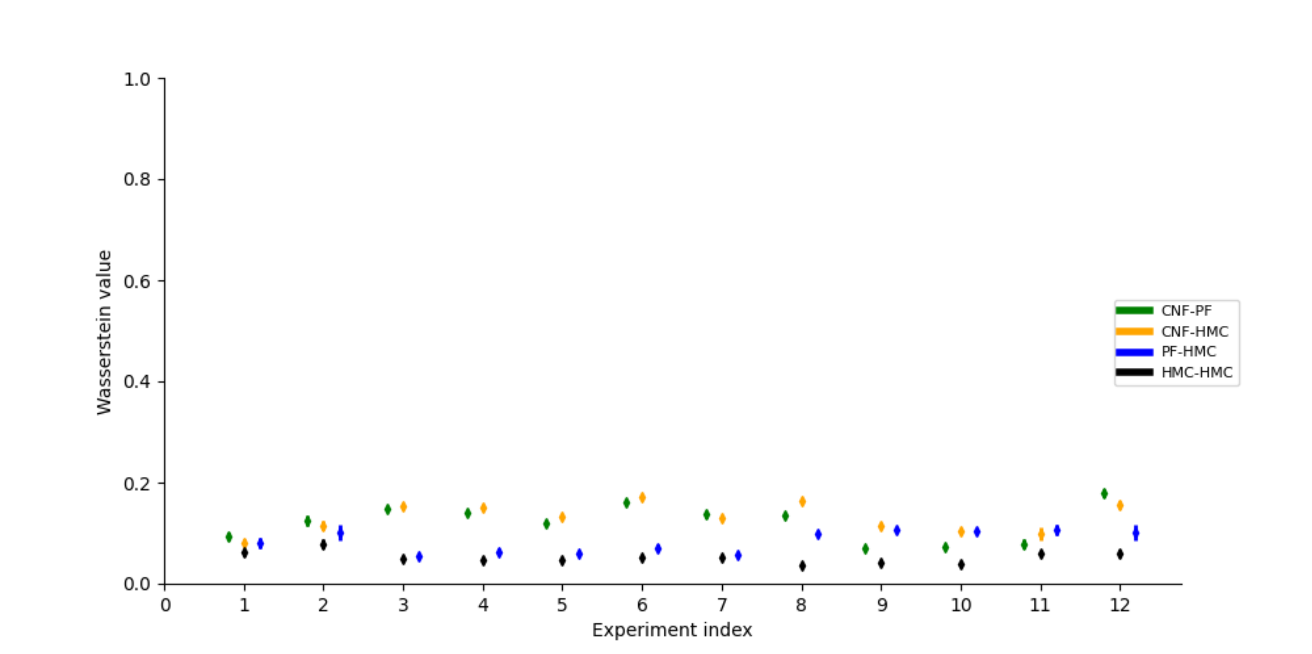

Figure S3.13: Wasserstein distances for the SIR model

Pairwise 1-Wasserstein distances between posterior samples across experiment indices for all method combinations. Error bars show Monte-Carlo variability.

Changes to Section 5.3:

This behavior is typical in models where certain parameters are weakly informed by the data (see [12] for a more thorough discussion). The PF posterior approximation showed a higher contraction than that of CNF. Additionally, it was also largely enveloped in the CNF posterior approximation, across parameters (Fig. 5A).PF posteriors were generally more concentrated than those of CNF and were largely enveloped by the CNF approximation across parameters (Fig. 5A). Despite these width differences, both methods generally include the true parameters in their posteriors, with CNF achieving 95.2% marginal coverage and PF achieving 91.7%. E epidemic trajectories generated from both methods fit the data equally well (Fig. B)with comparable energy scores , and MAP estimates together with RMSE scores were close to the true parameters (Table 7). PF estimates were marginally closer to the ground truth, highlighting the method's focus on trajectory agreement through resampling. PF produced marginally lower energy scores, highlighting the method's focus on trajectory agreement through resampling. As a secondary analysis, we assessed calibration and convergence diagnostics.The CNF–PF distances were comparable to the variability across repeated CNF runs, suggesting that differences between the two methods remain close to the stochastic variability inherent in CNF..

. . . Full diagnostics, including calibration plots, convergence statistics, Wasserstein-distances and parameter-recovery analyses, are provided in Supplementary Information S4 (dense datasets) and S5 (sparse datasets).

Changes to Section 5.4:

In the reparametrized setting, p~~P~~osterior distributions from CNF and PF showed close alignment across parameters, indicating similar exploration of the parameter space (Figure 6A). Unlike in the full SEIR2V model, the posterior approximations exhibited no signs of long-tailed behaviour. This is reflected in the 1-Wasserstein distances in log-parameter space, which were numerically smaller under reparametrization and decreased more strongly than the within-method baselines (CNF–CNF and PF–PF). Moreover, CNF–PF distances were of the same order as the variability observed across independent runs of each method, indicating markedly improved cross-method alignment. ~~Both methods yielded comparable MAP estimates near the true values, with minor deviations for c_0 and I_0, where PF shifted toward lower values.~~ Epidemic trajectories fit the data equally well (Figure 6B). ~~and RMSE values were similar across CNF, PF, and the true parameters, with PF showing marginally better agreement on the infection count data~~ with energy scores between CNF, PF, and the true parameters being close to each other in both model settings (Table 9, Table 10)).

Table 9: Predictive Energy Score for the reparametrized SEIR2V model with dense data.

	d-1-1	*d-1-2*	*r-1*	*r-2*	*r-3*	*r-4*
True Param.	0.1133	0.1153	0.0973	0.1386	0.0986	0.0997
CNF 0.1098	0.1066	0.0832	0.1176	0.1003	0.1035	
PF 0.1074	0.1043	0.0799	0.1163	0.0935	0.1052	
	r-5	*r-6*	*r-7*	*r-8*	*r-9*	*r-10*
True Param.	0.1455	0.1206	0.1463	0.1226	0.0913	0.0628
CNF 0.1264	0.1166	0.1418	0.0980	0.0818	0.05803	
PF 0.1277	0.1059	0.1384	0.0991	0.0792	0.0587	

Table 10: Predictive Energy Score for the full SEIR2V model with dense data from the reparametrized setting.

	d-1-1	*d-1-2*	*r-1*	*r-2*	*r-3*	*r-4*
True Param.	0.1132	0.1148	0.09754	0.1388	0.09826	0.09969
CNF	0.108	0.1067	0.09269	0.1227	0.1004	0.1063
PF	0.1062	.1035	0.07961	–	0.09438	0.1005
	r-5	*r-6*	*r-7*	*r-8*	*r-9*	*r-10*
True Param.	0.1456	0.1203	0.146	0.124	0.09142	0.06312
CNF	0.1327	0.1127	0.1445	0.09898	0.08096	0.05773
PF	–	0.1059	0.1382	0.09892	0.0796	0.05737

2. **On the SIR miscalibration.**
We agree that SBC failures in the SIR model must be taken seriously. In the revision, we explicitly analyze how this miscalibration arises and how it relates to the reference posteriors:

- We compare CNF posteriors directly to PF and to HMC on a finely discretized SDE, which serves as a gold-standard reference (see comment above).
- We show that the SBC deviations correspond to a systematic shift relative to these baselines.
- We clarify how this shift propagates into the predictive distribution, linking posterior miscalibration to its downstream effects.

Changes in the manuscript Section 5.2 (old Section 5.1):
~~Both CNF and PF yielded highly consistent posterior estimates for the SIR model. The marginal and joint posterior distributions overlapped almost perfectly (Figure 3A), and p~~ Across all datasets, the three posterior approximations PF and CNF showed high agreement with HMC. Marginal and joint posterior distributions overlapped closely (Figure 3A) with CNF exhibiting a small systematic offset to HMC and PF, which we attribute to its mild posterior miscalibration; we return to this point in the calibration analysis below. This pattern is also reflected in the Wasserstein distances: PF and HMC showed the closest agreement, while CNF was slightly farther from both, though all distances remained small in absolute terms.
...
CNF, in contrast, showed deviations in SBC ~~calibration~~ diagnostics (Supplementary Figure S3.14), reflecting limitations of the amortized posterior. Notably, these calibration issues were not apparent in the parameter recovery plots: recovered parameters aligned closely with the ground truth and error bars remained narrow (Supplementary Figure S3.16). Still, the miscalibration geometry generally matches the offset of CNF relative to PF and HMC. For example, β typically has an upward sloped SBC histogram which corresponds a left-shift in the β marginal. This highlights that while CNF's posterior uncertainty is imperfectly calibrated in a formal sense, the practical impact on point estimates and predictive accuracy is minimal.

3. On a conceptual level, the authors claim to use an amortized inference method (CNF), however they train it from scratch for every observation (if I understood it correctly). While this might make sense for different data shapes (dense vs sparse data), it is unclear why this is done even in same simulator and data settings.

Answer: Thank you for this question. In our setting, each synthetic dataset is generated with its own noise level and therefore its own data shape. Concretely, for every dataset we:

- draw parameters from the prior,
- simulate a trajectory from the SDE model
- pass this trajectory through a binomial observation model (500 test subjects),
- and perform inference under a Gaussian noise model with the corresponding standard deviations.

Because the observation noise scale varies continuously across datasets, the inference problem is not tied to a fixed discrete set of data shapes. Amortising over all possible noise levels would require amortisation over a continuous parameter, which is neither practical nor aligned with the intended use case. For this reason, we train the CNF separately for each dataset, even when the underlying simulator is the same.

At the same time, the method retains the key advantages of amortisation in the dimensions that are shared across datasets:

- the CNF amortises over all latent trajectories consistent with a given dataset,
- it amortises over the full prior and thus all parameter values,
- and it amortises over the simulator dynamics, which remain fixed across datasets.

This structure is precisely what enables simulation-based calibration (SBC) to be applied efficiently: once trained, the CNF can be evaluated repeatedly on simulated datasets without retraining, allowing us to run SBC at scale and diagnose posterior miscalibration directly. In the revised manuscript, we clarify that the per-dataset training reflects the continuously varying observation model, while the amortised structure of the CNF is what

makes SBC feasible and informative in our setting.

Changes in the manuscript (Section 3):
This amortization not only enables fast inference once training is complete, but also facilitates simulation-based calibration (SBC) diagnostics by allowing posterior approximations to be generated efficiently across many simulated datasets. At the same time, performance of CNFs is tied to the support of the training distribution: the model can interpolate to new values if they are close to the training support, but performance degrades when extrapolating far beyond it [13, 14]. In practice, this amortized structure is precisely what makes SBC feasible at scale, since once trained, the CNF can be evaluated repeatedly on simulated datasets without retraining.

Changes in the manuscript (Section 4):
For CNF, posterior approximations were obtained via neural posterior estimation with learned summary statistics [15], using the BayesFlow software (v1) for amortized Bayesian workflows [16]. We trained an independent neural network for each experiment rather than a single globally amortized model. This preserves amortization over latent trajectories and parameter values within each experiment, while avoiding the need to amortize over continuously varying noise levels across experiments, a setting in which global amortization would be inefficient and unnecessary. The resulting per-experiment amortization still enables efficient SBC diagnostics and fast posterior evaluation once training is complete.

4. On a technical levels, a lot of information is missing, e.g. details to subsample the sparse datasets (e.g. data shapes), exact usage of prior (and confusing "upper" and "lower" limits), the exact evaluation schemes, the reparametrization (which fixes on parameter (?) and therefore changes the problem) etc. Also CNFs are trained for a fixed, limited number of epochs with a small network capacity (in terms of number of summary statistics / hidden layer size). This can clearly decrease the performance and additional control experiments should be performed and the convergence of the networks investigated.

Answer: We Thank the reviewer for adressing this. We have restructured the Supplementary Information and expanded it to give more technical details on dataset generation, priors and evaluation schemes for all used models. See also the answer to comment 5.27 for the evaluation scheme description and the Supplementary Information file S1, for the full model descriptions. As for performance and convergence of the networks, we investigated this in detail via a hyperparameter search (together with reruns to assess stability) and examination of validation- and training loss curves. One part of the hyperparameter search was to assess in detail, how the summary dimension affects the output. We considered summary dimension sizes $2n+2$, $4n$ and $8n$ and did not find a benefit for higher summary dimension sizes. We give more details in the answers to the specific comments 5.5, 5.6 and 5.11. We changed the manuscript as follows:

Changes to Section 2: The model exhibits non-identifiability issues in the sense that different parameter combinations, lying along certain curves, can generate nearly indistinguishable epidemic trajectories (see Supplementary Information S1.3.6.2). Therefore, in addition to this model, we also consider a reparametrized version of the model with a reduced number of model parameters to be estimated. For this, wWe fix $\kappa^{-1} = 5$ ~~κ^{-1}~~ since κ seems to participate in the non-identifiable parameter combinations and use the following redefined parameters

$$r_0 = \beta \cdot \gamma^{-1}, \quad e_0 = \kappa^{-1} + \gamma^{-1}, \quad s_0 = s \cdot \gamma^{-1}, \tag{5}$$

while keeping t_{var} and I_0 as well as the observational and noise models unchanged. This transformation allows a comparisons of the inference methods CNF and PF in either parameter space without altering the underlying system.

Changes to Section 4:~~To ensure comparability, all experiments used identical hyperparameters and simulation budgets, determined through a preliminary hyperparameter search~~. Rather than fine-tuning each method for every individual experiment, which would confound performance differences with tuning effort, we selected CNF hyperparameters through a principled search procedure that we repeated to assess stability. We verified that training was well-behaved by inspecting training and validation loss curves, which consistently stabilized well before 100 epochs.

Changes to Supplementary Information:

S1.3 Models

This section describes the stochastic compartmental models used in our simulation study, together with their prior distributions, feasibility constraints, and model-specific data-generation procedures. We consider three model classes: SIS, SIR, and the two-variant SEIR model (SEIR2V) in both its full and reparameterized forms.

S1.3.1 Prior Bounds and Feasibility Constraints

In addition to the stated priors, we impose simple feasibility constraints to ensure that simulated trajectories remain within a plausible epidemiological range. These constraints are based on the basic reproduction number $R_0 = \beta\gamma^{-1}$ which becomes unrealistically small or large for many naïve combinations of β and γ^{-1}. To avoid such pathological regimes, we restrict the admissible values of β for a given γ^{-1} by repeatedly resampling β until the resulting R_0 lies within a reasonable range.
We additionally constrain the infectious period γ^{-1} to be greater than one day. This avoids numerical instability in the simulation and is consistent with epidemiological knowledge, as infectious periods shorter than one day are implausible for the diseases considered here.

S1.3.2 General Information on Data Generation

This section summarizes the components of the data-generation process that are shared across all models in our simulation study. These include the observational model, the noise assumptions, and the construction of dense and sparse observation schedules. Model-specific data-generation settings—such as parameter regimes, compartmental transitions, and any additional constraints—are provided in the corresponding model subsections below.
Across all models (SIS, SIR, and SEIR2V in both its full and reparameterized forms), we generated multiple synthetic datasets using dense observation schedules as the baseline, with sparse datasets derived where appropriate to mimic realistic epidemiological reporting. All datasets were generated under the requirement that simulated trajectories retain non-degenerate variance. Observations follow the measurement model described in Section 2.4 and are perturbed by binomial noise. For inference, we approximate this binomial noise using a Gaussian model; the rationale and computation of this approximation are detailed in the following subsection.

S1.3.3 Computation of standard deviation for the noise model

When conducting Bernoulli trials, such as testing individuals in a population, the sum of positive test is adequately modeled by a binomial distribution, provided the trials are independent and the success probability remains constant. If the number n of such Bernoulli trials conducted is large or the success probability p is neither exceedingly low nor high, the resulting binomial distribution $B(n, p)$ is well approximated by a normal distribution $\mathcal{N}(np, np(1-p))$, thanks to the de Moivre-Laplace Theorem, a special case of the central limit Theorem [17]. This approximation allows us to compute the standard deviation of the measurement noise directly from the count data and the known population size, and to incorporate it into the noise model. For our setups, the success probabilities are described in Section 2.4 of the main manuscript.
However, this approximation may become inaccurate for small sample sizes or highly skewed probabilities, where alternative methods or exact distributions might be preferable. When generating our synthetic data, we chose $n = 500$, which is sufficiently large to justify the Gaussian approximation.

S1.3.4 SIS

...

S1.3.4.3 Data generation

For the SIS model we drew ten parameter sets from the prior (subject to the non-degenerate variance constraint), listed in Table S1.8. From each set we generated one densely sampled dataset, with observations every 5 days from day 5 to day 50. Observations consist of a single observable, the infection count. The ten datasets are labeled *sis-1* to *sis-10*.

S1.3.5 SIR

...

S1.3.5.3 Data generation

For the SIR model we generated twelve parameter sets using two regimes:

1. Two hand-crafted parameter sets designed to mimic epidemiological settings.
2. Ten parameter sets drawn from the prior (again under the non-degenerate variance constraint), providing an unbiased foundation for our comparisons.

The parameter sets are listed in Table S1.12. From each set, we generated one densely sampled dataset, with observation points every 10 days from 10 to 100. Observations consist of two distinct observational functions: infection count and seroprevalence. This results in twelve datasets, labeled *sir-1* to *sir-12*, with *sir-1* and *sir-2* corresponding to the hand-crafted parameter sets.

S1.3.6 SEIR2V

...

S1.3.6.3 Data generation

SEIR2V (full) For the full SEIR2V model we generated twelve parameter sets using three regimes:

1. One hand-crafted parameter set based on the results for the SEIR2V model in [11].
2. One hand-crafted parameter set designed to mimic epidemiological settings.
3. Ten parameter sets drawn from the prior (again under the non-degenerate variance constraint), providing an unbiased foundation for our comparisons.

The resulting datasets and their associated ground-truth parameter values are listed in Table S1.17. For each of the two hand-crafted parameter sets, we generated two densely sampled datasets (four datasets total), labeled *d-1-1*, *d-1-2*, *d-2-1*, and *d-2-2*. Each of the ten prior-drawn parameter sets produced one densely sampled dataset, corresponding to *d-3* through *d-12*. This yields fourteen dense datasets in total.
To mimic realistic data sparsity, we constructed sparse datasets from the dense datasets as follows:

- From dense datasets *d-1-1* and *d-1-2*, which correspond to the publication-based parameter set, we generated three sparse datasets each using the following procedure:
  1. draw three integers specifying the number of infection-count timepoints, seroprevalence timepoints, and jointly observed timepoints;
  2. randomly subsample the dense dataset accordingly.
- From dense datasets *d-3* to *d-12*, which correspond to the ten parameter sets drawn from the prior, we generated one sparse dataset per dense dataset by randomly subsampling 8 infection-count observation times and 8 seroprevalence observation times.

This yields sixteen sparse datasets in total. The six sparse datasets derived from *d-1-1* and *d-1-2* are labeled *s-1-1-1* to *s-1-1-3* and *s-1-2-1* to *s-1-2-3*, respectively. The ten sparse datasets derived from *d-3* to *d-12* are labeled *s-3* to *s-12*. Table S1.18 provides the corresponding ground-truth parameter values for all sparse datasets.

SEIR2V (reparametrized) For the reparameterized SEIR2V model we generated eleven parameter sets using two regimes:

1. One hand-crafted parameter set based on the results for the SEIR2V model in [11]. Under the transformation in (5), this parameter set corresponds directly to the publication-based parameter set used for the full SEIR2V model.

2. Ten parameter sets drawn from the prior (again under the non-degenerate variance constraint), providing an unbiased foundation for our comparisons.

For the publication-based parameter set, we reused the same simulated trajectories as in the full model, namely the dense datasets *d-1-1* and *d-1-2*. For each of the ten prior-drawn parameter sets, we generated one densely sampled dataset. Observations consist of two distinct observational functions (infection count and seroprevalence), recorded every 7 days from day 7 to day 399.
In total, this yields twelve densely sampled datasets: the two reused datasets *d-1-1* and *d-1-2*, followed by ten newly generated datasets labeled *r-1* to *r-10*. Table S1.19 provides the ground-truth parameter values for the reparameterized model.
Additionally, to enable direct comparison between the reparameterized and full SEIR2V models, we transformed each reparameterized parameter set into its corresponding full-model parameterization using (5). Table S1.20 lists the resulting full-model parameter values.

Publication Data For an application of the methods to real-world data, we use data from an longitudinal cohort study in Ethiopia[11]. As reference parameters we use the parameter set reported therein. This dataset is labeled as *eth.*.

5. Overall, while it looks like the authors have built a nice set-up to solve the inverse problem for stochastic versions of the SIR model, their evaluation pipeline is limited and their claims are not properly backed-up by the presented experiments. Therefore I recommend to reject the paper. Below I attach more specific remarks, which may help the authors to improve their manuscript.

5.1 The intro to SBI is quite rough and can be a bit more elaborated. Additionally, it misses credits to some of the foundational works on SBI, e.g. the whole work from the sbi community is not mentioned (Boelts et al. 2024). Some specific points:

- l. 88: neural posterior methods are only one class of SBI methods. See Cranmer et al. or Deistler et al. (2025) for a categorization. Consequently l. 88 ff are not a complete picture of the SBI methods.
- l. 90 ff: Missing/wrong citation. Papamakarios et al. (2016) were the first in this line of work, and importantly Pampamakarios et al. (2021) (or any of his preceding papers) which invented and developed NFs.
- l. 95 ff: There has been (by today rather outdated) effort to benchmark different ABC/ SBI methods, which even covers a version of an SIR model and SMC-ABC (Lueckmann et al. 2021). So this claim is a bit misleading.
- The missing credits also holds true for Section 3.2

Answer: We thank the reviewer for raising this issue. We have changed the manuscript as follows:

Changes to the Introduction: Additionally, as these methods require massive numbers of simulations, more recent machine learning approaches have been adapted for simulation-based inference with neural networks [18]~~, namely neural posterior estimation methods~~. These promising techniques often circumvent hand-crafted ~~try to circumvent~~ summary statistics and directly approximate the distribution of the parameters from samples generated by the simulator. Therein, conditional normalizing flows (CNF) are an advanced method that uses invertible neural networks to generate samples from the posterior distribution of the parameters [19, 20].~~parameters [15].~~

Changes to Section 3.2: Neural posterior estimation (NPE) is a simulation-based inference (SBI) method which frames posterior construction as a (conditional) density-estimation problem[19]. Given a simulator that produces samples $(\mathcal{D}, \theta)$ by first drawing $\theta \sim p(\theta)$ and then $\mathcal{D} \sim p(\mathcal{D} \mid \theta)$, NPE fits a conditional neural network $p_\phi(\theta \mid \mathcal{D})$ to approximate the true posterior $p(\theta \mid \mathcal{D})$. Typically the data $\mathcal{D}$ are preprocessed by a (learned) summary statistic [15], which serves as the input to the conditional density estimator. For clarity, this preprocessing step is omitted from the notation below.
In this work, we focus on Conditional Normalizing Flows (CNFs) [21]. Normalizing flows have since been substantially developed and systematized [20, 22, 16].

5.2 Title is a bit misleading and should mention some SIR/epistemic model.

Answer: We agree with the reviewer that a more precise title can help to clarify the scope of the paper. We therefore included the area of application in the title.

Changes in the manuscript (Title)
Assessement of Simulation-based Inference Methods for Stochastic Compartmental Models in Epidemiological Research.

5.3 l. 125: While it is intuitively clear what is happening, the formal description of a compartmental model could be improved.

Answer: We think the reviewer for suggesting this enhancement. We made the description more formal and mathematically detailed.

Changes in the manuscript (Section 2)
Such a model partition a population into d_X mutually exclusive compartments and is characterized by the vector of compartment sizes $X \in \mathbb{R}^{d_X}$ together with the transitions of individuals between compartments. ~~A transition from compartment i to compartment j can be described in terms of the stochiometric coefficient $s_{i,j} \in \mathbb{R}^{d_x}$, the vector of changes to the compartment sizes, and the rate at which the transition happens~~

$$\sout{a^N_{i,j}(X) = cX_i \sum_{k \in I_{i,j}} \frac{X_k}{N},}$$

~~where c denotes a positive parameter, $I_{i,j}$ is an index set containing all indices $k \neq i$ of compartments involved in that transition, and N gives the total number of individuals in the population. Let $\mathcal{T}$ denote the set of all tuples (i, j), such that a transition from X_i to X_j exists.~~ Let $\mathcal{T}$ denote the set of admissible transitions. Each transition $(i, j) \in \mathcal{T}$ is characterized by a stoichiometric vector $s_{i,j} \in \mathbb{Z}^{d_X}$, which specifies the change in the compartment counts when the transition occurs. For the movement of one individual from compartment i to compartment j, $s_{i,j} = e_j - e_i$, where e_i denotes the i-th unit vector. The transition intensity is described by a propensity function $a^N_{i,j} : \mathcal{S}_N \to \mathbb{R}_+$, defined through

$$\Pr\big(X^N(t+h) = x + s_{i,j} \mid X^N(t) = x\big) = a^N_{i,j}(x)\, h + o(h), \qquad h \downarrow 0.$$

For infection-driven transitions, a common choice is a mass-action propensity of the form

$$a^N_{i,j}(X) = c_{i,j} X_i \sum_{k \in I_{i,j}} \frac{X_k}{N},$$

where $c_{i,j} > 0$ is a rate parameter and $I_{i,j}$ contains the compartments contributing to the force of infection. Other transitions, such as recovery, are typically modeled by linear propensities, for example $a^N_{i,j}(x) = c_{i,j} x_i$.
The resulting stochastic compartmental model is a continuous-time Markov jump process. In the large-population regime, it can be approximated by the multivariate stochastic differential equation (SDE) [23]:

5.4 Fig. 1 a,b: Caption should be (almost) self-containing, e.g. explain SEIR, wt, var.

Answer: We thank the reviewer for pointing this out. We have amended the caption as follows:

Changes to Section 2
Figure 1: Schematic representations of the models and methods. (a) Graph of compartments and possible transitions with corresponding rate parameters for the Susceptible - Infected - Susceptible (SIS) model. ~~(a)~~(b) Graph of compartments and possible transitions with corresponding rate parameters for the Susceptible - Infected - Recovered (SIR) ~~SIR~~ model. ~~(b)~~(c) Graph of compartments and possible transitions with corresponding rate parameters for the ~~two-variant SEIR~~ Susceptible-Exposed-Infected-Recovered (SEIR) model with virus variants wild-type (**wt**) and variant (**var**). ~~(c)~~(d) Workflow for the assessment of the Bayesian inference

5.5 l. 168: Why is kappa fixed? Isn't this a free parameter of the model which is inferred from data?

Answer: We thank the reviewer for this question. We fix κ^{-1} to 5, to define a reduced formulation of the SEIR2V model. In the revised manuscript, we clarify that fixing κ^{-1} is not an ad-hoc, but a deliberate choice. The corresponding changed section of the manuscript is included in the answer to comment 4.

5.6 Tab. 2: Variant recovery: where does the 1.35 come from? This is implementing the "prolonged infectious period", but shouldn't this also be inferred from data?

Answer: We agree that this choice is rather obscure and thank the reviewer for pointing this out. The factor 1.35 is not an ad-hoc modelling choice on our side. It is part of the original two-variant SEIR model introduced in [11], where the novel variant is assumed to have an infectious period 35% longer than the wild-type. Our goal in this section is to reproduce that published model as faithfully as possible so that PF and CNF can be compared under the same assumptions.To reflect this, we changed the manuscript as follows:

Changes in the manuscript (Section 2.2)
~~The novel variant is more potent in the sense that it shows a prolonged infectious period~~ The novel variant is assumed to have a 35% longer infectious period, following the original formulation of the two-variant SEIR model in [11]. We adopt this fixed factor to remain consistent with the published model on which our comparison is based. Additionally, ~~and that~~immunity from the wild-type variant does not protect against reinfection with the novel variant.

5.7 l. 204: Typically Bayesian inference is interested in the posterior distribution rather than the "best explaining" parameters. Which is explained later. The authors should adapt also here

Answer: We adapted this in the manuscript as follows:

To infer the unknown model parameters θ~~, which best explain the~~given data $\mathcal{D}^* = \{\overline{y}_{t_i} \mid i \in \{1, ..., n\}\}$, we apply Bayesian inference techniques.

5.8 l. 233: Again "conditional formalizing flows" seems to be restrictive here, as there are many other conditional density estimators. The authors don't have to explain them all, but at least mention that they are only considering this specific sub-class

Answer: We made this more clear in the manuscript.

In this section, we will introduce two approaches (*particle filters* [24] and one particular class of conditional density estimators, *conditional normalizing flows* [19, 18] ~~[18]~~) that circumvent the computation of the likelihood function.

5.9 Algo 2: Best practice is to optimize the CNF until convergence on a validation dataset, not for a fixed number of epochs T.

Answer: We thank the reviewer for pointing out this weakness in the current presentation. We amended the algorithm in the revised manuscript such that it is clear that we train happens until convergence of the neural network.

Changes to Section 3

Require: Data source S (pre-simulated dataset or simulator callable $\sim p(\theta)p(\mathcal{D} \mid \theta)$), base density $q(z)$, conditional flow $f_\phi(\cdot; \mathcal{D})$, optimizer Opt, learning rate η, batch size N, ~~iterations T,~~ inference data $\mathcal{D}^*$, inference sample count M

Ensure: Trained parameters ϕ; sampler for approximate posterior $p_\phi(\theta \mid \mathcal{D}_*)$

~~**for** $t = 1$ to T~~
repeat
    $\{(\theta_i, \mathcal{D}_i)\}_{i=1}^N \leftarrow$ sample from S (dataset or simulator)
    for $i = 1$ to N **do**
        $z_i \leftarrow f_\phi(\theta_i; \mathcal{D}_i)$
        $\log p_\phi(\theta_i \mid \mathcal{D}_i) \leftarrow \log q(z_i) + \log \left| \det \left(\frac{\partial f_\phi(\theta_i; \mathcal{D}_i)}{\partial \theta} \right) \right|$
    end for
    $\mathcal{L} \leftarrow -\frac{1}{N} \sum_{i=1}^N \log p_\phi(\theta_i \mid \mathcal{D}_i)$
    $\phi \leftarrow \mathrm{Opt}(\phi, \nabla_\phi \mathcal{L}, \eta)$
until convergence of ϕ
~~**end for**~~

procedure INFER($\mathcal{D}_*$, M) ▷ *M is number of posterior samples*
    for $m = 1$ to M **do**
        Sample $z_m \sim q(z)$
        $\theta_m \leftarrow f_\phi^{-1}(z_m; \mathcal{D}_*)$
    end for
    return $\{\theta_m\}_{m=1}^M$
end procedure

5.10 Fig. S3, b,d: Missing y-label. For how many particles is the ancestor tree? C: title cut off.

Answer: We thank the reviewer for this observation and corrected it in the manuscript. The number of particles shown in the ancestor tree is the number used for all the experiments, 200 particles. This is now included in the figure caption.

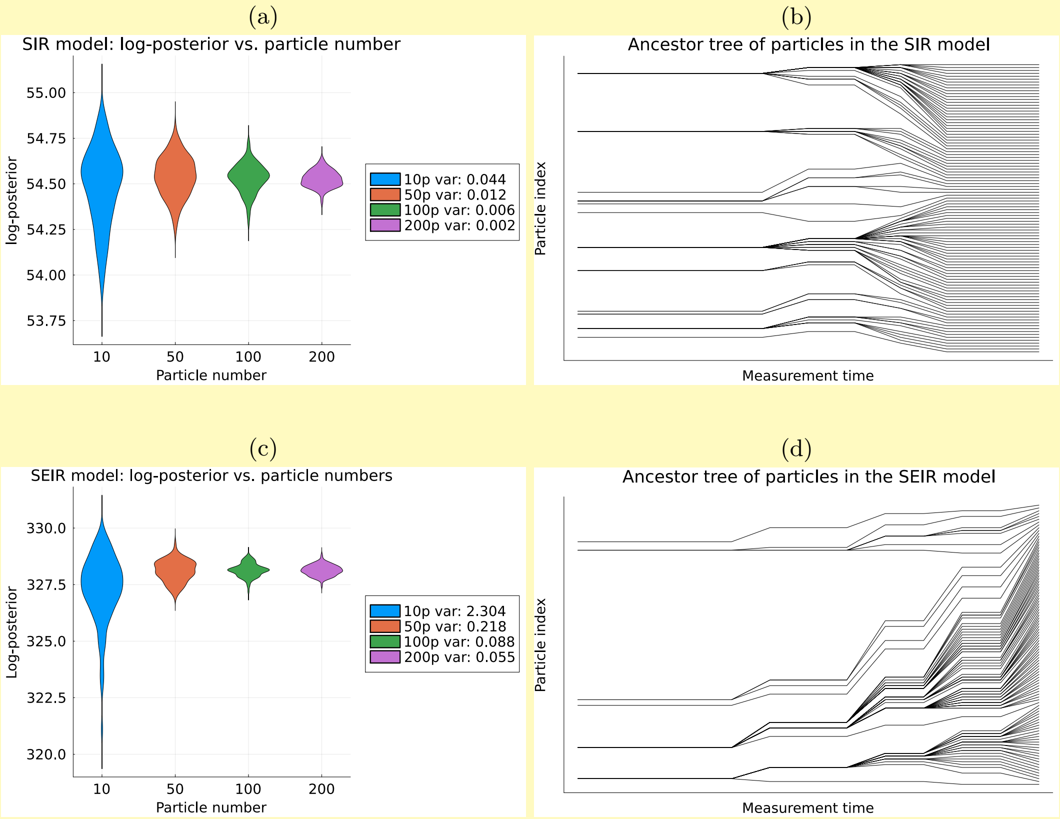

Figure S1.1: **Metrics for tuning of the particle number.** (a) and (c) visualize the variance of the log-likelihood estimate across different particle numbers for the SIR and SEIR model. (b) and (d) depict the ancestor trees of the 200 particles over time for the SIR and the SEIR model.

5.11 l. 355ff: CNF: the presented setting might be sub-optimal. Can the authors ensure that the network did already converge after 100 epochs? A latent dimension (dim of summary stats) of 2n+2 seems quite small, as the data (observations) are compressed into this dimension. Also, even a single Gaussian has $(n^2+n)/2$ free parameters. However, at this point it is unclear to me how large the observation space is for the different observational settings.

Answer: We thank the reviewer for raising these important points. In our conditional normalizing flow, the summary network serves only to condition the flow on the data; it does not need to represent the posterior distribution itself. The expressive power of the posterior approximation comes from the flow transformation, not from the dimensionality of the summary vector. The summary network therefore provides a compact context vector, while the flow learns the full nonlinear mapping from the base distribution to the posterior.
For this reason, the latent dimension of $2n+2$ should not be interpreted as a restriction on the posterior family. A model with n parameters does not require access to the full $(n^2+n)/2$-dimensional space of Gaussian covariances, because the true posterior occupies only a low-dimensional manifold within that space. The flow learns this man-

ifold directly through its coupling layers, which provide the necessary flexibility to represent non-Gaussian shapes and strong parameter correlations even when conditioned on a relatively small summary vector.
To ensure that the chosen summary dimension was not a bottleneck, we performed a parameter sweep over a range of summary dimensions. We did not observe any principled benefit from larger summary vectors in our setting, and the calibration results of this sweep are now included in the Supplementary Information. This supports the conclusion that the flow, rather than the summary dimension, is the primary source of flexibility in the posterior approximation.
Finally, we monitored training convergence and found that the loss stabilized well before 100 epochs; extending training did not improve validation likelihood or posterior diagnostics. We will clarify these points in the manuscript.

Changes to Section 4 To ensure comparability, all experiments used identical hyperparameters and simulation budgets~~, determined through a preliminary hyperparameter search~~. Rather than fine-tuning each method for every individual experiment, which would confound performance differences with tuning effort, we selected CNF hyperparameters through a principled search procedure that we repeated to assess stability. We verified that training was well-behaved by inspecting training and validation loss curves, which consistently stabilized well before 100 epochs. The dimension of the learned summary statistics was set to $2n_\theta + 2$, where n_θ denotes the number of model parameters. Training was conducted offline with 100,000 simulations for the training dataset and 400 simulations for validation. Models were trained for 100 epochs with a batch size of 32. Summary statistics were extracted using a SequenceNetwork [25] with 64 LSTM units, and the conditional normalizing flow was modeled using an InvertibleNetwork with spline coupling and eight coupling layers, both implemented within the BayesFlow framework. Detailed configurations are provided in Supplementary Information S1.1.3.

5.12 l. 360 ff: Handling missing data: What is the reasoning of implementing an approach for missing and sparse data if the neural networks are only trained on this specific pattern? Or did I misunderstood l. 365? For similarly structured training and test data, no additional processing is necessary.

Answer: We thank the reviewer for the opportunity to clarify this point. Our goal in implementing the missing-data handling was to obtain a consistent and extensible representation of the two observation functions. We adopted the approach of [26], which augments the input with binary indicators marking the presence or absence of each entry. This encoding allows the neural network to condition on missingness patterns when needed and provides a unified representation when combining observation functions recorded at different time points.
In our specific experimental setup, only dimensional consistency between the two observation functions is strictly required. The amortization capability of the mask was not used here. For sparse datasets, the neural networks were trained exclusively on the particular missingness pattern present in the simulated data to ensure a fair comparison between PF and CNF and to avoid confounding method differences with amortization artifacts.
We nevertheless included the binary mask deliberately, as it enables rapid amortization for practitioners who may wish to apply our framework to datasets with heterogeneous or irregular missingness patterns. We revised the manuscript accordingly:

Changes to Section 4: Handling non-equidistant time points and missing data required a specialized strategy. We adopted the approach of [26], which augments the input with binary indicators marking the presence or absence of each entry. This encoding allows the neural network to account for missing values during inference and provides a consistent representation when combining observation functions recorded at different time points. In our setup, only the dimensional consistency of the two observation functions is strictly required; however, we included the binary mask deliberately to facilitate rapid amortization for practitioners who may wish to extend our framework. For the present experiments, this amortization capability was not used: for sparse datasets, the neural networks were trained solely on the specific missingness pattern present in the simulated data to ensure a fair comparison between PF and CNF and to avoid confounding method differences with amortization artifacts. For experiments with dense, equidistant data, this additional encoding was not applied.

5.13 l. 378: For CNF the MAP can directly be obtained from the trained network by maximizing the (log-) posterior probability, which is not introducing an additional approximation via kernels.

Answer: We agree with the reviewer that, for CNFs, the MAP can in principle be obtained directly by maximizing the learned log-posterior, without introducing an additional approximation such as kernel smoothing. In our study, however, we deliberately used the same MAP approximation method for all inference methods to ensure a fair and comparable evaluation across approaches. Using a unified MAP estimator avoids method-specific advantages and keeps the comparison focused on the quality of the posterior approximation itself.

5.14 l 402. Referring to (almost) all suppl. tables in one place doesn't make sense.

Answer: We thank the reviewer for raising this point. The supplement was separated into several Supporting Information files, as requested by the journal. We chose to create one file for each results section containing all corresponding tables and figures. These files are referenced in the places where it is beneficial for the understanding of the manuscript.

5.15 Section 5: It is not clear at this point what the dimension of the observation space is in the different settings

Answer: We thank the reviewer for pointing out this confusing point. In terms of the two observables, the setup is described in Section 2.4, and this is the same across all experiments. For a better overview, we now provide a more detailed description of the data generating processes including the precise number of observed timepoints for each setting in the Supplementary Information, see answer to point 4.

5.16 Fig. 2: Ground-truth parameter should also be included into the 2d marginal.

Answer: We thank the reviewer for pointing this out. We have included the ground-truth parameter and MAP values in the 2d marginals. An example is in the answer to comment 1, showcasing the result for the SIS model.

5.17 Fig. 4: it is common to also scale the 2d marginals to the prior bounds/ meaningful prior support regions. Otherwise the information gain across dimensions is hard to judge

Answer: We agree that plotting all 2-D marginals on the full prior range can make the absolute information gain easier to judge. In our case, however, posterior mass typically occupies only a very small fraction of the prior volume. Using a fixed prior-range scaling would therefore compress the posterior contours to the point where differences

between methods become difficult to see. For this reason, we used experiment-specific zoom levels that keep the relevant posterior structure visible. Since the focus of Fig. 4 and similiar plots is on the relative differences between the two inference methods, this choice provides a clearer visual comparison.

To get a grasp on how much information is gained, we refer to the histogram of posterior approxiamtions compared to the prior depicted in Figure 4, formerly Figure 3.

5.18 Suppl. Figures should be arranged according to the main text.

Answer: We thank the reviewer for his comment. Due to the size of the supplement and the guidelines of the journal, the supplement is now split into several files. Each results section has an own supplementary information file with the corresponding figures and tables supporting the reported results. This also leads to a consistent numbering of supplementary figures and tables in their order of appearance in the main manuscript.

5.19 L. 451: should reference to Fig. S25

Answer: We thank the reviewer and corrected the typo accordingly.

5.20 Fig. 6 "True parameter" is misleading. There is no true parameter here. What are the black uncertainty intervals in B? These are not defined.

Answer: We thank the reviewer for pointing is out. The term "true parameter" is indeed misleading. We changed this to "Publication Parameter" to make clear that we compare against the value from the reference publication. We also defined the uncertainty intervals in the figure caption.

Changes in Section 5: **Evaluation of the posterior approximations for the two-variant SEIR model with real data.**
A Posterior approximations based on 10,000 samples, ~~true parameters~~ publication parameters and their reported standard deviation are indicated by bold black lines and joint MAP estimates appear in a darker shade. **B** Model fit with percentiles (50%, 90%, 95%) based on simulating the 10,000 samples.

5.21 L. 616: for flow matching Wildberger et al. (2023) might be of interest for the authors
L. 558: There are already a bunch of SBI methods dealing with these flexible settings, e.g. Gloeckler et al. (2024) or Moss et al. (2023). While I included some references below, I don't expect the authors to cite them in their manuscript. But I want to make them aware of research related to their topic, which might be of interest.

Answer: We thank the reviewer for making us aware of these related research articles.

5.22 Supporting Tables need a thorough proofreading. There is a lot of missing/repeated information. They should also be arranged in the order as appearing in main.

Answer: We proofread and rearranged the supplementary information and all tables. Furthermore, we removed redundant information. For a better overview we do not include all tables here in the answer.

5.23 Tab. S20: "Dense 1-1" and "Dense 1-2" are the same, but information for "Dense 2-1" is missing?

Answer: Thank you for pointing out the incomplete table. We added more datasets and a complete table with all used data generating parameters

Changes in the Supplementary Information:

Table S1.17: Ground-truth parameter sets corresponding to the dense datasets for the full SEIR2V model.

**Set**	γ^{-1}	κ^{-1}	β	s	t_{var}	I_0
d-1-1	17.00	5.00	0.0800	3.00	150	500.0
d-1-2	17.00	5.00	0.0800	3.00	150	500.0
d-2-1	11.70	8.40	0.2300	1.60	222	560.0
d-2-2	11.70	8.40	0.2300	1.60	222	560.0
d-3	15.47	3.97	0.0655	8.40	226	477.3
d-4	11.57	6.11	0.1605	8.34	318	169.6
d-5	24.05	6.63	0.0423	6.45	122	789.9
d-6	22.21	4.37	0.0807	1.91	274	831.2
d-7	23.34	3.52	0.0631	6.43	329	79.0
d-8	16.66	3.35	0.0699	4.95	289	604.8
d-9	17.98	6.59	0.1430	3.55	317	859.7
d-10	16.77	5.57	0.1786	1.18	172	31.8
d-11	18.63	9.93	0.1807	1.07	180	157.8
d-12	8.51	3.80	0.1731	4.66	299	327.2

5.24 Table S15: all values are the same. What is the subsampling ratio?

Answer: We thank the reviewer for the question. Yes, all values were the same, since all these datasets were generated by simulating with the same parameter vector. They were only different realizations and with different missingness and sparsity pattern. We added more details on the data generation and naming of datasets in the Supplementary Information S1.3, see answer to comment 4. There we also clarified subsampling processes for the sparse data generation. The subsampling ratio was around 1/7 for each observable, since we subsampled 8 timepoints out of 56 per observable. This generates between 8 and 16 timepoints, were we observe one or both observables.

5.25 Table S27: What are the different priors referring to? The computation time comparison is not fair, as different resources are used.

Answer: We thank for mentioning this. The different prior columns were artifacts from internal experimental runs and were removed. For the comparison of the methods we used the same machine and resource constraints in terms of CPU power and memory. However, the CNF is better parallelizable and could therefore use all available CPU kernels at once, which is one reason for the faster inference. We see this as a difference between the inference methods and not as a difference in computational resources.

Table S27: Comparison of computation times: Accumulated CPU-time in minutes for both methods applied to a dense dataset. For the PF we ran 4 chains on 4 cores in parallel, the CNF was parallelized over 20 cores.

Datasets	PF	CNF
SIR 1	1163.33	106.72
Dense 1-1	2095.98	252.96
Sparse 1-1-1	1798.1	132.29

5.26 Table S3: what are the upper and lower bounds for beta? Why are they dependent on gamma and why is the prior then Unif(0,1)? Please clarify.

Answer: We thank the reviewer for pointing out the ambiguity in the presentation of the priors. Our intention was to use uninformed priors, but in the considered models a large portion of the naïve prior support leads to unrealistic or uninformative trajectories. In particular, the effective reproduction number $R_0 = \beta\gamma^{-1}$ becomes either extremely small or extremely large for many combinations of β and γ^{-1}, which results in trajectories that are either almost flat or explode unrealistically. To avoid placing prior mass on such pathological regimes, we imposed simple constraints on β that depend on γ^{-1}, ensuring that R_0 remains within a plausible epidemiological range.
The prior is therefore written as Unif(0,1) to indicate that we do not impose any additional shape or information beyond these feasibility constraints; the effective bounds shown in Table S3 arise solely from enforcing biologically reasonable values of R_0.
For the SEIR2V model, we additionally restrained γ^{-1} to be greater than one for numerical stability. This is also consistent with domain knowledge, we deemed an infectious period shorter than one day as epidemiologically implausible.
We clarify this in the manuscript as shown in the answer to comment 4.

5.27

1. results solely rely on two (!) parameter settings for each model.
2. l. 381: RMSE is used in parameter and data space. While this is fine conceptually (but see comments above), the authors should make this more explicit.
3. SBC plots are just provided in the supplement without e.g. explaining what ECDF means. SBC is clearly off for SIR, but in different ways for different parameters. Why? There seems to be an oscillating behavior in the SBC plots, why? A deeper analysis is missing here.
4. RMSE should include some measure of uncertainty, e.g. std. or quantiles.

Answer: We thank the reviewer for stressing these points. We refer to Answers in comments 1. and 2. Additionally, since we replaced the RMSE with the energy score of the posterior predictive distribution, there is no intrinsic uncertainty measure associated with the energy score itself. Instead, the uncertainty arises solely from its numerical estimation. We expanded the Supplementary Information to include a short introduction on metrics and diagnostics used.

Changes in Supplementary Information:

S1.2 Metrics and Diagnostics

This section summarizes the metrics and diagnostics used to evaluate inference quality across models and datasets.

S1.2.1 1-Wasserstein distance in log-parameter space

To quantify discrepancies between inferred and true parameter distributions, we compute the 1-Wasserstein distance [27] between posterior samples of two methods. The distance is evaluated in log-parameter space to account for multiplicative scales and to avoid domination by parameters spanning several orders of magnitude.
The 1-Wasserstein distance has a simple heuristic interpretation: it measures the average amount of probability mass that must be moved to transform one posterior distribution into the other. A value of d means that, on average, posterior mass lies roughly a factor of $\exp(d)$ away from the second posterior.
Since the Wasserstein distance is computed from finite posterior samples, it inherits a non-negligible Monte Carlo error. This requires the usage of a proper baseline (e.g., the distance between two independent posterior draws from the same method), which reflects the intrinsic Monte Carlo noise of the estimator.

S1.2.2 Energy score of the posterior predictive distribution

Predictive performance is assessed using the energy score, a proper scoring rule for multivariate predictive distributions [28]. For each dataset, we draw 10,000 posterior predictive trajectories and compute the energy score using its standard pairwise formulation.
The pairwise term involves 10^8 trajectory pairs, which is computationally expensive. We therefore approximate it by subsampling 10,000 random pairs, which yields an unbiased Monte-Carlo estimate.
Monte Carlo error: The variance of the pairwise term decreases as $1/M$, where M is the number of sampled pairs. With $M = 10,000$, the Monte-Carlo standard error is typically well below the scale of variation across datasets, and we verified empirically that increasing M does not materially change the ranking or magnitude of energy scores. This provides a reliable and efficient approximation.

S1.2.3 $\hat{\mathbf{R}}$

The $\hat{\mathbf{R}}$ statistic measures convergence of MCMC chains by comparing within-chain to between-chain variance, where values below 1.01 indicate well mixed chains [29]. Intuitively this means that the chains have "forgotten" their initial values and therefore both variances are close to each other. To mitigate incorrect diagnoses of convergence failures for chains with heavy tails or varying variance across chains, we use an improved version using rank-normalization, folding and localization [30].

S1.2.4 Effective Sample Size

To measure the quality and uncertainty of the resulting posterior we report the effective sample size (ESS). The ESS quantifies, how much effectively independent information the chains contain and is defined in terms of a pooled autocorrelation estimate combining within-chain and between-chain information [30].

S1.2.5 Simulation-based calibration (SBC)

We assess calibration using simulation-based calibration. For each synthetic dataset, we compute (via the bayesflow package [16]):

- **Rank histograms**, obtained by ranking the true parameter among posterior samples
- **Empirical Cumulative Distribution Function (ECDF)** curves, comparing the empirical distribution of ranks to the uniform distribution.

Well-calibrated inference yields uniform rank histograms and ECDFs lying within the expected sampling envelope.

S1.2.6 Parameter-recovery plots

For conditional normalizing flows, we visualize parameter recovery by plotting posterior means and medians against the true parameters across all datasets using Bayesflow's plotting functionalities [16]. These plots provide a direct diagnostic of bias, shrinkage, and systematic distortions in the learned posterior.

References

[1] Hoffman MD, Gelman A, et al. The No-U-Turn sampler: adaptively setting path lengths in Hamiltonian Monte Carlo. J Mach Learn Res. 2014;15(1):1593–1623.

[2] Roberts GO. On inference for partially observed nonlinear diffusion models using the Metropolis-Hastings algorithm. Biometrika. 2001;88(3):603–621. doi:10.1093/biomet/88.3.603.

[3] Golightly A, Wilkinson DJ. Bayesian inference for stochastic kinetic models using a diffusion approximation. Biometrics. 2005;61(3):781–788. doi:10.1111/j.1541-0420.2005.00345.x.

[4] Papaspiliopoulos O, Roberts GO, Stramer O. Data Augmentation for Diffusions. Journal of Computational and Graphical Statistics. 2013;22(3):665–688. doi:10.1080/10618600.2013.783484.

[5] Dragatz C. Bayesian inference for diffusion processes. Fakultät für Mathematik, Informatik und Statistik der Ludwig-Maximilians-Universität München; 2010. doi:10.5282/edoc.12136.

[6] Whitaker GA, Golightly A, Boys RJ, Sherlock C. Improved bridge constructs for stochastic differential equations. Statistics and Computing. 2015;doi:10.1007/s11222-016-9660-3.

[7] van der Meulen F, Schauer M. Bayesian estimation of discretely observed multi-dimensional diffusion processes using guided proposals. Electronic Journal of Statistics. 2017;doi:10.1214/17-EJS1290.

[8] Fjelde TE, Xu K, Widmann D, Tarek M, Pfiffer C, Trapp M, et al. Turing.jl: a general-purpose probabilistic programming language. ACM Transactions on Probabilistic Machine Learning. 2025;.

[9] Kermack WO, McKendrick AG, Walker GT. A contribution to the mathematical theory of epidemics. Proceedings of the Royal Society of London, Series A. 1927;115(772):700–721. doi:10.1098/rspa.1927.0118.

[10] Silverman BW. Density estimation for statistics and data analysis. Routledge; 1998. doi:10.1201/9781315140919.

[11] Gudina EK, Ali S, Girma E, Gize A, Tegene B, Hundie GB, et al. Seroepidemiology and model-based prediction of SARS CoV 2 in Ethiopia: longitudinal cohort study among front-line hospital workers and communities. The Lancet Global Health. 2021;9(11):e1517–e1527. doi:10.1016/S2214-109X(21)00386-7.

[12] Wang Y, Blei D, Cunningham JP. Posterior collapse and latent variable non-identifiability. Advances in neural information processing systems. 2021;34:5443–5455. doi:10.48550/arXiv.2301.00537.

[13] Winkler C, Worrall D, Hoogeboom E, Welling M. Learning Likelihoods with Conditional Normalizing Flows. arXiv preprint. 2019;doi:10.48550/arXiv.1912.00042.

[14] Talts S, Betancourt M, Simpson D, Vehtari A, Gelman A. Validating Bayesian inference algorithms with simulation-based calibration. arXiv preprint. 2018;doi:10.48550/arXiv.1804.06788.

[15] Radev ST, Mertens UK, Voss A, Ardizzone L, Köthe U. BayesFlow: Learning complex stochastic models with invertible neural networks. IEEE transactions on neural networks and learning systems. 2020;33(4):1452–1466. doi:10.1109/TNNLS.2020.3042395.

[16] Radev ST, Schmitt M, Schumacher L, Elsemüller L, Pratz V, Schälte Y, et al.. BayesFlow: Amortized Bayesian Workflows With Neural Networks; 2023.

[17] Walker HM, Helen M. De Moivre on the law of normal probability. Smith, David Eugene A Source Book in Mathematics, Dover. 1985; p. 64690–4.

[18] Cranmer K, Brehmer J, Louppe G. The frontier of simulation-based inference. Proceedings of the National Academy of Sciences. 2020;117(48):30055–30062. doi:10.1073/pnas.1912789117.

[19] Papamakarios G, Murray I. Fast ε-free inference of simulation models with Bayesian conditional density estimation. In: Proceedings of the 30th International Conference on Neural Information Processing Systems. NIPS'16. Red Hook, NY, USA: Curran Associates Inc.; 2016. p. 1036–1044.

[20] Papamakarios G, Nalisnick E, Rezende DJ, Mohamed S, Lakshminarayanan B. Normalizing Flows for Probabilistic Modeling and Inference. Journal of Machine Learning Research. 2021;22(57):1–64. doi:10.48550/arXiv.1912.02762.

[21] Rezende D, Mohamed S. Variational inference with normalizing flows. In: International conference on machine learning. PMLR; 2015. p. 1530–1538.

[22] Boelts J, Deistler M, Gloeckler M, Álvaro Tejero-Cantero, Lueckmann JM, Moss G, et al. sbi reloaded: a toolkit for simulation-based inference workflows. Journal of Open Source Software. 2025;10(108):7754. doi:10.21105/joss.07754.

[23] Dragatz C. A Diffusion Approximation for an Epidemic Model; 2006.

[24] Golightly A, Wilkinson DJ. Bayesian parameter inference for stochastic biochemical network models using particle Markov chain Monte Carlo. Interface Focus. 2011;1(6):807–820. doi:10.1098/rsfs.2011.0047.

[25] Radev ST, Graw F, Chen S, Mutters NT, Eichel VM, Bärnighausen T, et al. OutbreakFlow: Model-based Bayesian inference of disease outbreak dynamics with invertible neural networks and its application to the COVID-19 pandemics in Germany. PLoS computational biology. 2021;17(10):e1009472. doi:10.1371/journal.pcbi.1009472.

[26] Wang Z, Hasenauer J, Schälte Y. Missing data in amortized simulation-based neural posterior estimation. PLOS Computational Biology. 2024;20(6):e1012184. doi:10.1371/journal.pcbi.1012184.

[27] Villani C, et al. Optimal transport: old and new. vol. 338. Springer; 2009. doi:https://doi.org/10.1007/978-3-540-71050-9.

[28] Gneiting T, Raftery AE. Strictly proper scoring rules, prediction, and estimation. Journal of the American statistical Association. 2007;102(477):359–378. doi:https://doi.org/10.1198/016214506000001437.

[29] Gelman A, Rubin DB. Inference from iterative simulation using multiple sequences. Statistical science. 1992;7(4):457–472. doi:10.1214/ss/1177011136.

[30] Vehtari A, Gelman A, Simpson D, Carpenter B, Bürkner PC. Rank-normalization, folding, and localization: An improved $\hat{R}$ for assessing convergence of MCMC (with discussion). Bayesian analysis. 2021;16(2). doi:10.1214/20-BA1221.
