# Supplementary material for: Assessment of simulation-based inference methods for stochastic compartmental models in epidemiological research
Source: PLoS One. 2026 Jul 13;21(7):e0353306. doi: 10.1371/journal.pone.0353306 (PMC13362117; doi:10.1371/journal.pone.0353306)
Supplement: Attachment — Submitted filename: review_report.pdf [file pone.0353306.s011.pdf]

# Review Report

**"Assessment of Simulation-Based Inference Methods for Stochastic Compartmental Models in Epidemiological Research"**
by V. Wieland et al. (2025)

The authors have addressed most of the concerns raised in the previous round of review. However, several issues remain and require clarification:

1. **Page 2, lines 25–26**
   PMCMC is not strictly likelihood-free, as it relies on an unbiased likelihood estimator. Conversely, CNFs can be likelihood-based depending on their formulation. Therefore, referring to both approaches as "likelihood-free" is technically questionable and should be reconsidered.

2. **Page 6, line 163**
   There is an inconsistency between the rate ($\beta SI$) presented in the text and the corresponding rate reported in Table 1.

3. **Page 7, Observation model**
   The statement that the first entry "describes the count data from testing" is misleading. In the model formulation, $y_1(t_i)$ represents prevalence (a latent model quantity), not raw testing counts. In practice, testing data are typically discrete counts (e.g., number of positive tests out of the number tested), rather than direct observations of population-level prevalence.

   The current formulation implicitly assumes that testing data (expressed as a proportion) can be represented as a noisy observation of prevalence; however, this assumption is not explicitly justified and should be clarified.

4. **Line 291**
   Replace $Y_{t_j}$ with $y_{t_j}$ for consistency in notation.

5. **Algorithm 1, Step 14**
   The term should be written as $g_\theta(y_{t_i} \mid \ldots)$ rather than $g_\theta(X_{t_i}^{(n)} \mid \ldots)$.

6. **CNF likelihood clarification**
   The authors should clarify the meaning of $p(D \mid \theta)$ in the CNF framework. Specifically, is this intended to represent the exact likelihood? Additionally, it should be explained how the dataset $D$ is sampled under this formulation.

7. **Line 487**
   The phrase "excellent marginal coverage" should be defined more precisely, as its meaning is currently unclear.

8. **Lines 507 and 552**
   Use appropriate cross-referencing (e.g., "see Supplementary Information, Section X") instead of plain text references.

9. **Figures (2B, 3B, 5B, 6B, 7B, and supplementary material)**
   The y-axis is labeled as infectious counts; however, it appears to represent proportions rather than counts. This should be corrected for accuracy.

10. **Figure 6**
    The label "CNF_2" is unclear. The authors should clarify its meaning and consider adjusting the color scheme to avoid confusion with PF.

11. **Figure 7 legend**
    The term "True parameters" should be replaced with "Reference parameters" for greater precision.

12. **Line 670**
    Use a proper cross-reference to Supplementary Information (e.g., "see Supplementary Information, Section S8").
