# Supplementary material for: Assessment of simulation-based inference methods for stochastic compartmental models in epidemiological research
Source: PLoS One. 2026 Jul 13;21(7):e0353306. doi: 10.1371/journal.pone.0353306 (PMC13362117; doi:10.1371/journal.pone.0353306)
Supplement: Attachment — Submitted filename: review2.pdf [file pone.0353306.s012.pdf]

Review for "**Assessment of Simulation-based Inference Methods for Stochastic Compartmental Models in Epidemiological Research**"

Summary The revision addresses the main concerns from the first round. The most substantial changes are: the addition of an HMC-based reference posterior for the SIS and SIR models (via data augmentation and finely discretized SDE); an increase from 2–6 to 10–12 prior-sampled synthetic datasets per setting; the introduction of the SIS model as an additional benchmark; replacement of RMSE by the Wasserstein-1 distance in log-parameter space and the energy score for predictive evaluation; clarification of the per-experiment CNF training design; and a greatly expanded supplement with technical details.

General Comments The most important remaining issue is that no reference posterior exists for the SEIR2V model — the most complex and application-relevant setting. The authors explain clearly why exact-likelihood HMC is infeasible there, but this fundamental limitation is not adequately communicated in the main text. As it stands, statements such as "both methods provide accurate and robust inference capabilities" read as stronger than the evidence supports, since for SEIR2V the two methods can only be compared against each other. The Discussion or Results section should state this limitation explicitly.

Two outlier datasets (sis-2, sis-4) are excluded from the SIS aggregate analysis due to large Wasserstein distances. The authors note that visual differences remain minor for these cases, but no a priori exclusion criterion is given. It is unclear whether the outliers were identified before or after inspecting the distances. The authors should either include all datasets, report results with and without exclusion, or state a predefined criterion.

The CNF miscalibration in the SIR model is now better contextualized via comparison to HMC, but the SBC failure is still somewhat dismissed by pointing to predictive accuracy. For an inference methods paper the posterior is the primary target. The authors should at least note whether the miscalibration is consistent across all 12 SIR datasets and comment briefly on its likely cause.

For the SEIR2V model, ESS values remain below 1000 for several PF runs, two datasets did not yield converged chains (r-2, r-5), and a third (r-6) has $\hat{R} > 1.18$ in Table S7.3 — yet none of these are flagged when reporting comparative results. The real-data run also has ESS < 500 for γ^{-1} and β (Table S8.2), which warrants a note when interpreting those posteriors.

Some methodological limitations remain. The CNF amortization is restricted to the per-experiment level, forgoing the broader generalization that motivated the approach; the binary masking scheme implemented for missing data handling was deliberately not used in the sparse experiments, leaving its practical utility undemonstrated. These are not grounds for rejection — the paper makes a valuable contribution as a proof-of-concept comparison of two inference paradigms in a realistic epidemiological setting - however, it is still lacking a well grounded systematic comparison. I recommend acceptance after a minor revision addressing the points above.

Specific Remarks:

1. The limitation that no HMC baseline exists for SEIR2V should be stated explicitly in the main text, not only in the supplementary methods.

2. The exclusion of sis-2 and sis-4 needs a stated, a priori criterion or results should be reported with and without these datasets.

3. The SBC analysis for the SIR model should comment on whether the miscalibration is consistent across all 12 datasets and give a brief mechanistic account.

4. Datasets where PF chains did not converge (r-2, r-5) or where $\hat{R} > 1.1$ (r-6: $\hat{R} \approx 1.19$, Table S7.3) should be flagged explicitly when reporting SEIR2V comparative results; the real-data run likewise has ESS < 500 for γ^{-1} and β (Table S8.2) and should be noted.

5. The ~10× speedup claim should note in the main text that CNF used 20 cores vs. 4 for PF, so the figure reflects parallelism as much as algorithmic efficiency.

6. Fig. 4 zooms posteriors to the posterior range, making it impossible to judge how much they have narrowed relative to the prior. A supplementary figure on the full prior scale would make this assessable.

7. The supplement shows both rank histograms and ECDF difference plots for every setting. These represent the same information; ECDF plots with simultaneous confidence bands (Säilynoja et al. 2022) are sufficient and their sole use would substantially shorten the supplement without any loss of diagnostic information.

8. The reparametrization (Section 5.4) fixes $\kappa^{-1} = 5$, reducing the parameter space from six to five dimensions. This is a model change, not a pure coordinate transformation. The improved CNF–PF alignment observed under reparametrization may partly reflect this simplification rather than the reparametrization per se. The authors should clarify this when interpreting Section 5.4 results.

9. Section 4 justifies per-experiment CNF training by stating that amortizing over continuously varying noise levels is infeasible, implying global amortization is impossible. This is not accurate: conditioning a normalizing flow on a continuous nuisance parameter is standard practice. The Discussion (lines 718–721) gives the correct account — broader amortization is possible but requires careful conditioning on metadata such as noise scales and time-grid structure. Section 4 should be revised to be consistent with the Discussion.

10. The color scheme used throughout the figures is problematic. In Fig. 6, CNF and PF posteriors are rendered as filled pdfs, making them impossible to distinguish. The same issue recurs across Figs. 2–7 and the supplementary posterior plots: filled density areas of two methods overlap and merge visually. Replacing filled densities with outlines, or using a colorblind-friendly palette with higher contrast, would substantially improve readability. Additionally, Fig. 7A caption states that the reported standard deviation of the publication parameters is indicated, but no uncertainty band is visible in the figure.
