# Supplementary material for: Assessment of simulation-based inference methods for stochastic compartmental models in epidemiological research
Source: PLoS One. 2026 Jul 13;21(7):e0353306. doi: 10.1371/journal.pone.0353306 (PMC13362117; doi:10.1371/journal.pone.0353306)
Supplement: Attachment — Submitted filename: Response_to_Reviewers_auresp_2.pdf [file pone.0353306.s014.pdf]

Author's response to reviewers' comments

The authors thank the reviewers and the editor for their constructive comments. Based on their remarks, we have updated the manuscript. We addressed all of their comments and tried to resolve the raised issues. The following points were changed in the manuscript: The manuscript was revised to address the reviewers' technical, methodological, and presentation-related comments. We clarified the terminology around likelihood evaluation and simulation-based inference, refined the observation model and notation, and made the limitations of the SEIR2V analysis more explicit, in particular the absence of a reference posterior for this model. We also revised the interpretation of convergence diagnostics, SBC results, outlier datasets, computational speedups, and the reparametrized model. In addition, we substantially improved the figures by correcting axis labels, replacing overlapping filled densities with contour-based posterior visualizations, using a colourblind-friendly scheme, and revising captions for clarity. Finally, we corrected typographical, grammatical, and cross-referencing issues throughout the manuscript and supplementary material.

- We clarified the terminology around likelihood evaluation and simulation-based inference.
- We made the limitations of the SEIR2V analysis more explicit, in particular the absence of a reference posterior.
- We revised the interpretation of convergence diagnostics, SBC results, outlier datasets, computational speedups and the reparametrization.
- We explained the observation model more thoroughly and made the observed quantities more precise.
- We improved the figures by replacing overlapped filled densities and using a colourblind-friendly scheme.
-

Furthermore, we polished the manuscript by correcting typographical, cross-referencing and and grammatical issues that were found during the revision.

Our point-by-point responses to the single points raised by the reviewers are provided below.

- Response to Reviewer 1
- Response to Reviewer 2

The teal boxes contain the reviewers' comments, while the yellow boxes contain the changes made in the manuscript. Within the yellow boxes, we have marked the changed part in red. To provide some context, the comments (teal) are surrounded by unchanged text (black).

Response to Reviewer 1

Comment 1: Page 2, lines 25–26:
PMCMC is not strictly likelihood-free, as it relies on an unbiased likelihood estimator. Conversely, CNFs can be likelihood-based depending on their formulation. Therefore, referring to both approaches as "likelihood-free" is technically questionable and should be reconsidered.

Answer:

We thank the reviewer for pointing out this misclassifications. The reviewer is right, that PMCMC uses an unbiased estimate of the likelihood, as we mentioned in the beginning of the Abstract and both methods at least implicitly assume that a likelihood can be defined and be sampled from for the model. The expression "likelihood-free" was more referring to the capabilities of the methods that one does not need to be able to directly evaluate the likelihood. We changed the wording to be more precise.

Changes in the manuscript (Abstract)

Addressing the challenges of intractable likelihoods for parameter inference in stochastic settings, our analysis highlights how ~~the likelihood-free methods~~ particle-filter-based likelihood estimation and flow-based posterior approximation can provide accurate and robust inference capabilities.

Comment 2: Page 6, line 163:
There is an inconsistency between the rate (βSI) presented in the text and the corresponding rate reported in Table 1.

Comment 4: line 281:
Replace Y_{t_j} with y_{t_j} for consistency in notation.

Comment 5: Algorithm 1, Step 14:
The term should be written as $g_\theta(y_{t_i} \mid \ldots)$ rather than $g_\theta(X_{t_i}^{(n)} \mid \ldots)$.

Answer:

The authors thank the reviewer for the thorough check and noting the typos and notational inconsistencies. All of them are fixed in the manuscript.

Changes in the manuscript (line 163):

Following simple mass-action incidence individuals get infected with a rate βSI $\beta\frac{SI}{N}$ and leave the infective class at rate γI...

Changes in the manuscript (line 281):

... and then those new particles are filtered based on their agreement with the data at hand $g_\theta(Y_{t_j} \mid X_{t_j})$ $g_\theta(y_{t_j} \mid X_{t_j})$, where the function g_θ is determined by the observation and noise model.

Changes in the manuscript (Algorithm 1, step 14):

$w_{t_i}^{(n)} \leftarrow \hat{w}_{t_{i-1}}^{(n)}$ $g_\theta(X_{t_i}^{(n)} \mid X_{t_{i-1}}^{(A_{t_i}^{(n)})})$ $g_\theta(y_{t_i} \mid X_{t_i})$

Comment 3: Page 7, Observation model:
The statement that the first entry "describes the count data from testing" is misleading. In the model formulation, $y_1(t_i)$ represents prevalence (a latent model quantity), not raw testing counts. In practice, testing data are typically discrete counts (e.g., number of positive tests out of the number tested), rather than direct observations of population-level prevalence.
The current formulation implicitly assumes that testing data (expressed as a proportion) can be represented as a noisy observation of prevalence; however, this assumption is not explicitly justified and should be clarified

Answer:

We thank the reviewer for this important clarification. We agree that the previous wording was misleading because the model quantity $y_1(t_i)$ represents latent prevalence rather than raw testing counts. We have revised the manuscript to make clear that we use the observational model under the assumption of a representative tested cohort, viewing the testing-derived data as noisy observations of the population prevalence. Additionally, we made the truncation of the observations clearer.

Changes in the manuscript (Section 2.4):

where the first entry is the ratio of currently infected people describing the count data from testing, and the second entry describes seroprevalence, the ratio of individuals with antibodies present due to a prior infection. where the first entry represents the latent prevalence of currently infected individuals. In practice, this quantity is not observed directly but is inferred from testing data. One method of inference is to consider the proportion of positive tests among all tests performed, under the assumption that the cohort tested is representative of the population as a whole. The observation model therefore treats testing-derived prevalence as noisy observations of the underlying population prevalence given by the model. The second entry represents seroprevalence, i.e., the proportion of individuals with antibodies due to a prior infection. Because the observation process may have several limitations, such as incomplete reporting or under-ascertainment, an additional scaling parameter s is used to capture that only a fraction of cases is reportedto account for the fact that only a fraction of infections may be captured in the reported testing data [1].

...

where we truncate $\overline{y}_1(t_i)$ and $\overline{y}_2(t_i)$ at a lower bound of 0 and at an upper bound of 1 to avoid unrealistic negative observations

Comment 6: CNF likelihood clarification:
The authors should clarify the meaning of $p(D \mid \theta)$ in the CNF framework. Specifically, is this intended to represent the exact likelihood? Additionally, it should be explained how the dataset D is sampled under this formulation.

Answer: We thank the reviewer for pointing this out. In this case, $p(D \mid \theta)$ is the implicit likelihood defined via the chosen simulator. We have amended the manuscript as follows.

Changes in the manuscript (Section 3.2):

Neural posterior estimation (NPE) is a simulation-based inference (SBI) method which frames posterior construction as a (conditional) density-estimation problem [2]. Given a simulator that produces samples $(\mathcal{D}, \theta)$ by first drawing $\theta \sim p(\theta)$ and then $\mathcal{D} \sim p(\mathcal{D} \mid \theta)$, NPE fits a conditional neural network $p_\phi(\theta \mid \mathcal{D})$ to approximate the true posterior $p(\theta \mid \mathcal{D})$. Here $p(\mathcal{D} \mid \theta)$ denotes the implicit likelihood defined by the stochastic forward model. It is the distribution over datasets induced by running the forward model at a fixed parameter θ. NPE sidesteps exact likelihood evaluation by relying only on these simulated samples.

Comment 7: line 487:
The phrase "excellent marginal coverage" should be defined more precisely, as its meaning is currently unclear.

Answer: We appreciate this observation and replaced "excellent marginal coverage" by what it is concretely, the share of true parameters lying in the respective 95% credible intervals.

Changes in the manuscript (Section 5.1):

We evaluated convergence and posterior estimates for the SIS model using CNF, PF, and a Hamiltonian Monte Carlo (HMC) baseline applied to a finely discretized version of the SDE. Across 10 synthetic dense datasets, all methods achieved excellent marginal coverage: the true parameters fell within the 95% credible interval for every posterior marginal (100% marginal coverage) ~~excellent marginal coverage~~ and produced highly consistent Wasserstein distances.

Changes in the manuscript (Section 5.2):

~~Across all datasets, PF and CNF achieved 83.33% marginal coverage and HMC achieved 87.5%, and the three posterior approximations showed high agreement overall. M~~Across all datasets, CNF and PF achieved 83.33% coverage of the 95% credible interval and HMC achieved 87.5% coverage. The three posterior approximations showed high agreement overall: marginal and joint distributions overlapped closely (Figure 3A), with CNF exhibiting a small systematic offset relative to HMC and PF.

Changes in the manuscript (Section 5.3):

Despite these width differences, both methods generally include the true parameters in their posteriors, with CNF achieving 95.2% ~~marginal~~ coverage of the 95% credible interval and PF achieving 91.7% coverage.

Comment 8: lines 507 and 552:
Use appropriate cross-referencing (e.g., "see Supplementary Information, Section X") instead of plain text references.

Comment 12: line 670:
Use a proper cross-reference to Supplementary Information (e.g., "see Supplementary Information, Section S8").

Answer: Thank you very much for making us aware of the plain text references. We changed the referencing of whole supporting information files to also use cross-referencing and adhere to the names of the files as outlined in the PLOS One guidelines.

Changes in the manuscript (Section 5):

...in Supplementary Information ~~S2~~ S2 Results.

in Supplementary Information ~~S3~~ S3 Results

...in Supplementary Information ~~S4 (dense datasets)~~ S4 Results for the dense datasets and ~~S5 (sparse datasets)~~ Supplementary Information S5 Results for the sparse datasets.

...in Supplementary Information ~~S6 (reparametrized model)~~ S6 Results for the reparametrized model and Supplementary Information ~~S7 (full model with datasets for the reparametrized model)~~ S7 Results for the full model with datasets of the reparametrized model.

...in Supplementary Information ~~S8~~ S8 Results.

Comment 9: Figures (2B, 3B, 5B, 6B, 7B, and supplementary material):
The y-axis is labeled as infectious counts; however, it appears to represent proportions rather than counts. This should be corrected for accuracy.

Answer: We appreciate the reviewer raising this point. We adjusted the labels to say "Prevalence" and "Seroprevalence". Additionally, we changed the description in the main text to reflect this.

Changes in the manuscript (Section 2.4):

where the first entry ~~is the ratio of currently infected people describing the count data from testing, and the second entry describes seroprevalence, the ratio of individuals with antibodies present due to a prior infection.~~ where the first entry represents the latent prevalence of currently infected individuals. In practice, this quantity is not observed directly but is inferred from testing data. One method of inference is to consider the proportion of positive tests among all tests performed, under the assumption that the cohort tested is representative of the population as a whole. The observation model therefore treats testing-derived prevalence as noisy observations of the underlying population prevalence given by the model. The second entry represents seroprevalence, i.e., the proportion of individuals with antibodies due to a prior infection. Because the observation process may have several limitations, such as incomplete reporting or under-ascertainment, an additional scaling parameter s is used ~~to capture that only a fraction of cases is reported~~to account for the fact that only a fraction of infections may be captured in the reported testing data [1].

Comment 10: Figure 6:
The label “CNF 2” is unclear. The authors should clarify its meaning and consider adjusting the color scheme to avoid confusion with PF.

Answer: We thank the reviewer for pointing out this issue. In this case, we mistakenly included the CNF reruns in the figure, which are not part of the primary assessment but part of the Wasserstein-baseline analysis. We adjusted the figure to match the other figures. More broadly, we reworked all of the posterior figures throughout the manuscript to improve readability and address a colour-scheme concern:

- Filled posterior densities in the corner panels were replaced by contour outlines (50% and 95% credible regions), so overlapping methods no longer merge into indistinct filled areas.
- The colour palette was replaced by a colourblind-friendly scheme, which we validated with the `colorspacious` simulator across deuteranomaly, protanomaly, and tritanomaly to confirm the methods remain distinguishable.
- Credible levels are now encoded by line style (solid: 50%, dashed: 95%) and methods by colour, so the two are separable independently of hue.
- True/reference parameters and MAP estimates are shown as distinct marker shapes (star and circle) rather than relying on colour alone.

Together these changes ensure the methods are clearly distinguishable, including under colour vision deficiencies.

In the same vein, we also improved on the figure captions.

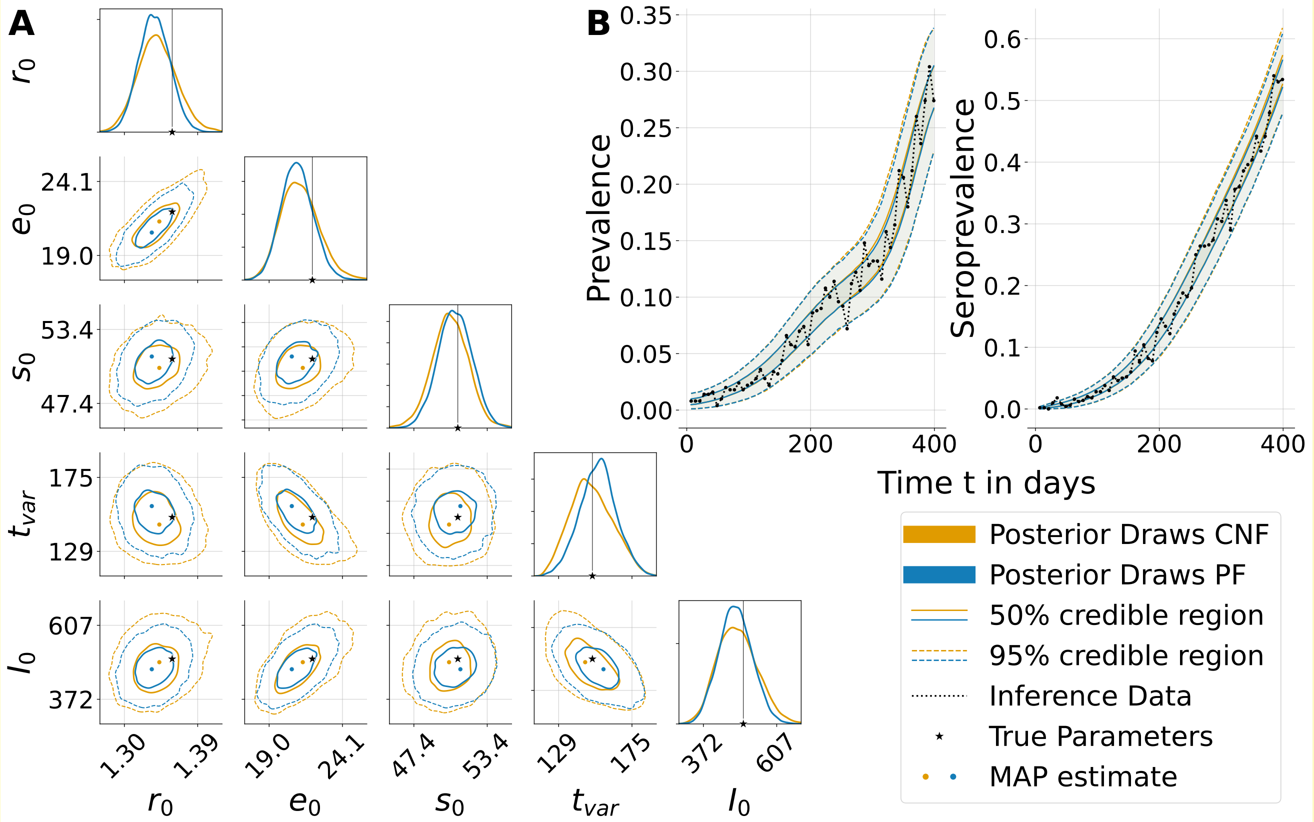

Figure 6: **Evaluation of the posterior approximations for the reparametrized two-variant SEIR model with dense data.**
A Posterior approximations from 10,000 samples. Contour gives the 50% (solid) and 95% (dashed) credible regions, coloured by method. Diagonals show the 1D marginals. Black stars mark the true parameters, coloured circles the joint MAP estimates. **B** Posterior predictive fit: bands give the 50% and 95% pointwise predictive intervals from the same samples (line styles as in **A**) with inference data shown as a dotted line.

Comment 11: Figure 7 legend
The term "True parameters" should be replaced with "Reference parameters" for greater precision.

Answer: We agree that "True parameters" should be replaced with "Reference parameters" and changed it accordingly in the figure.

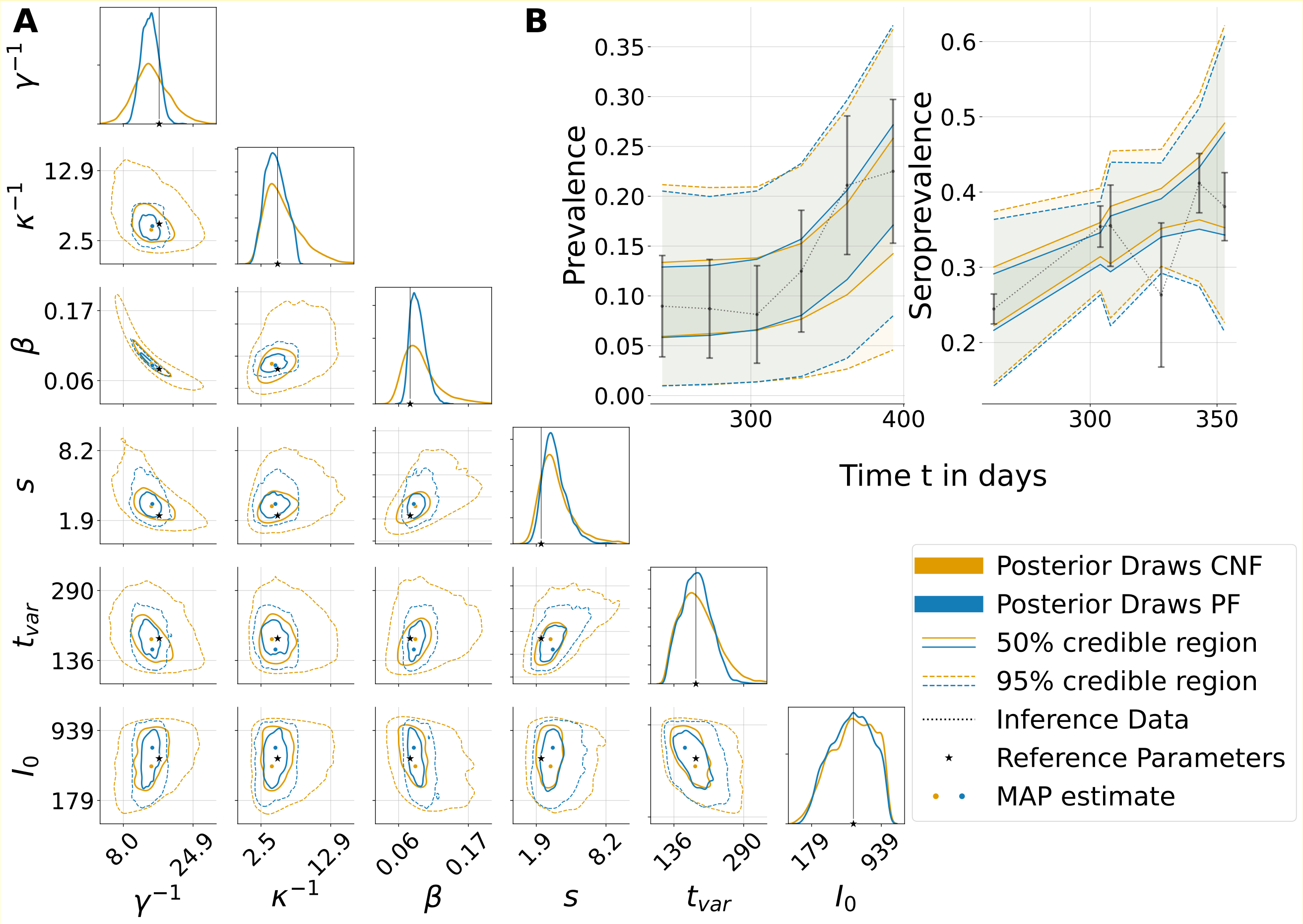

Figure 7: **Evaluation of the posterior approximations for the two-variant SEIR model with real data.** **A** Posterior approximations from 10,000 samples. Contours give the 50% (solid) and 95% (dashed) credible regions, coloured by method. Diagonals show the 1D marginals. Black stars mark the reference parameters, coloured circles the joint MAP estimates. **B** Posterior predictive fit: bands give the 50% and 95% pointwise predictive intervals from the same samples (line styles as in **A**). The inference data (published data from [1]) are shown as a dotted line with error bars.

Response to Reviewer 2

General Comments:
The most important remaining issue is that no reference posterior exists for the SEIR2V model — the most complex and application-relevant setting. The authors explain clearly why exact-likelihood HMC is infeasible there, but this fundamental limitation is not adequately communicated in the main text. As it stands, statements such as "both methods provide accurate and robust inference capabilities" read as stronger than the evidence supports, since for SEIR2V the two methods can only be compared against each other. The Discussion or Results section should state this limitation explicitly.
Two outlier datasets (sis-2, sis-4) are excluded from the SIS aggregate analysis due to large Wasserstein distances. The authors note that visual differences remain minor for these cases, but no a priori exclusion criterion is given. It is unclear whether the outliers were identified before or after inspecting the distances. The authors should either include all datasets, report results with and without exclusion, or state a predefined criterion.
The CNF miscalibration in the SIR model is now better contextualized via comparison to HMC, but the SBC failure is still somewhat dismissed by pointing to predictive accuracy. For an inference methods paper the posterior is the primary target. The authors should at least note whether the miscalibration is consistent across all 12 SIR datasets and comment briefly on its likely cause.
Two outlier datasets (sis-2, sis-4) are excluded from the SIS aggregate analysis due to large Wasserstein distances. The authors note that visual differences remain minor for these cases, but no a priori exclusion criterion is given. It is unclear whether the outliers were identified before or after inspecting the distances. The authors should either include all datasets, report results with and without exclusion, or state a predefined criterion.
For the SEIR2V model, ESS values remain below 1000 for several PF runs, two datasets did not yield converged chains (r-2, r-5), and a third (r-6) has $\hat{R} > 1.18$ in Table S7.3 — yet none of these are flagged when reporting comparative results. The real-data run also has ESS < 500 for γ^{-1} and β (Table S8.2), which warrants a note when interpreting those posteriors.
Some methodological limitations remain. The CNF amortization is restricted to the per-experiment level, forgoing the broader generalization that motivated the approach; the binary masking scheme implemented for missing data handling was deliberately not used in the sparse experiments, leaving its practical utility undemonstrated. These are not grounds for rejection — the paper makes a valuable contribution as a proof-of-concept comparison of two inference paradigms in a realistic epidemiological setting - however, it is still lacking a well grounded systematic comparison. I recommend acceptance after a minor revision addressing the points above.

Answer: The authors thank the reviewer for the further suggestions to improve the work. All points raised are addressed by the answers to the specific comments below.

Comment 1:
The limitation that no HMC baseline exists for SEIR2V should be stated explicitly in the main text, not only in the supplementary methods.

Answer: This is a very important point the reviewer raised. As explained in detail in the last answer letter and the supplementary information obtaining a reference distribution for the two-variant model was not possible. We now mentioned this more prominently in the introduction, results section and discussion of the main text.

Changes in the manuscript (Introduction):

For the more complex two-variant SEIR model the approximate computation of a reference posterior using HMC methods is not feasible, showcasing the need of the assessed Bayesian inference methods. Analyzing the agreement of the estimated marginal posteriors and model fits given by PF and CNF based on different synthetic datasets ~~for the two-variant SEIR model~~, including (partially) missing data, shows that both methods yield good fits to the data at hand.

Changes in the manuscript (Section 5):

For this model the computation of a reference posterior is not possible and we examine inference quality in terms of data fit and assess the differences of both methods.

Changes in the manuscript (Discussion):

Expanding the analysis, we applied both methods to a more complex stochastic two-variant SEIR model with practical non-identifiabilities and parameter couplings, that does not admit a reference posterior.

...

For the more complex two-variant SEIR model no reference posterior was available, leaving the assessment based on comparative observations between the two chosen methods.

Comment 2:
The exclusion of sis-2 and sis-4 needs a stated, a priori criterion or results should be reported with and without these datasets.

Answer: We thank the reviewer for pointing this out. We added the missing values and explained why each was set aside from the aggregate, for distinct reasons. For dataset 2, there is a real offset in the Wasserstein distances (in log parameter space) between CNF-HMC and PF-HMC, while CNF-PF remains small. For dataset 4, even the baseline HMC-HMC is notably elevated. All values are displayed visually in the supplementary information for the SIS model.

Changes in the manuscript (Section 5.1):

Throughout, each value pair gives the range (minimum to maximum) of the 1-Wasserstein distance in log-parameter space over subsampling repetitions, reflecting the Monte Carlo error of the procedure. Two datasets stood out for distinct reasons, identified by the in-distribution baseline (HMC-HMC), i.e. the distance of an HMC posterior to an independent HMC run on the same dataset. For sis-4, this baseline was itself elevated (0.116-0.214 vs. 0.056-0.119 across the remaining datasets), indicating an intrinsically dispersed posterior rather than a disagreement between methods. We therefore report it separately, as it inflates the between-method aggregate without reflecting method differences. For sis-2, the baseline was at the normal level (0.058-0.081) while only the HMC comparisons rose (0.175-0.199 for CNF-HMC and 0.204-0.230 for PF-HMC) and CNF-PF remained low (0.097-0.137), indicating a localized disagreement against HMC. We report aggregate between-method distances over the remaining datasets; for all cases, visual posterior differences remained minor. ~~Two datasets (sis-2 and sis-4) exhibited unusually large distances and were excluded from the aggregate analysis but even for these outliers, visual posterior differences remained minor.~~

~~After exclusion,~~Across the eight retained datasets, the in-distribution Monte Carlo baseline (HMC-HMC) was small, with values ranging from 0.056 to 0.119. Between-method distances were only moderately larger: CNF–PF ranged from 0.063 to 0.156, CNF–HMC ranged from 0.065 to 0.176 and PF–HMC ranged from 0.058 to 0.124. Interpreted on the log-parameter scale, these values correspond to average multiplicative shifts of roughly $6\% - 13\%$ for the baseline and $6\% - 17\%$, $7\% - 19\%$, and $6\% - 13\%$ for the between-method comparisons, respectively.

Comment 3:
The SBC analysis for the SIR model should comment on whether the miscalibration is consistent across all 12 datasets and give a brief mechanistic account.

Answer: We appreciate this suggestion. We examined the per-dataset SBC histograms and now report the consistency across the 12 datasets explicitly: the miscalibration in β is largely systematic (histograms predominantly sloping in the same direction), whereas γ^{-1} shows no single consistent shape. We added a brief mechanistic account, the consistency of the β bias points to a structural cause, most plausibly the constrained joint prior, rather than a dataset-specific artifact. A full diagnosis is beyond the scope of this comparison. We also clarify that this calibration gap does not propagate to parameter recovery, but we retain it as a genuine limitation of the amortized CNF since the posterior is the primary inferential target.

Changes in the manuscript (Section 5.2):

Beyond these direct comparisons, the methods differed in sampling quality. PF achieved excellent convergence, with ESS exceeding 2000 and $\hat{\mathbf{R}}$ values close to 1, indicating robust posterior exploration and well-mixed chains (Supplementary Tables S3.2 and S3.3). CNF, in contrast, showed deviations in SBC diagnostics (Supplementary Figure S3.14), reflecting limitations of the amortized posterior. For β, this miscalibration is largely consistent across all 12 datasets with SBC histograms predominantly sloping upwards. For γ^{-1}, the histograms did not show one consistent shape. The consistency of the β bias points to a structural rather than dataset-specific cause, likely rooted in the constrained joint prior, though we do not attempt a full diagnosis here. This miscalibration geometry matches the observed offset of CNF relative to PF and HMC: the upward-sloping β histogram corresponds to a left-shift in the β marginal.

Notably, these calibration issues did not carry over to parameter recovery, where recovered values aligned closely with the ground truth and error bars remained narrow (Supplementary Figure S3.16). Nonetheless, since the posterior is the primary inferential target, we regard this calibration gap as a genuine limitation of the amortized CNF here rather than a purely formal one, even though its impact on point estimates and predictive accuracy is minimal.

~~Notably, these calibration issues were not apparent in the parameter recovery plots: recovered parameters aligned closely with the ground truth and error bars remained narrow (Supplementary Figure S3.16). The miscalibration geometry also matches the observed offset of CNF relative to PF and HMC; for example, an upward-sloping SBC histogram for β corresponds to a left-shift in the β marginal. This highlights that while CNF's posterior uncertainty is imperfectly calibrated in a formal sense, the practical impact on point estimates and predictive accuracy is minimal.~~

Comment 4:
Datasets where PF chains did not converge (r-2, r-5) or where $\hat{R} > 1.1$ (r-6: $\hat{R} \approx 1.19$, Table S7.3) should be flagged explicitly when reporting SEIR2V comparative results; the real-data run likewise has ESS < 500 for γ^{-1} and β (Table S8.2) and should be noted.

Answer: The authors thank the reviewer for raising this point. Not all chains converged under the PMMH workflow in the most complex model we tried. We flagged this at the beginning of the corresponding results section .There, we now added a note on the $\hat{R}$ value for dataets $r-6$ and contextualized this behavior a bit more. Moreover, the low ESS are also marked for the model on real data in the respective results section.

Changes in the manuscript (Section 5.4):

In the full SEIR2V model setting, two datasets (*r-2* and *r-5*) did not yield converged Markov chains and one dataset (*r-6*) showed a high $\hat{R}$ value and small ESS for β and γ^{-1} under our PMMH workflow. This shows the challenges to achieve fast convergence and good mixing in MCMC based methods, if parameters are highly correlated due to unidentifiabilities in the model specification.

Changes in the manuscript (Section 5.5):

CNF exhibited good calibration alongside imperfect parameter recovery, while PF demonstrated convergence despite low effective sample sizes for β and γ^{-1}.

Changes in the manuscript (Discussion):

This adjustment resulted in better alignment between CNF and PF posterior estimates and markedly increased PF convergence and mixing.

Comment 5:
The $\sim 10\times$ speedup claim should note in the main text that CNF used 20 cores vs. 4 for PF, so the figure reflects parallelism as much as algorithmic efficiency.

Answer: Thank you very much for the suggestion. Indeed, the computational benefit of CNF is split between algorithmic efficiency and the capabilities to parallelize over more cores, both leading to faster computation times. This is exactly what we want to highlight here and now state this more explicitly.

Changes in the manuscript (Section 5.4):

Additionally, CNF are computationally more efficient and can be parallelized easily, while PF are restricted by their sequential nature and only one core per chain can be leveraged. Therefore, CNF were approximately 10-times faster, when parallelized to 20-cores compared to PF on 4-cores, independent of the used dataset (Supplementary Table S1.22).

Comment 6:
Fig. 4 zooms posteriors to the posterior range, making it impossible to judge how much they have narrowed relative to the prior. A supplementary figure on the full prior scale would make this assessable.

Answer: We thank the reviewer for this feedback. Figure 4 already covers the full prior range (shown in grey).

Comment 7:
The supplement shows both rank histograms and ECDF difference plots for every setting. These represent the same information; ECDF plots with simultaneous confidence bands (Säilynoja et al. 2022) are sufficient and their sole use would substantially shorten the supplement without any loss of diagnostic information.

Answer: We appreciate this suggestion. The reviewer is correct that the two diagnostics are derived from the same rank statistics, and that ECDF-difference plots with simultaneous confidence bands are sufficient to assess overall calibration. We retain both because, in our experience, they emphasize complementary aspects: the ECDF-difference plots are best suited to a global assessment of calibration adequacy, while the per-parameter rank histograms make the direction and shape of any miscalibration (e.g. over- vs. underdispersion, bias toward the prior) more immediately legible to readers less familiar with ECDF-band diagnostics.

Comment 8:
The reparametrization (Section 5.4) fixes $\kappa^{-1} = 5$, reducing the parameter space from six to five dimensions. This is a model change, not a pure coordinate transformation. The improved CNF–PF alignment observed under reparametrization may partly reflect this simplification rather than the reparametrization per se. The authors should clarify this when interpreting Section 5.4 results.

Answer: We thank the reviewer for this suggestion. Additional to the explanation of model parameter number reduction in the model section, we added an explicit clarification, when interpreting the results.

Changes in the manuscript (Section 5.4):

In summary, ~~reducing~~ removing parameter dependencies by reducing the number of estimated parameters improved identifiability and brought CNF and PF posterior shapes into closer agreement.
...
However, the reparametrization did not enhance predictive performance and relies on reducing the parameter space dimension by fixing κ^{-1}, which should be approached with caution in applied inference.

Comment 9:
Section 4 justifies per-experiment CNF training by stating that amortizing over continuously varying noise levels is infeasible, implying global amortization is impossible. This is not accurate: conditioning a normalizing flow on a continuous nuisance parameter is standard practice. The Discussion (lines 718–721) gives the correct account — broader amortization is possible but requires careful conditioning on metadata such as noise scales and time-grid structure. Section 4 should be revised to be consistent with the Discussion.

Answer: The reviewer is right that global amortization is possible and standard practice, and we thank them for pointing this out. Global amortization was out of scope of our comparison as it introduces another level of fine-tuning, wwhich would introduce an additional layer of design choices that could confound the comparison. We adjusted the manuscript as follows to make this clearer.

Changes in the manuscript (Section 4):

For CNF, posterior approximations were obtained via neural posterior estimation with learned summary statistics [3], using the BayesFlow software (v1) for amortized Bayesian workflows [4]. We trained an independent neural network for each experiment rather than a single globally amortized model. This preserves amortization over latent trajectories and parameter values within each experiment. Global amortization across experiments is possible in principle but doing so over continuous nuisance variables requires careful conditioning to remain reliable, and broadening the amortization scope was not necessary for our comparison. We therefore adopted per-experiment amortization, which isolates the methodological comparison from the additional design choices that broad amortization would introduce. The resulting per-experiment amortization still enables efficient SBC diagnostics and fast posterior evaluation once training is complete. ~~, while avoiding the need to amortize over continuously varying noise levels across experiments, a setting in which global amortization would be inefficient and unnecessary. The resulting per-experiment amortization still enables efficient SBC diagnostics and fast posterior evaluation once training is complete.~~

Comment 10:
The color scheme used throughout the figures is problematic. In Fig. 6, CNF and PF posteriors are rendered as filled pdfs, making them impossible to distinguish. The same issue recurs across Figs. 2–7 and the supplementary posterior plots: filled density areas of two methods overlap and merge visually. Replacing filled densities with outlines, or using a colorblind-friendly palette with higher contrast, would substantially improve readability. Additionally, Fig. 7A caption states that the reported standard deviation of the publication parameters is indicated, but no uncertainty band is visible in the figure.

Answer: We thank the reviewer for raising these issues. Filled densities indeed make overlapping methods hard to distinguish, and we have reworked the posterior figures throughout the manuscript accordingly:

- In the corner panels, filled posterior densities were replaced by contour outlines (50% and 95% credible regions), so overlapping methods no longer merge visually.
- The colour palette was replaced by a colourblind-friendly scheme, which we validated with the `colorspacious` simulator across deuteranomaly, protanomaly, and tritanomaly to confirm the methods remain distinguishable.
- Credible levels are now encoded by line style (solid: 50%, dashed: 95%) and methods by colour, so the two are separable independently of hue, and point estimates use distinct marker shapes (star for true/reference parameters, circle for MAP).

For the caption of Fig. 7, in panel A we removed the reference to a standard deviation, which was not displayed. The uncertainty of the reference data is instead shown as error bars in panel B, now noted in the caption.

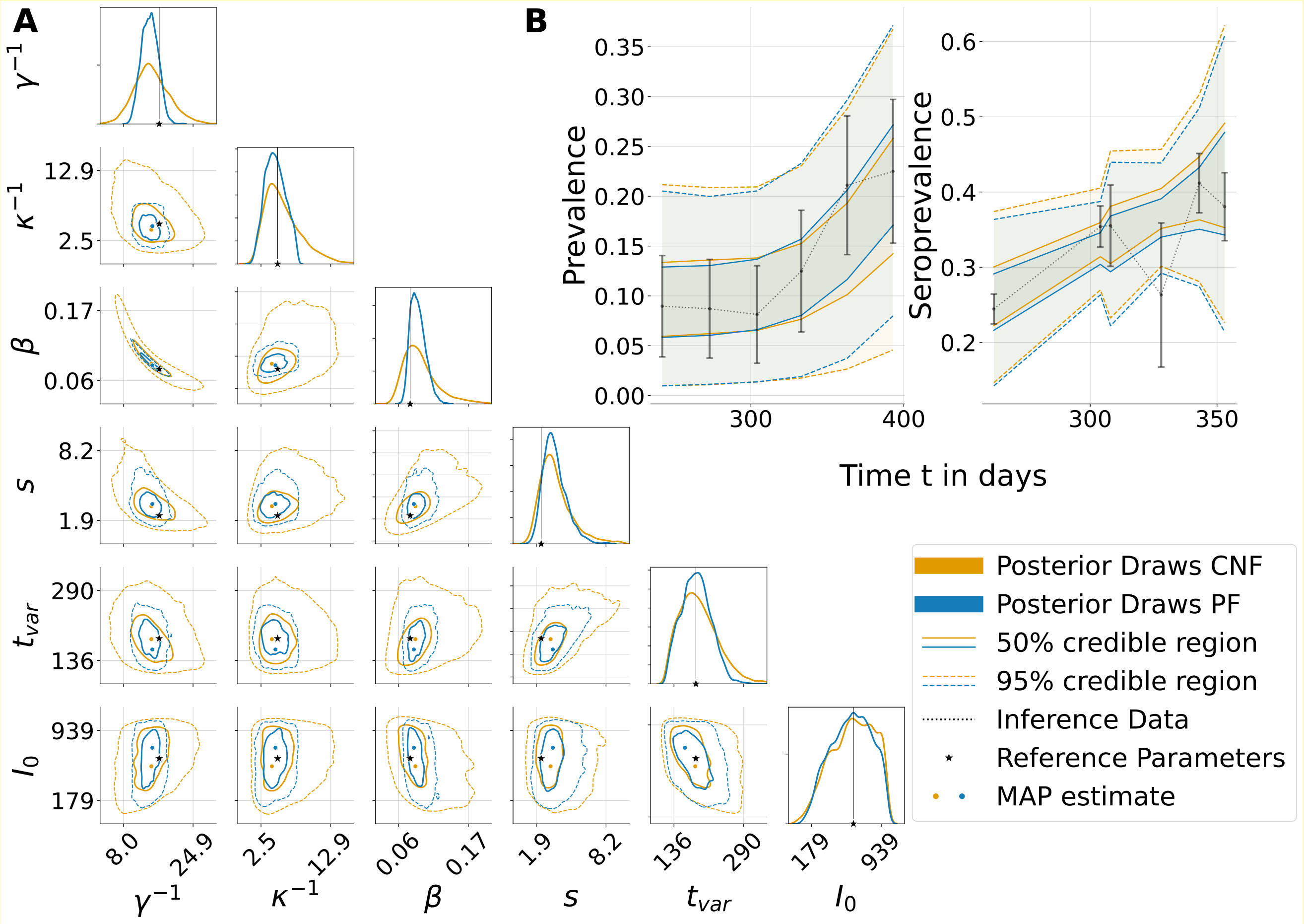

Figure 7: **Evaluation of the posterior approximations for the two-variant SEIR model with real data.** **A** Posterior approximations from 10,000 samples. Contours give the 50% (solid) and 95% (dashed) credible regions, coloured by method. Diagonals show the 1D marginals. Black stars mark the reference parameters, coloured circles the joint MAP estimates. **B** Posterior predictive fit: bands give the 50% and 95% pointwise predictive intervals from the same samples (line styles as in **A**). The inference data (published data from [1]) are shown as a dotted line with error bars.

Changes in the manuscript (Section 4):

This is complemented by a visual comparison of the model fit. For the evaluations we draw 10,000 parameter vector samples from each approximate posterior distribution and, for each parameter vector, simulate an observable trajectory to create an ensemble of independent posterior predictive samples obtained by evaluating $p(y \mid \theta)$ for draws $\theta \sim p(\theta \mid \mathcal{D}^*)$. We then plot the central 50%~~, 90%,~~ and 95% intervals of the ensemble against the data. ~~These are independent posterior predictive samples, obtained by evaluating $p(y \mid \theta)$ for draws $\theta \sim p(\theta \mid \mathcal{D}^*)$.~~

References

[1] Gudina EK, Ali S, Girma E, Gize A, Tegene B, Hundie GB, et al. Seroepidemiology and model-based prediction of SARS CoV 2 in Ethiopia: longitudinal cohort study among front-line hospital workers and communities. The Lancet Global Health. 2021;9(11):e1517–e1527. doi:10.1016/S2214-109X(21)00386-7.

[2] Papamakarios G, Murray I. Fast ε-free inference of simulation models with Bayesian conditional density estimation. In: Proceedings of the 30th International Conference on Neural Information Processing Systems. NIPS'16. Red Hook, NY, USA: Curran Associates Inc.; 2016. p. 1036–1044.

[3] Radev ST, Mertens UK, Voss A, Ardizzone L, Köthe U. BayesFlow: Learning complex stochastic models with invertible neural networks. IEEE transactions on neural networks and learning systems. 2020;33(4):1452–1466. doi:10.1109/TNNLS.2020.3042395.

[4] Radev ST, Schmitt M, Schumacher L, Elsemüller L, Pratz V, Schälte Y, et al.. BayesFlow: Amortized Bayesian Workflows With Neural Networks; 2023.
