# Supplementary material for: Assessment of simulation-based inference methods for stochastic compartmental models in epidemiological research
Source: PLoS One. 2026 Jul 13;21(7):e0353306. doi: 10.1371/journal.pone.0353306 (PMC13362117; doi:10.1371/journal.pone.0353306)
Supplement: Peer review history

# Peer review: Decision Letter 0

**DOI:** 10.1371/journal.pone.0353306.r001

**Authors:** Sarafa Iyaniwura

2 Feb 2026

PONE-D-25-64393

Assessment of Simulation-based Inference Methods for Stochastic Compartmental Models

PLOS One

Dear Dr. Hasenauer,

Thank you for submitting your manuscript to PLOS ONE. After careful consideration, we feel that it has merit but does not fully meet PLOS ONE’s publication criteria as it currently stands. Therefore, we invite you to submit a revised version of the manuscript that addresses the points raised during the review process.

Please submit your revised manuscript by Mar 19 2026 11:59PM. If you will need more time than this to complete your revisions, please reply to this message or contact the journal office at plosone@plos.org. When you're ready to submit your revision, log on to https://www.editorialmanager.com/pone/ and select the 'Submissions Needing Revision' folder to locate your manuscript file.

Please include the following items when submitting your revised manuscript:

- A letter that responds to each point raised by the academic editor and reviewer(s). You should upload this letter as a separate file labeled 'Response to Reviewers'.
- A marked-up copy of your manuscript that highlights changes made to the original version. You should upload this as a separate file labeled 'Revised Manuscript with Track Changes'.
- An unmarked version of your revised paper without tracked changes. You should upload this as a separate file labeled 'Manuscript'.

If you would like to make changes to your financial disclosure, please include your updated statement in your cover letter. Guidelines for resubmitting your figure files are available below the reviewer comments at the end of this letter.

If applicable, we recommend that you deposit your laboratory protocols in protocols.io to enhance the reproducibility of your results. Protocols.io assigns your protocol its own identifier (DOI) so that it can be cited independently in the future. For instructions see: https://journals.plos.org/plosone/s/submission-guidelines#loc-laboratory-protocols. Additionally, PLOS ONE offers an option for publishing peer-reviewed Lab Protocol articles, which describe protocols hosted on protocols.io. Read more information on sharing protocols at https://plos.org/protocols?utm_medium=editorial-email&utm_source=authorletters&utm_campaign=protocols.

We look forward to receiving your revised manuscript.

Kind regards,

Sarafa A Iyaniwura, Ph.D.

Academic Editor

PLOS One

Journal Requirements:

When submitting your revision, we need you to address these additional requirements.

1. Please ensure that your manuscript meets PLOS ONE's style requirements, including those for file naming. The PLOS ONE style templates can be found at

https://journals.plos.org/plosone/s/file?id=wjVg/PLOSOne_formatting_sample_main_body.pdf and

https://journals.plos.org/plosone/s/file?id=ba62/PLOSOne_formatting_sample_title_authors_affiliations.pdf.

2. Please note that PLOS One has specific guidelines on code sharing for submissions in which author-generated code underpins the findings in the manuscript. In these cases, we expect all author-generated code to be made available without restrictions upon publication of the work. Please review our guidelines at https://journals.plos.org/plosone/s/materials-and-software-sharing#loc-sharing-code and ensure that your code is shared in a way that follows best practice and facilitates reproducibility and reuse.

3. Thank you for stating in your Funding Statement:

“This work was supported by the Deutsche Forschungsgemeinschaft (DFG, German Research under Germany’s Excellence Strategy (EXC 2151—390873048), by the European Union via ERC grant INTEGRATE (grant no 101126146) to J.H. and by the University of Bonn via the Bonn Center for Mathematical Life Sciences and the Schlegel Professorship of J.H.). This work received further funding from the Initiative and Networking Fund of the Helmholtz Association (grant agreement number KA1-Co-08, Project LOKI-Pandemics).”

Please provide an amended statement that declares *all* the funding or sources of support (whether external or internal to your organization) received during this study, as detailed online in our guide for authors at http://journals.plos.org/plosone/s/submit-now.  Please also include the statement “There was no additional external funding received for this study.” in your updated Funding Statement.

Please include your amended Funding Statement within your cover letter. We will change the online submission form on your behalf.

4. Please update your submission to use the PLOS LaTeX template. The template and more information on our requirements for LaTeX submissions can be found at http://journals.plos.org/plosone/s/latex.

5. Please include captions for your Supporting Information files at the end of your manuscript, and update any in-text citations to match accordingly. Please see our Supporting Information guidelines for more information: http://journals.plos.org/plosone/s/supporting-information.

6. If the reviewer comments include a recommendation to cite specific previously published works, please review and evaluate these publications to determine whether they are relevant and should be cited. There is no requirement to cite these works unless the editor has indicated otherwise.

Additional Editor Comments (if provided):

Although reviewer #2 suggests rejection, I believe the manuscript will benefit more from a revision. As a result, I gave it a major revision. Authors should address all the concerns raised by this reviewer, and use them to improve the quality of the manuscript. These are very important concerns they have highlighted and they need to be addressed before the manuscript can be accepted.

[Note: HTML markup is below. Please do not edit.]

Reviewers' comments:

Reviewer's Responses to Questions

**Comments to the Author**

1. Is the manuscript technically sound, and do the data support the conclusions?

The manuscript must describe a technically sound piece of scientific research with data that supports the conclusions. Experiments must have been conducted rigorously, with appropriate controls, replication, and sample sizes. The conclusions must be drawn appropriately based on the data presented.

Reviewer #1: Yes

Reviewer #2: No

**********

2. Has the statistical analysis been performed appropriately and rigorously?

Reviewer #1: Yes

Reviewer #2: No

**********

3. Have the authors made all data underlying the findings in their manuscript fully available?

The PLOS Data policy requires authors to make all data underlying the findings described in their manuscript fully available without restriction, with rare exception (please refer to the Data Availability Statement in the manuscript PDF file). The data should be provided as part of the manuscript or its supporting information, or deposited to a public repository. For example, in addition to summary statistics, the data points behind means, medians and variance measures should be available. If there are restrictions on publicly sharing data—e.g. participant privacy or use of data from a third party—those must be specified.

Reviewer #1: Yes

Reviewer #2: Yes

**********

4. Is the manuscript presented in an intelligible fashion and written in standard English?

PLOS ONE does not copyedit accepted manuscripts, so the language in submitted articles must be clear, correct, and unambiguous. Any typographical or grammatical errors should be corrected at revision, so please note any specific errors here.

Reviewer #1: Yes

Reviewer #2: Yes

**********

5. Review Comments to the Author

Please use the space provided to explain your answers to the questions above. You may also include additional comments for the author, including concerns about dual publication, research ethics, or publication ethics. (Please upload your review as an attachment if it exceeds 20,000 characters)

Reviewer #1: This paper compares the performance of two Bayesian inference methodologies: Particle Markov Chain Monte Carlo (PMCMC) and Conditional Normalizing Flows (CNF), for state and parameter estimation in stochastic compartmental models. The methods are evaluated through simulation studies as well as an application to data from the Ethiopian COVID-19 outbreak. The results demonstrate that both approaches can yield accurate estimates of key epidemiological parameters and effectively track disease dynamics, with PMCMC providing more accurate estimates, while CNF is computationally more efficient.

Overall, I find the paper to be well written, with results that are clearly presented and well motivated. I do, however, have several comments that I believe would further improve the clarity, correctness, and completeness of the manuscript.

Main comments:

1. Page 2, lines 78–79:

In pseudo-marginal MCMC (PMMH), particle filters are used to estimate the likelihood, not to propose parameters. The wording in this sentence should be revised accordingly.

2. Normal approximation of the Bernoulli distribution (A.2 Supplementary information):

How is the parameter p (success probability) chosen in the normal approximation used to compute the standard deviation in each case? It would be helpful to report the resulting standard deviation for each analysis conducted. Additionally, how do the authors handle numerical instability in situations where the case count takes the value zero?

3. Section 3.1 (Particle Markov Chain Monte Carlo Methods):

Although this section is entitled Particle Markov Chain Monte Carlo Methods, it mainly describes the particle filter, with only a brief mention of how it is embedded within the Metropolis–Hastings algorithm. The authors could improve clarity by including pseudo-code for the PMMH algorithm (with acceptance probability), explicitly showing how the likelihood is replaced by its unbiased estimate in Equation (5).

4. Algorithm 1, Step 6:

No information is given about the effective sample size (ESS), although this is discussed in the supplementary material. A brief explanation or reference in the main text would be helpful.

5. Algorithm 1, Step 7:

In the resampling step, the weights should be W_{t_{i-1}}, since W_{t_i} is not yet available at that stage.

6. RMSE results in Tables 5–6 and supplementary tables:

In some cases, the RMSE of incidence/prevalence computed using the known ground-truth parameters is higher than that obtained using PF or CNF estimates in the simulated data, which appears counterintuitive. Could the authors explain why this occurs? Additionally, how exactly is the RMSE computed when using the true parameters?

7. Figure 6 caption:

The term “true parameters” is not appropriate, as true parameter values are not available for real data. The authors could instead use “reference parameters”.

8. Page 18, lines 580–593 (computational time):

CPU times are only reported for the SIR model. Providing CPU times for the SEIR model (with two variants), both for simulated and real data, would further support the claims made in this paragraph.

9. Prior distribution for the SIR model (Table S3):

The prior specification for β is confusing. The table states that the prior is U(0,1), but also indicates a lower bound of 0.95 × γ. Since the lower bound of γ is 1, this would imply β ~ U(0.95,1). On the other hand, if the prior is truly U(0,1), it is unclear how a MAP (PF or CNF) estimate of approximately 0.1 (which is less than 0.95 × γ) is obtained. My guess is that the lower and upper bounds refer to the normal prior mentioned in Table S27. The authors should clearly define and reconcile these prior specifications.

Reviewer #2: [please see attached pdf for the full review. ]

Summary

The authors study parameter inference for stochastic epidemic models using two probabilistic approaches. Focusing on stochastic SIR and extended SEIR models, they assess how particle filtering (PF) and conditional normalizing flows (CNF) approximate the posterior distribution. The methods are first evaluated on synthetic data and subsequently applied to real-world epidemiological observations from Ethiopia.

General Comments

The authors do a good job in setting the stage and introducing the problem and explaining the different SIR models. However their evaluation lacks in depth and most claims are not supported by the presented analysis. Their whole evaluation is based on very few (2-6) synthetic observation. But, as they even shown in the parameter recovery plots (e.g. Fig. S24), performance can vary vastly for different parameter settings. This should be taken into account and all analysis should be performed on an adequate set of (synthetic) datasets, commonly chosen as simulations based on samples from the prior.

While benchmarking likelihood-free inference methods is challenging, there are nevertheless tools to investigate the results in more depth. For the SIR model, I assume that the likelihood is even tractable and standard MCMC approaches could serve as a baseline to which the full posterior can be compared to (e.g. in terms of KL divergence, or classifier-two-sample test, or other measures). The authors oversimplify the comparison by mainly reporting RMSE, which can be done for the posterior predictive, bur which falls short in the parameter space, where only the MAP is considered. While SBC is an appropriate measure, the authors don’t take the failure in the simple SIR modle properly into account, and again refer to the predictive performance. However, a correct posterior should be the target of the inverse modelling approach. The claims of to wide or narrow posteriors (e.g. l.540) is therefore not supported by the analysis, as they never compare against a proper reference posterior, and is more anecdotally based on very few observations. Same holds true for other claims and their interpretations in their result sections are questionable and not backed up by proper analysis.

On a conceptual level, the authors claim to use an amortized inference method (CNF), however they train it from scratch for every observation (if I understood it correctly). While this might make sense for different data shapes (dense vs sparse data), it is unclear why this is done even in same simulator and data settings.

On a technical levels, a lot of information is missing, e.g. details to subsample the sparse datasets (e.g. data shapes), exact usage of prior (and confusing “upper“ and “lower“ limits), the exact evaluation schemes, the reparametrization (which fixes on parameter (?) and therefore changes the problem) etc. Also CNFs are trained for a fixed, limited number of epochs with a small network capacity (in terms of number of summary statistics / hidden layer size). This can clearly decrease the performance and additional control experiments should be performed and the convergence of the networks investigated.

Overall, while it looks like the authors have built a nice set-up to solve the inverse problem for stochastic versions of the SIR model, their evaluation pipeline is limited and their claims are not properly backed-up by the presented experiments. Therefore I recommend to reject the paper.

Below I attach more specific remarks, which may help the authors to improve their manuscript.

**********

6. PLOS authors have the option to publish the peer review history of their article (what does this mean?). If published, this will include your full peer review and any attached files.

If you choose “no”, your identity will remain anonymous but your review may still be made public.

**Do you want your identity to be public for this peer review?**  For information about this choice, including consent withdrawal, please see our Privacy Policy.

Reviewer #1: No

Reviewer #2: No

**********

[NOTE: If reviewer comments were submitted as an attachment file, they will be attached to this email and accessible via the submission site. Please log into your account, locate the manuscript record, and check for the action link "View Attachments". If this link does not appear, there are no attachment files.]

To ensure your figures meet our technical requirements, please review our figure guidelines: https://journals.plos.org/plosone/s/figures

You may also use PLOS’s free figure tool, NAAS, to help you prepare publication quality figures: https://journals.plos.org/plosone/s/figures#loc-tools-for-figure-preparation.

NAAS will assess whether your figures meet our technical requirements by comparing each figure against our figure specifications.

Attachment Submitted filename: Review.pdf

# Author comment: Author response to Decision Letter 1

**DOI:** 10.1371/journal.pone.0353306.r002

9 Apr 2026

Dear Editor,

It is with great pleasure that we received the reviews and your feedback on our manuscript. We are happy that the topic is considered interesting and important. The points that needed a major revision were mainly the lack of a comparison to a baseline psoterior, more rigorous evaluation metrics and the insufficient number of synthetic datasets to support our results.

In our revised version we addressed this points thoroughly by a new subsection in the modelling and results part introducing a simple SIS-model, for which we then computed a baseline posterior using state-of-the-art HMC sampling. For the SIR-model we were able to compute the baseline posterior as well and assessed the performance of CNF and PF in comparison to the baseline psoterior for both models. Additionally, the number of datasets was increased by nearly 4-times using now10 synthetic datasets for each experimental setup. Evaluation is now based on the Wasserstein-1 distance to compare posterior distributions in the parameter space and the energy score as a proper scoring rule for posterior predictive checks. Moreover, we streamlined our results figures and added more details on the calibration of the methods. In addition, we concretized the title of the proposed manuscript to „Assessment of Simulation-based Inference Methods for Stochastic Compartmental Models in Epidemiological Research“.

We hope that these changes and additional parts improve the manuscript and find your approval.

Please, find a detailed answer to all the reviewers comments and changes made in the manusrcipt attached.

We kindly ask to include this amended Funding Statement as requested:

“This work was supported by the Deutsche Forschungsgemeinschaft (DFG, German Research under Germany’s Excellence Strategy (EXC 2151—390873048), by the European Union via ERC grant INTEGRATE (grant no 101126146) to J.H. and by the University of Bonn via the Bonn Center for Mathematical Life Sciences and the Schlegel Professorship

of J.H.). This work received further funding from the Initiative and Networking Fund of the Helmholtz Association (grant agreement number KA1-Co-08, Project LOKI-Pandemics). There was no additional external funding received for this study.”

Thank you for considering our revised submission.

Kind regards,

Attachment Submitted filename: Response to Reviewers.pdf

# Peer review: Decision Letter 1

**DOI:** 10.1371/journal.pone.0353306.r003

**Authors:** Sarafa Iyaniwura

11 May 2026

PONE-D-25-64393R1

Assessment of Simulation-based Inference Methods for Stochastic Compartmental Models in Epidemiological Research

PLOS One

Dear Dr. Hasenauer,

Thank you for submitting your manuscript to PLOS ONE. After careful consideration, we feel that it has merit but does not fully meet PLOS ONE’s publication criteria as it currently stands. Therefore, we invite you to submit a revised version of the manuscript that addresses the points raised during the review process.

Please submit your revised manuscript by Jun 25 2026 11:59PM. If you will need more time than this to complete your revisions, please reply to this message or contact the journal office at plosone@plos.org. When you're ready to submit your revision, log on to https://www.editorialmanager.com/pone/ and select the 'Submissions Needing Revision' folder to locate your manuscript file.

Please include the following items when submitting your revised manuscript:

- A letter that responds to each point raised by the academic editor and reviewer(s). You should upload this letter as a separate file labeled 'Response to Reviewers'.
- A marked-up copy of your manuscript that highlights changes made to the original version. You should upload this as a separate file labeled 'Revised Manuscript with Track Changes'.
- An unmarked version of your revised paper without tracked changes. You should upload this as a separate file labeled 'Manuscript'.

If you would like to make changes to your financial disclosure, please include your updated statement in your cover letter. Guidelines for resubmitting your figure files are available below the reviewer comments at the end of this letter.

If applicable, we recommend that you deposit your laboratory protocols in protocols.io to enhance the reproducibility of your results. Protocols.io assigns your protocol its own identifier (DOI) so that it can be cited independently in the future. For instructions see: https://journals.plos.org/plosone/s/submission-guidelines#loc-laboratory-protocols. Additionally, PLOS ONE offers an option for publishing peer-reviewed Lab Protocol articles, which describe protocols hosted on protocols.io. Read more information on sharing protocols at https://plos.org/protocols?utm_medium=editorial-email&utm_source=authorletters&utm_campaign=protocols.

As the corresponding author, your ORCID iD is verified in the submission system and will appear in the published article. PLOS supports the use of ORCID, and we encourage all coauthors to register for an ORCID iD and use it as well. Please encourage your coauthors to verify their ORCID iD within the submission system before final acceptance, as unverified ORCID iDs will not appear in the published article. *Only*  the individual author can complete the verification step; PLOS staff *cannot*  verify ORCID iDs on behalf of authors.

We look forward to receiving your revised manuscript.

Kind regards,

Sarafa A Iyaniwura, Ph.D.

Academic Editor

PLOS One

**Journal Requirements:**

1. If the reviewer comments include a recommendation to cite specific previously published works, please review and evaluate these publications to determine whether they are relevant and should be cited. There is no requirement to cite these works unless the editor has indicated otherwise.

2. Please review your reference list to ensure that it is complete and correct. If you have cited papers that have been retracted, please include the rationale for doing so in the manuscript text, or remove these references and replace them with relevant current references. Any changes to the reference list should be mentioned in the rebuttal letter that accompanies your revised manuscript. If you need to cite a retracted article, indicate the article’s retracted status in the References list and also include a citation and full reference for the retraction notice.

**Additional Editor Comments:**

Authors should address the concerns listed by the reviewers.

[Note: HTML markup is below. Please do not edit.]

Reviewers' comments:

Reviewer's Responses to Questions

**Comments to the Author**

1. If the authors have adequately addressed your comments raised in a previous round of review and you feel that this manuscript is now acceptable for publication, you may indicate that here to bypass the “Comments to the Author” section, enter your conflict of interest statement in the “Confidential to Editor” section, and submit your "Accept" recommendation.

Reviewer #1: (No Response)

Reviewer #2: (No Response)

**********

2. Is the manuscript technically sound, and do the data support the conclusions?

The manuscript must describe a technically sound piece of scientific research with data that supports the conclusions. Experiments must have been conducted rigorously, with appropriate controls, replication, and sample sizes. The conclusions must be drawn appropriately based on the data presented.

Reviewer #1: Yes

Reviewer #2: Partly

**********

3. Has the statistical analysis been performed appropriately and rigorously?

Reviewer #1: Yes

Reviewer #2: Yes

**********

4. Have the authors made all data underlying the findings in their manuscript fully available?

The PLOS Data policy requires authors to make all data underlying the findings described in their manuscript fully available without restriction, with rare exception (please refer to the Data Availability Statement in the manuscript PDF file). The data should be provided as part of the manuscript or its supporting information, or deposited to a public repository. For example, in addition to summary statistics, the data points behind means, medians and variance measures should be available. If there are restrictions on publicly sharing data—e.g. participant privacy or use of data from a third party—those must be specified.

Reviewer #1: Yes

Reviewer #2: Yes

**********

5. Is the manuscript presented in an intelligible fashion and written in standard English?

PLOS ONE does not copyedit accepted manuscripts, so the language in submitted articles must be clear, correct, and unambiguous. Any typographical or grammatical errors should be corrected at revision, so please note any specific errors here.

Reviewer #1: Yes

Reviewer #2: Yes

**********

6. Review Comments to the Author

Please use the space provided to explain your answers to the questions above. You may also include additional comments for the author, including concerns about dual publication, research ethics, or publication ethics. (Please upload your review as an attachment if it exceeds 20,000 characters)

**Reviewer #1:**  The authors have addressed most of the concerns raised in the previous round of review. However, several issues remain and require clarification (see attached file)

**Reviewer #2:**  Summary

The revision addresses the main concerns from the first round. The most substantial changes are: the addition of an HMC-based reference posterior for the SIS and SIR models (via data augmentation and finely discretized SDE); an increase from 2–6 to 10–12 prior-sampled synthetic datasets per setting; the introduction of the SIS model as an additional benchmark; replacement of RMSE by the Wasserstein-1 distance in log-parameter space and the energy score for predictive evaluation; clarification of the per-experiment CNF training design; and a greatly expanded supplement with technical details.

General Comments

The most important remaining issue is that no reference posterior exists for the SEIR2V model — the most complex and application-relevant setting. The authors explain clearly why exact-likelihood HMC is infeasible there, but this fundamental limitation is not adequately communicated in the main text. As it stands, statements such as "both methods provide accurate and robust inference capabilities" read as stronger than the evidence supports, since for SEIR2V the two methods can only be compared against each other. The Discussion or Results section should state this limitation explicitly.

Two outlier datasets (sis-2, sis-4) are excluded from the SIS aggregate analysis due to large Wasserstein distances. The authors note that visual differences remain minor for these cases, but no a priori exclusion criterion is given. It is unclear whether the outliers were identified before or after inspecting the distances. The authors should either include all datasets, report results with and without exclusion, or state a predefined criterion.

The CNF miscalibration in the SIR model is now better contextualized via comparison to HMC, but the SBC failure is still somewhat dismissed by pointing to predictive accuracy. For an inference methods paper the posterior is the primary target. The authors should at least note whether the miscalibration is consistent across all 12 SIR datasets and comment briefly on its likely cause.

For the SEIR2V model, ESS values remain below 1000 for several PF runs, two datasets did not yield converged chains (r-2, r-5), and a third (r-6) has R̂ > 1.18 in Table S7.3 — yet none of these are flagged when reporting comparative results. The real-data run also has ESS < 500 for γ⁻¹ and β (Table S8.2), which warrants a note when interpreting those posteriors.

Some methodological limitations remain. The CNF amortization is restricted to the per-experiment level, forgoing the broader generalization that motivated the approach; the binary masking scheme implemented for missing data handling was deliberately not used in the sparse experiments, leaving its practical utility undemonstrated. These are not grounds for rejection — the paper makes a valuable contribution as a proof-of-concept comparison of two inference paradigms in a realistic epidemiological setting - however, it is still lacking a well grounded systematic comparison. I recommend acceptance after a minor revision addressing the points above.

See also pdf for specific comments.

**********

7. PLOS authors have the option to publish the peer review history of their article (what does this mean?). If published, this will include your full peer review and any attached files.

If you choose “no”, your identity will remain anonymous but your review may still be made public.

**Do you want your identity to be public for this peer review?**  For information about this choice, including consent withdrawal, please see our Privacy Policy.

Reviewer #1: No

Reviewer #2: No

**********

[NOTE: If reviewer comments were submitted as an attachment file, they will be attached to this email and accessible via the submission site. Please log into your account, locate the manuscript record, and check for the action link "View Attachments". If this link does not appear, there are no attachment files.]

To ensure your figures meet our technical requirements, please review our figure guidelines: https://journals.plos.org/plosone/s/figures

You may also use PLOS’s free figure tool, NAAS, to help you prepare publication quality figures: https://journals.plos.org/plosone/s/figures#loc-tools-for-figure-preparation.

NAAS will assess whether your figures meet our technical requirements by comparing each figure against our figure specifications.

Attachment Submitted filename: review_report.pdf

Attachment Submitted filename: review2.pdf

# Author comment: Author response to Decision Letter 2

**DOI:** 10.1371/journal.pone.0353306.r004

10 Jun 2026

It is with great pleasure that we received the reviews and feedback on our revised manuscript. We are happy that most concerns from the first round were already resolved and the additional analysis was taken as a valuable improvement of the original paper.

The main revisions include improving the description of the observation model, correcting notational inconsistencies, and making the limitations of the SEIR2V analysis more explicit. In particular, we now state more clearly that no reference posterior is available for the SEIR2V model, so the corresponding results should be interpreted as a comparison between the two approximate inference methods rather than as validation against a reference posterior.

We also revised the discussion of the SIS and SIR analyses. For the SIS model, we now explain the treatment of the datasets that stood out in the Wasserstein-distance comparison. For the SIR model, we added a more explicit interpretation of the SBC miscalibration and its consistency across datasets. Furthermore, we now flag convergence and effective sample size limitations in the SEIR2V analyses more clearly, including for the real-data application.

In response to the reviewers’ comments on presentation, we reworked the posterior figures throughout the manuscript and supplementary material. Filled posterior densities were replaced by contour outlines, the colour scheme was changed to a colourblind-friendly palette, credible levels are now encoded by line style, and figure captions were revised for clarity. We also corrected figure labels, improved cross-references to the supplementary material, and fixed typographical and grammatical issues throughout the manuscript.

We hope that these changes improve the manuscript even further and find your approval.

Please, find a detailed answer to all the reviewers comments and changes made in the manuscript attached

Attachment Submitted filename: Response_to_Reviewers_auresp_2.pdf

# Peer review: Decision Letter 2

**DOI:** 10.1371/journal.pone.0353306.r005

**Authors:** Sarafa Iyaniwura

22 Jun 2026

Assessment of Simulation-based Inference Methods for Stochastic Compartmental Models in Epidemiological Research

PONE-D-25-64393R2

Dear Dr. Hasenauer,

We’re pleased to inform you that your manuscript has been judged scientifically suitable for publication and will be formally accepted for publication once it meets all outstanding technical requirements.

Within one week, you’ll receive an e-mail detailing the required amendments. When these have been addressed, you’ll receive a formal acceptance letter and your manuscript will be scheduled for publication.

An invoice will be generated when your article is formally accepted. Please note, if your institution has a publishing partnership with PLOS and your article meets the relevant criteria, all or part of your publication costs will be covered. Please make sure your user information is up-to-date by logging into Editorial Manager at Editorial Manager® and clicking the ‘Update My Information' link at the top of the page. For questions related to billing, please contact billing support.

If your institution or institutions have a press office, please notify them about your upcoming paper to help maximize its impact. If they’ll be preparing press materials, please inform our press team as soon as possible -- no later than 48 hours after receiving the formal acceptance. Your manuscript will remain under strict press embargo until 2 pm Eastern Time on the date of publication. For more information, please contact onepress@plos.org.

Kind regards,

Sarafa A Iyaniwura, Ph.D.

Academic Editor

PLOS One

Additional Editor Comments (optional):

Reviewers' comments:

Reviewer's Responses to Questions

**Comments to the Author**

1. If the authors have adequately addressed your comments raised in a previous round of review and you feel that this manuscript is now acceptable for publication, you may indicate that here to bypass the “Comments to the Author” section, enter your conflict of interest statement in the “Confidential to Editor” section, and submit your "Accept" recommendation.

Reviewer #1: All comments have been addressed

**********

2. Is the manuscript technically sound, and do the data support the conclusions?

The manuscript must describe a technically sound piece of scientific research with data that supports the conclusions. Experiments must have been conducted rigorously, with appropriate controls, replication, and sample sizes. The conclusions must be drawn appropriately based on the data presented.

Reviewer #1: Yes

**********

3. Has the statistical analysis been performed appropriately and rigorously?

Reviewer #1: Yes

**********

4. Have the authors made all data underlying the findings in their manuscript fully available?

The PLOS Data policy requires authors to make all data underlying the findings described in their manuscript fully available without restriction, with rare exception (please refer to the Data Availability Statement in the manuscript PDF file). The data should be provided as part of the manuscript or its supporting information, or deposited to a public repository. For example, in addition to summary statistics, the data points behind means, medians and variance measures should be available. If there are restrictions on publicly sharing data—e.g. participant privacy or use of data from a third party—those must be specified.

Reviewer #1: Yes

**********

5. Is the manuscript presented in an intelligible fashion and written in standard English?

PLOS ONE does not copyedit accepted manuscripts, so the language in submitted articles must be clear, correct, and unambiguous. Any typographical or grammatical errors should be corrected at revision, so please note any specific errors here.

Reviewer #1: Yes

**********

6. Review Comments to the Author

Please use the space provided to explain your answers to the questions above. You may also include additional comments for the author, including concerns about dual publication, research ethics, or publication ethics. (Please upload your review as an attachment if it exceeds 20,000 characters)

Reviewer #1: I thank the authors for adequately addressing all comments and making the corresponding revisions to the manuscript.

There is one minor issue remaining: in Step 14 of Algorithm 1, a particle index appears to be missing. The term should be written as X_{ti}^{(n)}

**********

7. PLOS authors have the option to publish the peer review history of their article (what does this mean?). If published, this will include your full peer review and any attached files.

If you choose “no”, your identity will remain anonymous but your review may still be made public.

**Do you want your identity to be public for this peer review?**  For information about this choice, including consent withdrawal, please see our Privacy Policy.

Reviewer #1: No

**********

# Editor report: Acceptance letter

**DOI:** 10.1371/journal.pone.0353306.r006

**Authors:** Sarafa Iyaniwura

PONE-D-25-64393R2

PLOS One

Dear Dr. Hasenauer,

I'm pleased to inform you that your manuscript has been deemed suitable for publication in PLOS One. Congratulations! Your manuscript is now being handed over to our production team.

At this stage, our production department will prepare your paper for publication. This includes ensuring the following:

* All references, tables, and figures are properly cited

* All relevant supporting information is included in the manuscript submission,

* There are no issues that prevent the paper from being properly typeset

You will receive further instructions from the production team, including instructions on how to review your proof when it is ready. Please keep in mind that we are working through a large volume of accepted articles, so please give us a few days to review your paper and let you know the next and final steps.

Lastly, if your institution or institutions have a press office, please let them know about your upcoming paper now to help maximize its impact. If they'll be preparing press materials, please inform our press team within the next 48 hours. Your manuscript will remain under strict press embargo until 2 pm Eastern Time on the date of publication. For more information, please contact onepress@plos.org.

You will receive an invoice from PLOS for your publication fee after your manuscript has reached the completed accept phase. If you receive an email requesting payment before acceptance or for any other service, this may be a phishing scheme. Learn how to identify phishing emails and protect your accounts at https://explore.plos.org/phishing.

If we can help with anything else, please email us at customercare@plos.org.

Thank you for submitting your work to PLOS ONE and supporting open access.

Kind regards,

PLOS ONE Editorial Office Staff

on behalf of

Dr. Sarafa A Iyaniwura

Academic Editor

PLOS One
